# Identification and Management of Pediatric Sepsis: A Medical Student Curricular Supplement for PICU and NICU Rotations

**DOI:** 10.15766/mep_2374-8265.11142

**Published:** 2021-04-23

**Authors:** Nicole B. Anderson, Mai-King Chan, Cristina Gutierrez, Kristi Kambestad, Valencia Walker

**Affiliations:** 1 Resident, Department of Pediatrics, University of California, Los Angeles, David Geffen School of Medicine; 2 Assistant Clinical Professor, Department of Pediatrics University of California, Los Angeles, David Geffen School of Medicine; 3 Clinical Neonatologist, Department of Pediatrics, Children's Hospital of Orange County; 4 Assistant Dean, Equity and Diversity Inclusion, University of California, Los Angeles, David Geffen School of Medicine

**Keywords:** Simulation, Flipped Classroom, Neonatal Sepsis, Neonatal/Pediatric Intensive Care Units, PICU, NICU

## Abstract

**Introduction:**

Medical students frequently report lack of confidence and skill in managing ill pediatric patients. We aimed to implement targeted learning interventions to address these knowledge gaps, specifically focusing on pediatric sepsis. Our objective was to create a curriculum to advance knowledge and confidence in identifying and managing pediatric sepsis.

**Methods:**

We designed this curriculum to augment medical student pediatric ICU (PICU) and neonatal ICU (NICU) rotations. We first emailed students a pretest and upon completion, we emailed students a series of brief educational videos. Students then participated in a simulation experience designed to assess their ability to diagnose and manage severe sepsis. We provided immediate debriefing after each session. Upon completion of the simulation experience, we emailed students a posttest (identical to the pretest). The pre-/posttest included multiple-choice questions to assess the students’ ability to recognize and manage pediatric sepsis and septic shock, as well as Likert-scale questions assessing confidence levels in diagnosis and management of pediatric sepsis. We performed paired Student *t* tests comparing knowledge-based question scores and Likert-scale results.

**Results:**

Of students, 40 enrolled in and 30 completed the curriculum between 2015 and 2020. When comparing pre- and posttest results, we found a significant improvement in knowledge scores (33% mean increase, 95% CI [22%-45%], *p* < .001) and confidence levels (mean increase in Likert scale score of 1.5, 95% CI [1.2-1.7], *p* < .001).

**Discussion:**

Results suggested that the curriculum advanced students’ knowledge scores and improved self-reported confidence in managing theoretical pediatric patients with sepsis.

## Educational Objectives

By the end of this curriculum, learners will be able to:

1. Identify and classify the stages of shock in the pediatric patient.
2. Recognize the common signs and symptoms associated with sepsis in the pediatric population.
3. Apply the Surviving Sepsis guidelines for management of severe sepsis and septic shock in pediatric patients.

## Introduction

Care of critically ill pediatric patients is a crucial component of pediatric training, but medical students and first-year residents frequently report lack of confidence and skill in managing critically ill pediatric patients. We believe targeted learning interventions are needed to address knowledge gaps in this area. Currently, the ACGME requires that pediatric trainees spend a minimum of 4 months in critical care rotations, which includes neonatal and pediatric intensive care.^1^ However, as there are no standardized intensive care requirements among medical schools, the medical student intensive care experience varies widely, with adult critical care rotations being more common than pediatric or neonatal critical care. Not only does this reduce exposure and interest in the fields of pediatric and neonatal critical care, it puts many pediatric trainees in a position of starting residency with no or minimal experience caring for critically ill pediatric patients. This leads to trainee anxiety and stress, and potential inappropriate management or harm to patients.

To address this, we developed a pediatric sepsis curriculum for medical students that utilized contemporary learning strategies, including self-study material, simulation-based training, and direct in-person feedback, with the goal of increasing knowledge and confidence in the identification and management of pediatric sepsis and septic shock among participants. We chose the topic of pediatric sepsis since it is among the most common serious conditions with which pediatric patients present to the hospital in the United States. The global burden of pediatric sepsis is significant, with a mortality rate up to 5% for sepsis and up to 20% for severe sepsis.^2^ These hospitalizations cost billions of dollars per year and are estimated to cost up to 12 times as much as other childhood hospitalizations.^3^ While some components of adult critical care medicine can be carried over to pediatrics, the care of critically ill infants and children is unique from adult medicine in many ways.^4^ Pediatric sepsis, in particular, is a disease state distinct from adult sepsis. Pediatric trainees must have a basic understanding of these differences in order to successfully care for infants and children presenting with sepsis.

Historically, didactic teaching has delivered passive acquisition of knowledge by learners. Recent educational innovations encourage independent and self-directed learning, and promote active acquisition of knowledge. The flipped classroom approach represents one of these innovative methods. In a flipped classroom, learners independently study and review relevant content for the educational topic. During the subsequent face-to-face session with an instructor, the learners are encouraged to utilize previously reviewed information collaboratively to solve case-based scenarios. This format encourages active cognitive processing through social interaction and lends itself to promoting teaching and learning efficacy in medical education. With our curriculum, participants completed online videos prior to participating in simulation sessions, which replicated a flipped classroom approach to learning.

Models utilizing simulation-based technology have been implemented at other institutions to teach resuscitation and emergency room care in the setting of septic shock to medical students and residents.^5–8^ However, we are not aware of a longitudinal learning experience specific to pediatric and neonatal sepsis which incorporated self-learning tools, in-person teaching, and simulation-based experience, which makes our curriculum somewhat unique.

We chose fourth-year medical students (MS 4s) rotating through the neonatal ICU (NICU) and pediatric ICU (PICU) as subjects due to their presumed interest in pediatrics, and their critical position at the cusp of the transition point from medical student learner to resident learner.

## Methods

### Development

This curriculum consists of a pre- and posttest, online educational videos, and a simulation experience, with incorporated feedback and teaching in a safe learning environment. We designed our curriculum to augment standard NICU and PICU rotations for MS 4s at a single institution, using elements of established conceptual frameworks, including Kern's six-step approach to curriculum development,^9^ the theory of deliberate practice,^10^ and logic modeling. We originally implemented the curriculum as a pilot in 2015, at which time our outcomes were promising and feedback from students was overwhelmingly positive, prompting us to continue the curriculum.

Educational material regarding pediatric sepsis is generally based on the definitions established by the International Pediatric Sepsis Consensus Conference in 2005,^11^ which includes terminology such as systemic inflammatory response syndrome (SIRS), sepsis, severe sepsis, and septic shock. While the newest Surviving Sepsis campaign pediatric guidelines were released in February of 2020,^12^ the updated pediatric sepsis definitions remain pending.^13^ These updated guidelines apply to all patients from birth (≥37 weeks gestational age) to 18 years of age with severe sepsis or septic shock.

The pre-/posttest includes knowledge questions based on the American Academy of Pediatrics’ Pediatrics Review and Education Program self-assessment question format. The question and curriculum content was originally administered based on 2005 Surviving Sepsis pediatric guidelines, however we have since updated the curriculum to reflect the 2020 Surviving Sepsis guidelines.^12^ Knowledge questions originally addressed the following severe sepsis and septic shock topics: (1) fluid resuscitation, (2) vasoactive drug choice, (3) antibiotic timing, (4) goal urine output, and (5) target hemoglobin levels. We have since updated questions 2, 4, and 5 to reflect the 2020 Surviving Sepsis guidelines,^12^ as the answers were either no longer true or no longer relevant. Question 2 still covers vasoactive drug choice, however we have updated it to reflect current guidelines, as epinephrine or norepinephrine rather than dopamine are now considered first-line vasoactive agents in septic shock. As goal urine output was not addressed in the 2020 Surviving Sepsis guidelines,^12^ we changed the topic of question 4 to appropriate broad-spectrum antibiotic choice in neonatal sepsis. Furthermore, the 2020 guidelines state that there are insufficient data to provide a hemoglobin transfusion threshold for unstable patients in shock, thus the topic of question 5 was changed to SIRS criteria. Thus, knowledge questions for those using this curriculum going forward address the following severe sepsis and septic shock topics: (1) fluid resuscitation, (2) vasoactive drug choice, (3) antibiotic timing, (4) neonatal antibiotic choice, and (5) SIRS criteria.

### Implementation

We invited MS 4s electively rotating through the institution's PICU and NICU from July 2015 through February 2020 to participate. In addition to clinical rounds and didactic lectures included in the standard 3-week ICU rotation, we emailed students, inviting them to participate in our supplemental curriculum. Participation was voluntary and students consented to partake in the curriculum. They were not obligated to complete the curriculum, although it was encouraged. We did not offer a financial or other incentive to participate.

At the beginning of their rotation, we emailed students a pretest (Appendix A) to complete via Survey Monkey. The pre-/posttest includes five multiple-choice questions to assess students’ knowledge of the diagnosis and appropriate management of sepsis and shock in pediatric patients, based on concepts from the 2020 Surviving Sepsis campaign. The pre-/posttest also includes three Likert-scale questions designed to assess confidence level in: (1) making a clinical diagnosis of shock, (2) managing ill pediatric patients, and (3) leading a pediatric code. Of note, we did not expect confidence level in leading a pediatric code to be particularly high amongst medical students before or after completing this curriculum; however, our goal was to contribute to advancement in confidence and comfort level in dealing with pediatric codes, not expertise.

Upon completion of the pretest, we emailed students links to four online video modules of PowerPoint presentations with voiceover commentary. Each module was approximately 8 to 12 minutes in duration. Due to copyright concerns in the videos, we instead provide readers with the topic PowerPoints along with the accompanying voiceover scripts. Instructors should record narrated PowerPoints using the provided slides and scripts for the introductory principles of the following topics:

1. Identifying pediatric shock (Appendices B and C).
2. Identifying pediatric sepsis (Appendices D and E).
3. Managing pediatric sepsis and septic shock (Appendices F and G).
4. Hemodynamics and pressor support (Appendices H and I).

During the second week of their rotation and after completion of the online modules, we invited students to participate in a 2-hour immersive mock-code simulation experience designed to assess their ability to diagnose and manage severe sepsis and septic shock. All students were excused from clinical duty for this activity and had already undergone an introduction to basic ICU procedures, such as bag-mask ventilation and intubation. We implemented three simulation scenarios (Appendices J, K, and L) with patient ages of 16 years, 4 years, and 6 months. All cases progressed to septic shock and included the same learning objectives, cues, and observable actions. We included at least two simulation scenarios in each session, with each simulated case lasting approximately 30 to 45 minutes. If time allowed, we performed all three cases. Following the simulations sessions, the instructor(s) administered an additional interactive didactic session provided via PowerPoint (Appendix M), designed to reinforce the learning points reviewed in the online modules.

#### Simulation equipment

We conducted simulations at the UCLA Simulation Center, using a simulation room with an adjacent control room equipped with a one-way window. We incorporated three interactive manikins, which correlated with the approximate age of the patient in each case. The manikins we used included the Laerdal SimMan 3G for the 16-year-old, and the Gaumard Pediatric HAL and Neonate HAL for the 4-year-old and 6-month-old, respectively. Continuous cardiorespiratory monitoring was available for all cases. We provided students with stethoscopes, blood pressure cuffs, electrocardiogram leads, IV supplies, oxygen, and airway and intubation equipment.

#### Simulation personnel

Personnel required for these sessions included one or more instructors, one or more simulator operators, someone to play the role of the nurse, and someone to play the role of the parent. Oftentimes, due to personnel shortages, the nurse and the parent were played by the same person. When possible, we had pediatric registered nurses certified in simulation training play the role of the nurse. Instructors were pediatric attending physicians, pediatric subspecialty fellows or pediatric residents certified to provide simulation training. They were responsible for simulation feedback, procedural skill review, and teaching. Instructors and the simulator operators viewed the students’ actions during the simulation via a one-way window. Simulator operators were trained simulation center staff members.

The parent gave the initial history and answered any follow-up questions related to the history of present illness. Students could ask the nurse to take vitals, place the patient on a monitor, provide equipment, place a nasal cannula or face mask, place IV access, administer medication, and draw labs. Lab results and clinical updates were provided to students by the nurse. We did not provide imaging results if imaging was requested. If students requested medications, the nurse would ask for dosing, if not provided, and verbalize medication administration. IV medications, including IV fluids, were not given until IV access was obtained. The nurse was wearing a headset through which instructors and simulation staff were able to relay communication. Instructors asked the nurse to provide clinical prompts for students when needed.

#### Simulation debriefing

Each simulation was followed by an immediate debriefing and feedback session prior to starting the next scenario. We reviewed educational objectives, with a focus on individual learners’ needs. Feedback was given using the ask-tell-ask method,^14^ which we believe fosters students’ abilities to identify their own strengths and weaknesses, as well as provides preceptors with opportunities to provide positive and constructive feedback to learners.

### Assessment

We assessed learner performance during the simulations based on an evaluation checklist we incorporated into the case summary document (Appendices J, K, and L). Learning objectives and educational strategies were informed by Kern's six-step approach to curriculum development.^12^ During the third and final week of their rotation, after completion of the simulation experience, we emailed students a link to the posttest (Appendix A). The posttest was identical to the pretest and was used to evaluate improvement in students’ knowledge and confidence levels in managing pediatric sepsis after curriculum completion.

We compared pre- and posttest answers to evaluate the impact of the curriculum. We used percent correct to measure the knowledge score, and the average of Likert-scale confidence scores (1 = *very uncomfortable*, 5 = *very comfortable*) to measure confidence level. Next, we performed paired Student *t* tests to compare pre- and posttest knowledge scores and confidence levels and reported mean differences with 95% CIs. As not all students completed the curriculum, not all students who took the pretest took the posttest. Thus, we performed paired Student *t* tests using matched unique identification numbers (last three digits of their phone number) on pre- and postknowledge scores among only those students who completed both a pre- and a posttest. All tests were two-sided, and *p* < .05 was considered statistically significant. Analyses were performed using Microsoft Excel and GraphPad. Our study was granted Institutional Review Board exemption (reference #18-001873; May 12, 2018).

## Results

Forty MS 4s rotating through either the NICU or the PICU began the curriculum, with 30 students (75%) completing the curriculum between 2015 and 2020. As MS 4 students could elect to take the NICU or PICU rotation at any time in the academic year, their experience and knowledge was variable prior to participation in the curriculum. Among participants who completed the curriculum (*n* = 30), there was a significant difference in pretest and posttest knowledge, with a mean increase in score of 33%, 95% CI (22%-45%), *p* < .001, as well as a significant difference in pretest and posttest confidence levels, with a mean increase in Likert scale score of 1.5, 95% CI (1.2-1.7), *p* < .001.

Among individual knowledge questions, questions 2 and 4 did not show a statistically significant improvement, although both showed trends toward improved scores (Table 1). Question 2, which was the most commonly missed question (*M*_pre_= 0.32, *M*_post_ = .50), was based on prior guidelines and related to vasoactive drug choice in “cold shock.” Question 4 addressed goal urine output for a pediatric patient in shock. As mentioned in the Methods section, we have since modified both questions 2 and 4 to reflect current guidelines and best practices (Appendix A). All other knowledge and confidence questions revealed a statistically significant improvement (Tables 1 and 2). Among the Likert-scale questions, the question assessing confidence in running a pediatric code was the lowest scoring answer, as we had anticipated given students’ level of training.

**Table 1.**
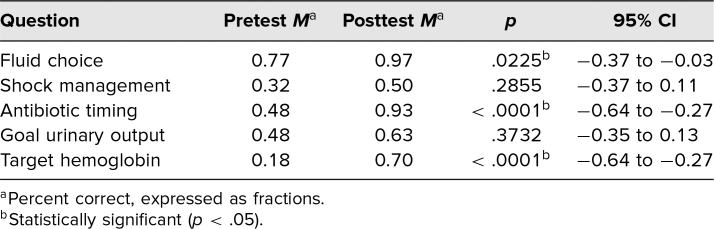
Mean Difference in Knowledge Question Scores Among Students Who Completed the Curriculum (*n* = 30)

**Table 2.**
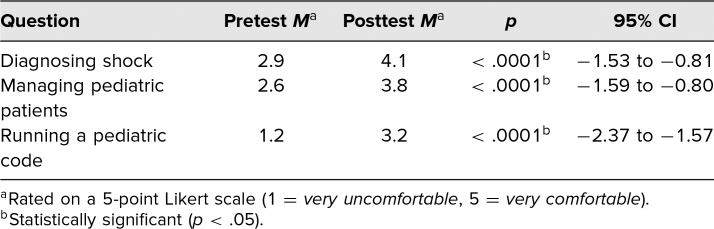
Mean Difference in Comfort Level Scores Among Students Who Completed the Curriculum (*n* =30)

## Discussion

To address the lack of established pediatric sepsis curricula designed to accompany medical student rotations, we developed a pediatric sepsis flipped classroom simulation designed to accompany MS 4 rotations in the NICU and PICU. We believe that this curriculum adds a valuable tool to the medical literature, which is lacking any such curricula. Our incorporation of a flipped classroom design with self-study educational modules, followed by in-person simulations and material review, was a particularly unique aspect amongst the educational material available on pediatric sepsis. Ideally, MS 4s transitioning to residency, particularly pediatric residency, can utilize this curricular design to advance their skill set and confidence in caring for pediatric patients presenting with sepsis. The curriculum would be best implemented as a supplement to medical student rotations in neonatal or pediatric critical care; however, it may be useful in pediatric emergency care rotations or residency education.

The results of our pre-/posttest analysis suggested that the curriculum advanced students’ knowledge and improved self-reported confidence in managing theoretical pediatric patients with sepsis. During the simulation sessions we witnessed improvement in performance of correct steps based on the Surviving Sepsis guidelines, as well as improved technical skills in each subsequent simulation case, demonstrating the application of deliberate practice. Almost all learners expressed praise for the curriculum, endorsing a perceived increase in their knowledge of pediatric sepsis from the course modules, as well as improved hands-on skills from the simulations. We received the most positive feedback on the simulation sessions, with students expressing appreciation for the opportunity to practice case-based scenarios.

The strengths of our curriculum development included the relatively long data collection period, study completion rate, quantitative data obtained, and generalizability. This project spanned multiple academic years and demonstrated similar and consistent results over time. Of the students who enrolled in the curriculum, 75% completed the course. We were able to obtain quantitative data representing learner knowledge and confidence before and after participation in the curriculum. Lastly, we designed the curriculum to be generalizable to all pediatric learners. In fact, we plan to expand this curriculum in the near future by extending participation to include pediatric resident physicians.

Our project has several limitations, including a relatively small sample size, a single institution experience and possible selection and test/retest bias of participants. A control group of medical students enrolled in NICU or PICU rotations who take the pre-/posttests, but do not participate in the curriculum, would be ideal. Given the small number of students who enroll in NICU and PICU rotations, as well as our desire to offer every student the opportunity to participate, this was not feasible at our institution. Given that this was a single institution experience, additional research is needed for broader application and standardization. Furthermore, pre-/posttest results may vary based on time of year and students’ prior exposures, given that students had variable experiences prior to participation in the study. However, all students had completed their core rotations, including pediatrics, during their third year of medical school training, suggesting a more even playing field during MS 4.

One challenge we faced was finding time in medical students’ schedules to fit in this educational opportunity. We were ultimately able to incorporate the curriculum into medical student education by including it in the established NICU and PICU rotations, and by working with department leadership to ensure protected learning times. Thus, for others to implement this curriculum, it is imperative to find or make space in students’ or residents’ schedules. Another challenge that other institutions may face is the accessibility of a simulation lab and simulation staff. For sites that cannot access simulation technology, the simulation cases can still be utilized via role-play in person, or remotely via video communication interfaces.

Moving forward, we plan to continue the curriculum utilizing our updated material based on the 2020 Surviving Sepsis pediatric guidelines,^12^ and analyze the impact of the course using our updated pre-/posttest (Appendix A). We would also like to expand the course to include pediatric residents by incorporating it into a relevant and time-allowing clinical rotation. While we have started with sepsis, in the future this curricular model can be utilized to cover other pediatric and neonatal critical care topics.

## Appendices

Pre- & Posttest.docxModule 1 - Pediatric Shock.pptxScript 1 - Pediatric Shock.docxModule 2 - Pediatric Sepsis.pptxScript 2 - Pediatric Sepsis.docxModule 3 - Management of Sepsis & Septic Shock.pptxScript 3 - Management of Sepsis & Septic Shock. docxModule 4 - Hemodynamics & Pressor Support.pptxScript 4 - Hemodynamics & Pressor Support.docxSimulation Case 1.docxSimulation Case 2.docxSimulation Case 3.docxPostsimulation Review Quiz.pptx
All appendices are peer reviewed as integral parts of the Original Publication.
