# Supplementary material for: Identification and Management of Pediatric Sepsis: A Medical Student Curricular Supplement for PICU and NICU Rotations
Source: MedEdPORTAL. 2021 Apr 23;17:11142. doi: 10.15766/mep_2374-8265.11142 (PMC8063627; doi:10.15766/mep_2374-8265.11142)
Supplement: Supplementary file 1 — Pre- & Posttest.docxModule 1 - Pediatric Shock.pptxScript 1 - Pediatric Shock.docxModule 2 - Pediatric Sepsis.pptxScript 2 - Pediatric Sepsis.docxModule 3 - Management of Sepsis & Septic Shock.pptxScript 3 - Management of Sepsis & Septic Shock. docxModule 4 - Hemodynamics & Pressor Support.pptxScript 4 - Hemodynamics & Pressor Support.docxSimulation Case 1.docxSimulation Case 2.docxSimulation Case 3.docxPostsimulation Review Quiz.pptx [file mep_2374-8265.11142-s001.zip › A. Pre- & Posttest.docx]

1) Which of the following is the most appropriate initial resuscitation for a patient in septic shock?

A) Albumin 5% 10 cc/kg bolus run over 5 minutes

B) Albumin 25% 10cc/kg bolus run over 15 minutes

C) Normal saline 10cc/kg bolus run over 15 minutes

**D) Lactated ringer 20cc/kg bolus run over 5 minutes**

2) Which of the following medications should be started for fluid refractory septic shock?

A) Dopamine

**B) Norepinephrine**

C) Vasopressin

D) Hydrocortisone

3) According to the Surviving Sepsis Guidelines, within what time frame should empiric antibiotics be administered after recognition of septic shock?

A) Within 10-15 minutes

B) Within 30 minutes

**C) Within 1 hour**

D) No defined time. Antibiotics should be delayed until after blood cultures are obtained.

4) A previously healthy newborn develops septic shock after a day of lethargy and fever. Which initial medications do you administer?

A) Ampicillin

B) Gentamicin

C) Caspofungin

**D) A and B**

E) A, B and C

5) Which of the following children meets SIRS criteria?

A) An 8-year-old girl with a temperature of 37.5 °C, tachycardia, normal respiratory rate and normal leukocyte count.

B) A 1-month-old boy with a temperature of 35.8 °C, normal heart rate, normal respiratory rate and elevated leukocyte count.

C) A 12-year-old boy with a temperature of 37 °C, normal heart rate, tachypnea and elevated leukocyte count.

D) A 2-year-old girl with a temperature of 38.2 °C, tachycardia, tachypnea and elevated leukocyte count.

E) B and D

6) How comfortable are you in making a clinical diagnosis of shock?

A) Very uncomfortable

B) Somewhat uncomfortable

C) Neutral

D) Somewhat comfortable

E) Very comfortable

7) What is your level of comfort in managing pediatric patients?

A) Very uncomfortable

B) Somewhat uncomfortable

C) Neutral

D) Somewhat comfortable

E) Very comfortable

8) How comfortable do you feel about running a pediatric code?

A) Very uncomfortable

B) Somewhat uncomfortable

C) Neutral

D) Somewhat comfortable

E) Very comfortable

9) What are the last 3 digits of your phone number?

10) How can we improve the program? What should we do differently for the next group? We greatly value your feedback!

Survey created by:

Kristi Kambestad, MD

Valencia Walker, MD

Cristina Gutierrez, MD

Nicole Anderson, MD

Mai-King Chan, MD
