# Supplementary material for: Identification and Management of Pediatric Sepsis: A Medical Student Curricular Supplement for PICU and NICU Rotations
Source: MedEdPORTAL. 2021 Apr 23;17:11142. doi: 10.15766/mep_2374-8265.11142 (PMC8063627; doi:10.15766/mep_2374-8265.11142)
Supplement: Supplementary file 1 — Pre- & Posttest.docxModule 1 - Pediatric Shock.pptxScript 1 - Pediatric Shock.docxModule 2 - Pediatric Sepsis.pptxScript 2 - Pediatric Sepsis.docxModule 3 - Management of Sepsis & Septic Shock.pptxScript 3 - Management of Sepsis & Septic Shock. docxModule 4 - Hemodynamics & Pressor Support.pptxScript 4 - Hemodynamics & Pressor Support.docxSimulation Case 1.docxSimulation Case 2.docxSimulation Case 3.docxPostsimulation Review Quiz.pptx [file mep_2374-8265.11142-s001.zip › B. Module 1 - Pediatric Shock.pptx]

## Slide 1
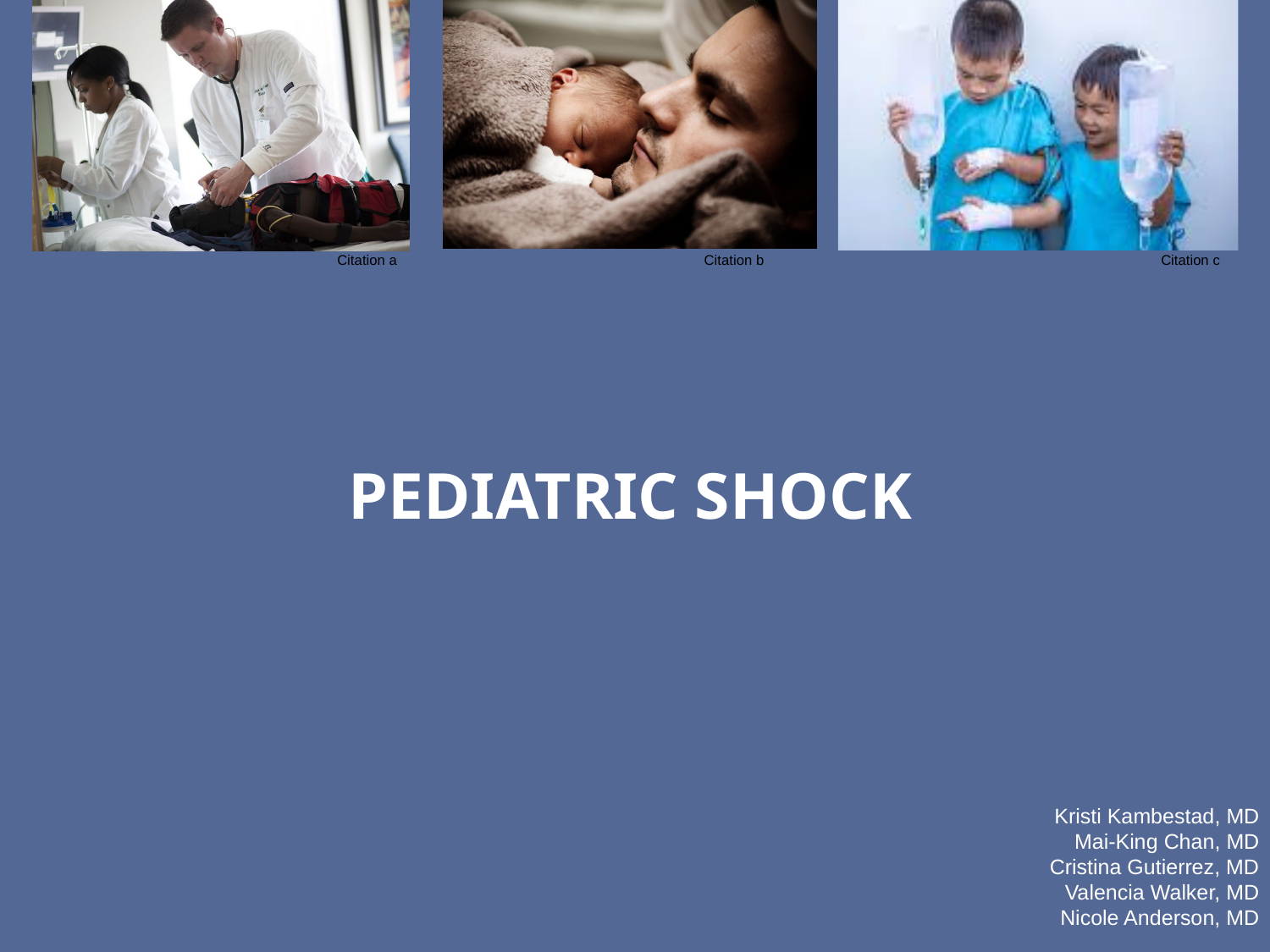

Citation a
Citation b
Citation c
PEDIATRIC SHOCK
Kristi Kambestad, MD
Mai-King Chan, MD
Cristina Gutierrez, MD
Valencia Walker, MD
Nicole Anderson, MD

## Slide 2
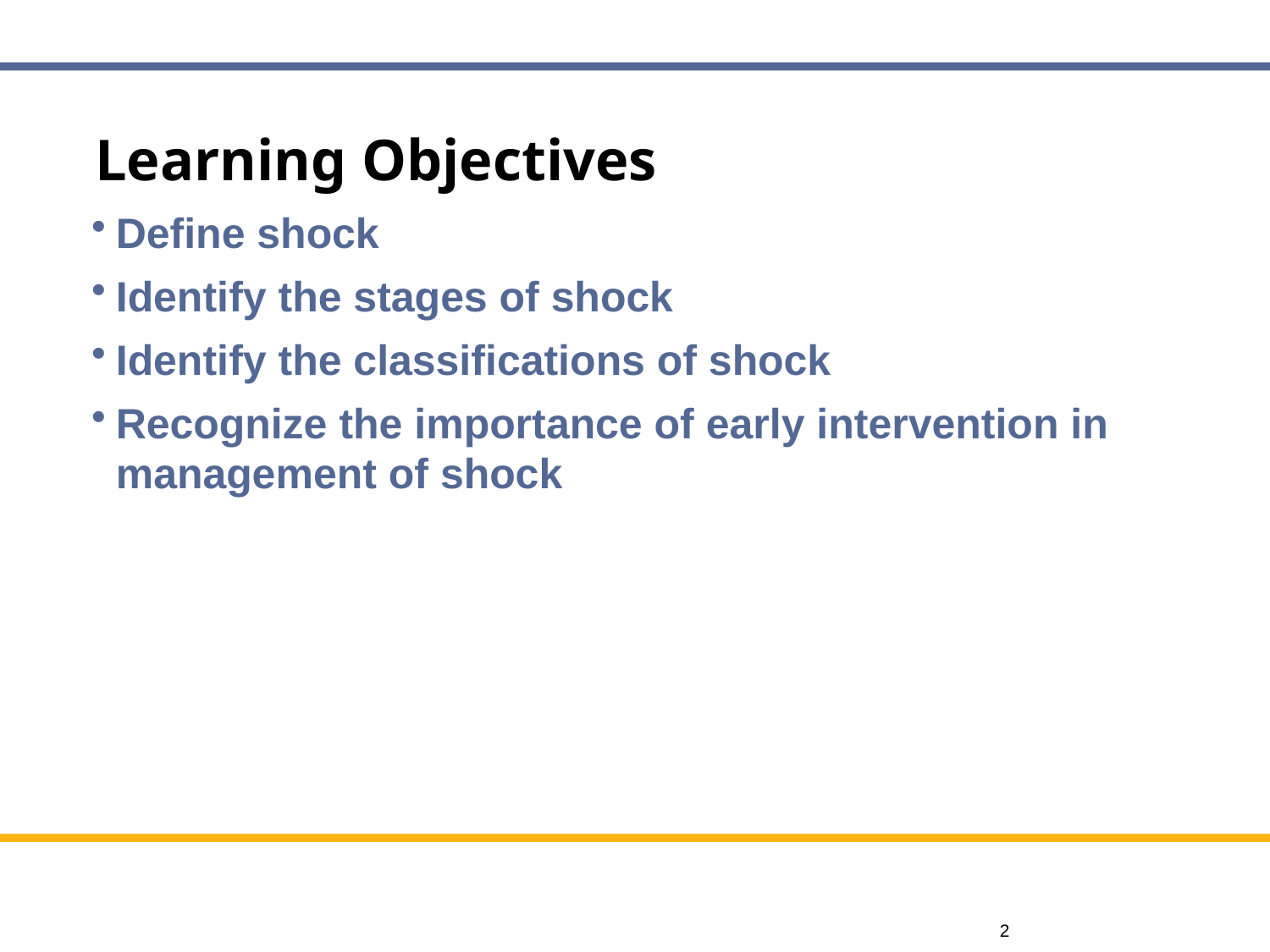

Learning Objectives
Define shock
Identify the stages of shock
Identify the classifications of shock
Recognize the importance of early intervention in management of shock
2

## Slide 3
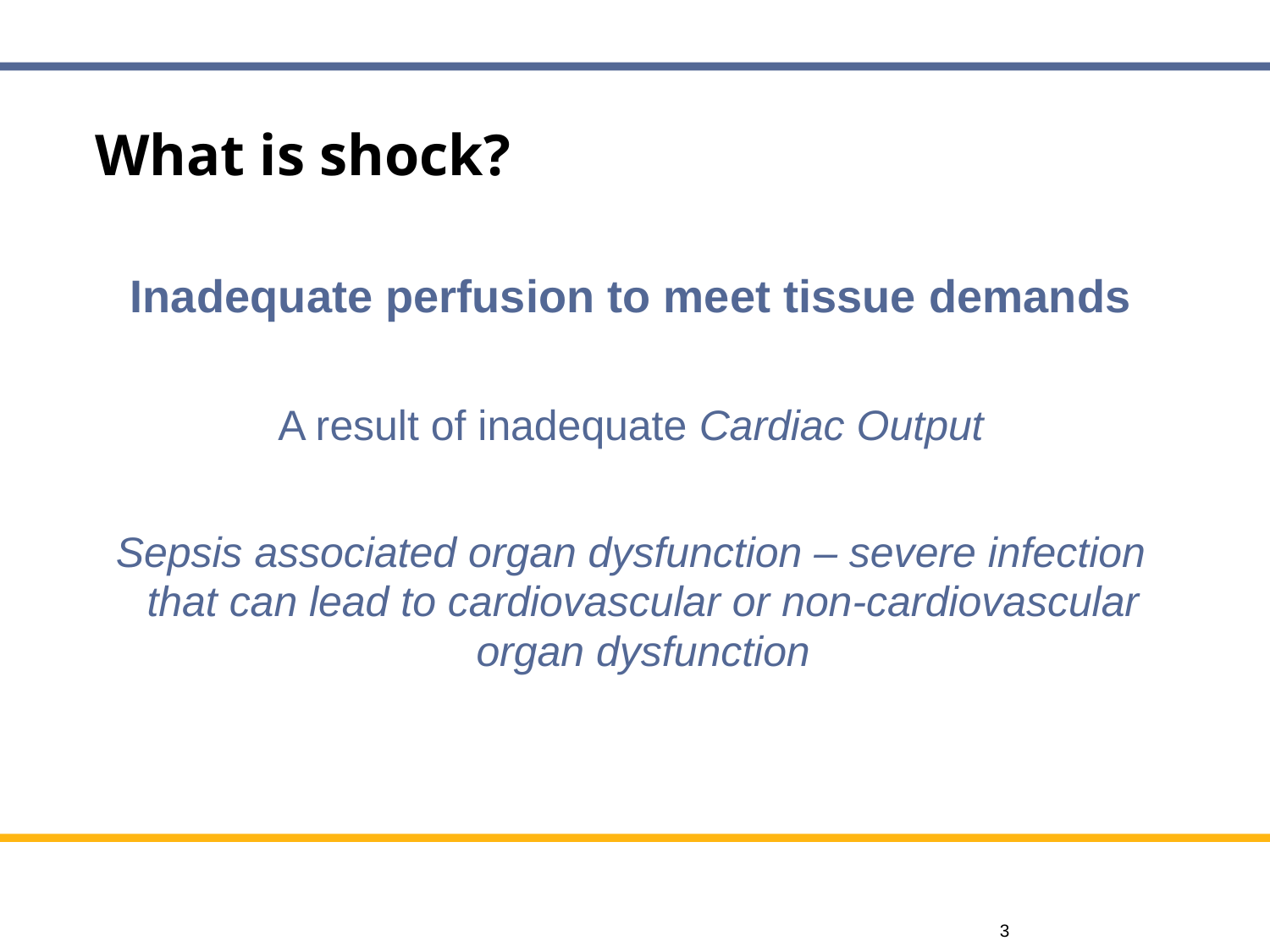

What is shock?
Inadequate perfusion to meet tissue demands
A result of inadequate Cardiac Output
Sepsis associated organ dysfunction – severe infection that can lead to cardiovascular or non-cardiovascular organ dysfunction
3

## Slide 4
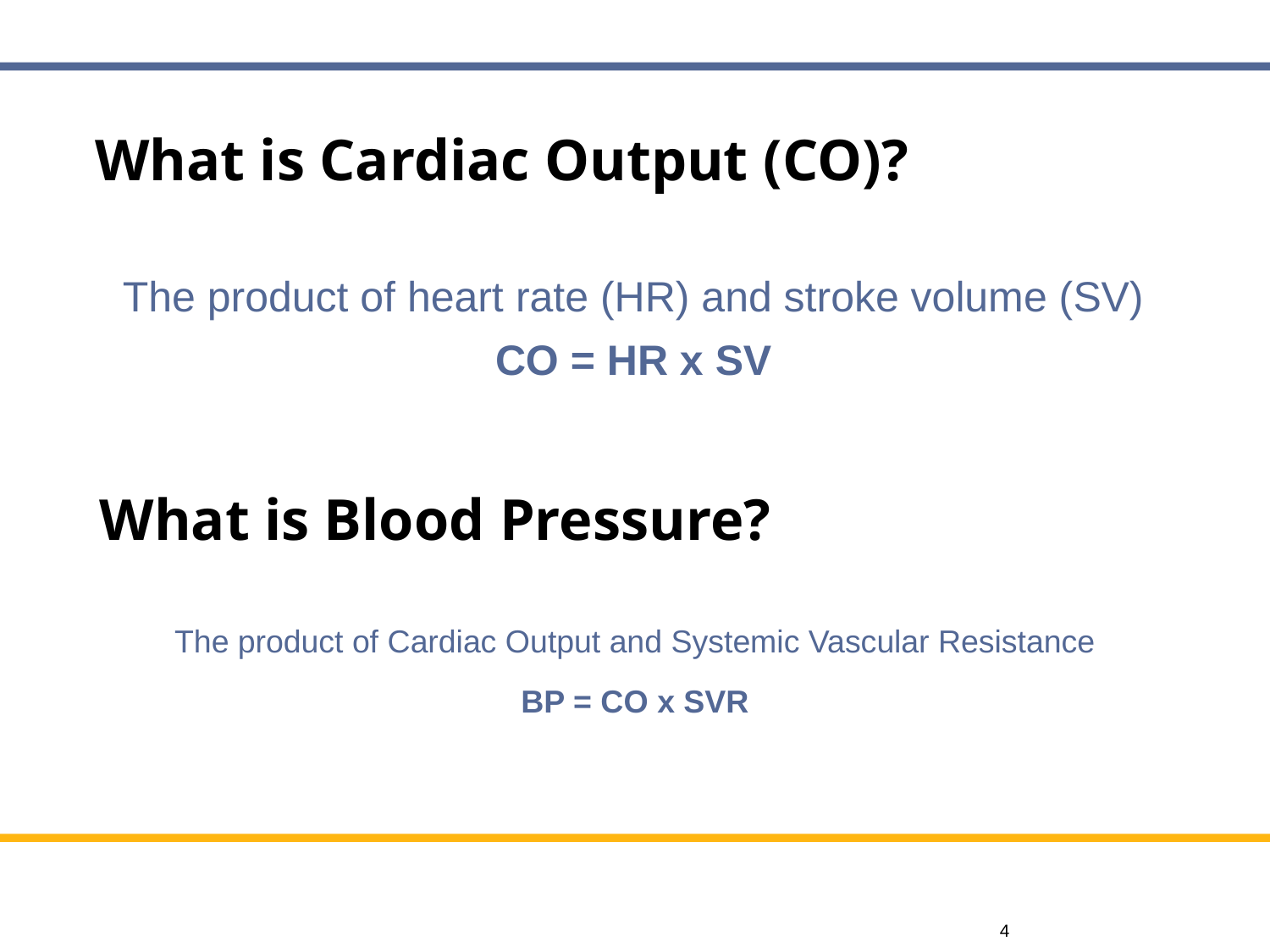

What is Cardiac Output (CO)?
The product of heart rate (HR) and stroke volume (SV)
CO = HR x SV
What is Blood Pressure?
The product of Cardiac Output and Systemic Vascular Resistance
BP = CO x SVR
4

## Slide 5
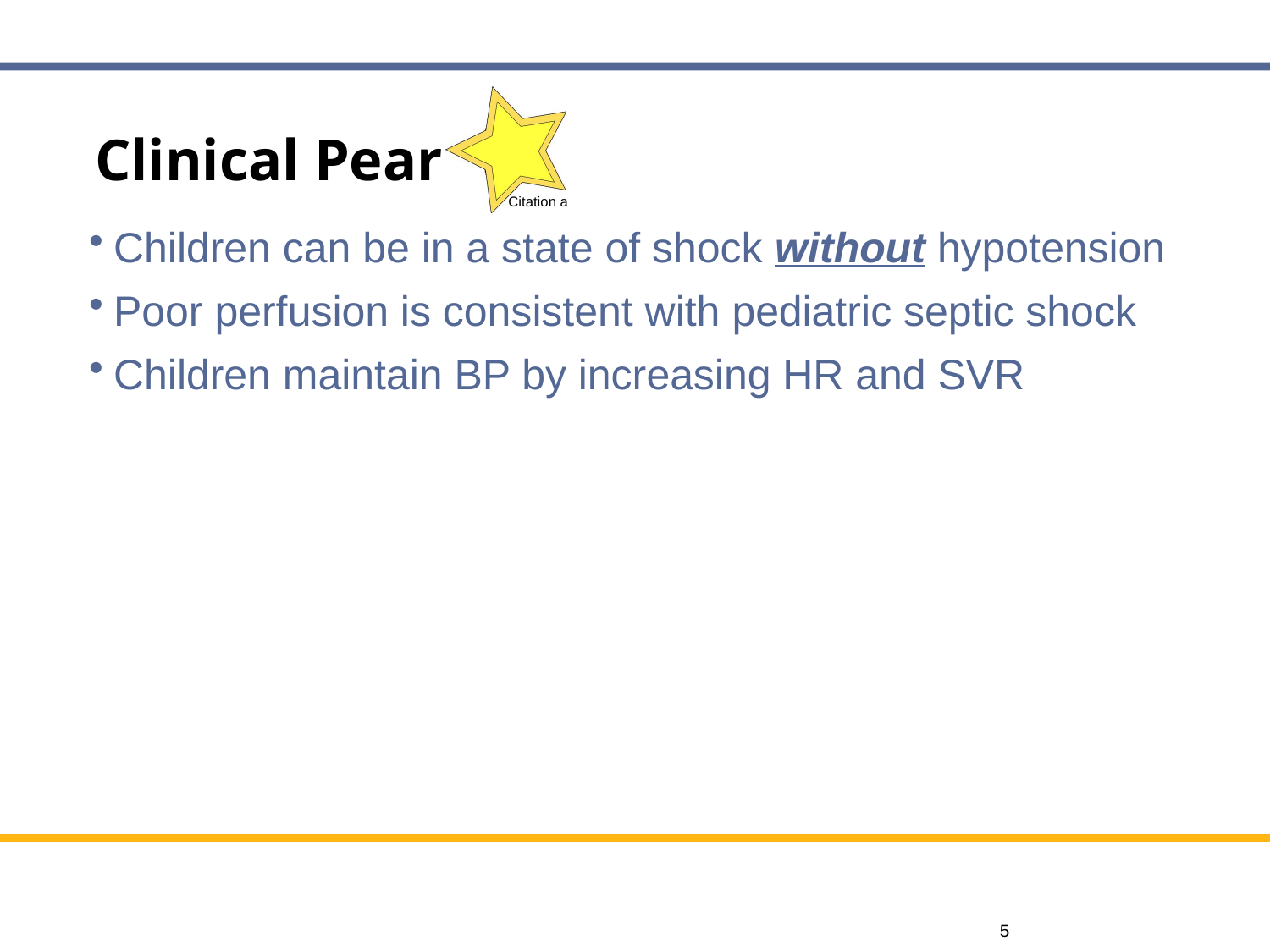

Clinical Pearl
Citation a
Children can be in a state of shock without hypotension
Poor perfusion is consistent with pediatric septic shock
Children maintain BP by increasing HR and SVR
5

## Slide 6
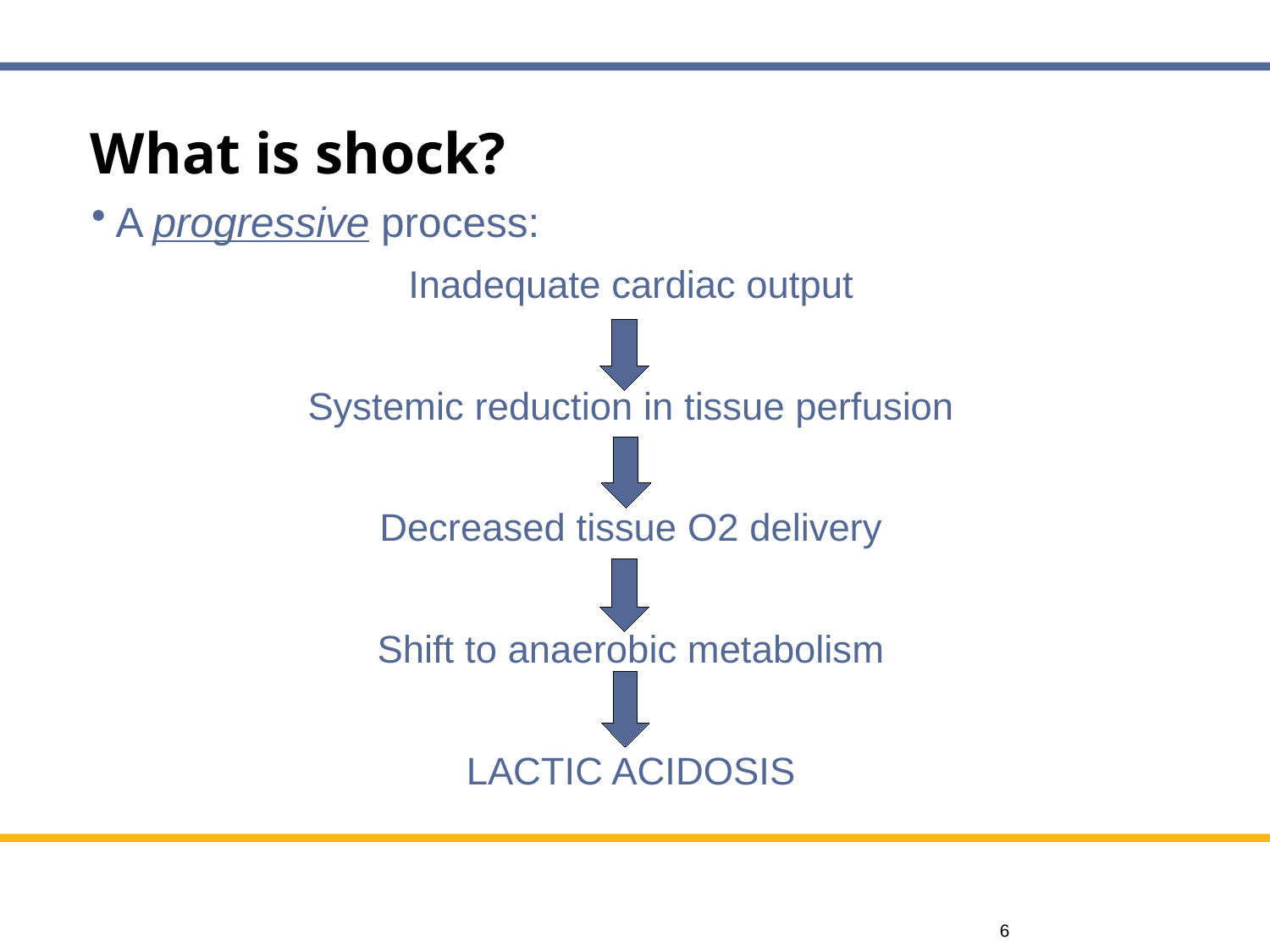

What is shock?
A progressive process:
Inadequate cardiac output
Systemic reduction in tissue perfusion
Decreased tissue O2 delivery
Shift to anaerobic metabolism
LACTIC ACIDOSIS
6

## Slide 7
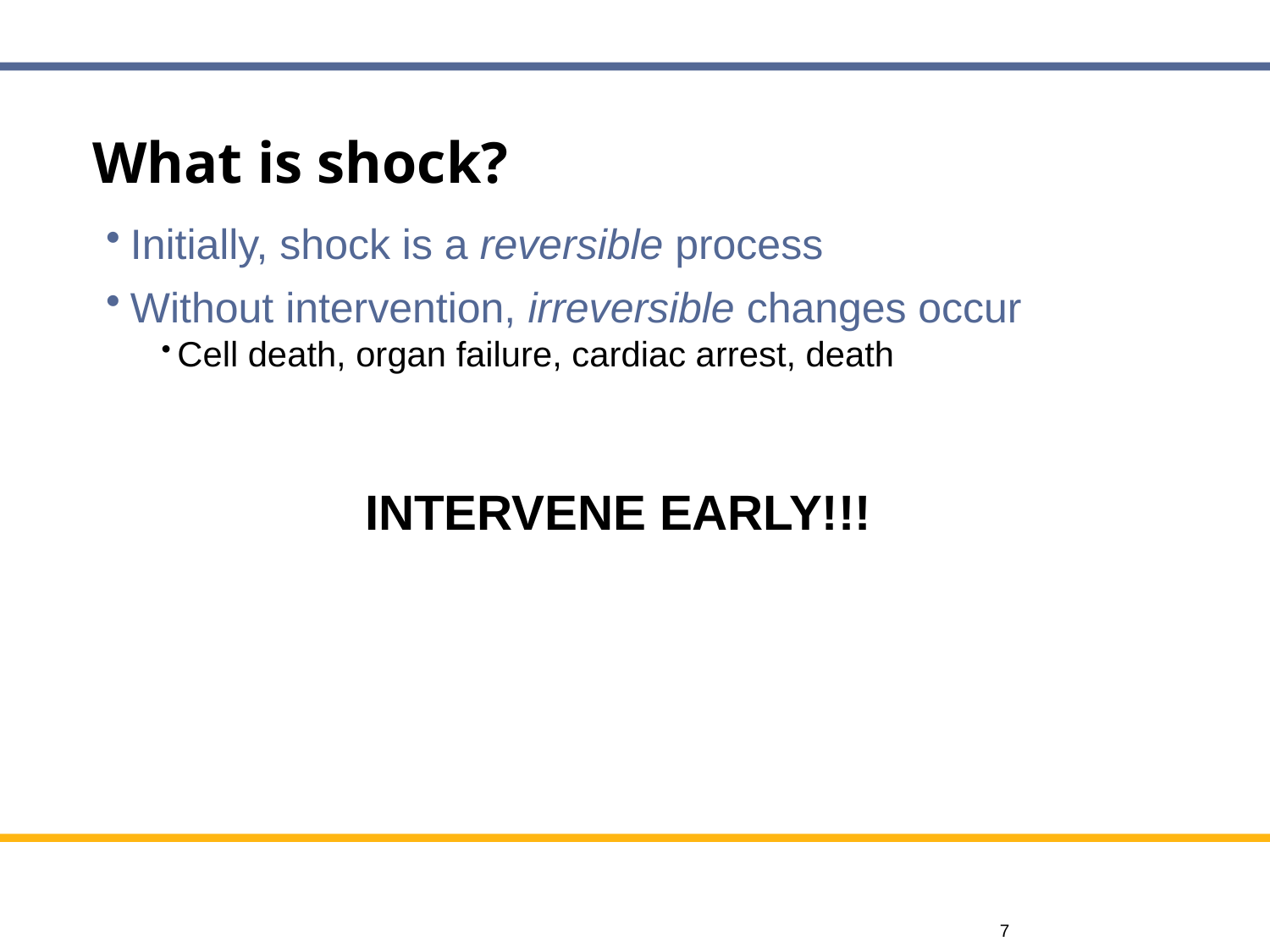

What is shock?
Initially, shock is a reversible process
Without intervention, irreversible changes occur
Cell death, organ failure, cardiac arrest, death
INTERVENE EARLY!!!
7

## Slide 8
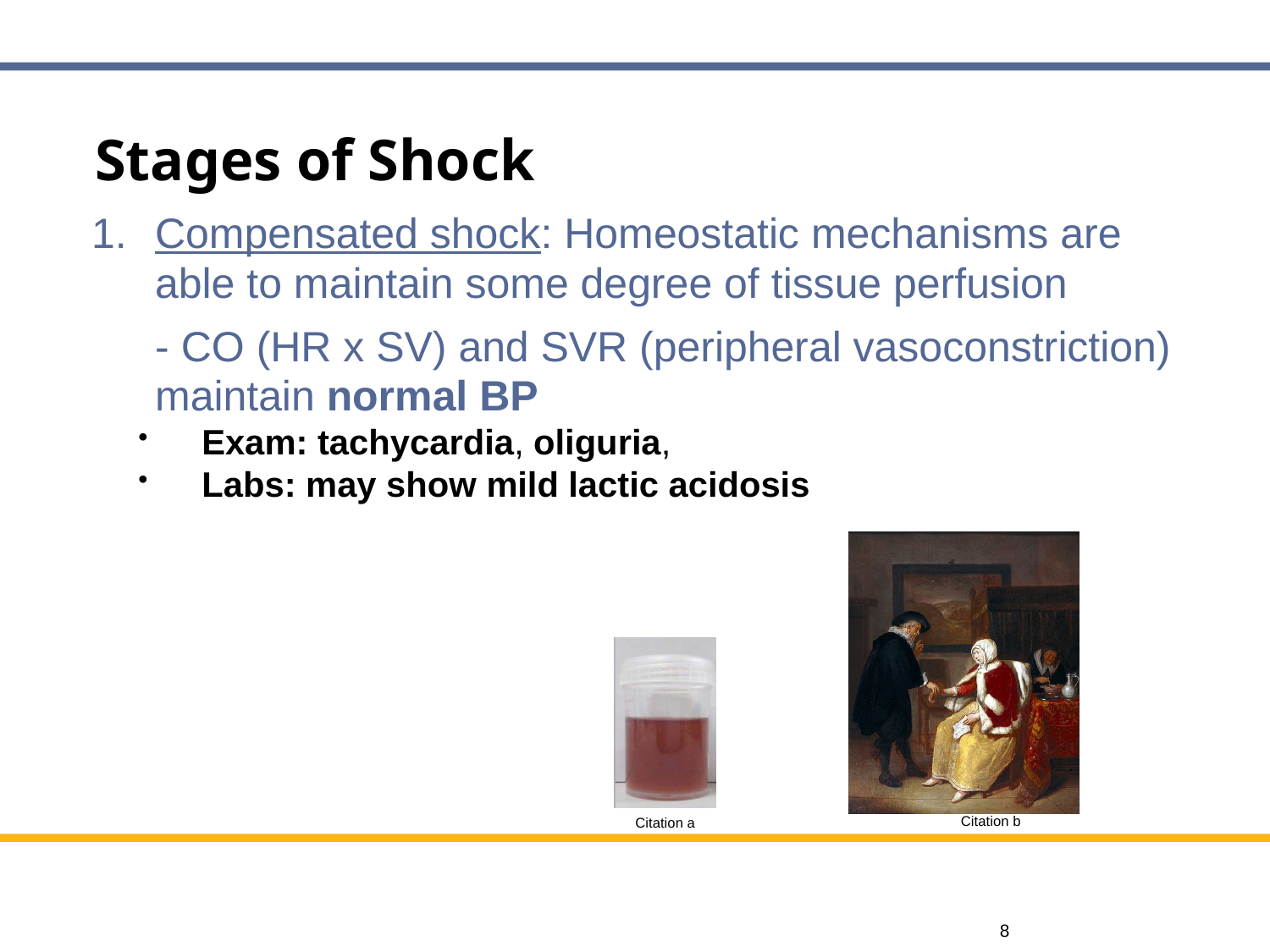

Stages of Shock
Compensated shock: Homeostatic mechanisms are able to maintain some degree of tissue perfusion
	- CO (HR x SV) and SVR (peripheral vasoconstriction) maintain normal BP
Exam: tachycardia, oliguria,
Labs: may show mild lactic acidosis
Citation b
Citation a
8

## Slide 9
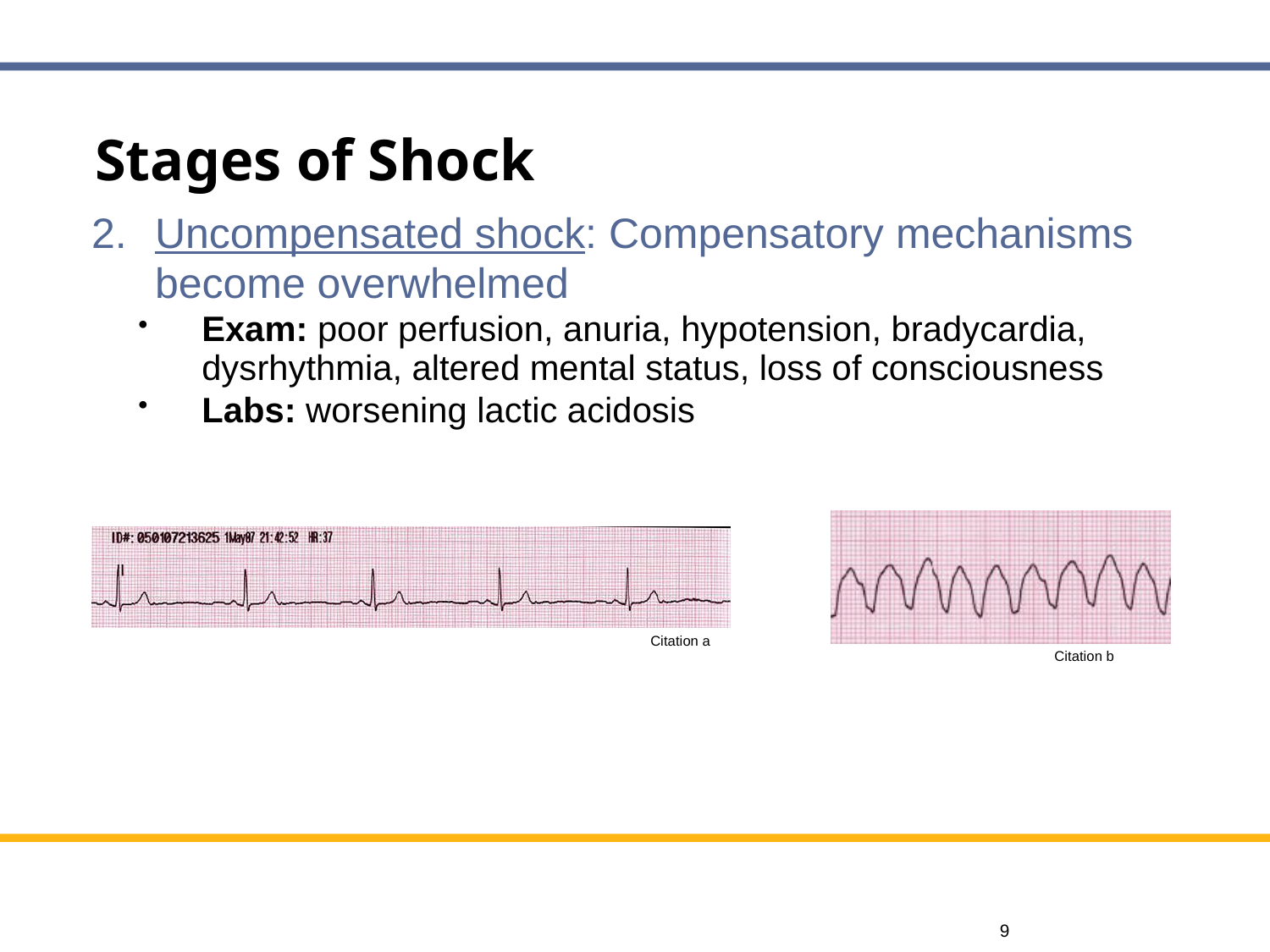

Stages of Shock
Uncompensated shock: Compensatory mechanisms become overwhelmed
Exam: poor perfusion, anuria, hypotension, bradycardia, dysrhythmia, altered mental status, loss of consciousness
Labs: worsening lactic acidosis
Citation a
Citation b
9

## Slide 10
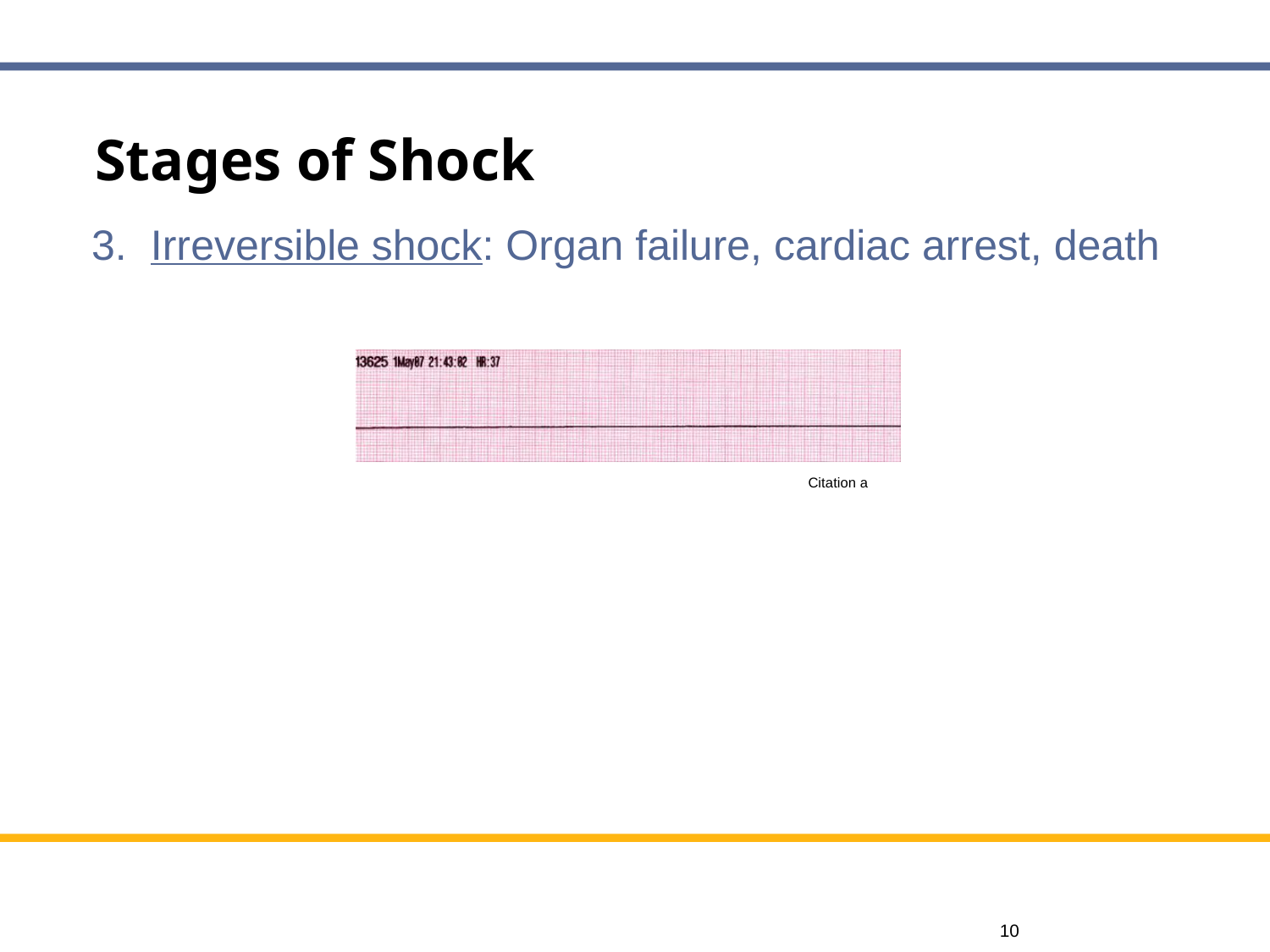

Stages of Shock
3. Irreversible shock: Organ failure, cardiac arrest, death
Citation a
10

## Slide 11
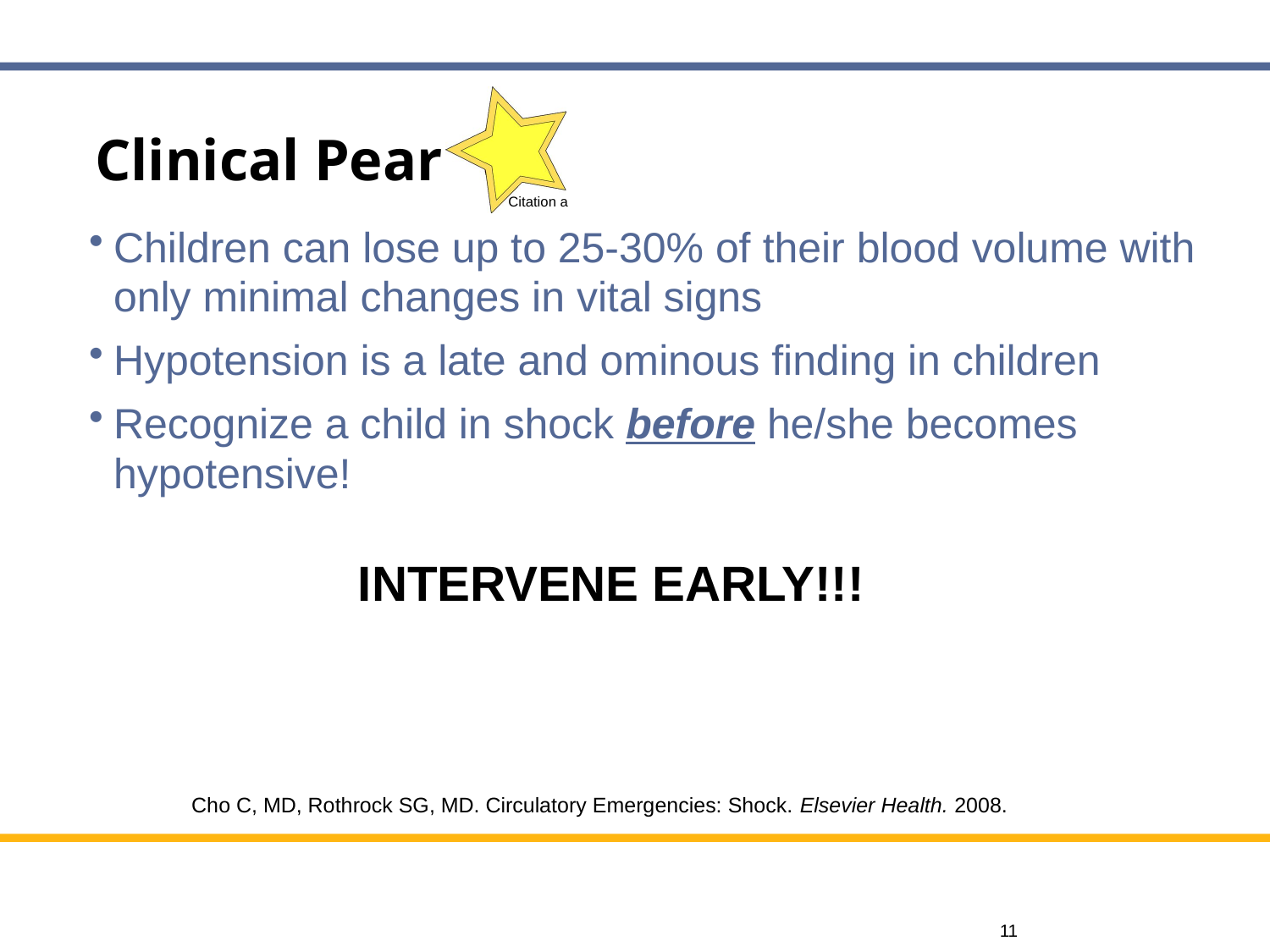

Clinical Pearl
Citation a
Children can lose up to 25-30% of their blood volume with only minimal changes in vital signs
Hypotension is a late and ominous finding in children
Recognize a child in shock before he/she becomes hypotensive!
INTERVENE EARLY!!!
Cho C, MD, Rothrock SG, MD. Circulatory Emergencies: Shock. Elsevier Health. 2008.
11

## Slide 12
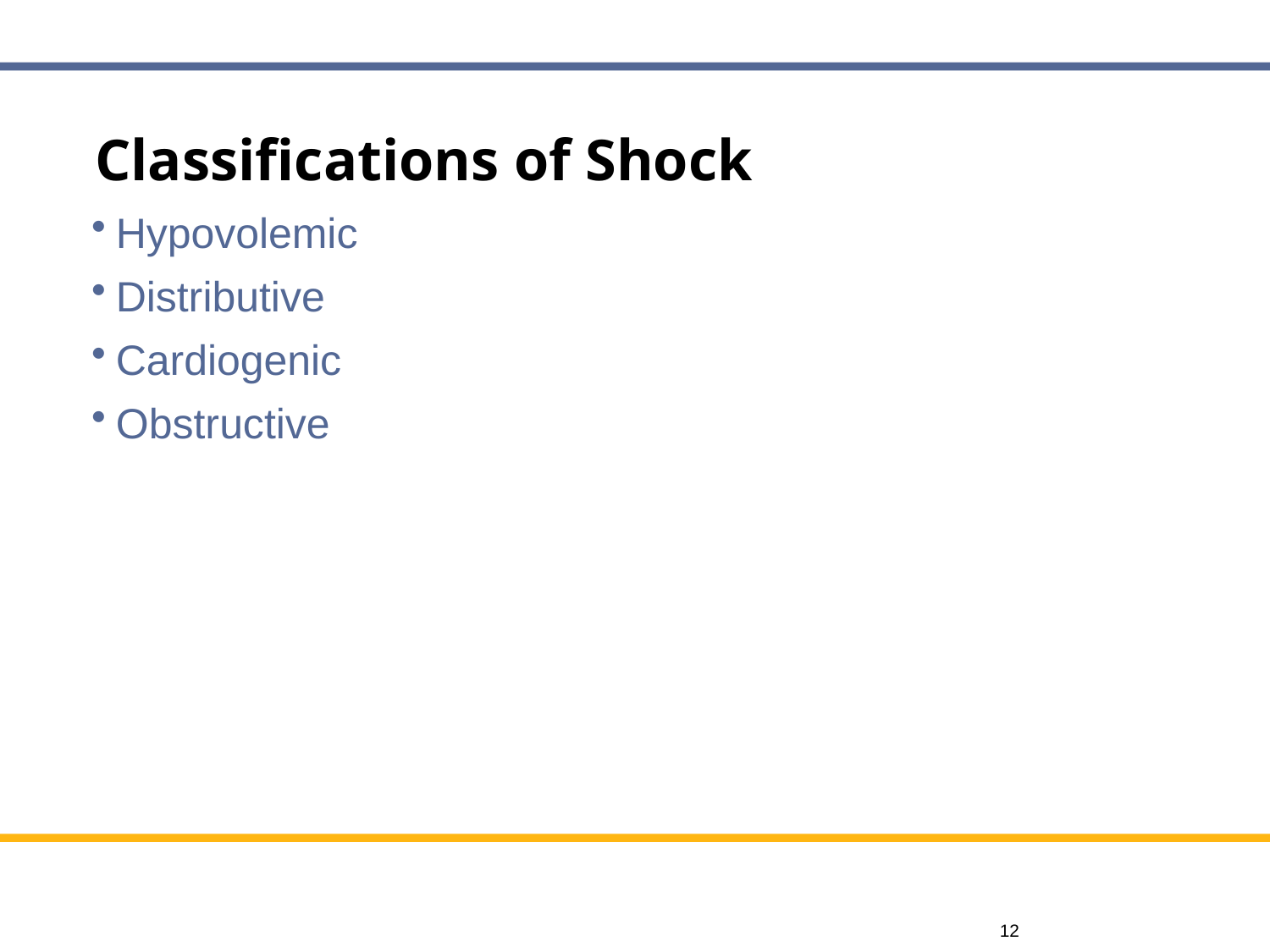

Classifications of Shock
Hypovolemic
Distributive
Cardiogenic
Obstructive
12

## Slide 13
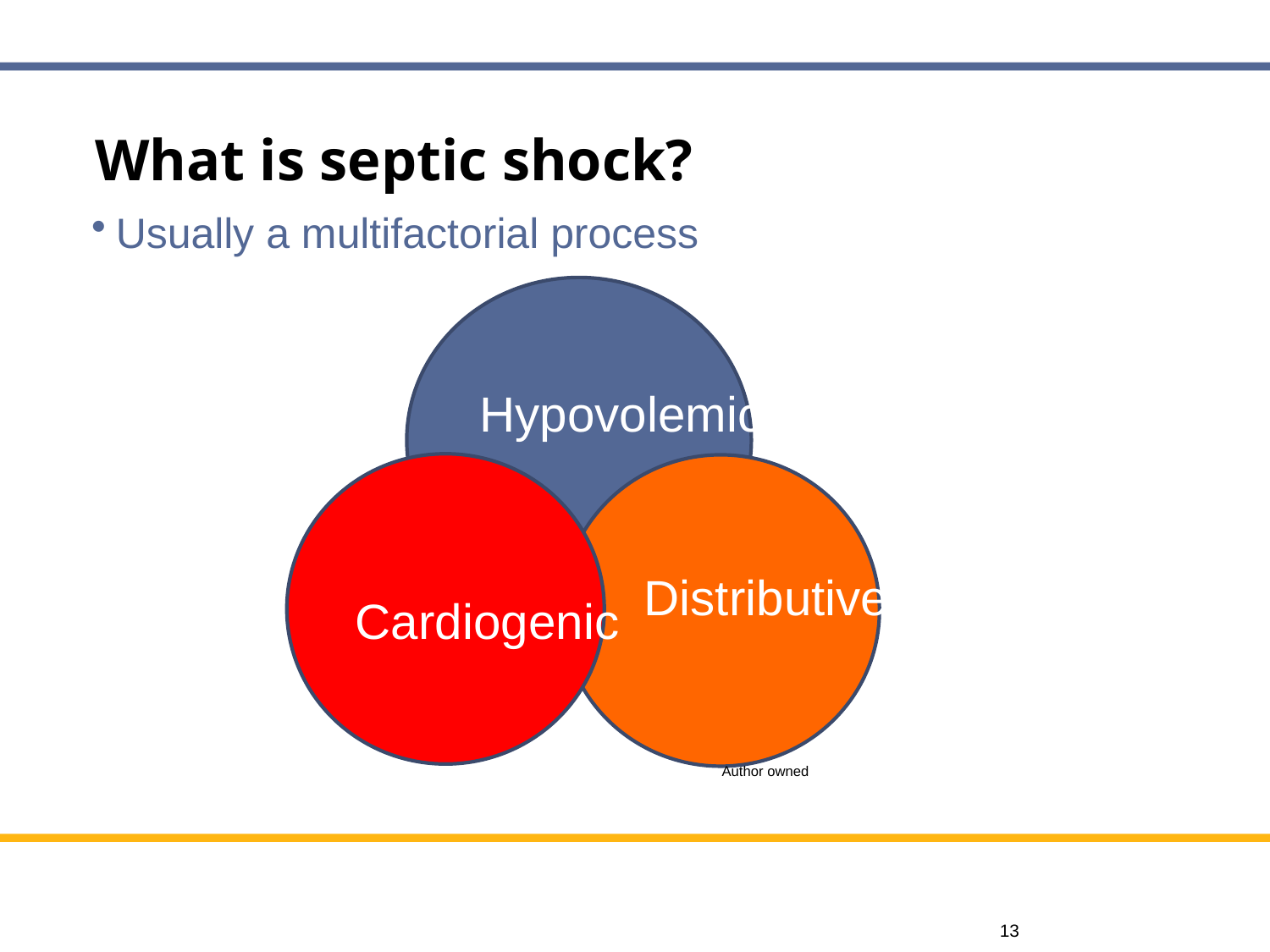

What is septic shock?
Usually a multifactorial process
Hypovolemic
Distributive
Cardiogenic
Author owned
13

## Slide 14
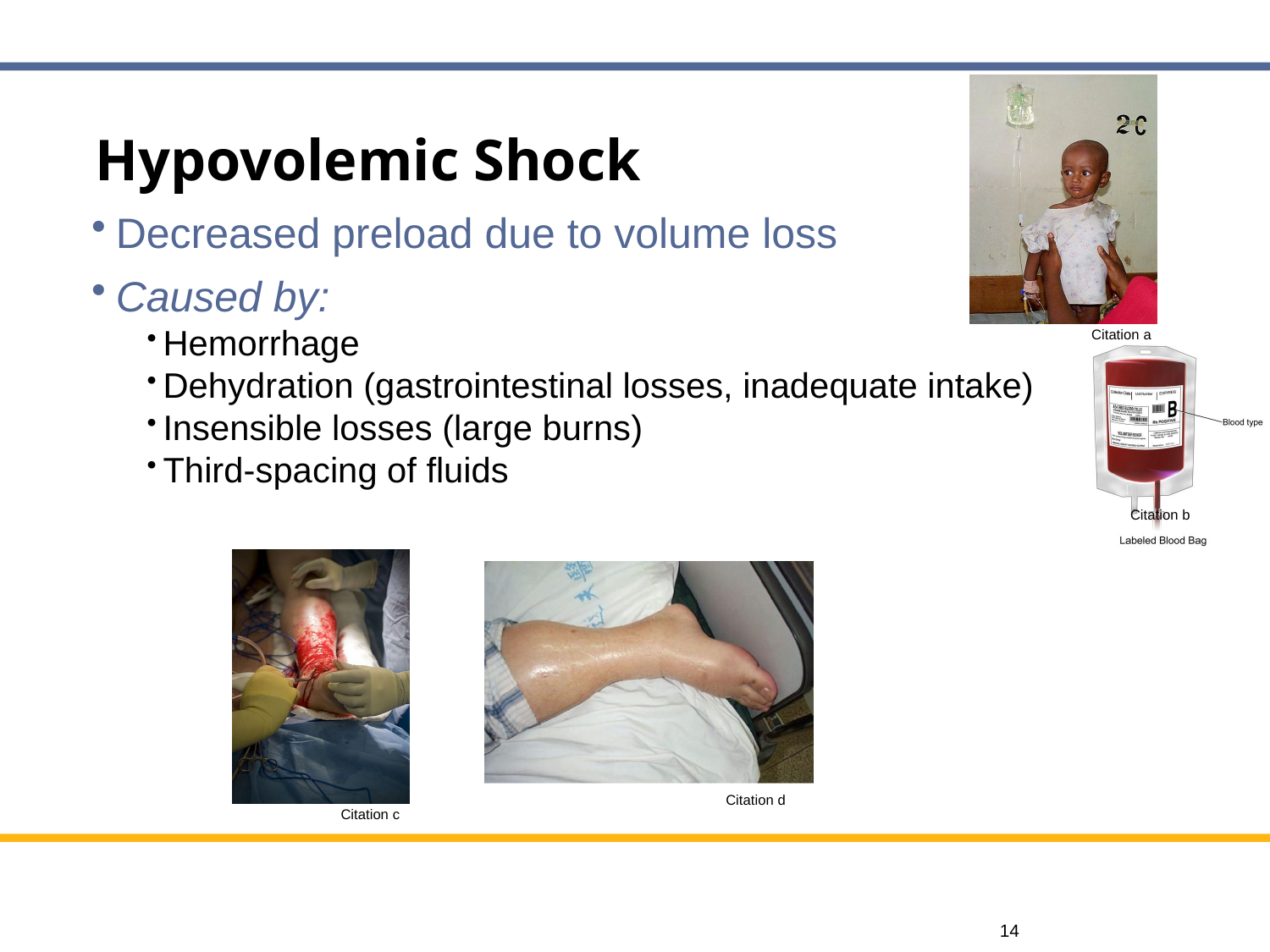

Hypovolemic Shock
Decreased preload due to volume loss
Caused by:
Hemorrhage
Dehydration (gastrointestinal losses, inadequate intake)
Insensible losses (large burns)
Third-spacing of fluids
Citation a
Citation b
Citation d
Citation c
14

## Slide 15
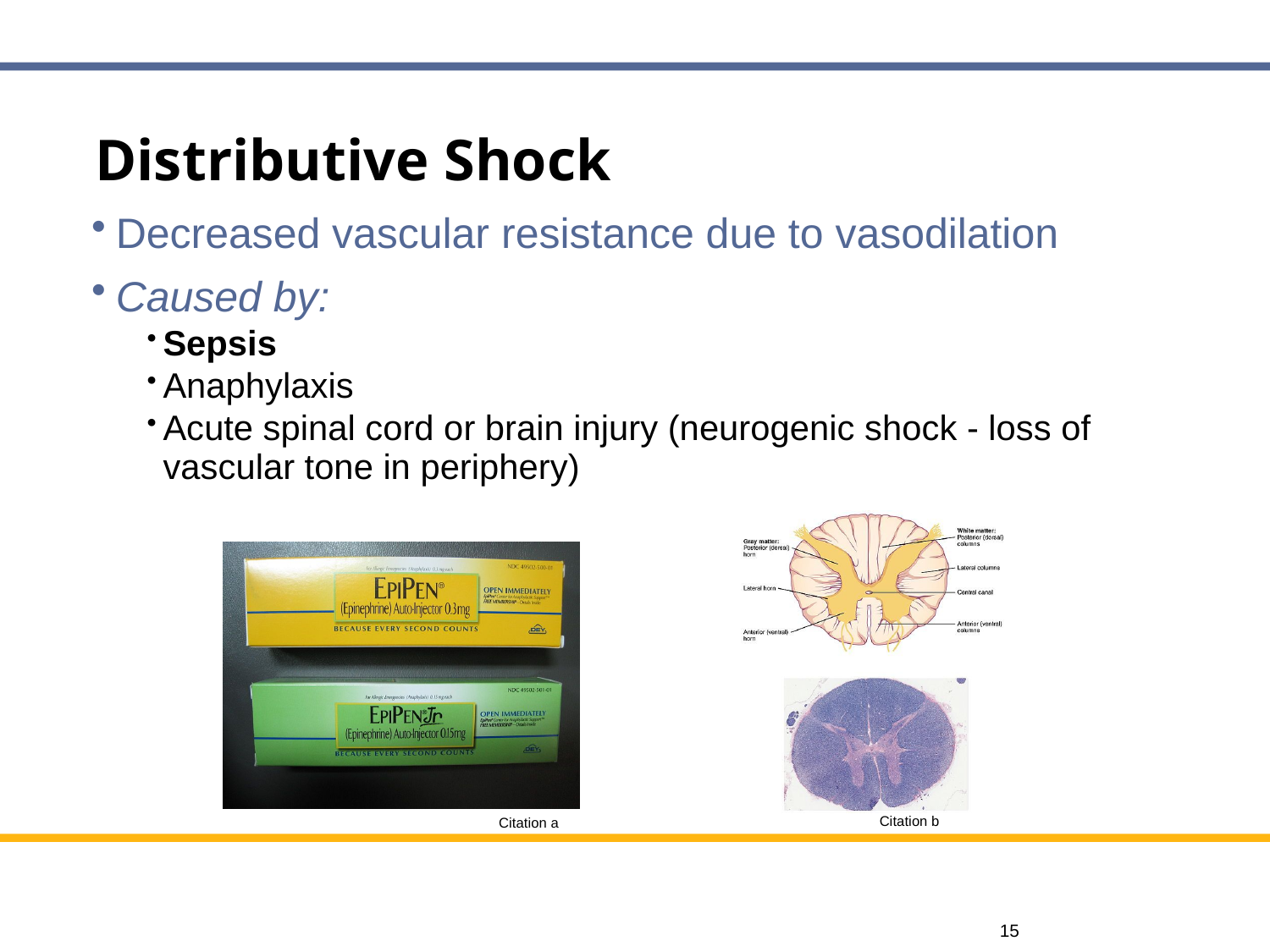

Distributive Shock
Decreased vascular resistance due to vasodilation
Caused by:
Sepsis
Anaphylaxis
Acute spinal cord or brain injury (neurogenic shock - loss of vascular tone in periphery)
Citation b
Citation a
15

## Slide 16
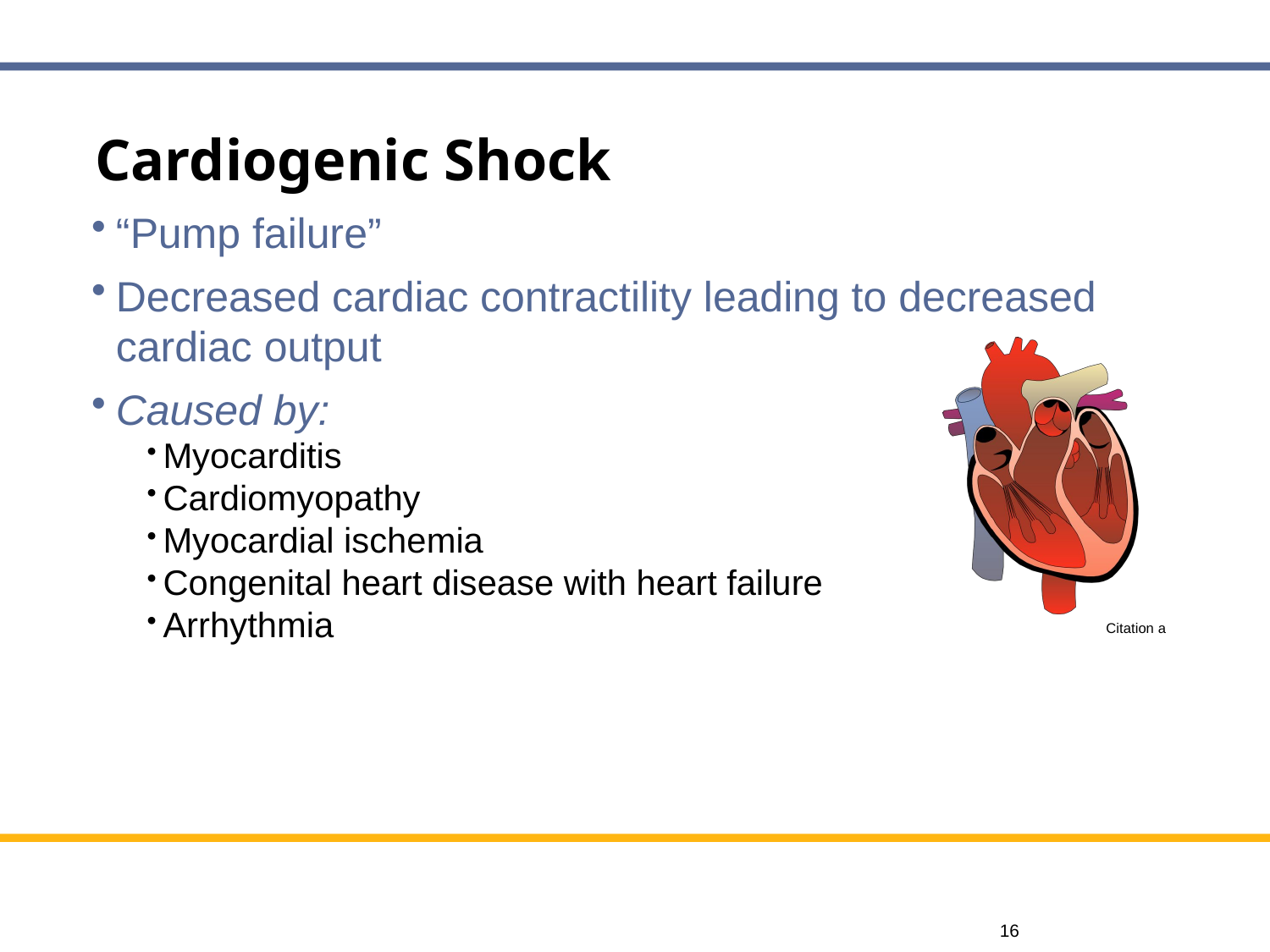

Cardiogenic Shock
“Pump failure”
Decreased cardiac contractility leading to decreased cardiac output
Caused by:
Myocarditis
Cardiomyopathy
Myocardial ischemia
Congenital heart disease with heart failure
Arrhythmia
Citation a
16

## Slide 17
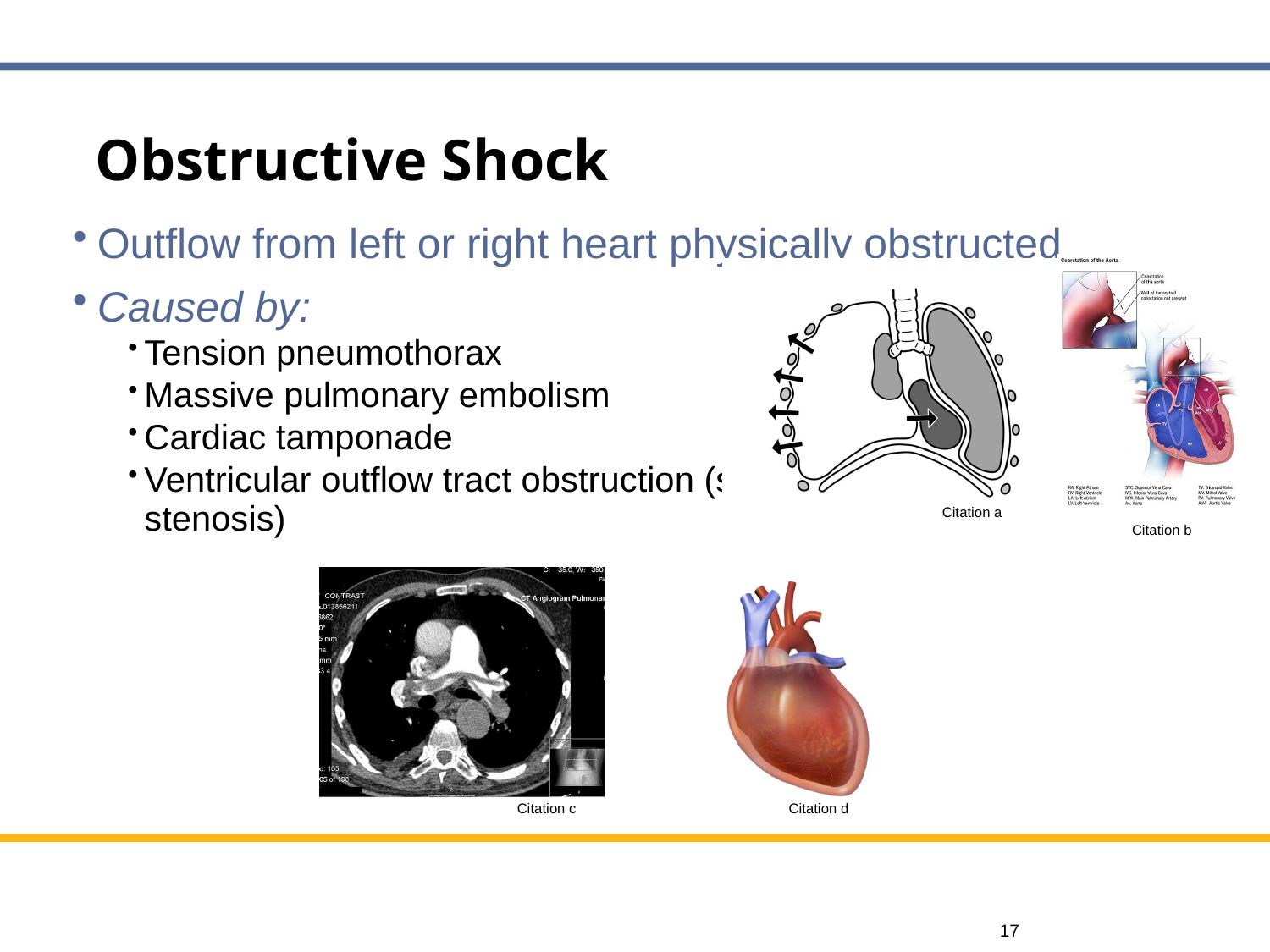

Obstructive Shock
Outflow from left or right heart physically obstructed
Caused by:
Tension pneumothorax
Massive pulmonary embolism
Cardiac tamponade
Ventricular outflow tract obstruction (severe coarctation, aortic stenosis)
Citation a
Citation b
Citation c
Citation d
17

## Slide 18
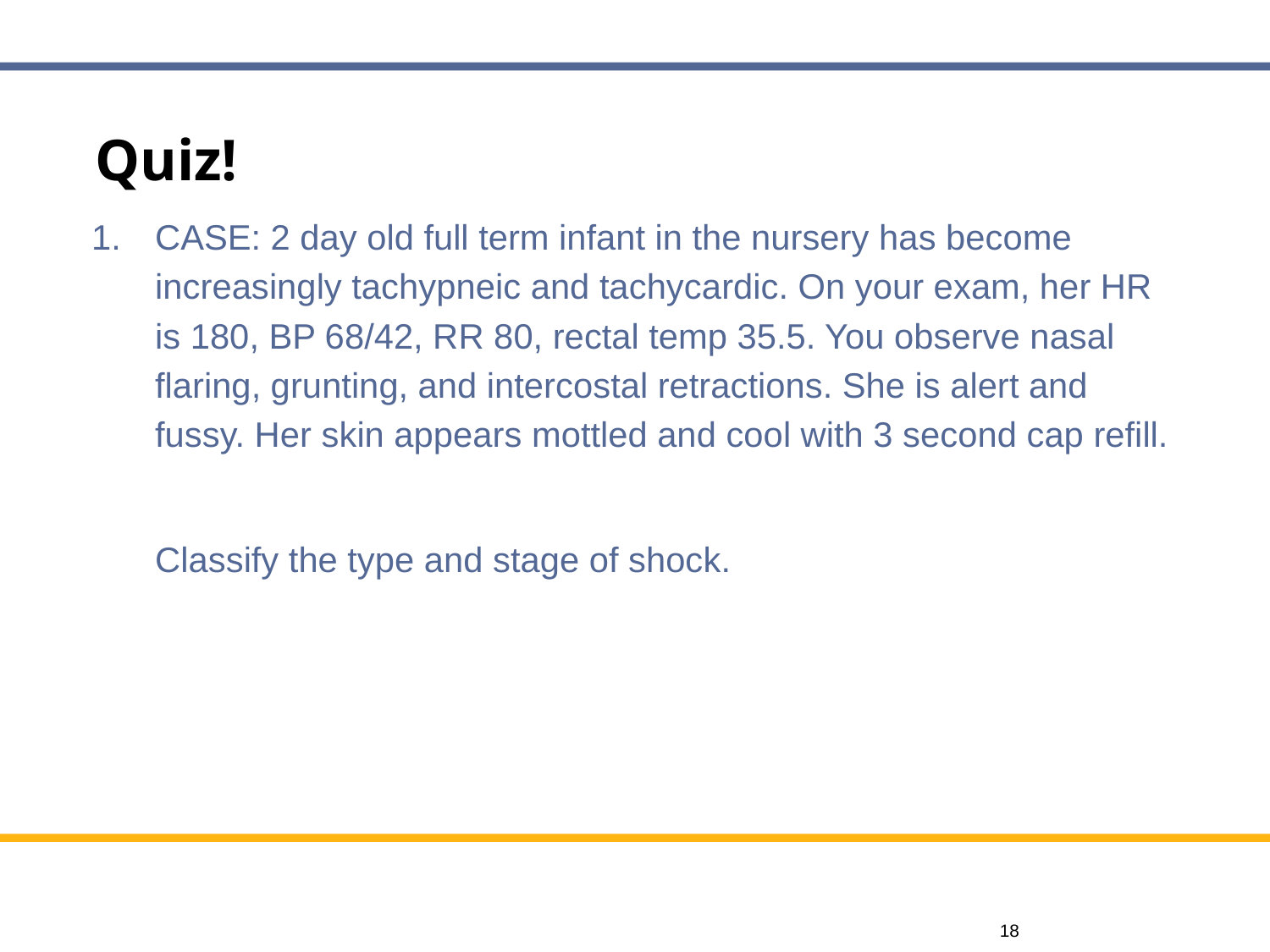

Quiz!
CASE: 2 day old full term infant in the nursery has become increasingly tachypneic and tachycardic. On your exam, her HR is 180, BP 68/42, RR 80, rectal temp 35.5. You observe nasal flaring, grunting, and intercostal retractions. She is alert and fussy. Her skin appears mottled and cool with 3 second cap refill.
	Classify the type and stage of shock.
18

## Slide 19
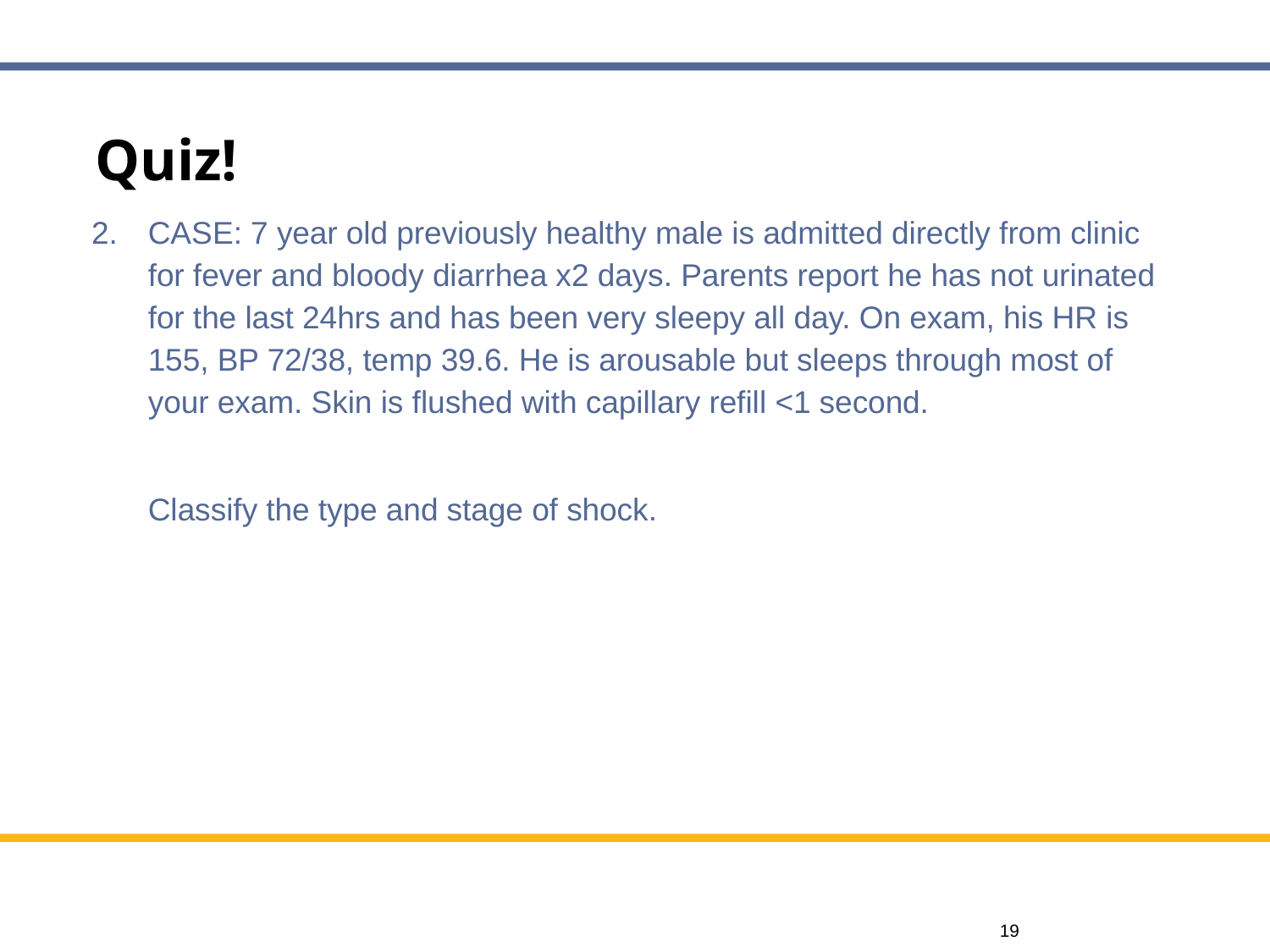

Quiz!
CASE: 7 year old previously healthy male is admitted directly from clinic for fever and bloody diarrhea x2 days. Parents report he has not urinated for the last 24hrs and has been very sleepy all day. On exam, his HR is 155, BP 72/38, temp 39.6. He is arousable but sleeps through most of your exam. Skin is flushed with capillary refill <1 second.
	Classify the type and stage of shock.
19

## Slide 20
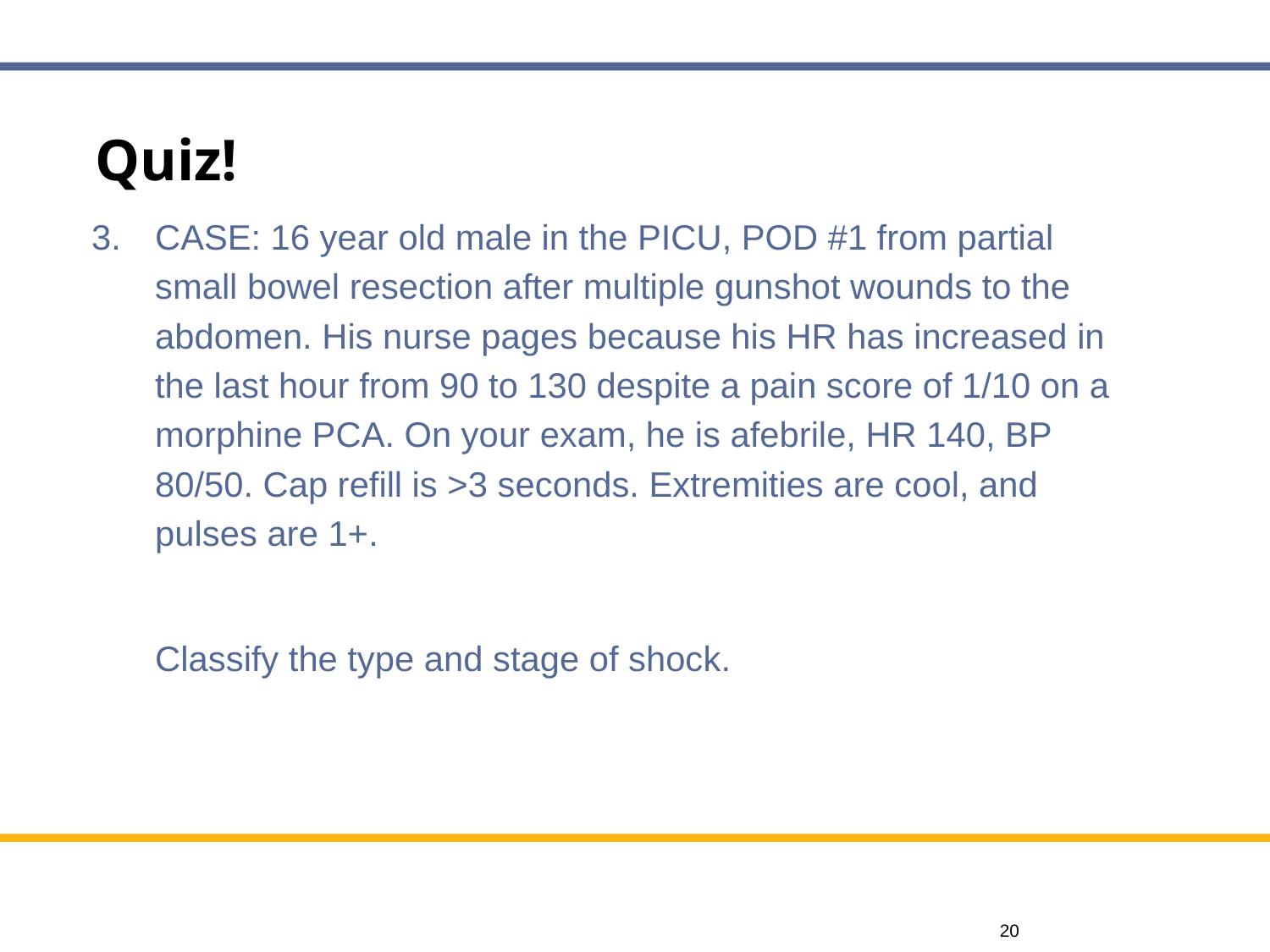

Quiz!
3.	CASE: 16 year old male in the PICU, POD #1 from partial small bowel resection after multiple gunshot wounds to the abdomen. His nurse pages because his HR has increased in the last hour from 90 to 130 despite a pain score of 1/10 on a morphine PCA. On your exam, he is afebrile, HR 140, BP 80/50. Cap refill is >3 seconds. Extremities are cool, and pulses are 1+.
	Classify the type and stage of shock.
20
