# Supplementary material for: Identification and Management of Pediatric Sepsis: A Medical Student Curricular Supplement for PICU and NICU Rotations
Source: MedEdPORTAL. 2021 Apr 23;17:11142. doi: 10.15766/mep_2374-8265.11142 (PMC8063627; doi:10.15766/mep_2374-8265.11142)
Supplement: Supplementary file 1 — Pre- & Posttest.docxModule 1 - Pediatric Shock.pptxScript 1 - Pediatric Shock.docxModule 2 - Pediatric Sepsis.pptxScript 2 - Pediatric Sepsis.docxModule 3 - Management of Sepsis & Septic Shock.pptxScript 3 - Management of Sepsis & Septic Shock. docxModule 4 - Hemodynamics & Pressor Support.pptxScript 4 - Hemodynamics & Pressor Support.docxSimulation Case 1.docxSimulation Case 2.docxSimulation Case 3.docxPostsimulation Review Quiz.pptx [file mep_2374-8265.11142-s001.zip › C. Script 1 - Pediatric Shock.docx]

**Pediatric Shock**

1. This education module continues our Pediatric Sepsis online curriculum. Our topic here is an introduction to pediatric shock.
2. The learning objectives are:
   - Define shock
   - Identify the stages of shock
   - Identify the classifications of shock
   - Recognize the importance of early intervention in management of shock
3. So, what is shock? Shock is a state that occurs when perfusion is not adequate to meet tissue demands. It is a result of inadequate cardiac output. The 2020 sepsis guidelines also include “sepsis associated organ dysfunction” in the definition of shock. This includes children who have severe infection that can lead to either cardiovascular or non-cardiovascular organ dysfunction, or both.
4. Now before we move on, it is important to review the concept of cardiac output. Cardiac output is the product of heart rate and stroke volume, commonly represented with the formula shown here. Cardiac output is important because this is how hemodynamic stability is established and maintained. Blood pressure is a means to characterize hemodynamic stability. It is the product of cardiac output and systemic vascular resistance.
5. PEARL. Now you’ve seen in this slide before, but it’s important to reinforce that children can be in a state of shock without becoming hypotensive. Signs of poor perfusion are consistent with shock as children can maintain blood pressure by increasing their heart rates and systemic vascular resistance.
6. This figure here shows the steps that occur in a state of shock. Shock is a progressive process that often results from inadequate cardiac output, causing a systemic reduction in tissue perfusion and decreased oxygen delivery to tissues. This leads to a shift to anaerobic metabolism and subsequent lactic acidosis.
7. Initially shock is a process that can be reversed. However, if your interventions are ineffective or occur in a delayed fashion, irreversible changes occur. These irreversible changes include cell death, organ failure, cardiac arrest, and death. What is our take-away message? Do something for your patient while this process is still reversible!
8. So, just as there are stages of sepsis, there are also stages of shock. The first stage is compensated shock. In this stage, the body is able to maintain some tissue perfusion. Cardiac output and systemic vascular resistance maintain a normal blood pressure. What you’ll find on exam is a patient that is tachycardic and has decreased urine output. The current 2020 Sepsis Guidelines no longer supports isolated clinical signs to apply the terms “hot” or “cold” shock categories to children.
9. The next stage is uncompensated shock. Now the body is starting to shut down. On exam, you will observe poor perfusion, *no* urine output, hypotension, bradycardia and arrhythmias, altered mental status, and even potentially loss of consciousness. The patient’s laboratory values will show a worsening lactic acidosis that may not respond to your interventions.
10. Lastly, there is irreversible shock, at which time organ failure, cardiac arrest, and death will occur. No interventions are effective in this stage, and lab values are severely deranged.
11. PEARL – Here’s another pediatric pearl for you! Did you know – Children, unlike adults, can lose up to 25-30% of their blood volume with only minimal changes in vital signs. Hypotension is a late and ominous finding in children. Similar to the previous pearl that we shared, this is clearly an important piece of information. Your goal is to recognize a child in shock *before* he/she becomes hypotensive, and as I stressed before, intervene early!
12. Now we’ll discuss the different classifications of shock. These include hypovolemic, distributive, cardiogenic, and obstructive shock.
13. It is important to know the different classifications of shock because they can all play a role in septic shock. It is best to think of septic shock as a multifactorial process where you may see features characteristic of hypovolemic, cardiogenic, and distributive shock.
14. Let’s start with hypovolemic shock. This can be characterized as decreased preload due to volume loss. It can be caused by hemorrhage or excessive bleeding, dehydration typically through GI losses or inadequate intake. Patients may also have insensible losses as seen with burns covering a significant percentage of body surface area, as well as from 3^rd^ spacing of fluids into extravascular compartments. Third spacing causes a relative *intra*vascular volume depletion.
15. Now let’s discuss distributive shock. Distributive shock is characterized by vasodilation that leads to decreased vascular resistance. Common causes include sepsis, anaphylaxis, and acute spinal cord injury. This type of distributive shock known as neurogenic shock results from loss of vascular tone in the periphery.
16. The next type of shock we will discuss is cardiogenic shock. This is essentially failure of the heart to pump effectively. The resulting decreased cardiac contractility leads to decreased cardiac output. Cardiogenic shock can be caused by any of the following:
    - Myocarditis
    - Cardiomyopathy
    - Myocardial ischemia
    - Congenital heart disease with heart failure
    - Arrhythmia
17. The last type of shock we will discuss is obstructive shock. This occurs when outflow of blood from the left or right heart is physically blocked. This can be caused by a tension pneumothorax, a massive pulmonary embolus, cardiac tamponade, or a ventricular outflow tract obstruction, such as that seen with severe coarctation or aortic stenosis.
18. We finish with a short quiz! There are 3 cases here for you to think about in advance, and they will be discussed at your simulation session.
