# Supplementary material for: Identification and Management of Pediatric Sepsis: A Medical Student Curricular Supplement for PICU and NICU Rotations
Source: MedEdPORTAL. 2021 Apr 23;17:11142. doi: 10.15766/mep_2374-8265.11142 (PMC8063627; doi:10.15766/mep_2374-8265.11142)
Supplement: Supplementary file 1 — Pre- & Posttest.docxModule 1 - Pediatric Shock.pptxScript 1 - Pediatric Shock.docxModule 2 - Pediatric Sepsis.pptxScript 2 - Pediatric Sepsis.docxModule 3 - Management of Sepsis & Septic Shock.pptxScript 3 - Management of Sepsis & Septic Shock. docxModule 4 - Hemodynamics & Pressor Support.pptxScript 4 - Hemodynamics & Pressor Support.docxSimulation Case 1.docxSimulation Case 2.docxSimulation Case 3.docxPostsimulation Review Quiz.pptx [file mep_2374-8265.11142-s001.zip › D. Module 2 - Pediatric Sepsis.pptx]

## Slide 1
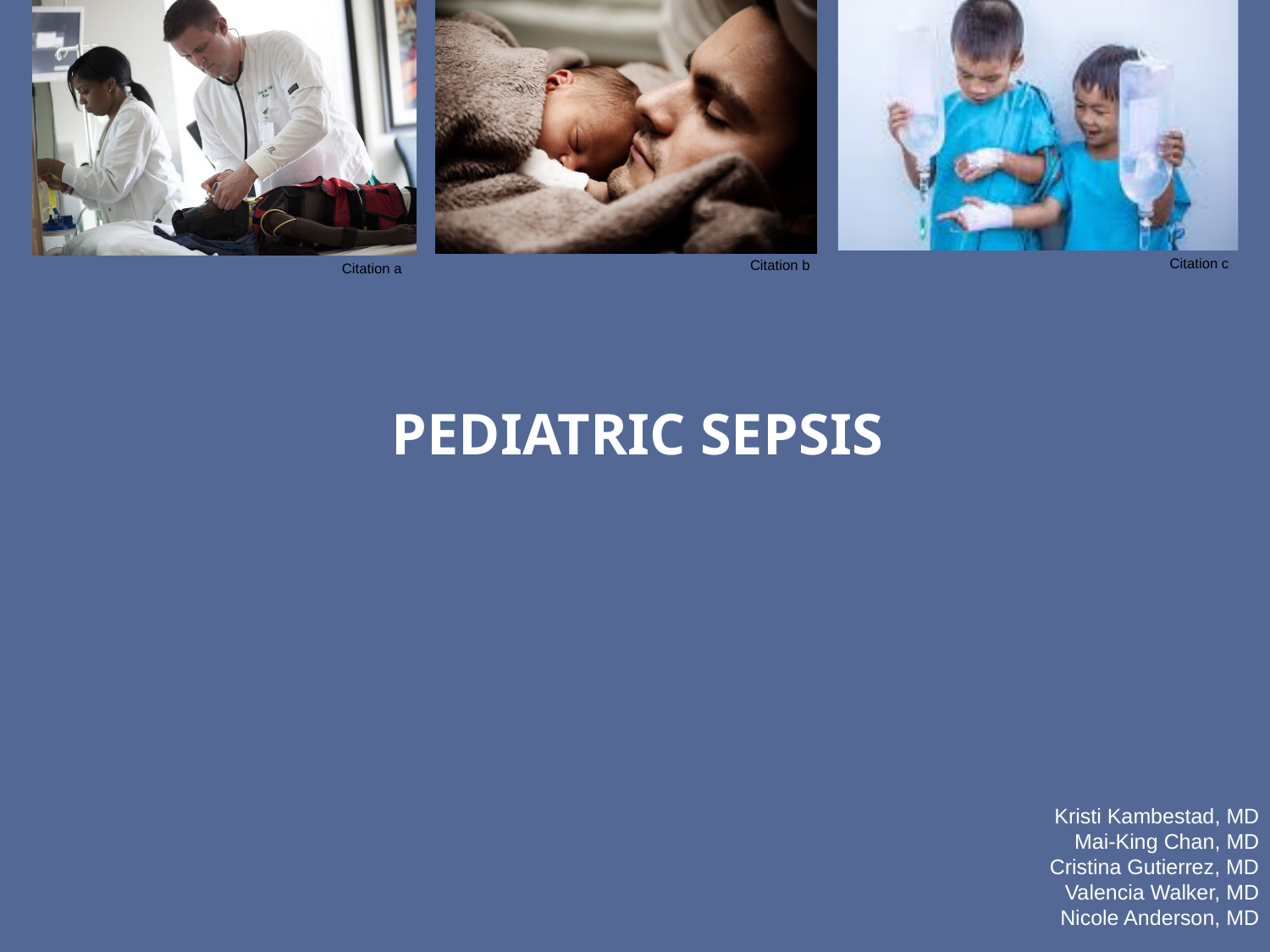

Citation c
Citation b
Citation a
# PEDIATRIC SEPSIS
Kristi Kambestad, MD
Mai-King Chan, MD
Cristina Gutierrez, MD
Valencia Walker, MD
Nicole Anderson, MD

## Slide 2
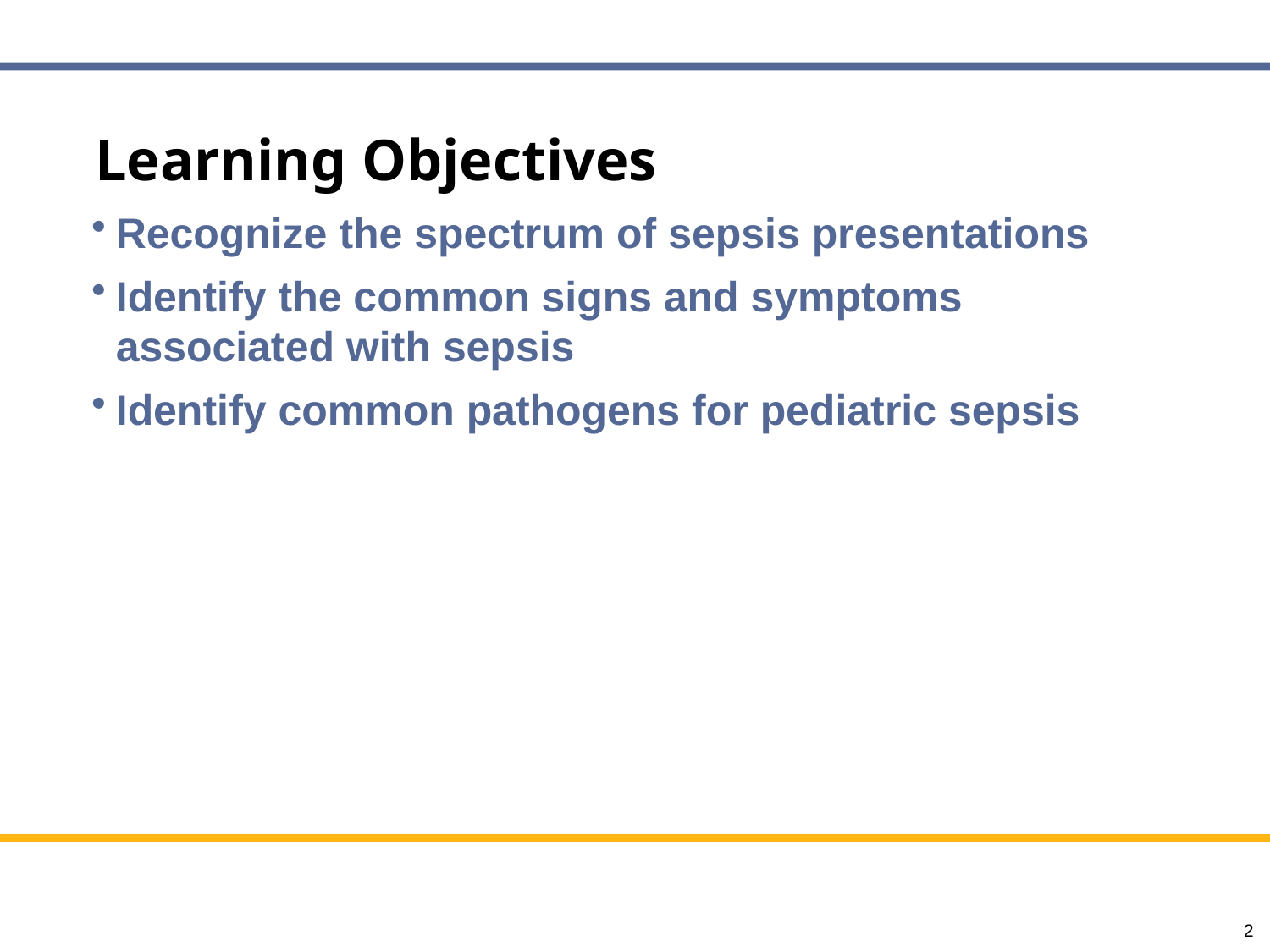

# Learning Objectives
Recognize the spectrum of sepsis presentations
Identify the common signs and symptoms associated with sepsis
Identify common pathogens for pediatric sepsis
2

## Slide 3
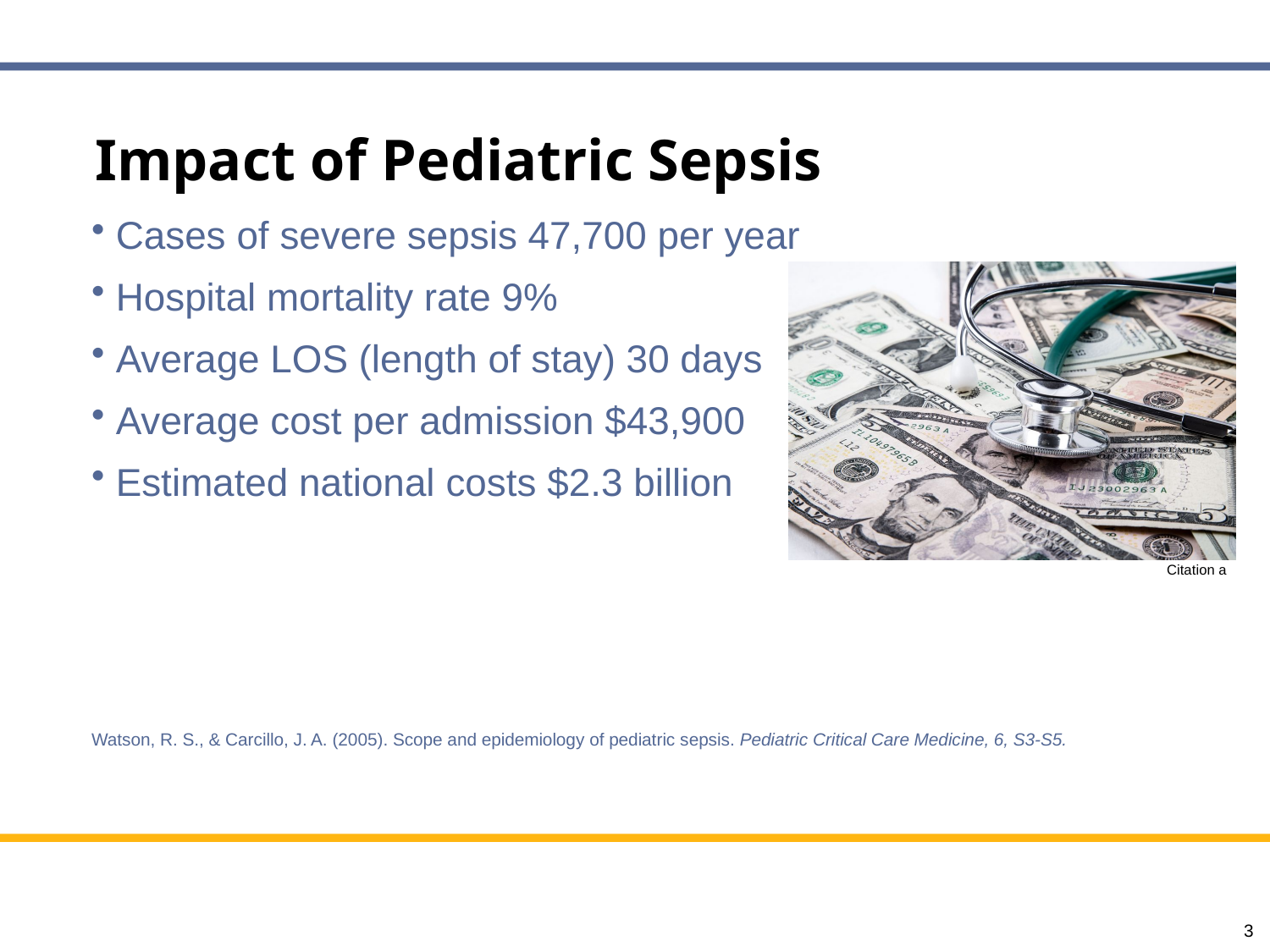

# Impact of Pediatric Sepsis
Cases of severe sepsis 47,700 per year
Hospital mortality rate 9%
Average LOS (length of stay) 30 days
Average cost per admission $43,900
Estimated national costs $2.3 billion
Watson, R. S., & Carcillo, J. A. (2005). Scope and epidemiology of pediatric sepsis. Pediatric Critical Care Medicine, 6, S3‐S5.
Citation a
3

## Slide 4
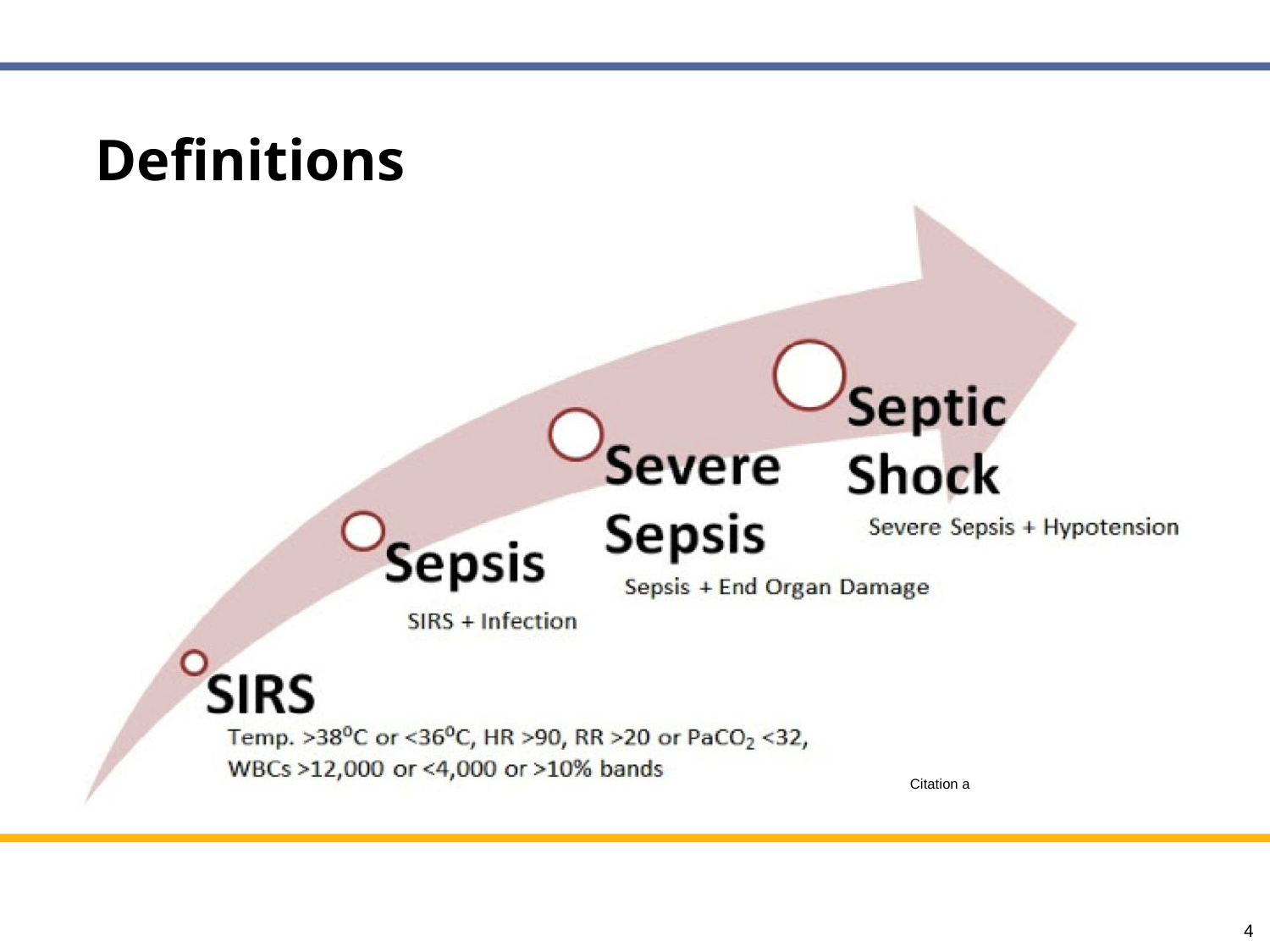

# Definitions
Citation a
4

## Slide 5
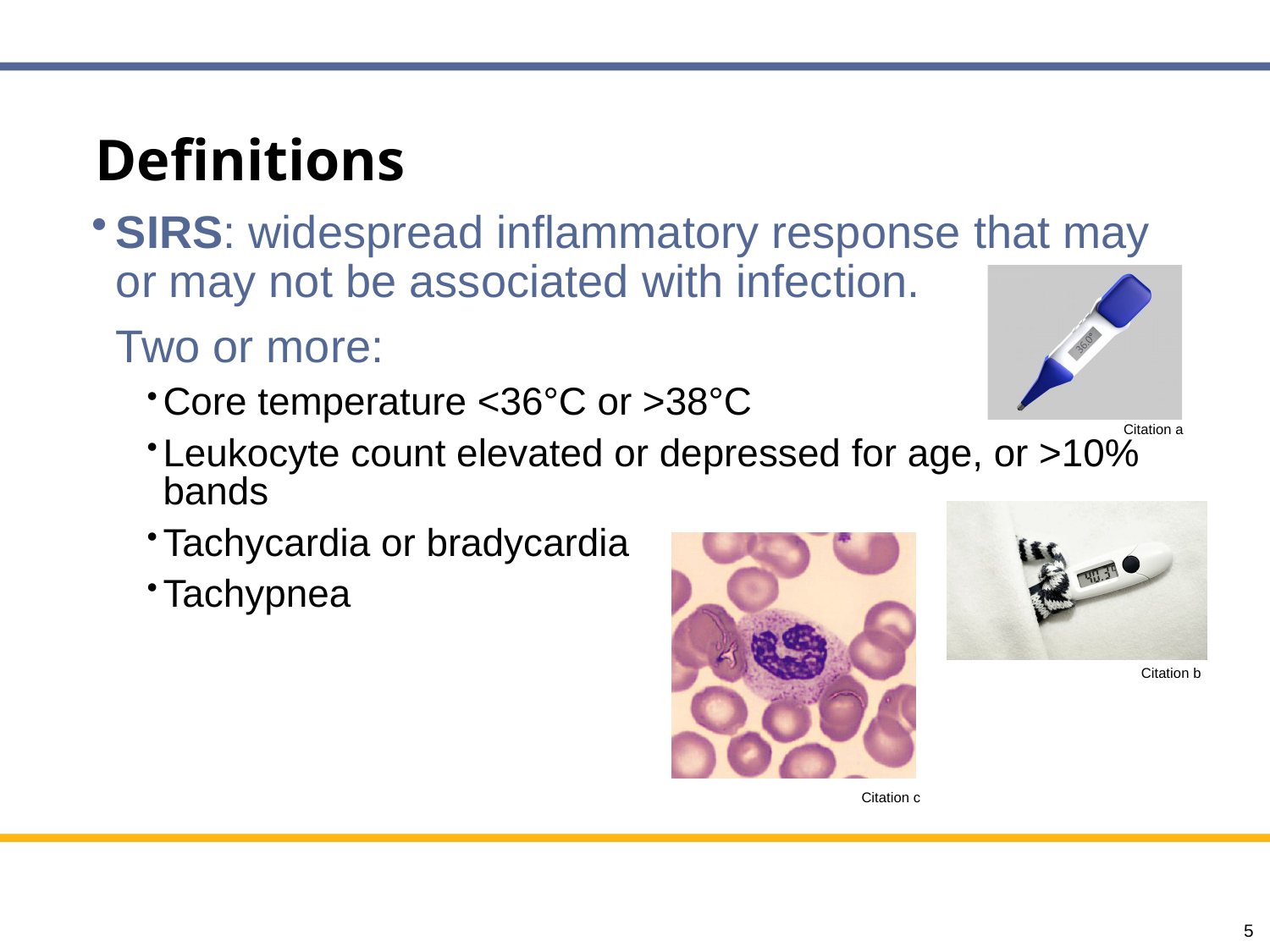

# Definitions
SIRS: widespread inflammatory response that may or may not be associated with infection.
	Two or more:
Core temperature <36°C or >38°C
Leukocyte count elevated or depressed for age, or >10% bands
Tachycardia or bradycardia
Tachypnea
Citation a
Citation b
Citation c
5

## Slide 6
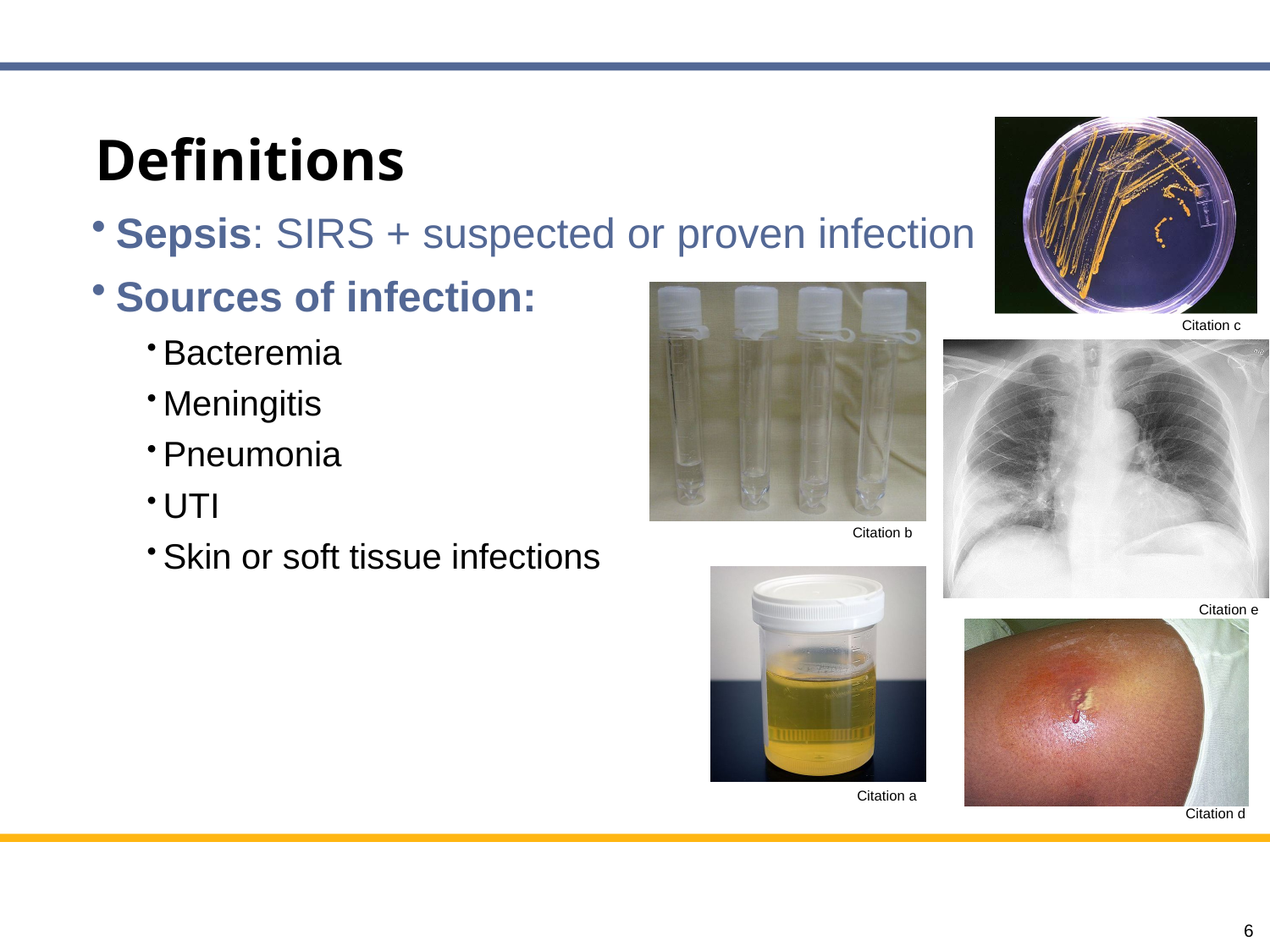

# Definitions
Sepsis: SIRS + suspected or proven infection
Sources of infection:
Bacteremia
Meningitis
Pneumonia
UTI
Skin or soft tissue infections
Citation c
Citation b
Citation e
Citation a
Citation d
6

## Slide 7
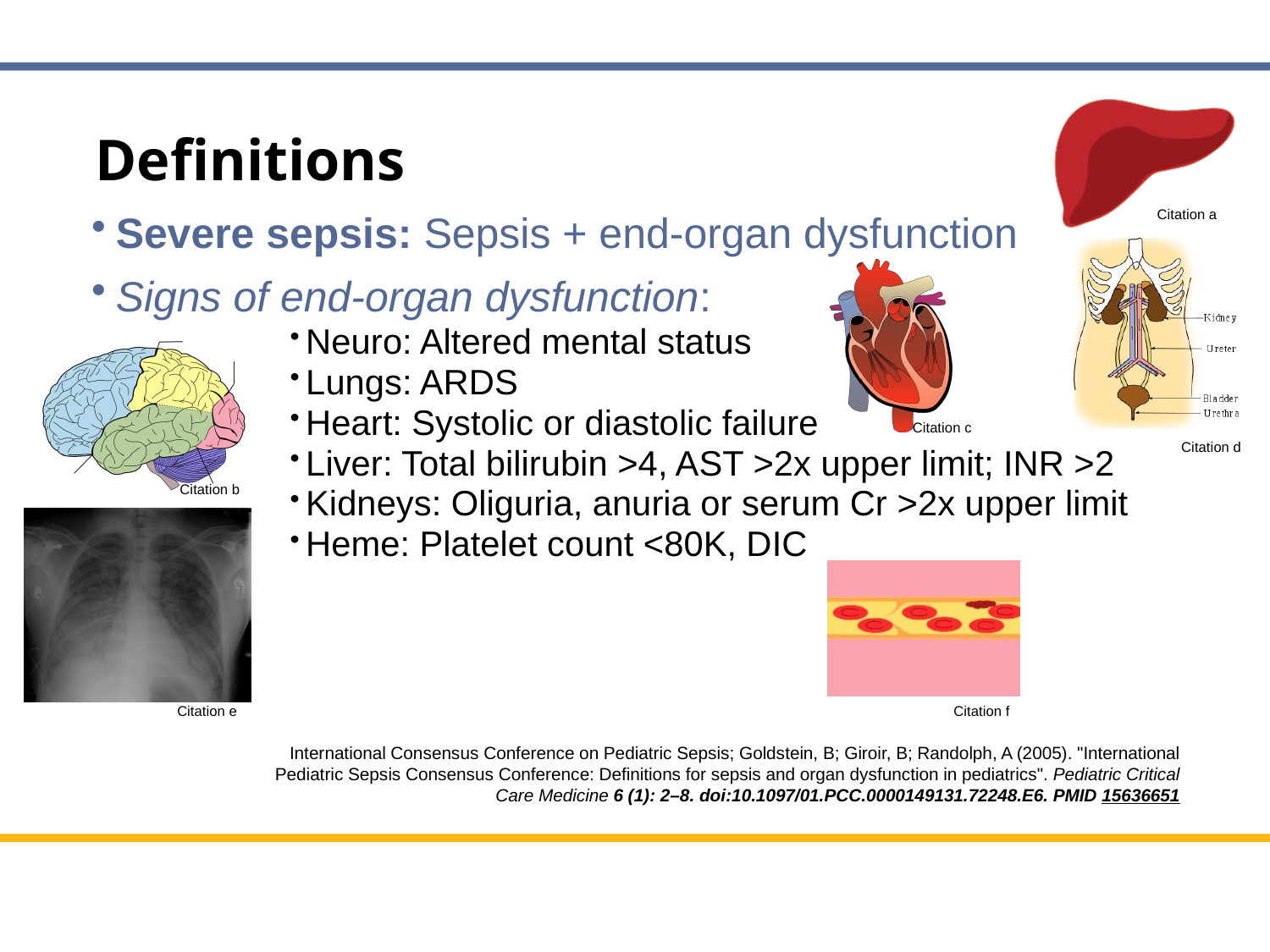

# Definitions
Citation a
Severe sepsis: Sepsis + end-organ dysfunction
Signs of end-organ dysfunction:
Neuro: Altered mental status
Lungs: ARDS
Heart: Systolic or diastolic failure
Liver: Total bilirubin >4, AST >2x upper limit; INR >2
Kidneys: Oliguria, anuria or serum Cr >2x upper limit
Heme: Platelet count <80K, DIC
Citation c
Citation d
Citation b
Citation e
Citation f
International Consensus Conference on Pediatric Sepsis; Goldstein, B; Giroir, B; Randolph, A (2005). "International Pediatric Sepsis Consensus Conference: Definitions for sepsis and organ dysfunction in pediatrics". Pediatric Critical Care Medicine 6 (1): 2–8. doi:10.1097/01.PCC.0000149131.72248.E6. PMID 15636651

## Slide 8
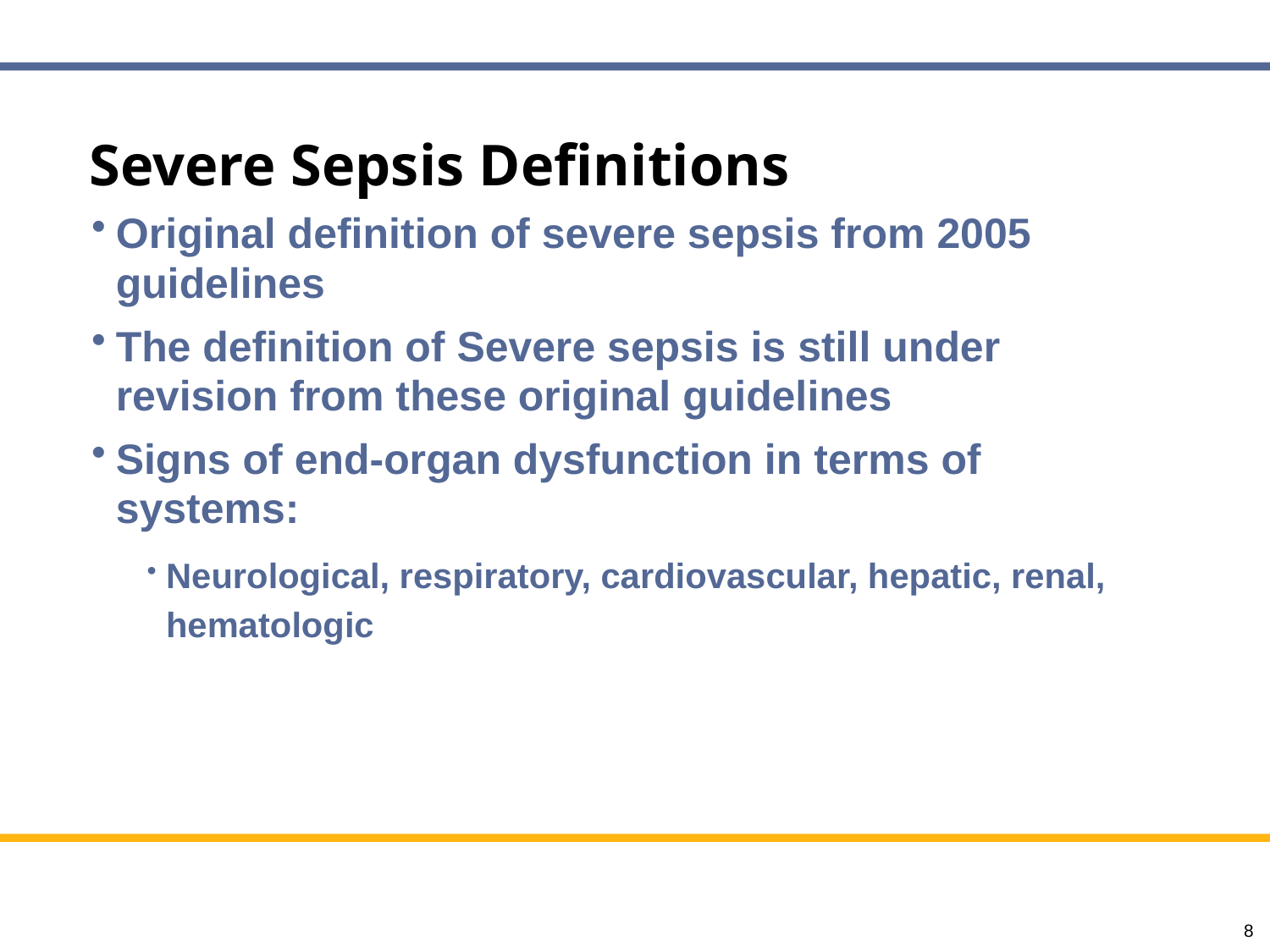

Severe Sepsis Definitions
Original definition of severe sepsis from 2005 guidelines
The definition of Severe sepsis is still under revision from these original guidelines
Signs of end-organ dysfunction in terms of systems:
Neurological, respiratory, cardiovascular, hepatic, renal, hematologic
8

## Slide 9
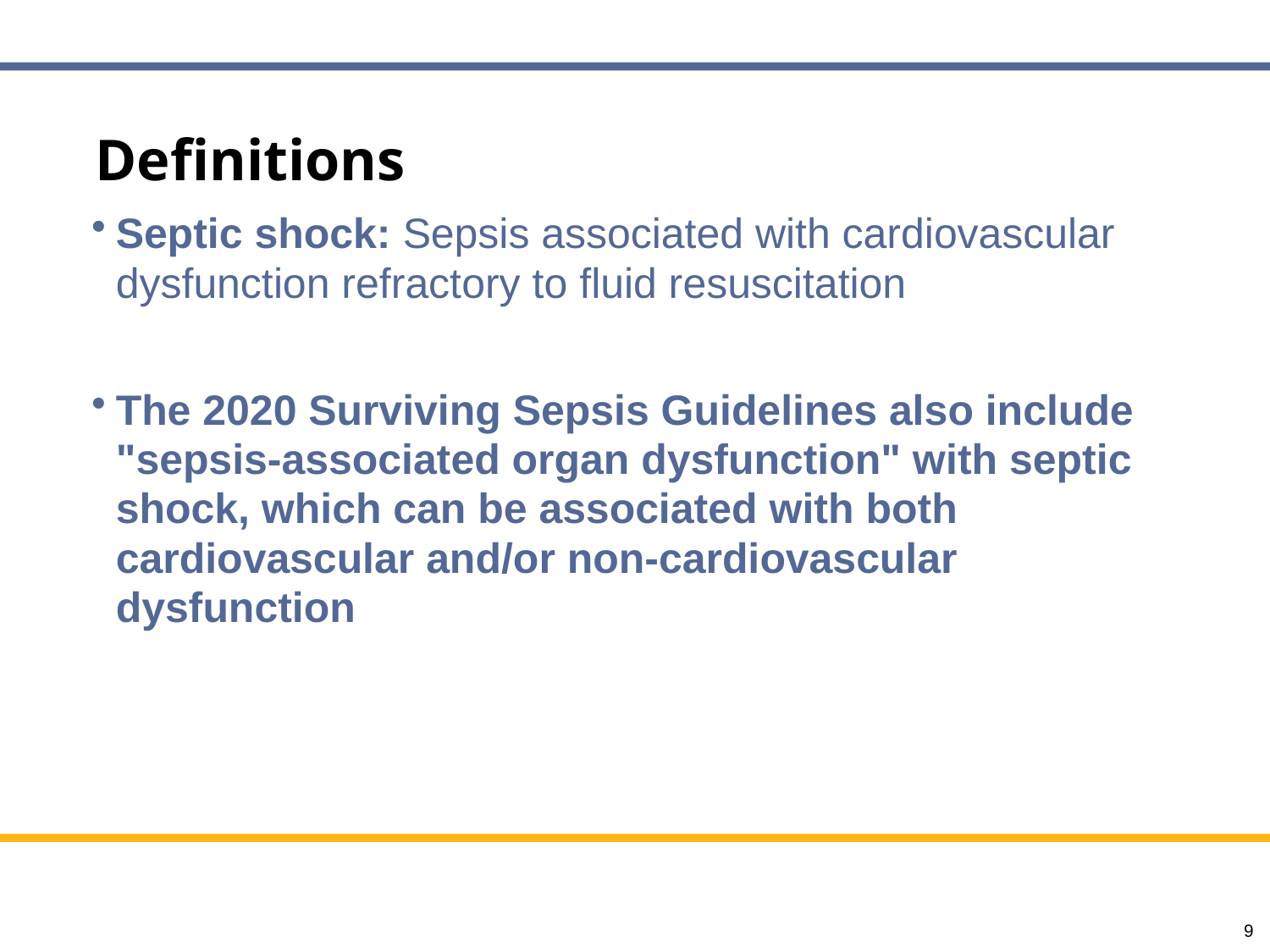

# Definitions
Septic shock: Sepsis associated with cardiovascular dysfunction refractory to fluid resuscitation
The 2020 Surviving Sepsis Guidelines also include "sepsis-associated organ dysfunction" with septic shock, which can be associated with both cardiovascular and/or non-cardiovascular dysfunction
9

## Slide 10
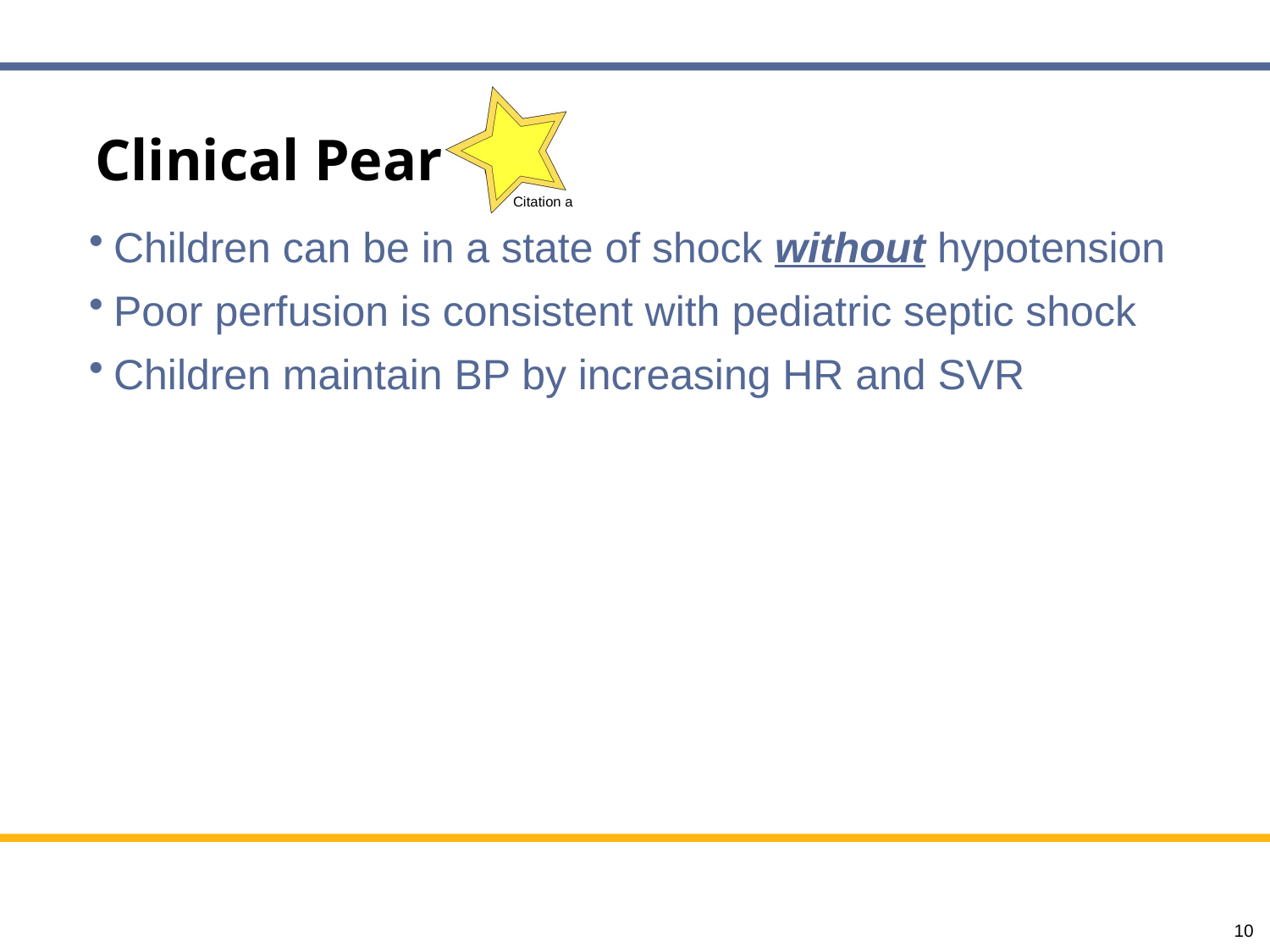

# Clinical Pearl
Citation a
Children can be in a state of shock without hypotension
Poor perfusion is consistent with pediatric septic shock
Children maintain BP by increasing HR and SVR
10

## Slide 11
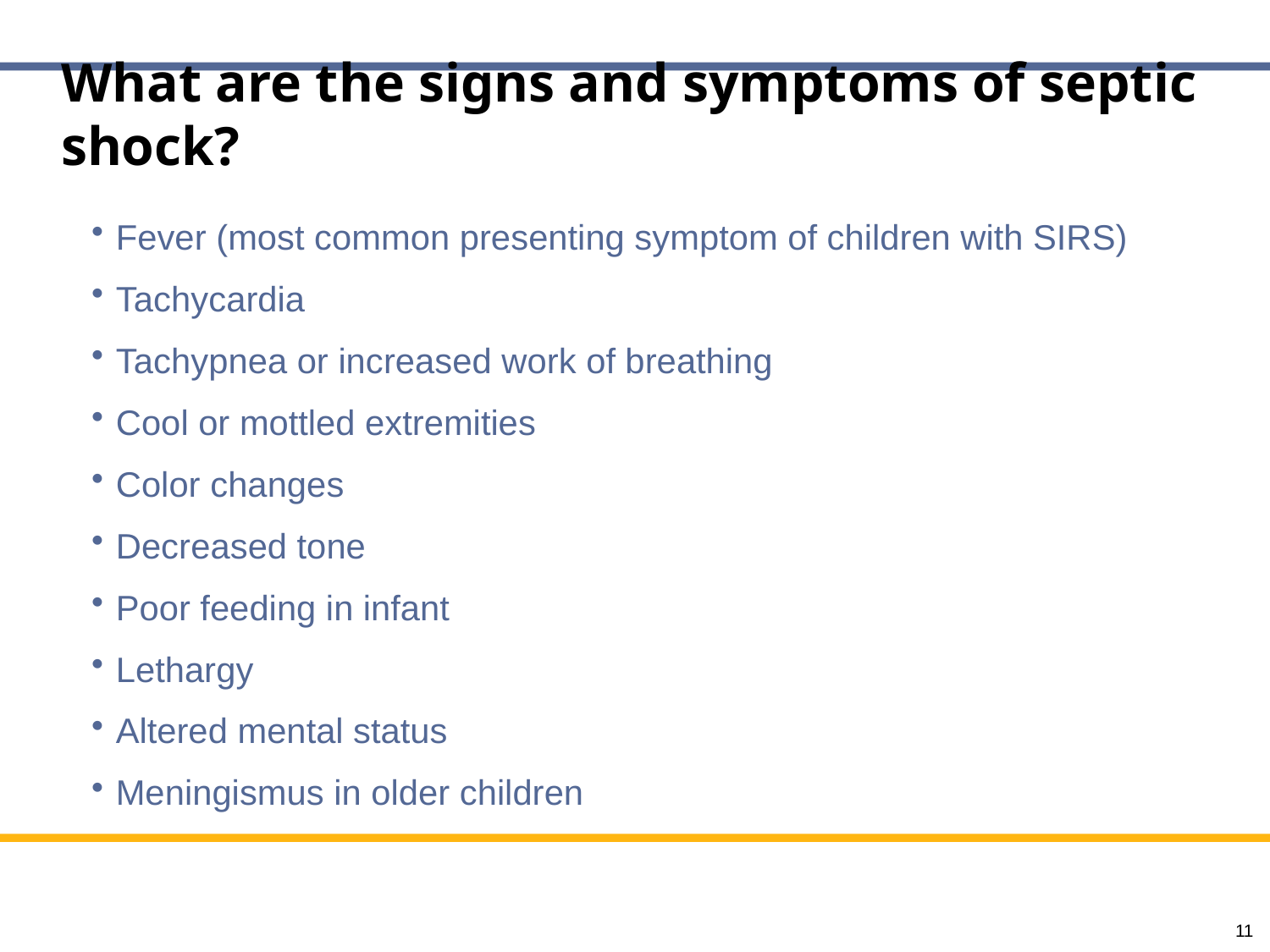

# What are the signs and symptoms of septic shock?
Fever (most common presenting symptom of children with SIRS)
Tachycardia
Tachypnea or increased work of breathing
Cool or mottled extremities
Color changes
Decreased tone
Poor feeding in infant
Lethargy
Altered mental status
Meningismus in older children
11

## Slide 12
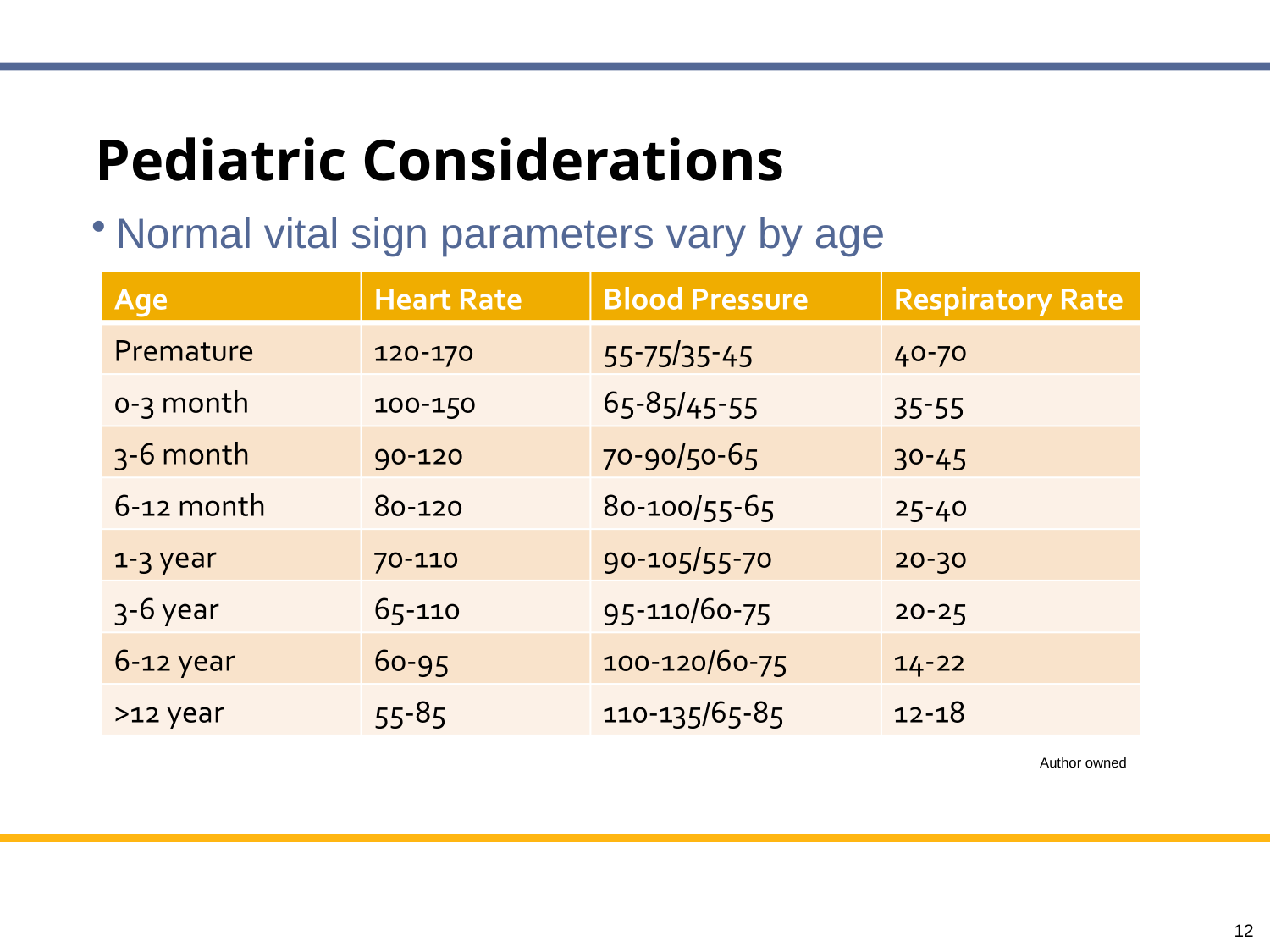

# Pediatric Considerations
Normal vital sign parameters vary by age
Author owned
12

## Slide 13
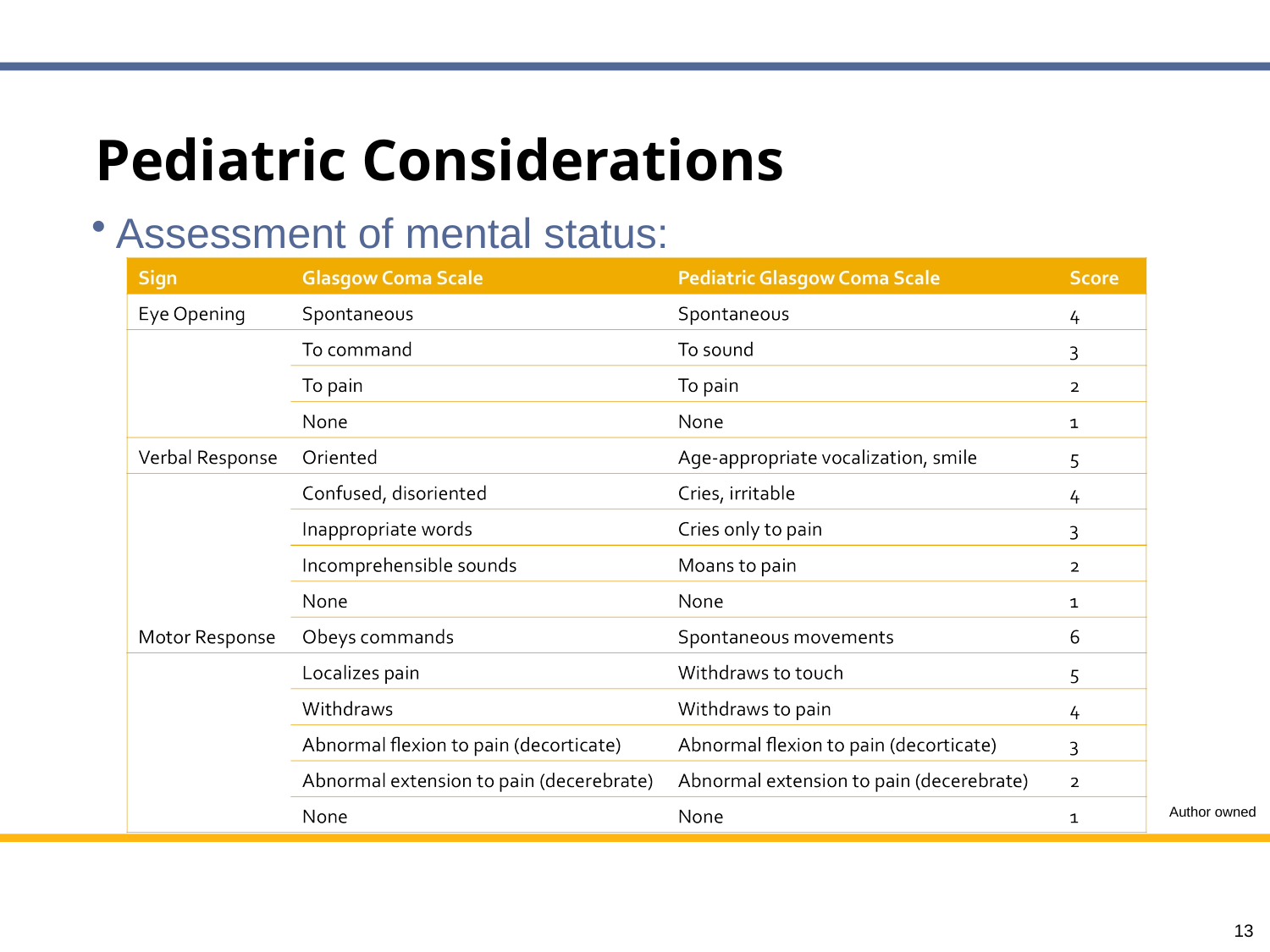

# Pediatric Considerations
Assessment of mental status:
Author owned
13

## Slide 14
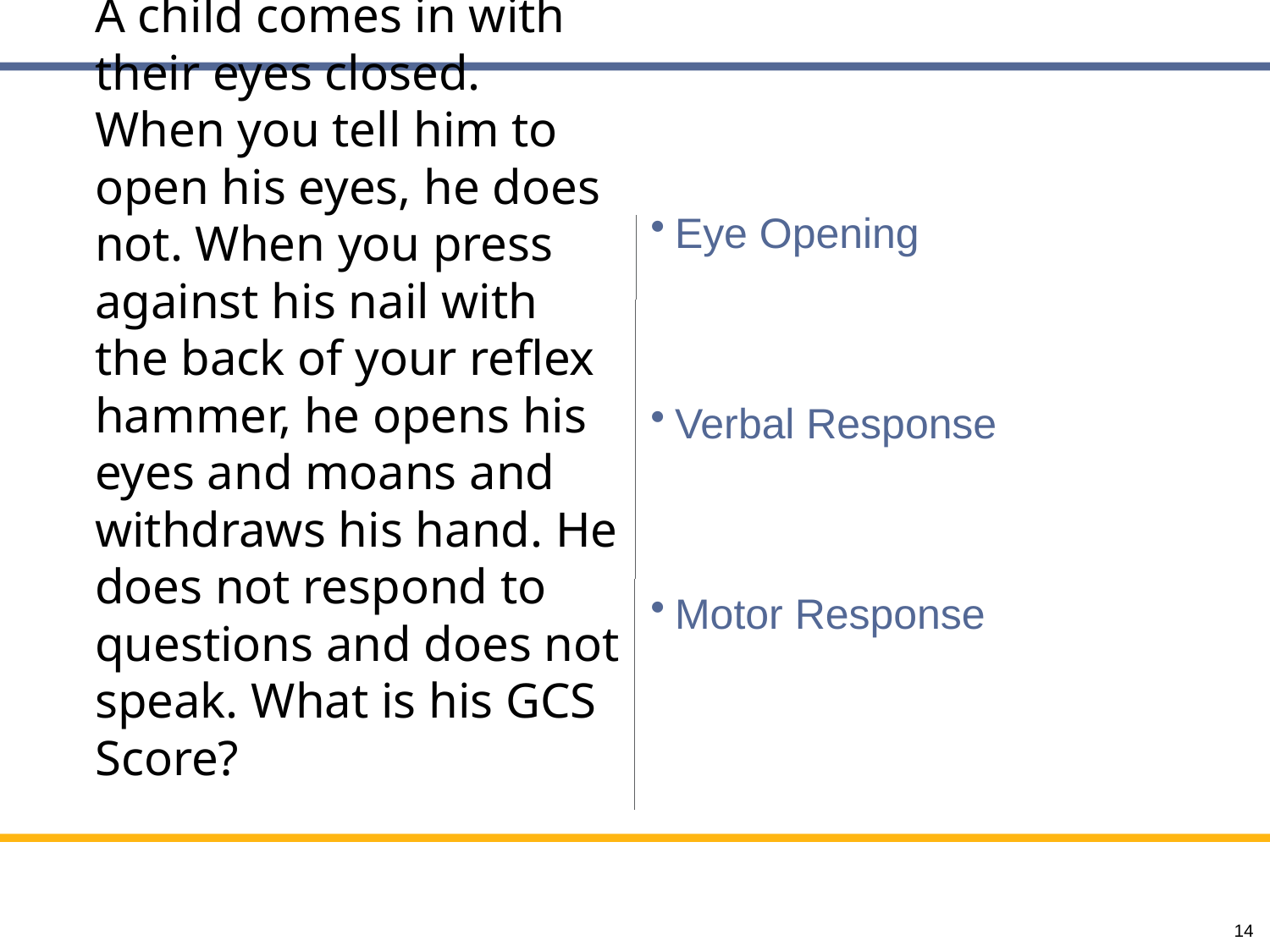

# A child comes in with their eyes closed. When you tell him to open his eyes, he does not. When you press against his nail with the back of your reflex hammer, he opens his eyes and moans and withdraws his hand. He does not respond to questions and does not speak. What is his GCS Score?
Eye Opening
Verbal Response
Motor Response
14

## Slide 15
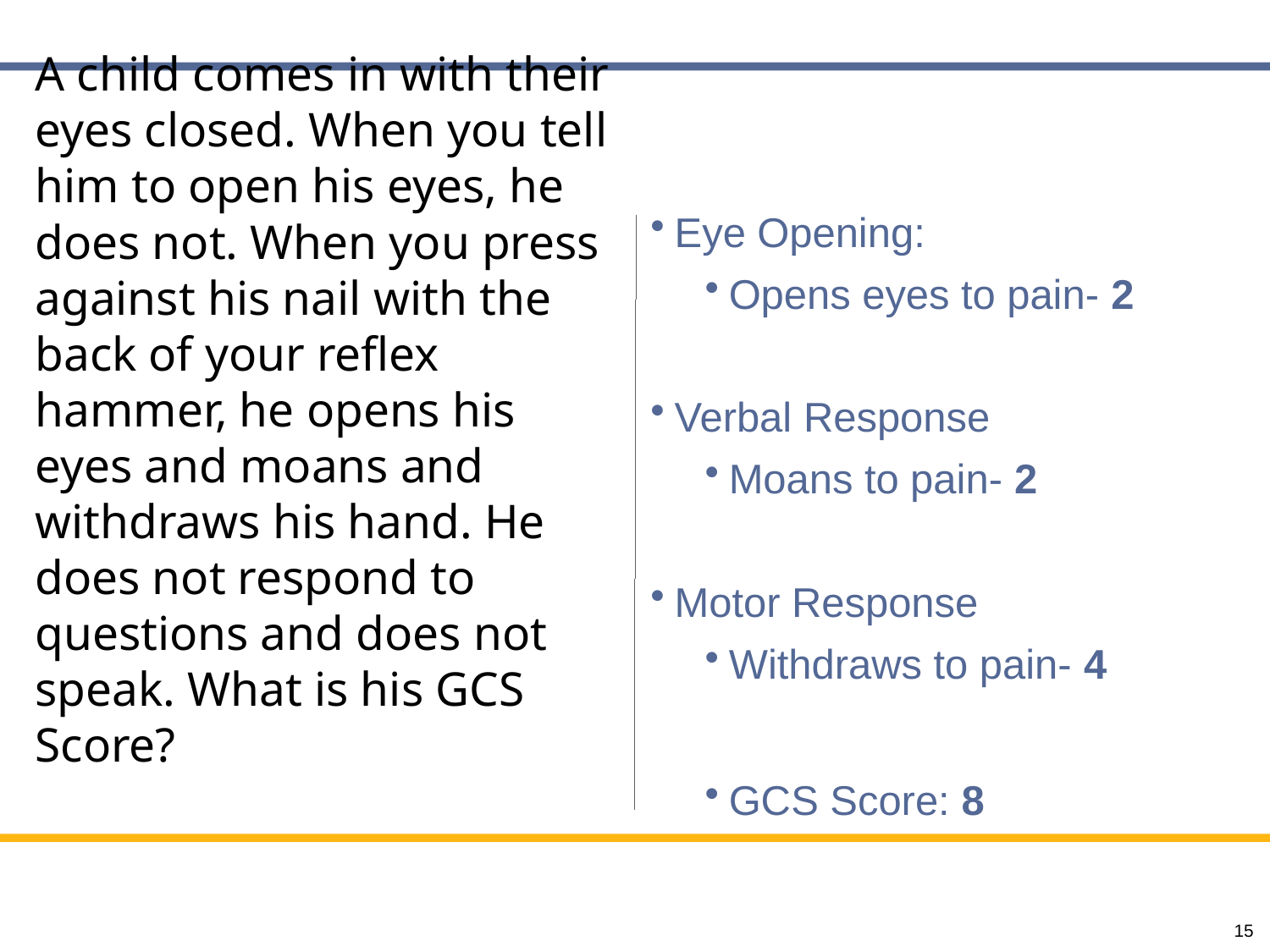

# A child comes in with their eyes closed. When you tell him to open his eyes, he does not. When you press against his nail with the back of your reflex hammer, he opens his eyes and moans and withdraws his hand. He does not respond to questions and does not speak. What is his GCS Score?
Eye Opening:
Opens eyes to pain- 2
Verbal Response
Moans to pain- 2
Motor Response
Withdraws to pain- 4
GCS Score: 8
15

## Slide 16
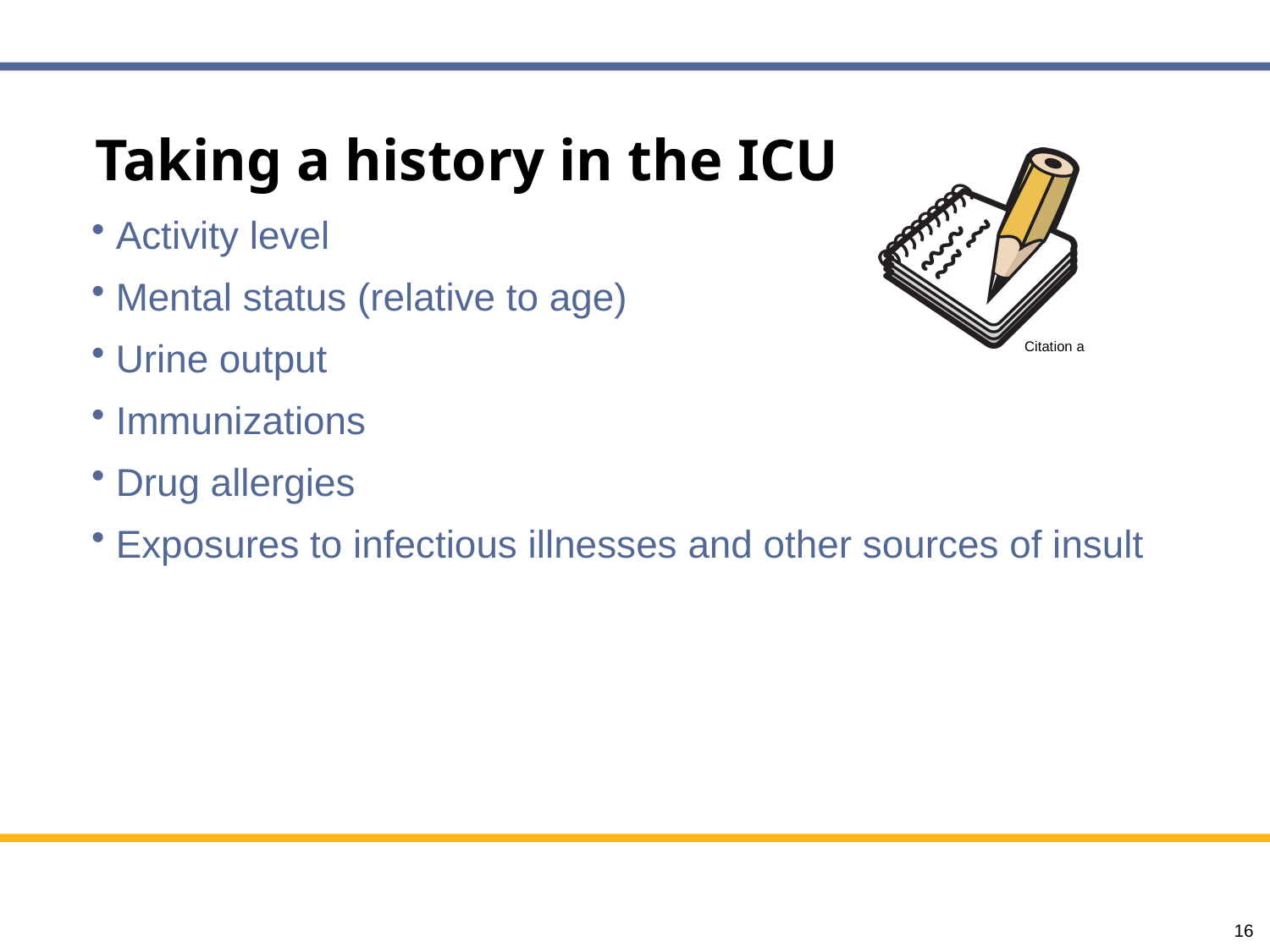

# Taking a history in the ICU
Activity level
Mental status (relative to age)
Urine output
Immunizations
Drug allergies
Exposures to infectious illnesses and other sources of insult
Citation a
16

## Slide 17
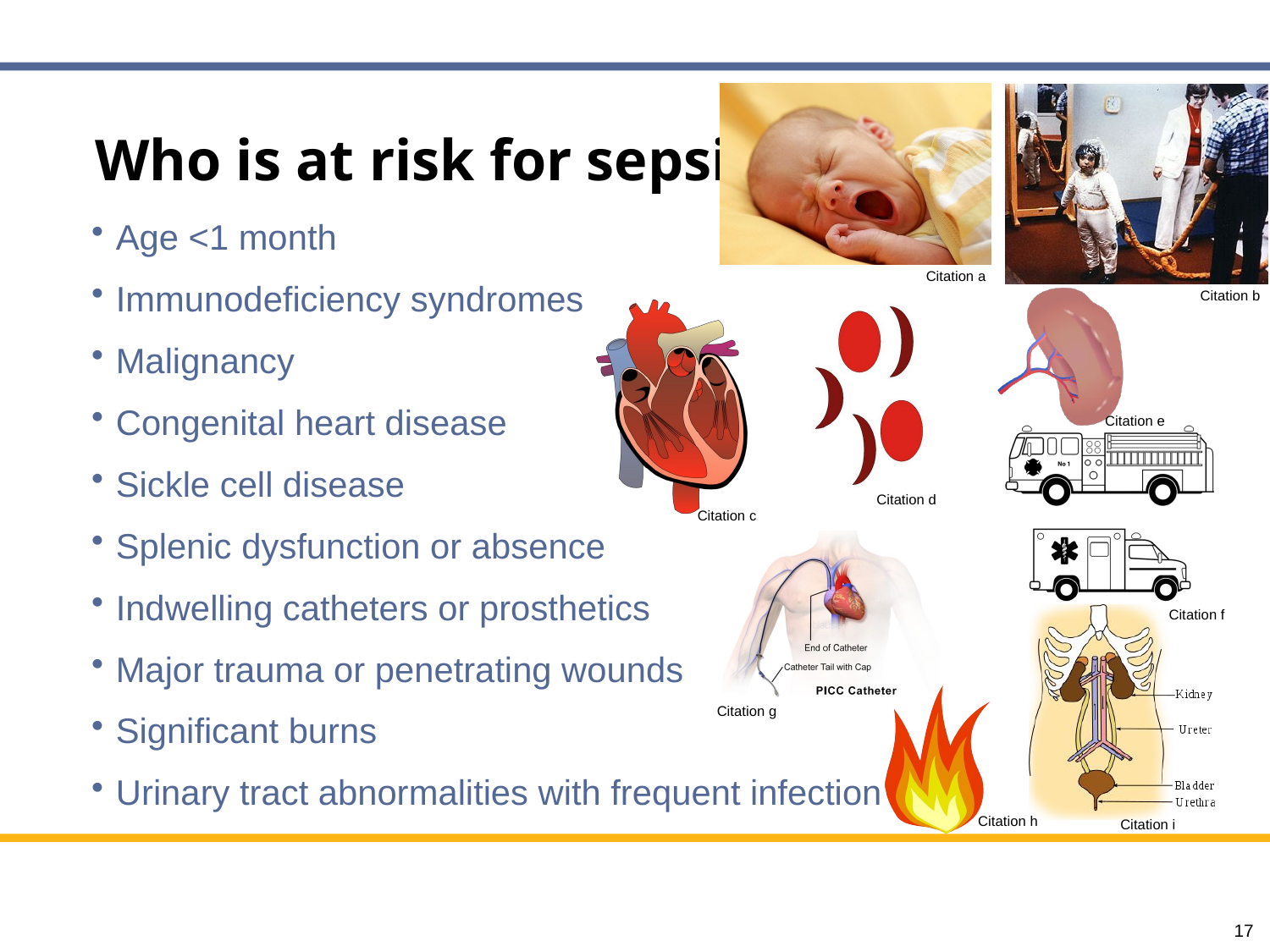

# Who is at risk for sepsis?
Age <1 month
Immunodeficiency syndromes
Malignancy
Congenital heart disease
Sickle cell disease
Splenic dysfunction or absence
Indwelling catheters or prosthetics
Major trauma or penetrating wounds
Significant burns
Urinary tract abnormalities with frequent infection
Citation a
Citation b
Citation e
Citation d
Citation c
Citation f
Citation g
Citation h
Citation i
17

## Slide 18
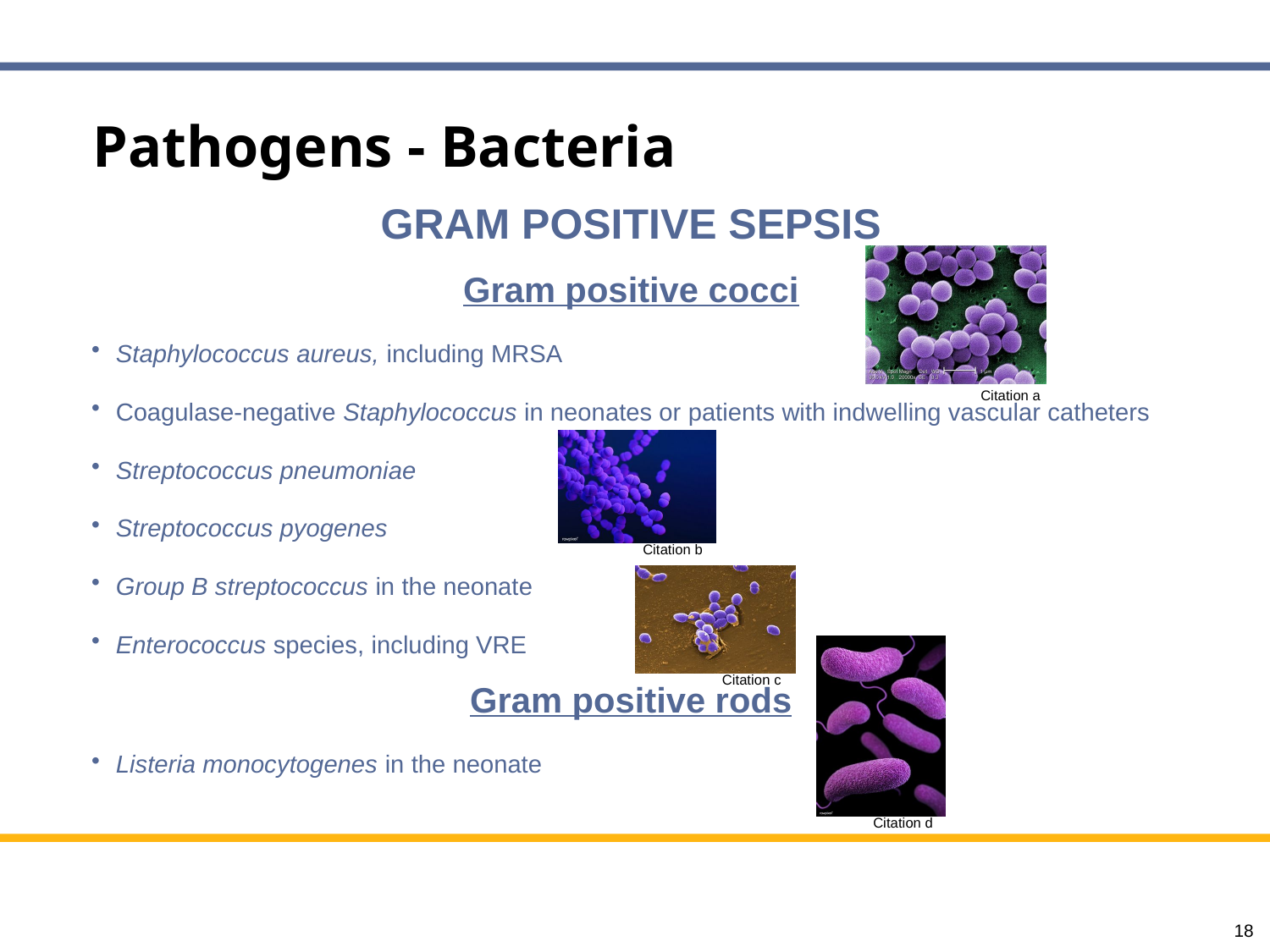

# Pathogens - Bacteria
GRAM POSITIVE SEPSIS
Gram positive cocci
Staphylococcus aureus, including MRSA
Coagulase-negative Staphylococcus in neonates or patients with indwelling vascular catheters
Streptococcus pneumoniae
Streptococcus pyogenes
Group B streptococcus in the neonate
Enterococcus species, including VRE
Gram positive rods
Listeria monocytogenes in the neonate
Citation a
Citation b
Citation c
Citation d
18

## Slide 19
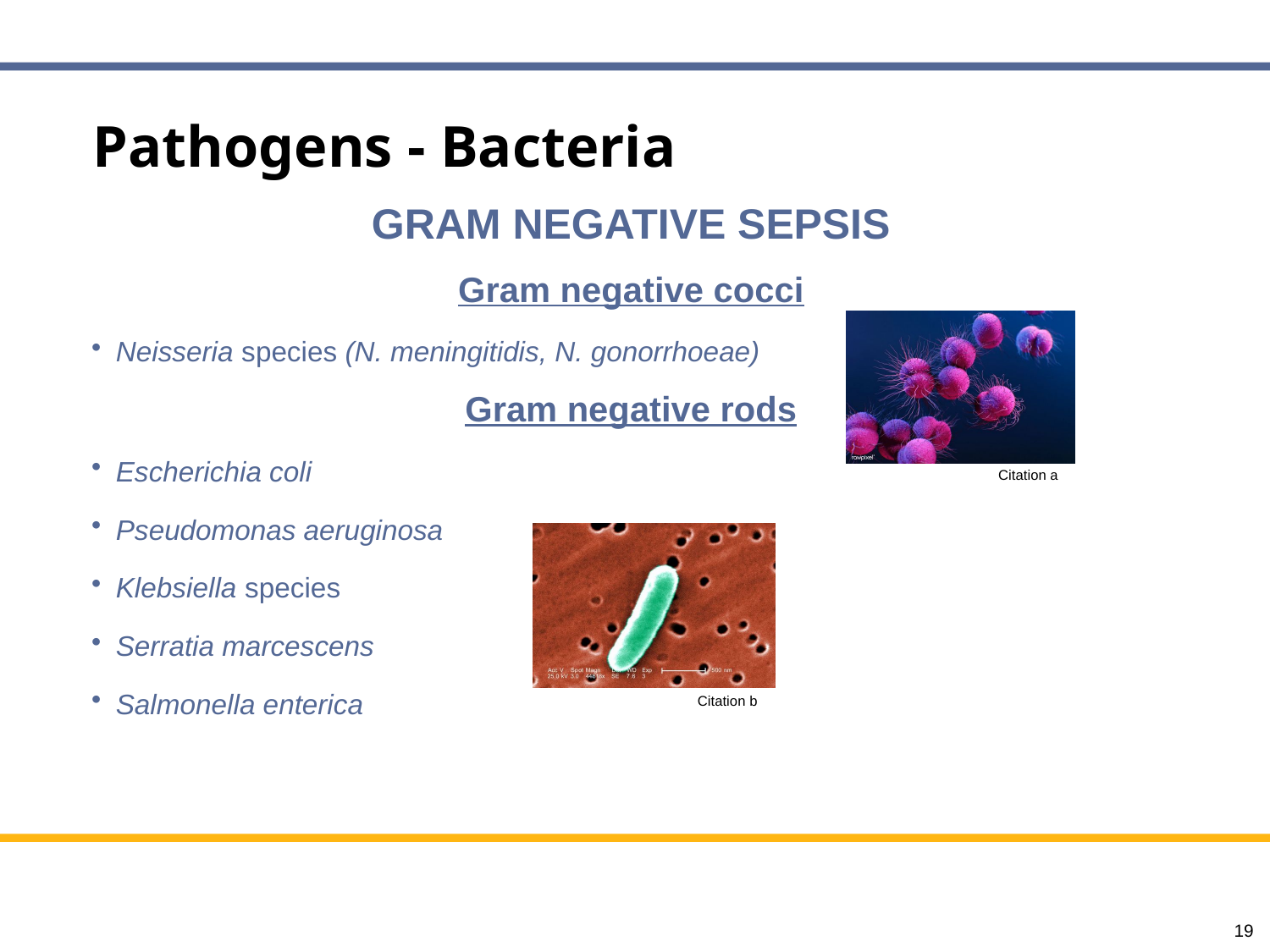

# Pathogens - Bacteria
GRAM NEGATIVE SEPSIS
Gram negative cocci
Neisseria species (N. meningitidis, N. gonorrhoeae)
Gram negative rods
Escherichia coli
Pseudomonas aeruginosa
Klebsiella species
Serratia marcescens
Salmonella enterica
Citation a
Citation b
19

## Slide 20
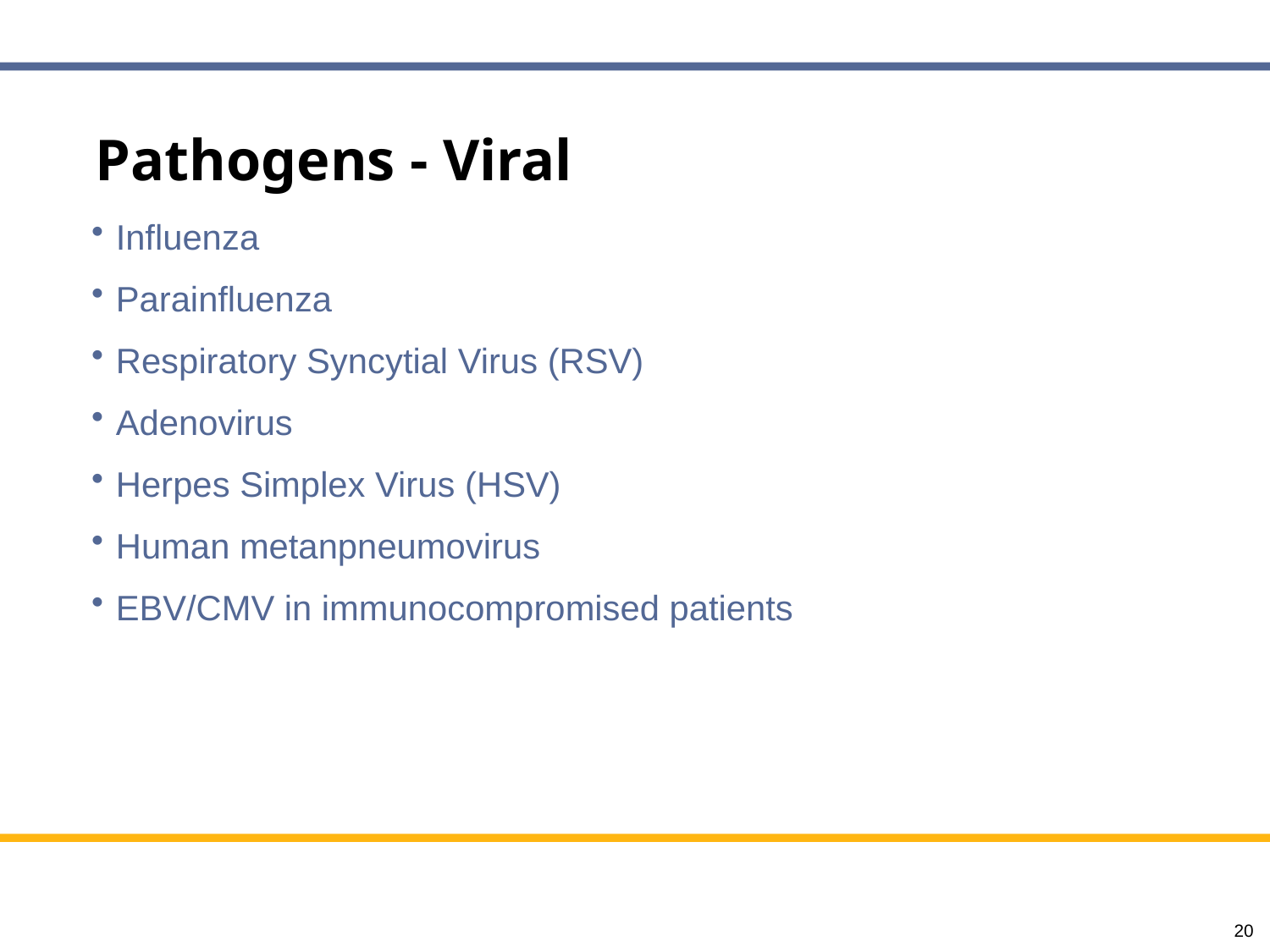

# Pathogens - Viral
Influenza
Parainfluenza
Respiratory Syncytial Virus (RSV)
Adenovirus
Herpes Simplex Virus (HSV)
Human metanpneumovirus
EBV/CMV in immunocompromised patients
20

## Slide 21
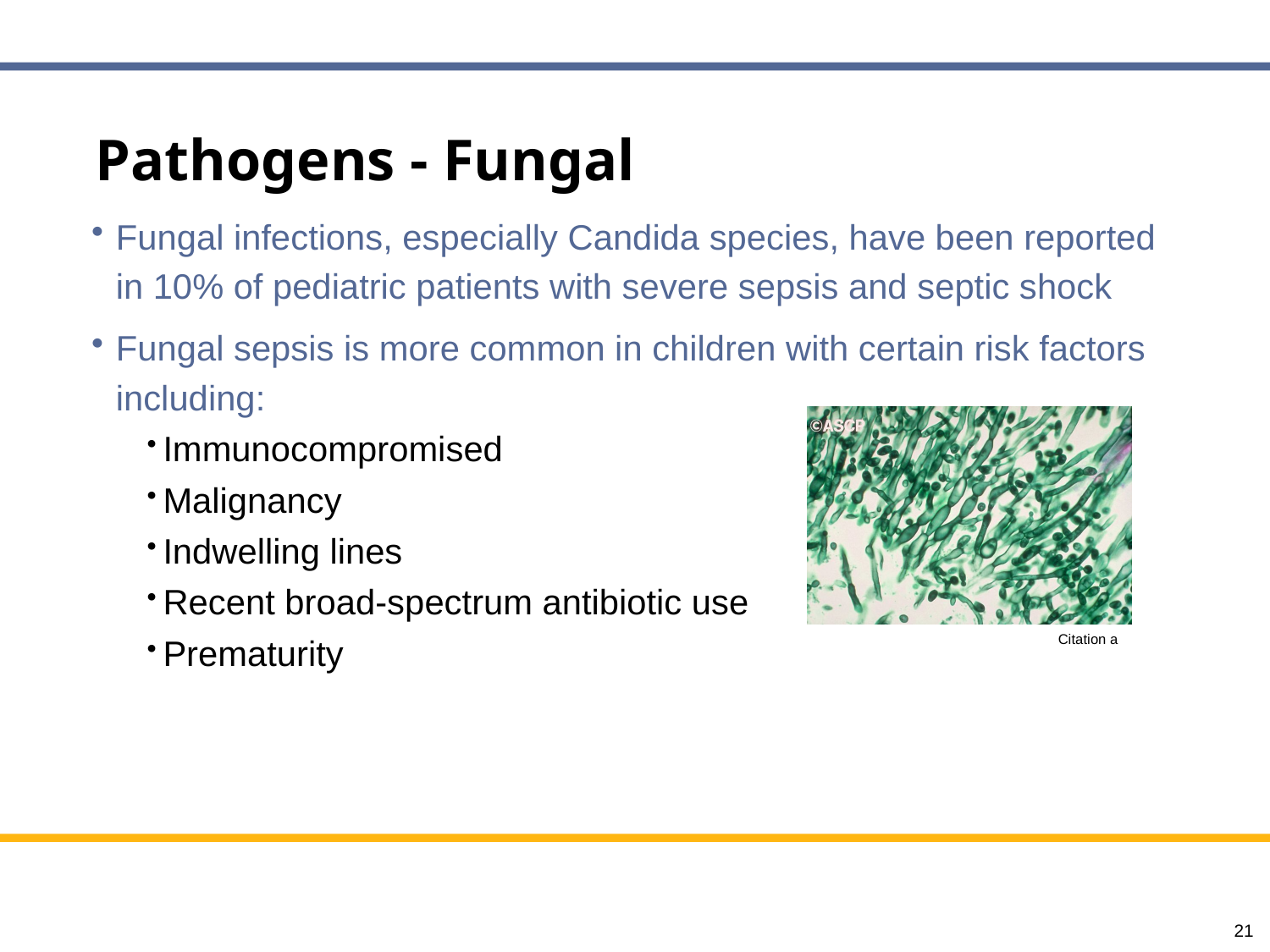

# Pathogens - Fungal
Fungal infections, especially Candida species, have been reported in 10% of pediatric patients with severe sepsis and septic shock
Fungal sepsis is more common in children with certain risk factors including:
Immunocompromised
Malignancy
Indwelling lines
Recent broad-spectrum antibiotic use
Prematurity
Citation a
21

## Slide 22
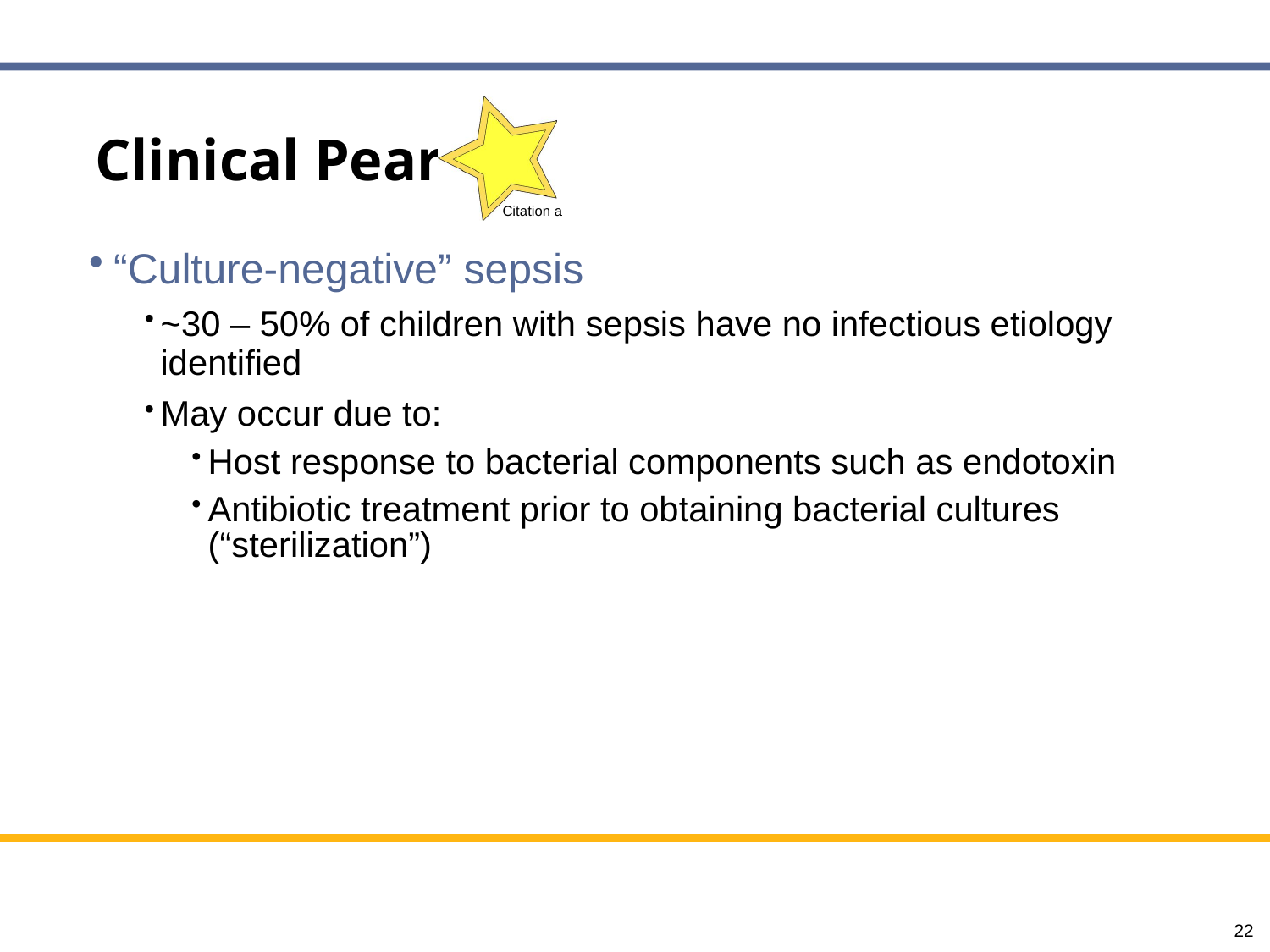

# Clinical Pearl
Citation a
“Culture-negative” sepsis
~30 – 50% of children with sepsis have no infectious etiology identified
May occur due to:
Host response to bacterial components such as endotoxin
Antibiotic treatment prior to obtaining bacterial cultures (“sterilization”)
22

## Slide 23
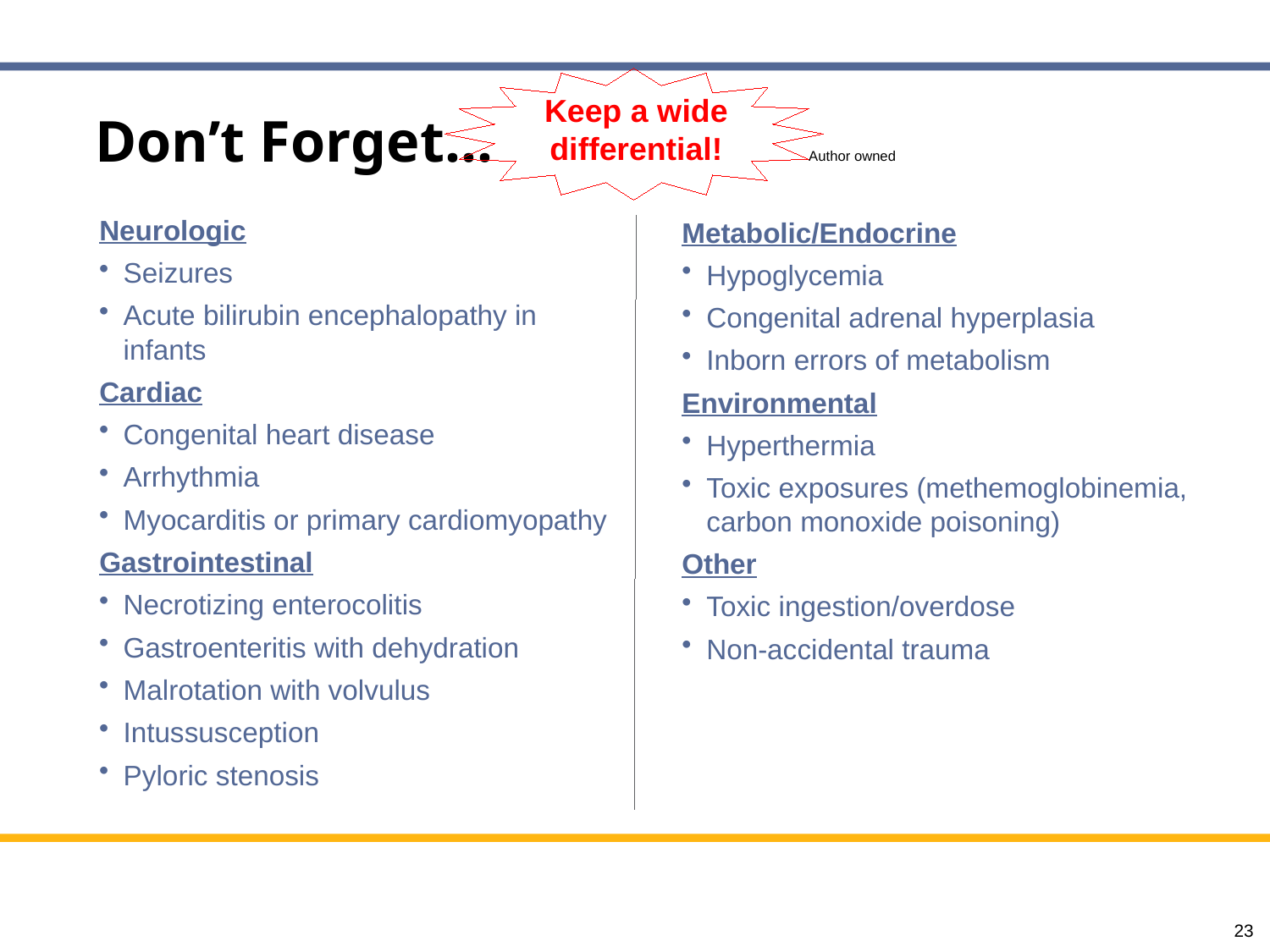

# Don’t Forget…
Keep a wide differential!
Author owned
Neurologic
Seizures
Acute bilirubin encephalopathy in infants
Cardiac
Congenital heart disease
Arrhythmia
Myocarditis or primary cardiomyopathy
Gastrointestinal
Necrotizing enterocolitis
Gastroenteritis with dehydration
Malrotation with volvulus
Intussusception
Pyloric stenosis
Metabolic/Endocrine
Hypoglycemia
Congenital adrenal hyperplasia
Inborn errors of metabolism
Environmental
Hyperthermia
Toxic exposures (methemoglobinemia, carbon monoxide poisoning)
Other
Toxic ingestion/overdose
Non-accidental trauma
23

## Slide 24
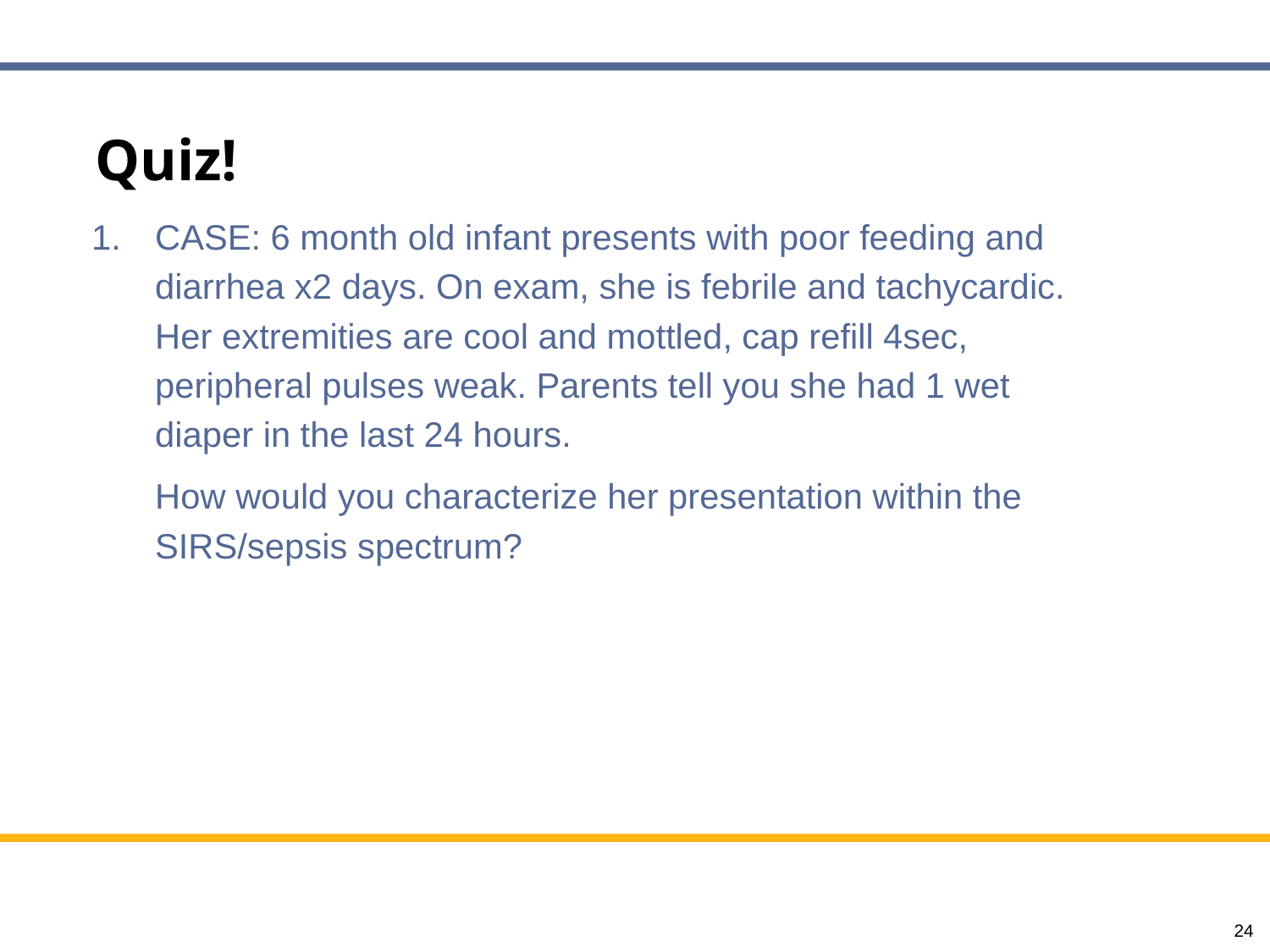

# Quiz!
CASE: 6 month old infant presents with poor feeding and diarrhea x2 days. On exam, she is febrile and tachycardic. Her extremities are cool and mottled, cap refill 4sec, peripheral pulses weak. Parents tell you she had 1 wet diaper in the last 24 hours.
	How would you characterize her presentation within the SIRS/sepsis spectrum?
24

## Slide 25
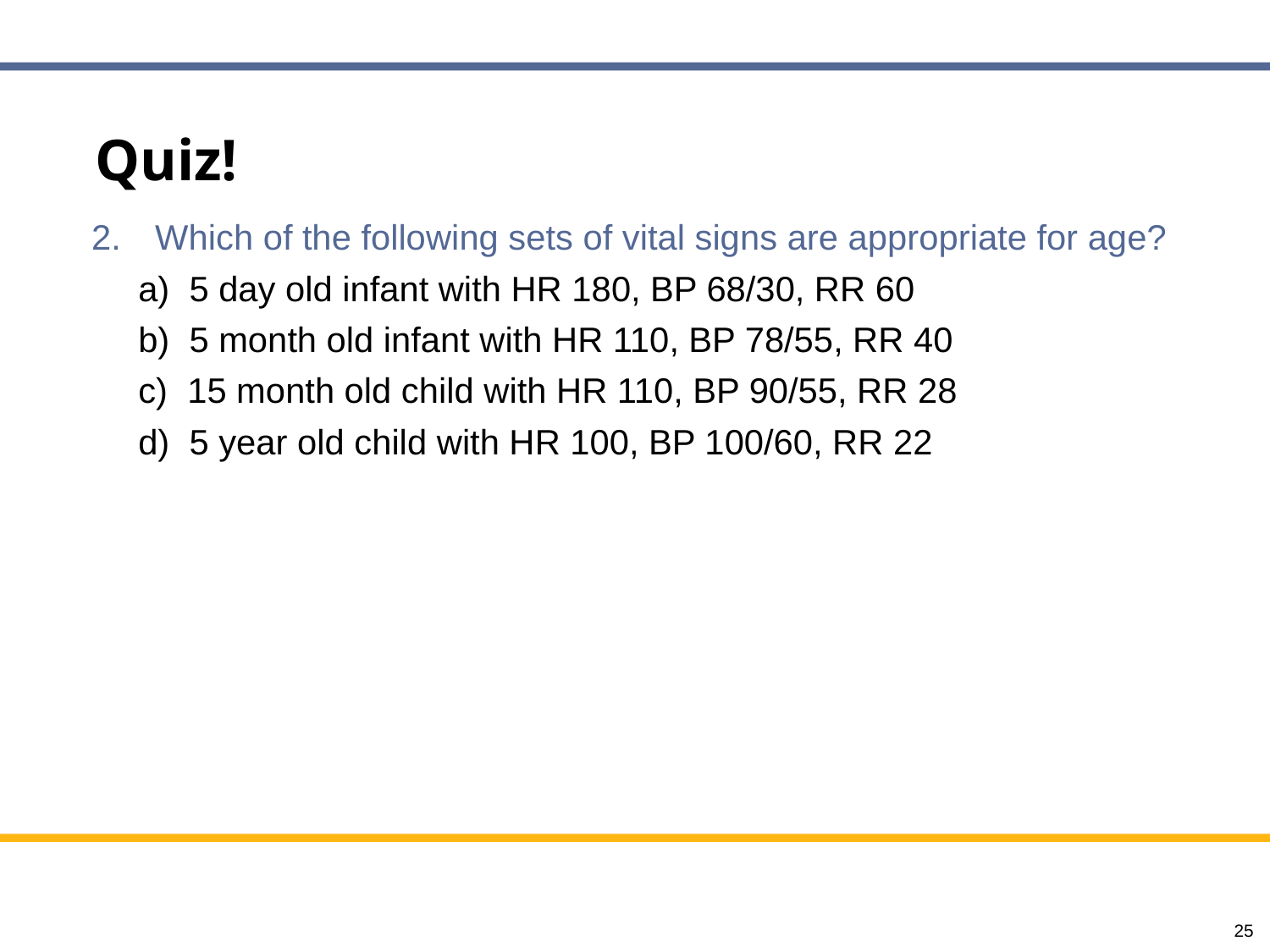

# Quiz!
Which of the following sets of vital signs are appropriate for age?
a) 5 day old infant with HR 180, BP 68/30, RR 60
b) 5 month old infant with HR 110, BP 78/55, RR 40
c) 15 month old child with HR 110, BP 90/55, RR 28
d) 5 year old child with HR 100, BP 100/60, RR 22
25

## Slide 26
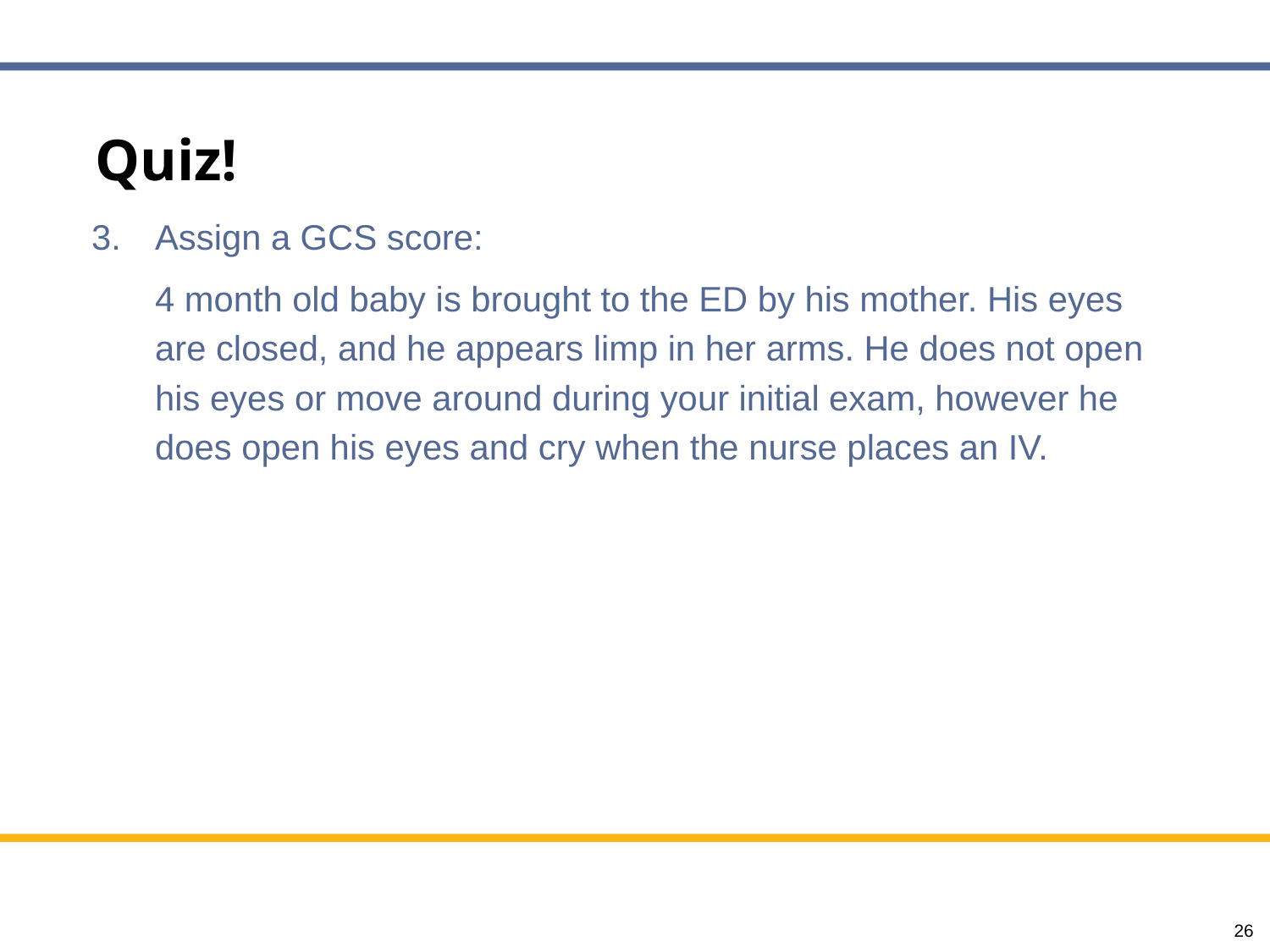

# Quiz!
Assign a GCS score:
	4 month old baby is brought to the ED by his mother. His eyes are closed, and he appears limp in her arms. He does not open his eyes or move around during your initial exam, however he does open his eyes and cry when the nurse places an IV.
26
