# Supplementary material for: Identification and Management of Pediatric Sepsis: A Medical Student Curricular Supplement for PICU and NICU Rotations
Source: MedEdPORTAL. 2021 Apr 23;17:11142. doi: 10.15766/mep_2374-8265.11142 (PMC8063627; doi:10.15766/mep_2374-8265.11142)
Supplement: Supplementary file 1 — Pre- & Posttest.docxModule 1 - Pediatric Shock.pptxScript 1 - Pediatric Shock.docxModule 2 - Pediatric Sepsis.pptxScript 2 - Pediatric Sepsis.docxModule 3 - Management of Sepsis & Septic Shock.pptxScript 3 - Management of Sepsis & Septic Shock. docxModule 4 - Hemodynamics & Pressor Support.pptxScript 4 - Hemodynamics & Pressor Support.docxSimulation Case 1.docxSimulation Case 2.docxSimulation Case 3.docxPostsimulation Review Quiz.pptx [file mep_2374-8265.11142-s001.zip › E. Script 2 - Pediatric Sepsis.docx]

**Pediatric Sepsis**

1. Welcome to the Pediatric Sepsis online curriculum. Our topic here is an introduction to pediatric sepsis.
2. The learning objectives will be to:
   - Recognize the spectrum of sepsis presentations
   - Identify the common signs and symptoms associated with sepsis
   - Identify common pathogens for pediatric sepsis
3. Pediatric sepsis has a significant impact on the pediatric patient population. There are almost 48,000 cases of pediatric sepsis per year with a mortality rate of 9%. The average length of stay is about 1 month and costs upwards of $40,000 per admission, which leads to an estimated national cost of 2.3 billion dollars to our healthcare system.
4. While recognizing that this diagnosis carries a significant burden of disease, it is important to understand the different distinctions of sepsis presentations. This graph highlights the progression of worsening sepsis.
5. Initially, there is SIRS which stands for systemic inflammatory response syndrome. SIRS can then progress to sepsis, severe sepsis, and ultimately septic shock. Let’s go through each stage in a little more detail.
6. SIRS is characterized as a widespread inflammatory response that may or may not be associated with infection. This inflammatory response requires 2 or more of the following:
   - Core temperature <36°C or >38°C
   - Leukocyte count elevated or depressed for age, or >10% bands
   - Tachycardia or bradycardia
   - Tachypnea
7. Sepsis is the presence of SIRS plus a suspected or proven source of infection. Sources can include bacteremia with a positive blood culture, meningitis based on the results of CSF studies, pneumonia as shown on this CXR, urinary tract infection from a sterile culture, and/or the presence of skin or soft tissue infection.
8. The 2005 Sepsis guidelines defined “severe sepsis” as greater than or equal to 2 SIRS criteria, with suspected or confirmed invasive infection, as well as cardiac dysfunction, severe ARDS, or at least 2 non-cardiovascular system dysfunctions. New guidelines have broadened this definition to include severe infection that leads to life threatening organ dysfunction. Of note, the definition of pediatric sepsis was not updated during the 2020 Sepsis Guidelines and remains under revision from the initial 2005 guidelines. Signs of end-organ dysfunction can be thought of in terms of systems. A neurological sign may be altered mental status. Respiratory signs include acute respiratory distress syndrome. Cardiovascular signs include systolic or diastolic heart failure. Hepatic dysfunction can be demonstrated with abnormal laboratory values – specifically for pediatrics these include a total bilirubin >4 or AST greater than 2x upper limit of normal. There can also be evidence of liver synthetic dysfunction with an INR >2. Signs of renal dysfunction include oliguria or anuria or a doubling of the serum creatinine. Hematologic dysfunction includes thrombocytopenia with a platelet count less than 80,000 and DIC.
9. Septic shock is the most ominous of sepsis presentations as it is associated with severe cardiovascular dysfunction that is refractory to fluid resuscitation. The 2020 Sepsis Guidelines also include “sepsis-associated organ dysfunction” with septic shock which can be associated with both cardiovascular and/or non-cardiovascular dysfunction.
10. Pearl - Children, unlike adults, can be in a state of shock without hypotension. Signs of poor perfusion are consistent with shock. Children maintain their BP by increasing heart rate and SVR.
11. So, what are some of the signs and symptoms of septic shock?

- Fever is the most common presenting symptom
- Tachycardia
- Tachypnea or increased work of breathing. This picture shows an infant with intercostal retractions. Infants may also grunt or have nasal flaring when in respiratory distress.
- Cool or mottled extremities as shown here
- Other color changes such as cyanosis, pallor, or ashen
- Decreased tone, which also leads to…
- Poor feeding in the infant and…
- Lethargy
- Altered mental status
- You may also see signs of meningeal irritation on exam of an older child, although meningeal signs are *not* a reliable indicator of meningitis in neonates and infants.

1. To recognize the signs and symptoms of SIRS and sepsis, it is important to recognize the age-appropriate normative values for vital signs. This chart provides the average heart rate, blood pressure, and respiratory rates seen in various pediatric age groups.
2. Similarly, this chart provides an explanation for signs that should be evaluated with using the Pediatric Glasgow Coma Scale for infants and non-verbal patients.
3. A child comes in with their eyes closed. When you tell him to open his eyes, he does not. When you press against his nail with the back of your reflex hammer, he opens his eyes and moans and withdraws his hand. He does not respond to questions and does not speak. What is his GCS Score?
4. Let’s add up our score. His eyes open to pain (2), he moans to pain (2) and he withdraws to pain (4). That gives us a GSC score of 8.
5. *Click fast for animation.* Taking a history in the ICU will help you to assess what stage a patient is presenting in. There are several important historical questions to consider. These include activity level (for example, was the child playing, sleeping, more fussy than usual?), mental status relative to age, urine output (for infants it may be helpful to ask parents about a change in number of wet diapers). Be sure to ask about immunization status and whether the patient is up to date as you may encounter some parents who have opted for customized or delayed vaccination schedules. Remember to inquire about any allergies, especially to medications. And lastly, a critical piece of information is determining if there are any sick contacts, recent travel, or other possible sources of infection.
6. Now who is at risk for sepsis? Risk factors include:
   - Infants <1 month of age
   - Immunodeficiency syndromes
   - Malignancy
   - Congenital heart disease
   - Sickle cell disease
   - Splenic dysfunction or absence
   - Indwelling catheters or prosthetics
   - Major trauma or penetrating wounds
   - Significant burns
   - Urinary tract abnormalities with frequent infection
7. There are a variety of pathogens that frequently cause sepsis. These include bacterial, viral, and fungal pathogens. Common gram-positive organisms are listed here, and include staph species (including MRSA), strep pneumo, group A strep, group B strep, enterococcus (including VRE), and listeria. Staph bacteria will often be described as gram positive cocci in clusters and Strep as gram positive cocci in chains as shown in the images here. Group B strep is an important cause of infection in the neonate. Gram positive rods causing sepsis include Listeria, particularly in the newborn patient population.
8. Gram negative sepsis may be due to Neisseria species, which are gram negative cocci. Or, more commonly, due to gram negative rods such as E. coli, pseudomonas, klebsiella, serratia, and salmonella.
9. Common viral pathogens that may cause a severe sepsis picture include:
   - Influenza
   - Parainfluenza
   - Respiratory Syncytial Virus (RSV)
   - Adenovirus
   - Herpes Simplex Virus (HSV)
   - Human metapneumovirus
   - EBV/CMV in immunocompromised patients
10. Lastly fungal infections, especially Candida species, have been reported in 10% of patients with septic shock. A fungal source will be more common in children with certain risk factors including:
    - Immunocompromised
    - Malignancy
    - Indwelling lines
    - Recent broad-spectrum antibiotic use
    - Prematurity
11. PEARL – Here is another pediatric pearl! There is a specific term known as culture negative sepsis that describes approximately 30-50% of pediatric patients with no identifiable infectious etiology in the presence of sepsis. This may occur due to a host response to a bacterial endotoxin or due to treatment with antibiotics prior to obtaining cultures.
12. We’ve now spent a considerable amount of time discussing sepsis risk factors, signs, symptoms, and causes. However, there are many other conditions that may present with a very similar clinical picture. Although this is a busy slide, it is included here to remind you of how extensive your differential diagnosis should be. This list is also categorized by system.
13. We finish with a short quiz! There are 3 questions here for you to answer in advance, and they will be discussed at your simulation session.
