# Supplementary material for: Identification and Management of Pediatric Sepsis: A Medical Student Curricular Supplement for PICU and NICU Rotations
Source: MedEdPORTAL. 2021 Apr 23;17:11142. doi: 10.15766/mep_2374-8265.11142 (PMC8063627; doi:10.15766/mep_2374-8265.11142)
Supplement: Supplementary file 1 — Pre- & Posttest.docxModule 1 - Pediatric Shock.pptxScript 1 - Pediatric Shock.docxModule 2 - Pediatric Sepsis.pptxScript 2 - Pediatric Sepsis.docxModule 3 - Management of Sepsis & Septic Shock.pptxScript 3 - Management of Sepsis & Septic Shock. docxModule 4 - Hemodynamics & Pressor Support.pptxScript 4 - Hemodynamics & Pressor Support.docxSimulation Case 1.docxSimulation Case 2.docxSimulation Case 3.docxPostsimulation Review Quiz.pptx [file mep_2374-8265.11142-s001.zip › F. Module 3 - Management of Sepsis & Septic Shock.pptx]

## Slide 1
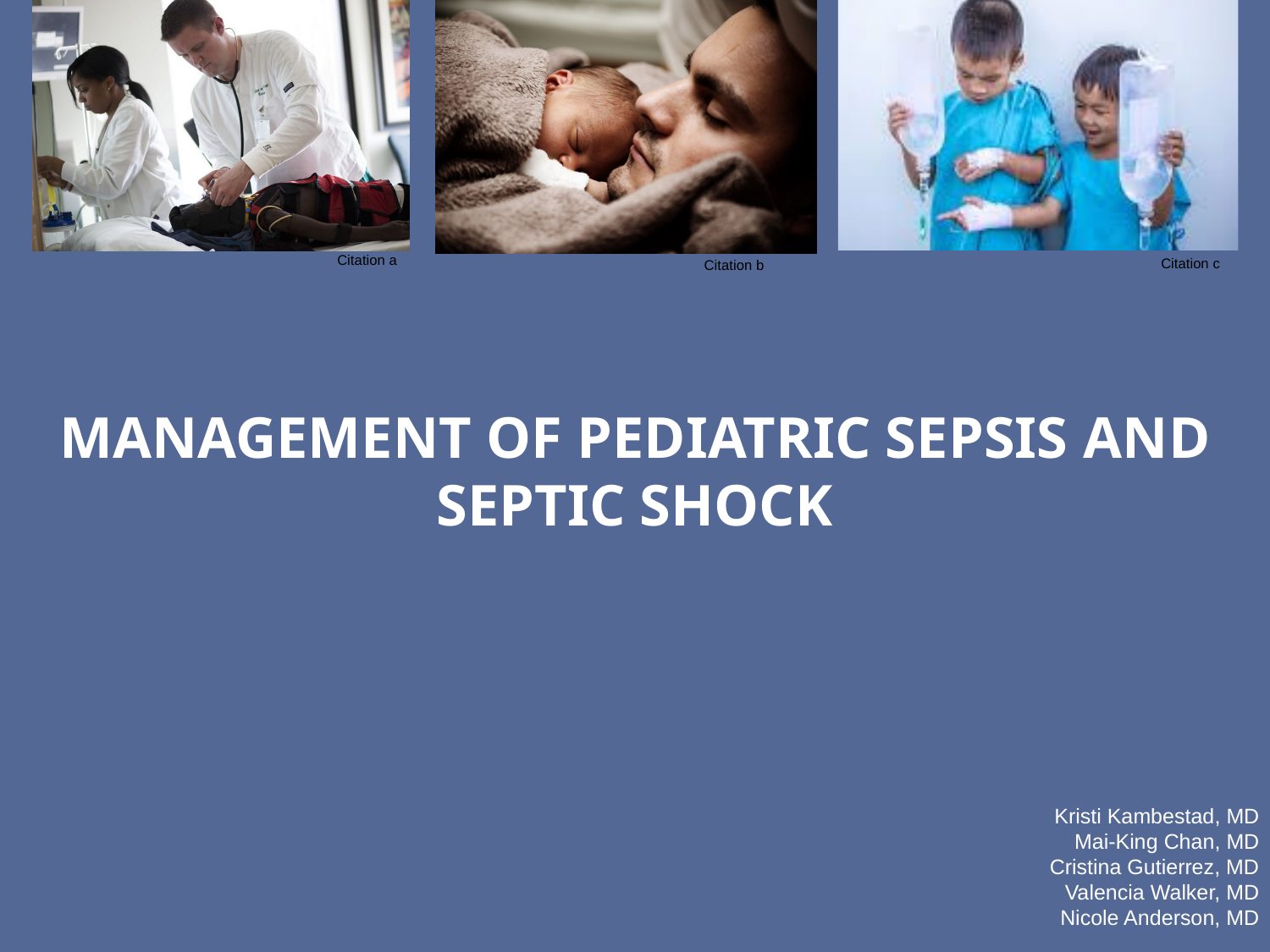

Citation a
Citation c
Citation b
MANAGEMENT OF PEDIATRIC SEPSIS AND SEPTIC SHOCK
Kristi Kambestad, MD
Mai-King Chan, MD
Cristina Gutierrez, MD
Valencia Walker, MD
Nicole Anderson, MD

## Slide 2
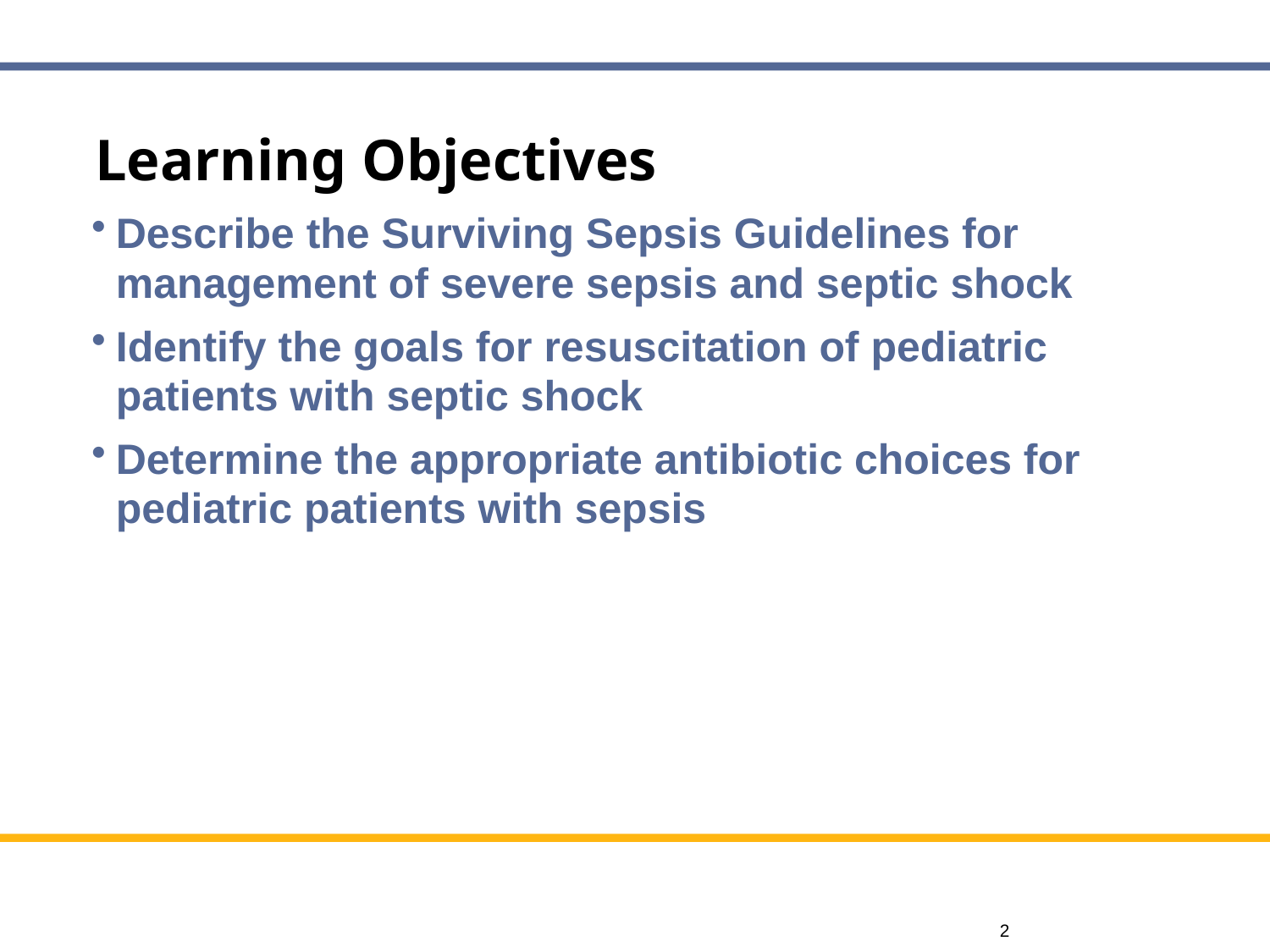

Learning Objectives
Describe the Surviving Sepsis Guidelines for management of severe sepsis and septic shock
Identify the goals for resuscitation of pediatric patients with septic shock
Determine the appropriate antibiotic choices for pediatric patients with sepsis
2

## Slide 3
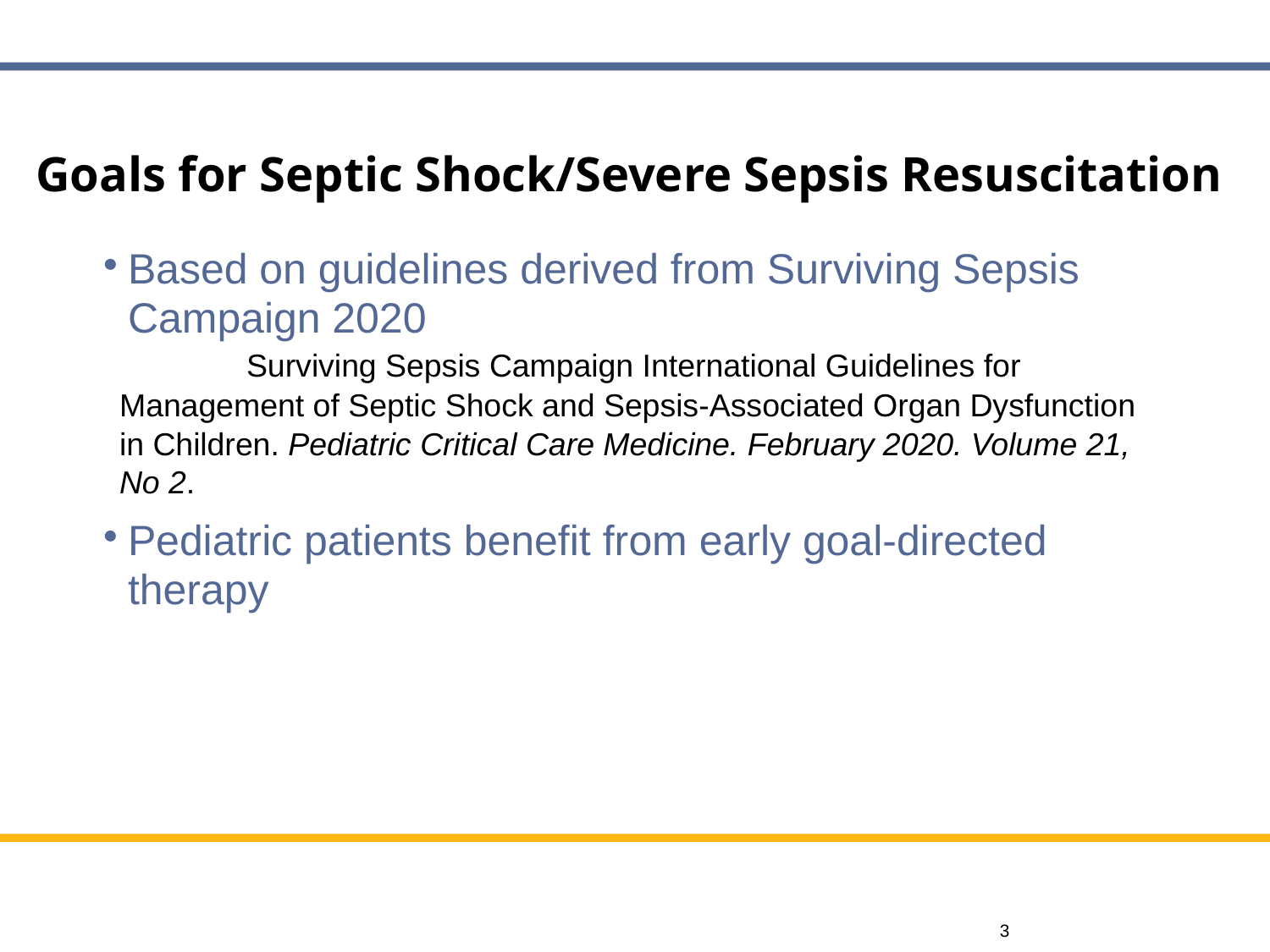

Goals for Septic Shock/Severe Sepsis Resuscitation
Based on guidelines derived from Surviving Sepsis Campaign 2020
	Surviving Sepsis Campaign International Guidelines for Management of Septic Shock and Sepsis-Associated Organ Dysfunction in Children. Pediatric Critical Care Medicine. February 2020. Volume 21, No 2.
Pediatric patients benefit from early goal-directed therapy
3

## Slide 4
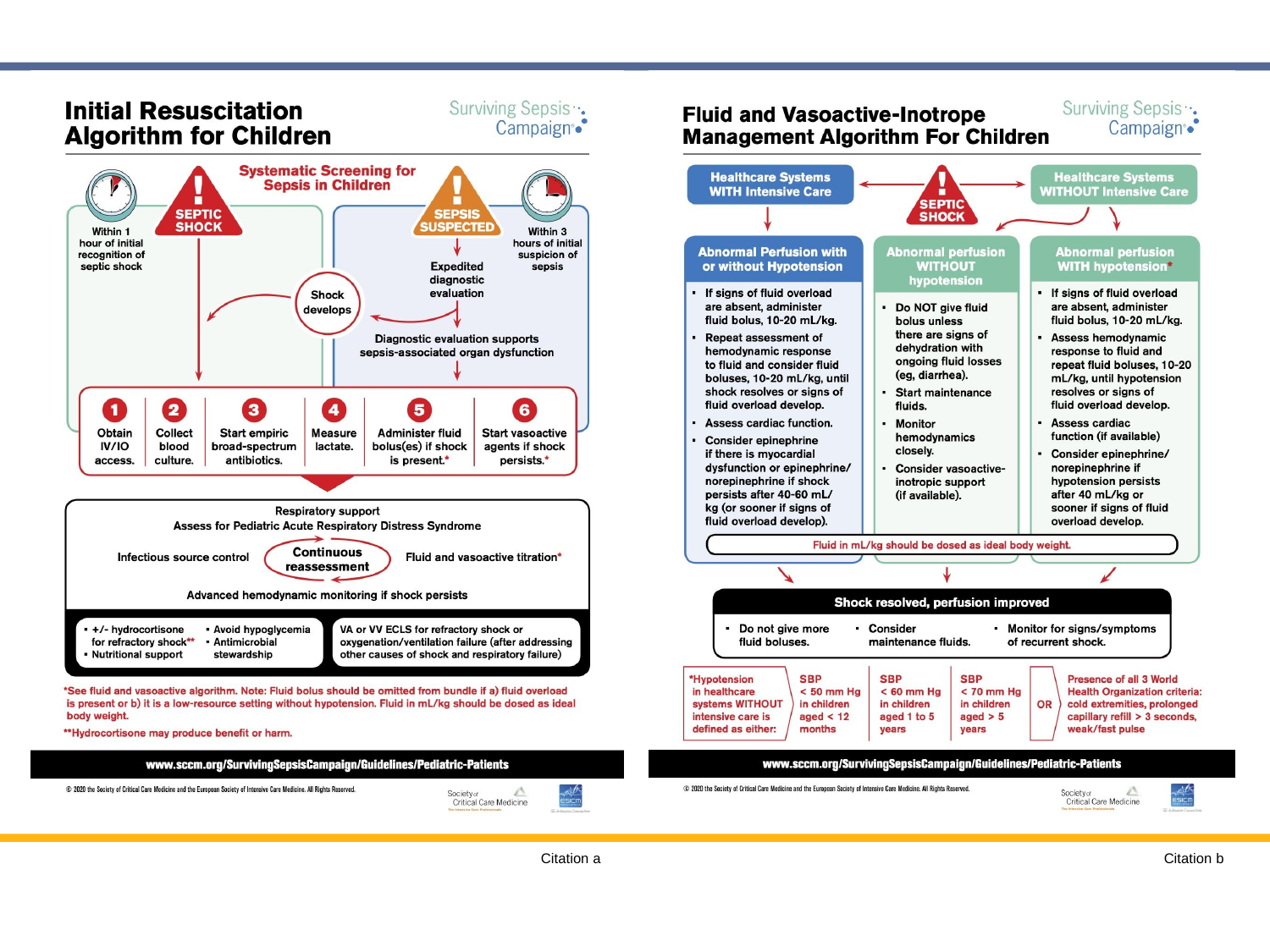

Citation a
Citation b

## Slide 5
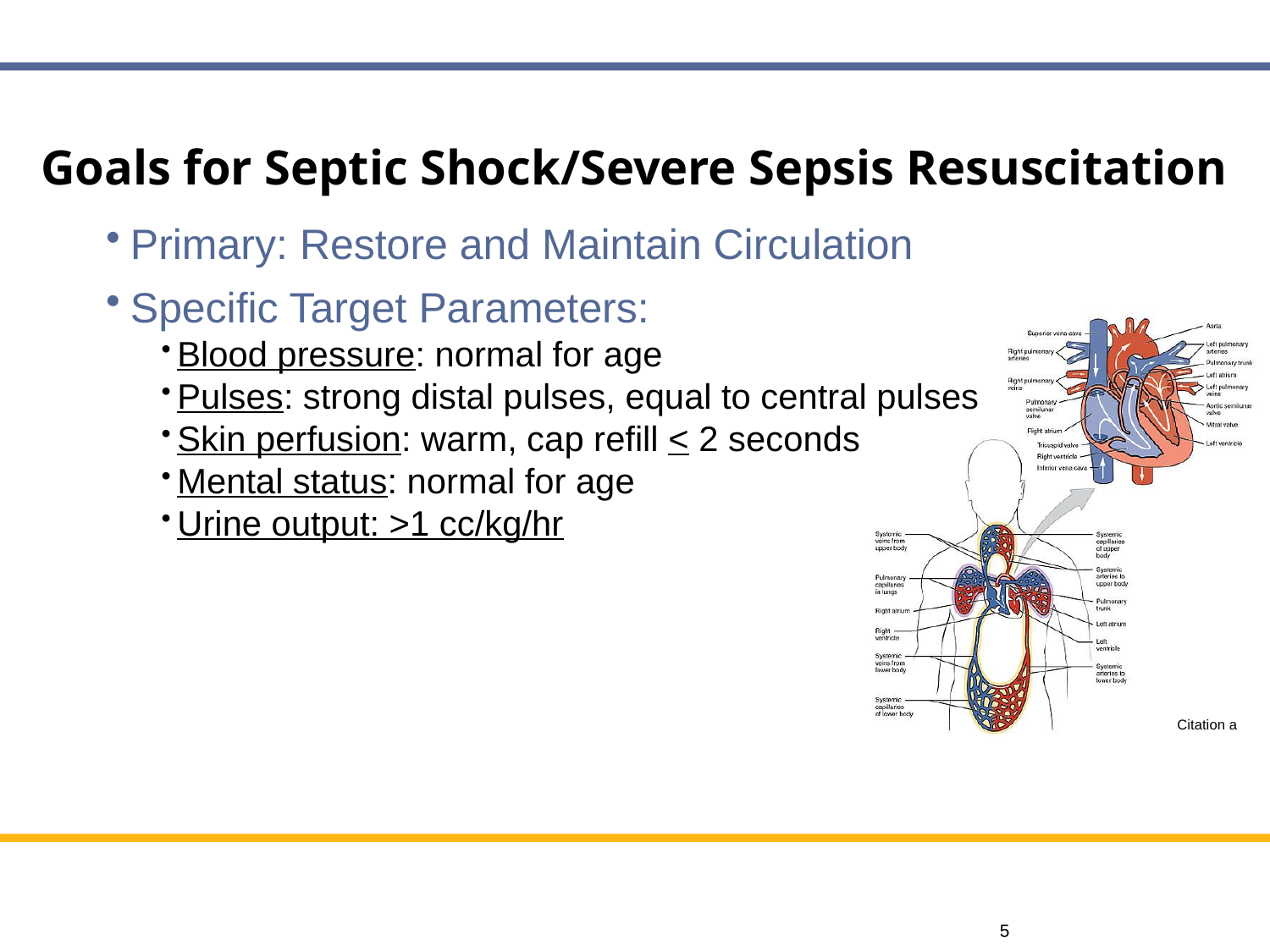

Goals for Septic Shock/Severe Sepsis Resuscitation
Primary: Restore and Maintain Circulation
Specific Target Parameters:
Blood pressure: normal for age
Pulses: strong distal pulses, equal to central pulses
Skin perfusion: warm, cap refill < 2 seconds
Mental status: normal for age
Urine output: >1 cc/kg/hr
Citation a
5

## Slide 6
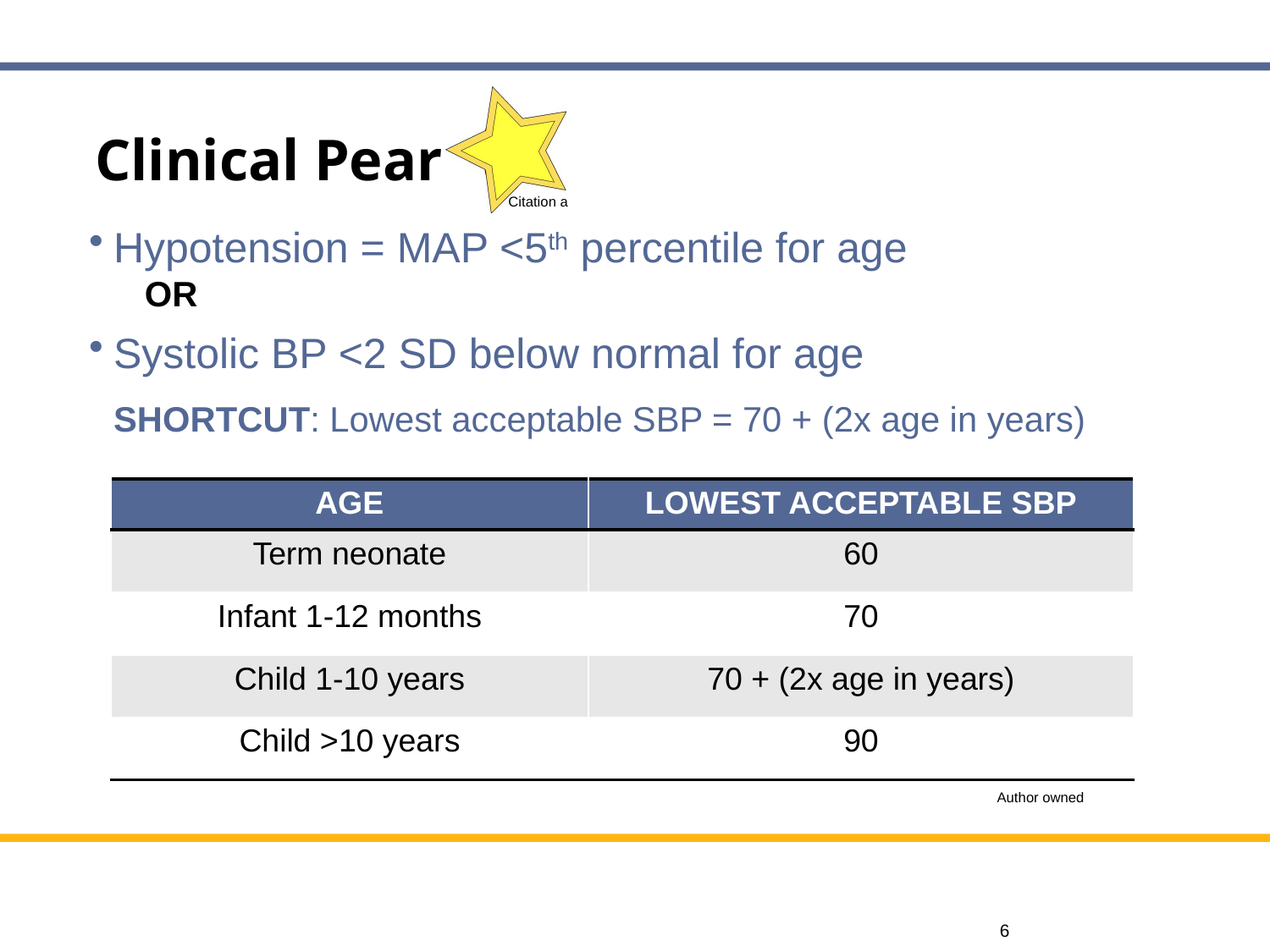

Clinical Pearl
Citation a
Hypotension = MAP <5th percentile for age
OR
Systolic BP <2 SD below normal for age
	SHORTCUT: Lowest acceptable SBP = 70 + (2x age in years)
| AGE | LOWEST ACCEPTABLE SBP |
| --- | --- |
| Term neonate | 60 |
| Infant 1-12 months | 70 |
| Child 1-10 years | 70 + (2x age in years) |
| Child >10 years | 90 |
Author owned
6

## Slide 7
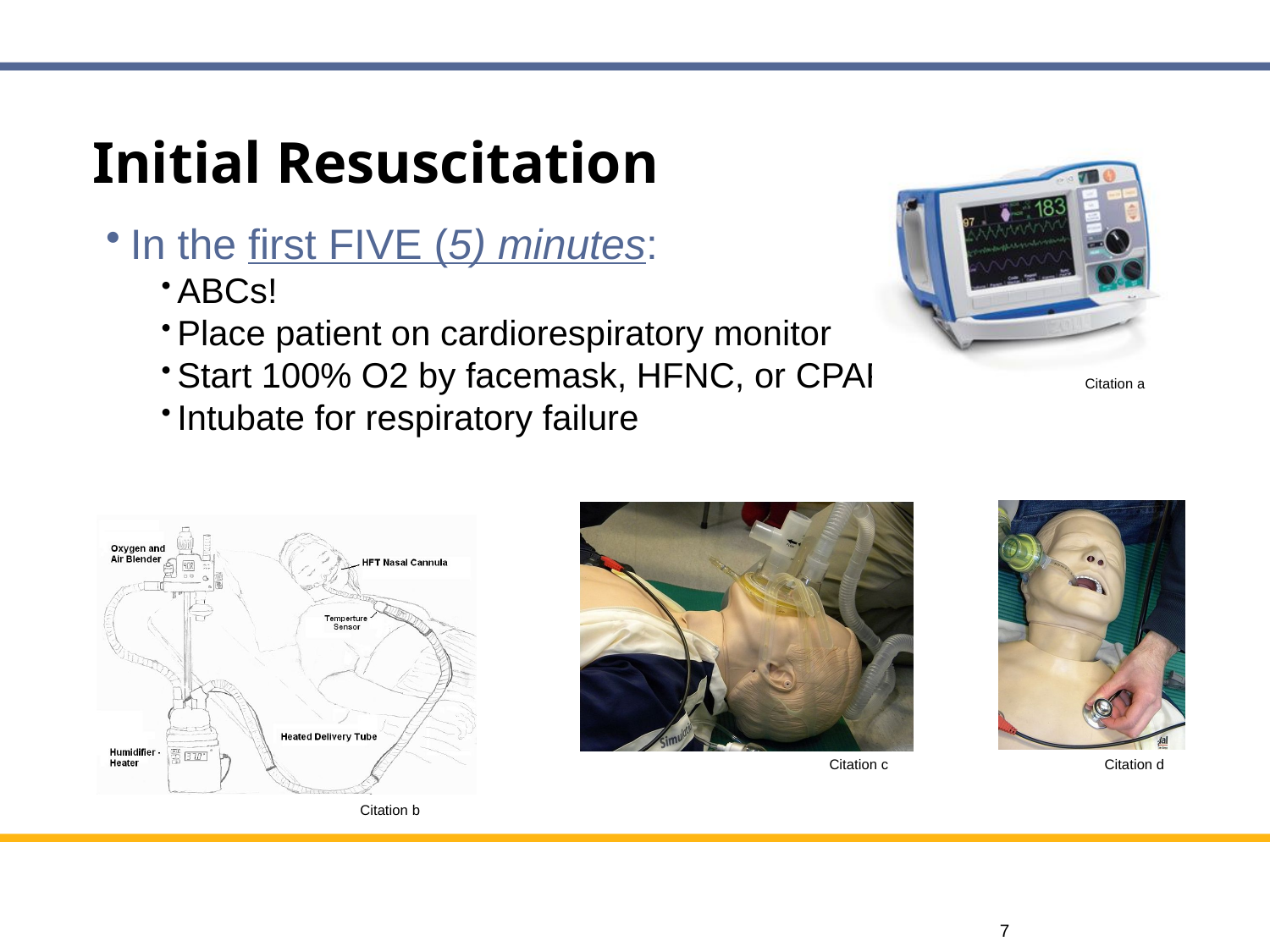

Initial Resuscitation
In the first FIVE (5) minutes:
ABCs!
Place patient on cardiorespiratory monitor
Start 100% O2 by facemask, HFNC, or CPAP
Intubate for respiratory failure
Citation a
Citation c
Citation d
Citation b
7

## Slide 8
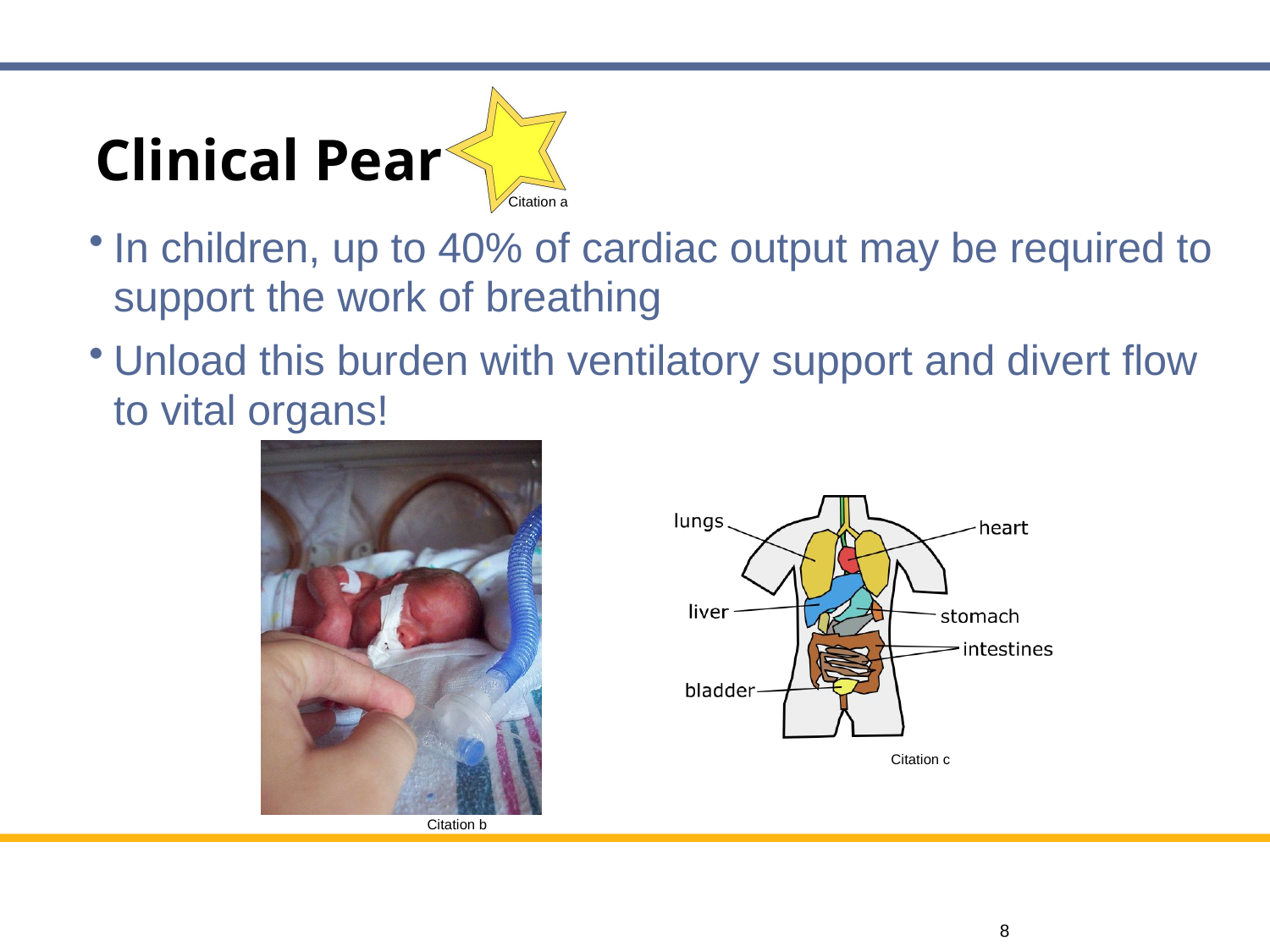

Clinical Pearl
Citation a
In children, up to 40% of cardiac output may be required to support the work of breathing
Unload this burden with ventilatory support and divert flow to vital organs!
Citation c
Citation b
8

## Slide 9
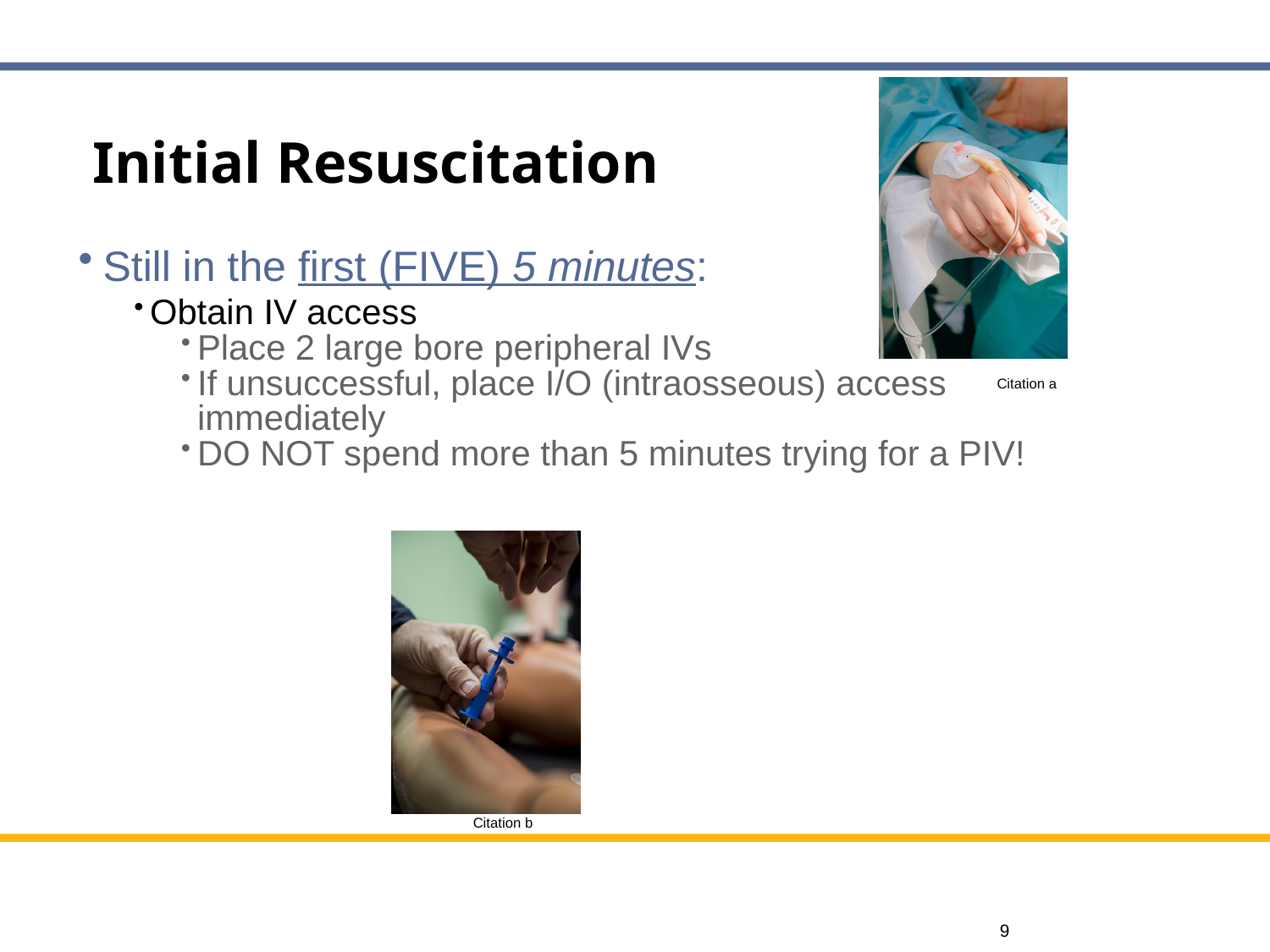

Initial Resuscitation
Still in the first (FIVE) 5 minutes:
Obtain IV access
Place 2 large bore peripheral IVs
If unsuccessful, place I/O (intraosseous) access immediately
DO NOT spend more than 5 minutes trying for a PIV!
Citation a
Citation b
9

## Slide 10
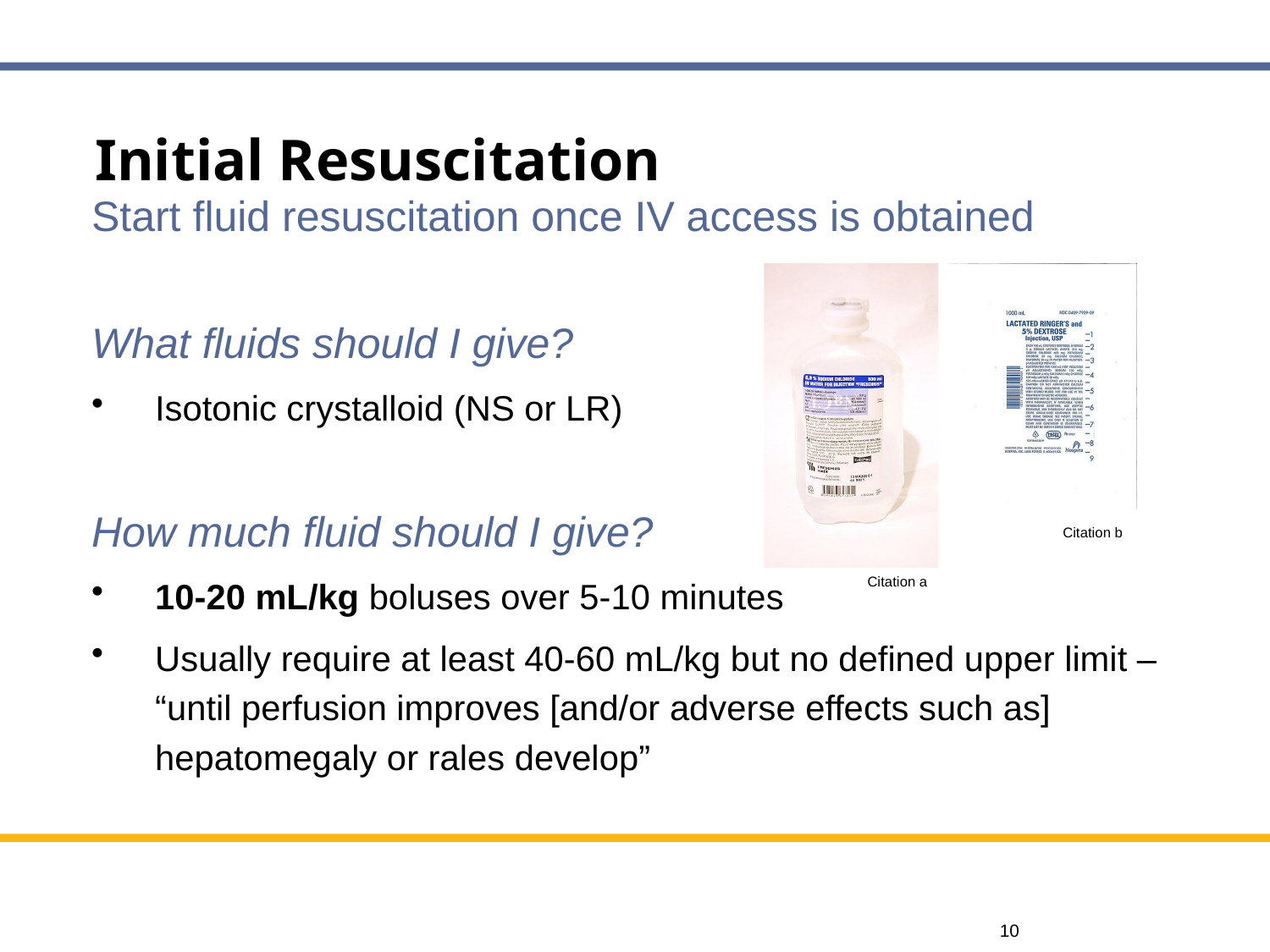

Initial Resuscitation
Start fluid resuscitation once IV access is obtained
What fluids should I give?
Isotonic crystalloid (NS or LR)
How much fluid should I give?
10-20 mL/kg boluses over 5-10 minutes
Usually require at least 40-60 mL/kg but no defined upper limit – “until perfusion improves [and/or adverse effects such as] hepatomegaly or rales develop”
Citation b
Citation a
10

## Slide 11
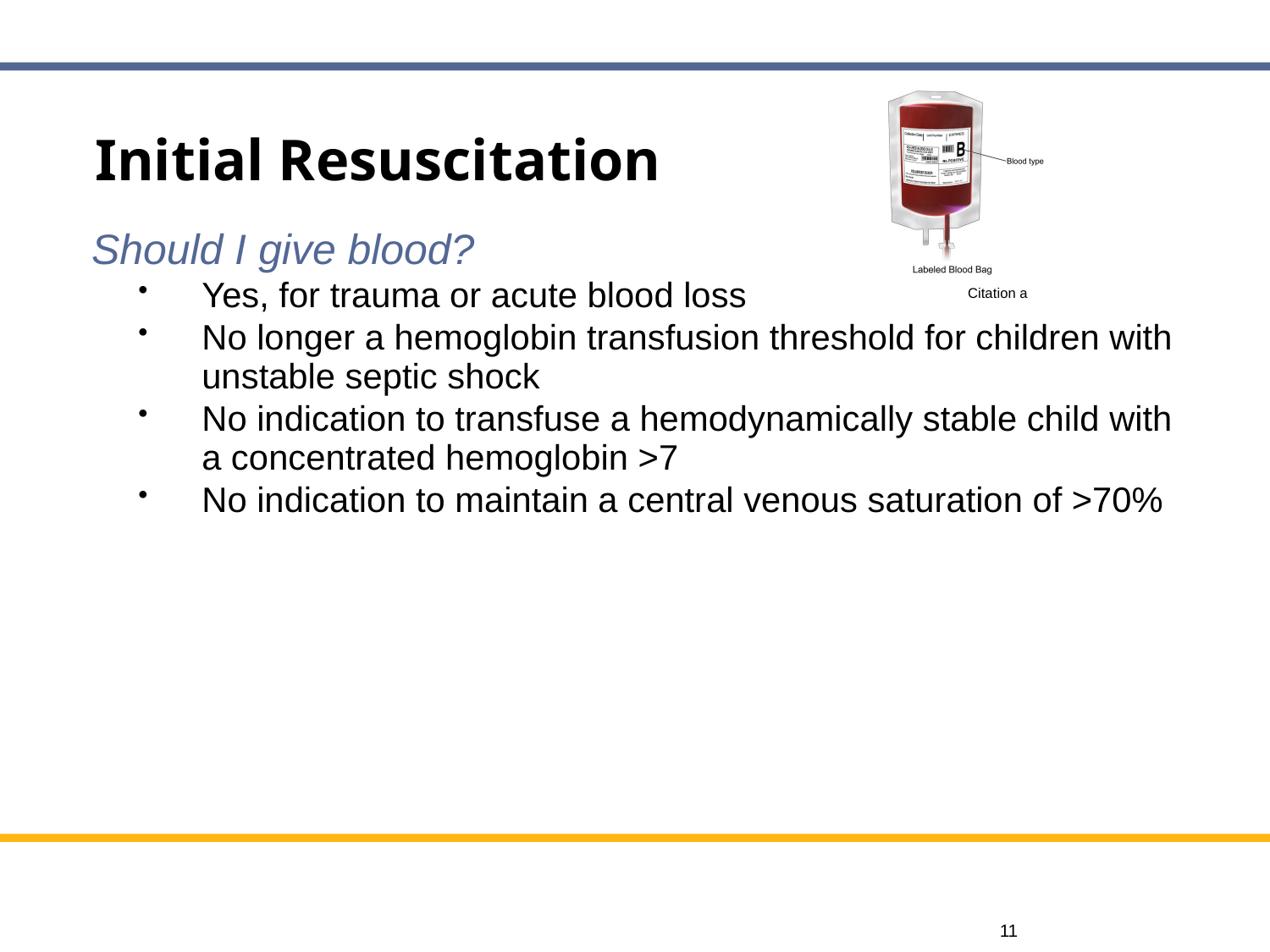

Initial Resuscitation
Should I give blood?
Yes, for trauma or acute blood loss
No longer a hemoglobin transfusion threshold for children with unstable septic shock
No indication to transfuse a hemodynamically stable child with a concentrated hemoglobin >7
No indication to maintain a central venous saturation of >70%
Citation a
11

## Slide 12
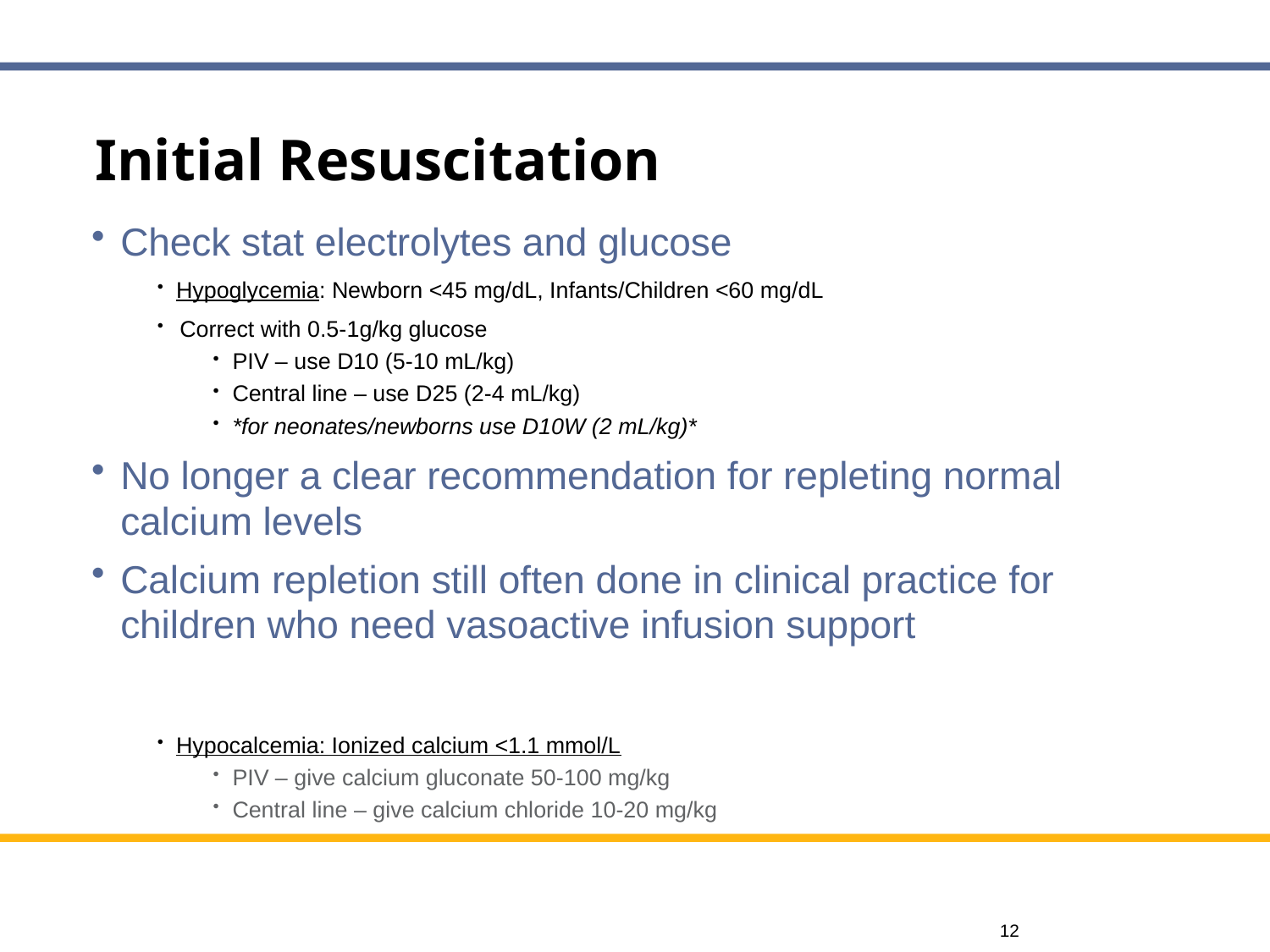

Initial Resuscitation
Check stat electrolytes and glucose
Hypoglycemia: Newborn <45 mg/dL, Infants/Children <60 mg/dL
Correct with 0.5-1g/kg glucose
PIV – use D10 (5-10 mL/kg)
Central line – use D25 (2-4 mL/kg)
*for neonates/newborns use D10W (2 mL/kg)*
No longer a clear recommendation for repleting normal calcium levels
Calcium repletion still often done in clinical practice for children who need vasoactive infusion support
Hypocalcemia: Ionized calcium <1.1 mmol/L
PIV – give calcium gluconate 50-100 mg/kg
Central line – give calcium chloride 10-20 mg/kg
12

## Slide 13
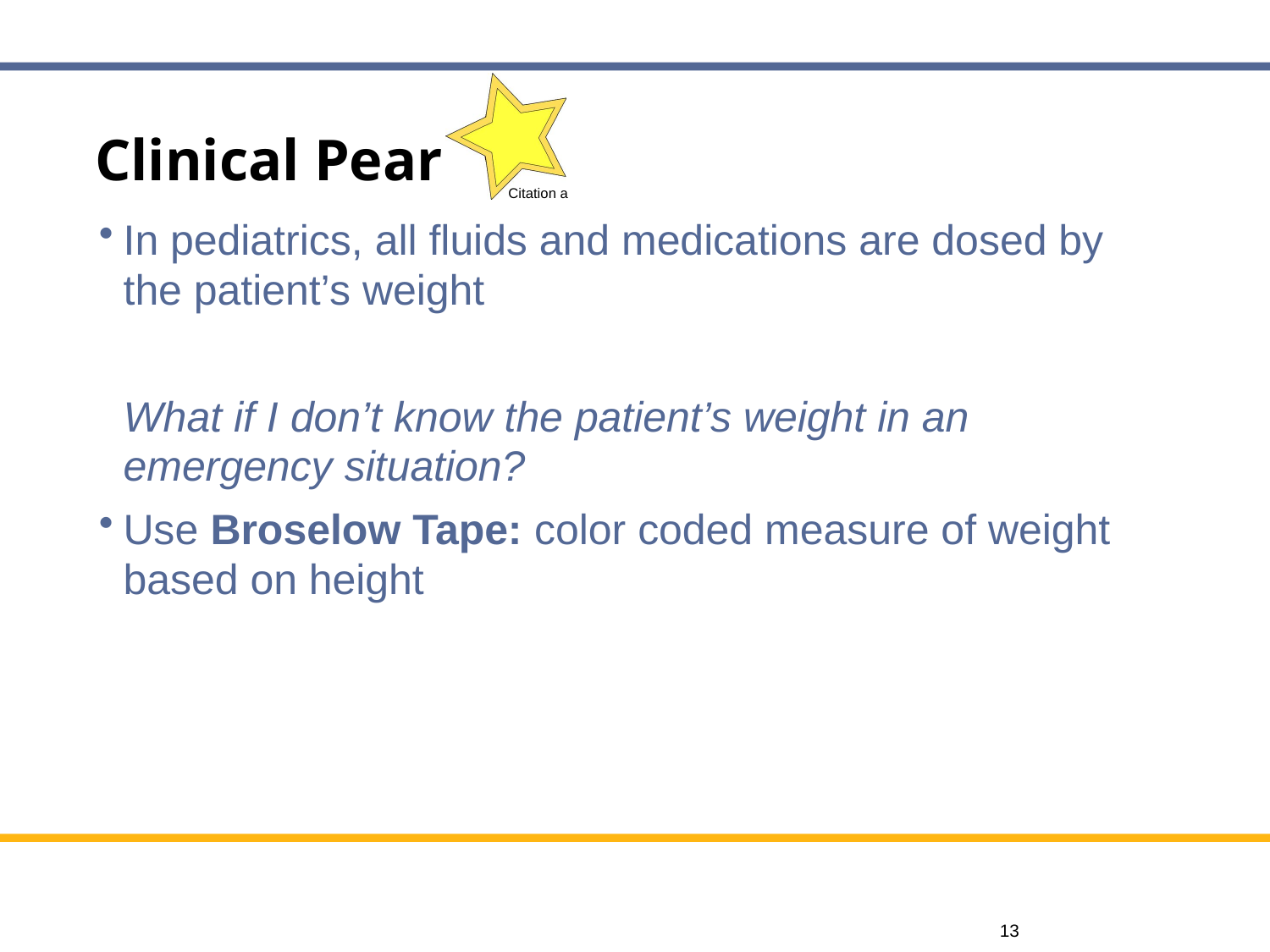

Clinical Pearl
Citation a
In pediatrics, all fluids and medications are dosed by the patient’s weight
	What if I don’t know the patient’s weight in an emergency situation?
Use Broselow Tape: color coded measure of weight based on height
13

## Slide 14
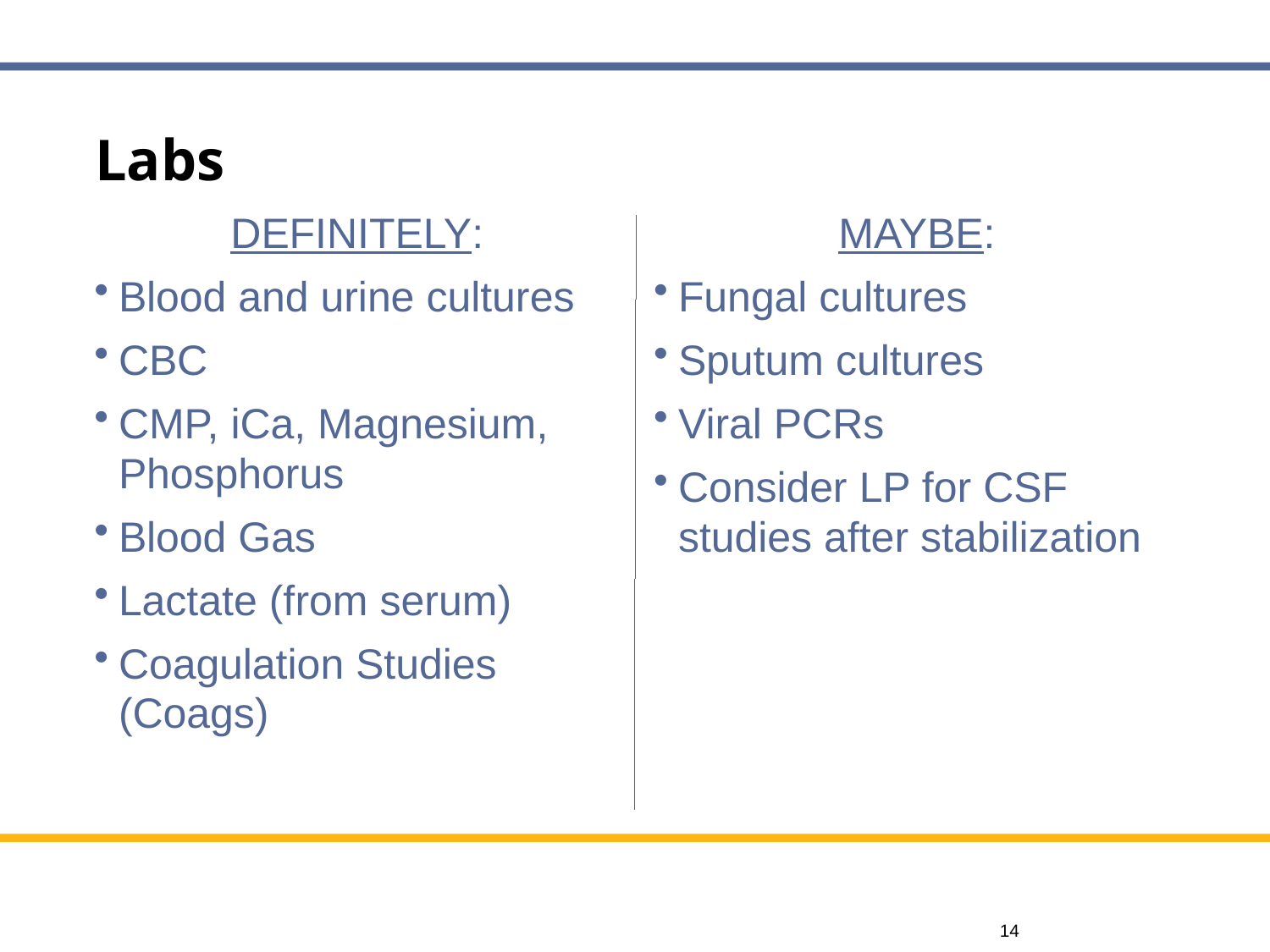

Labs
DEFINITELY:
Blood and urine cultures
CBC
CMP, iCa, Magnesium, Phosphorus
Blood Gas
Lactate (from serum)
Coagulation Studies (Coags)
MAYBE:
Fungal cultures
Sputum cultures
Viral PCRs
Consider LP for CSF studies after stabilization
14

## Slide 15
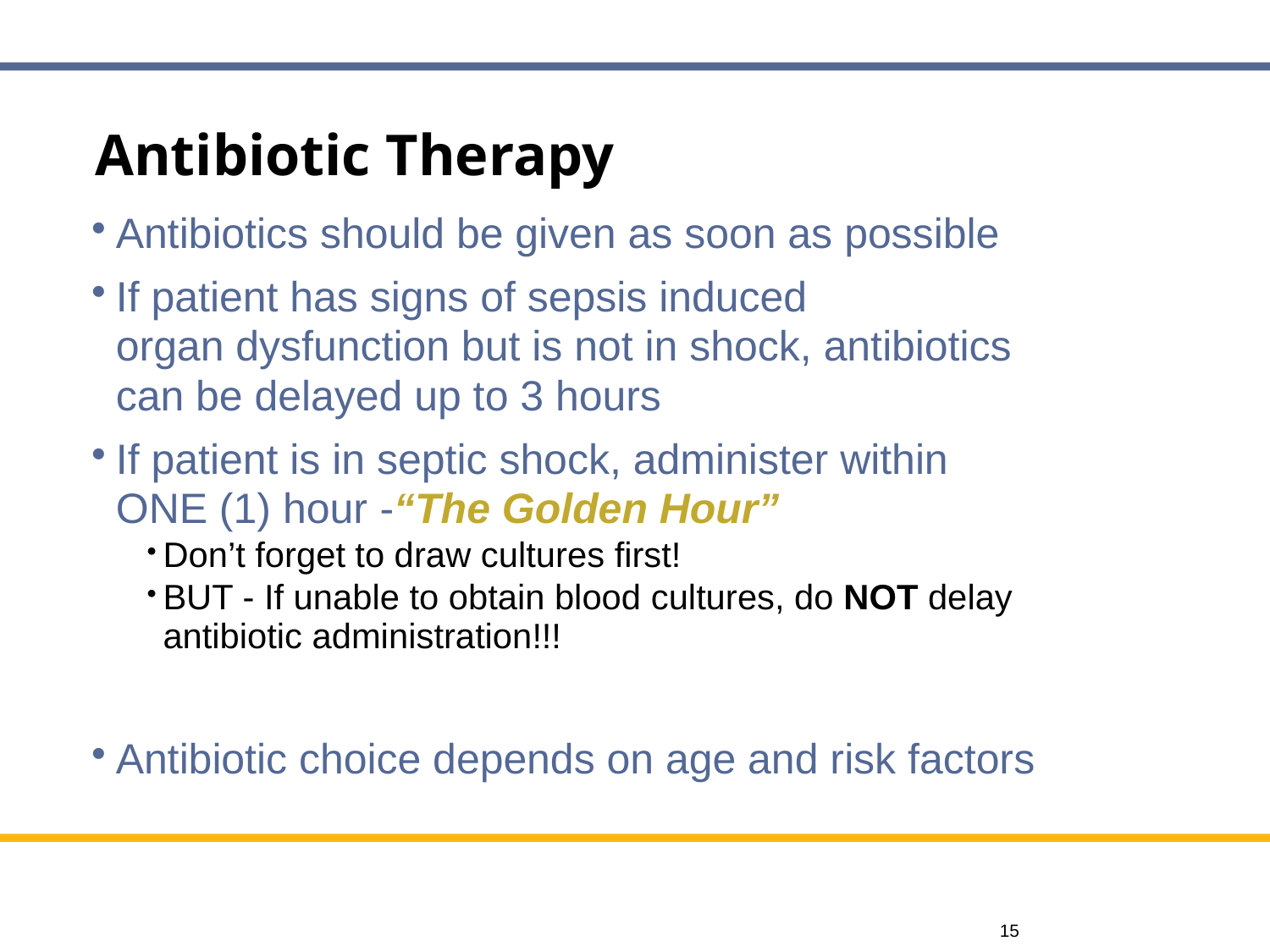

Antibiotic Therapy
Antibiotics should be given as soon as possible
If patient has signs of sepsis induced organ dysfunction but is not in shock, antibiotics can be delayed up to 3 hours
If patient is in septic shock, administer within ONE (1) hour -“The Golden Hour”
Don’t forget to draw cultures first!
BUT - If unable to obtain blood cultures, do NOT delay antibiotic administration!!!
Antibiotic choice depends on age and risk factors
15

## Slide 16
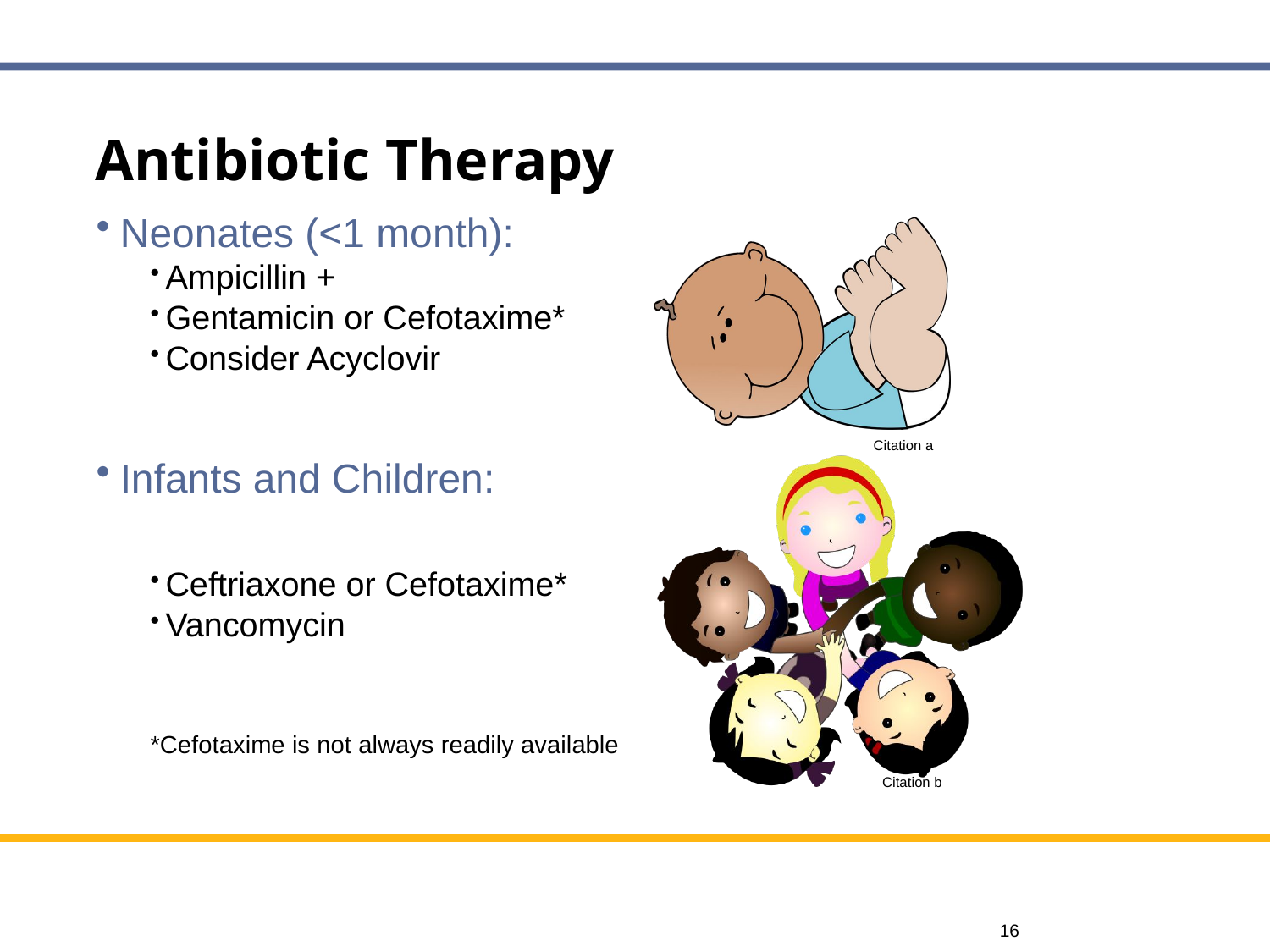

Antibiotic Therapy
Neonates (<1 month):
Ampicillin +
Gentamicin or Cefotaxime*
Consider Acyclovir
Infants and Children:
Ceftriaxone or Cefotaxime*
Vancomycin
*Cefotaxime is not always readily available
Citation a
Citation b
16

## Slide 17
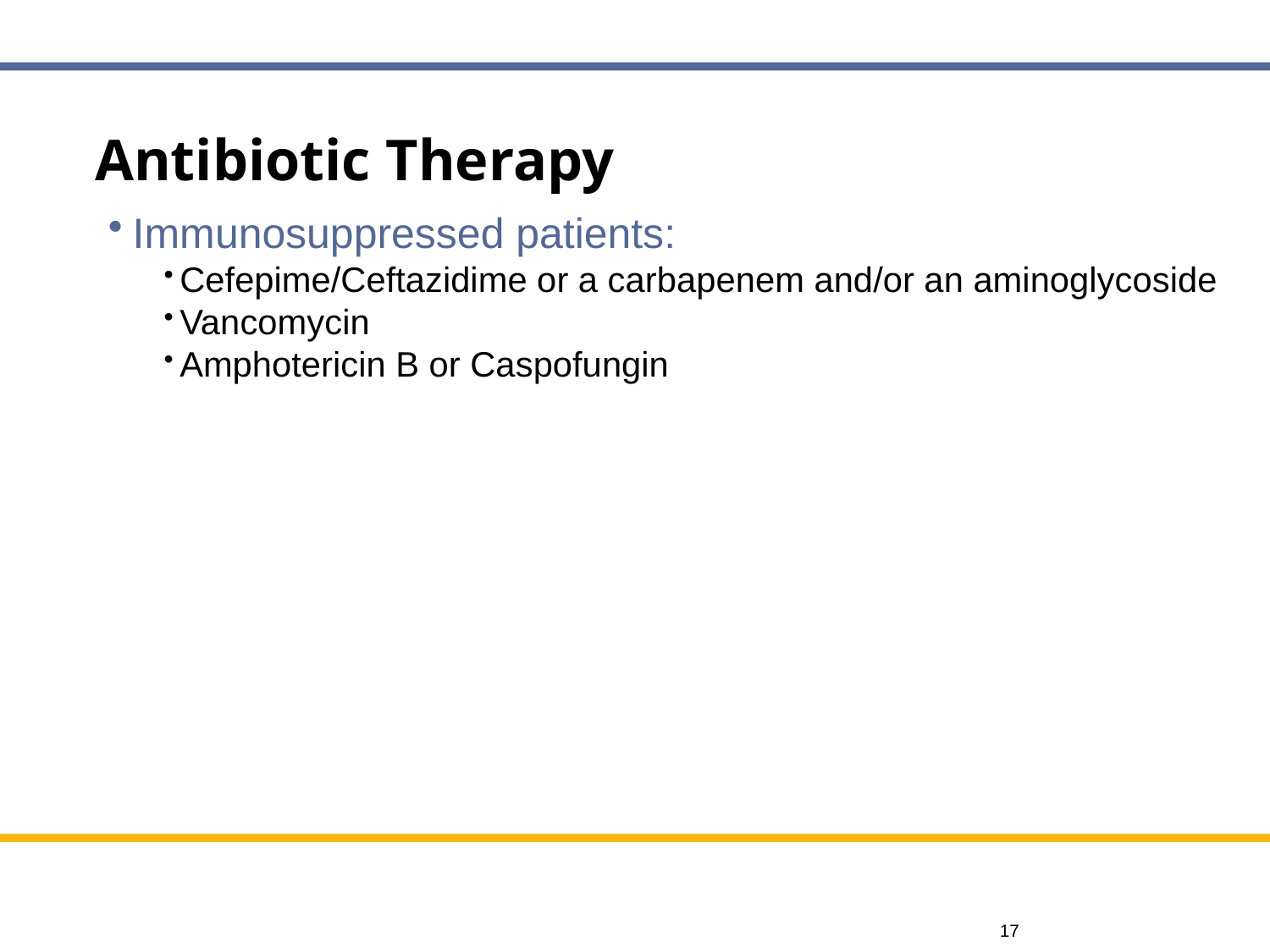

Antibiotic Therapy
Immunosuppressed patients:
Cefepime/Ceftazidime or a carbapenem and/or an aminoglycoside
Vancomycin
Amphotericin B or Caspofungin
17

## Slide 18
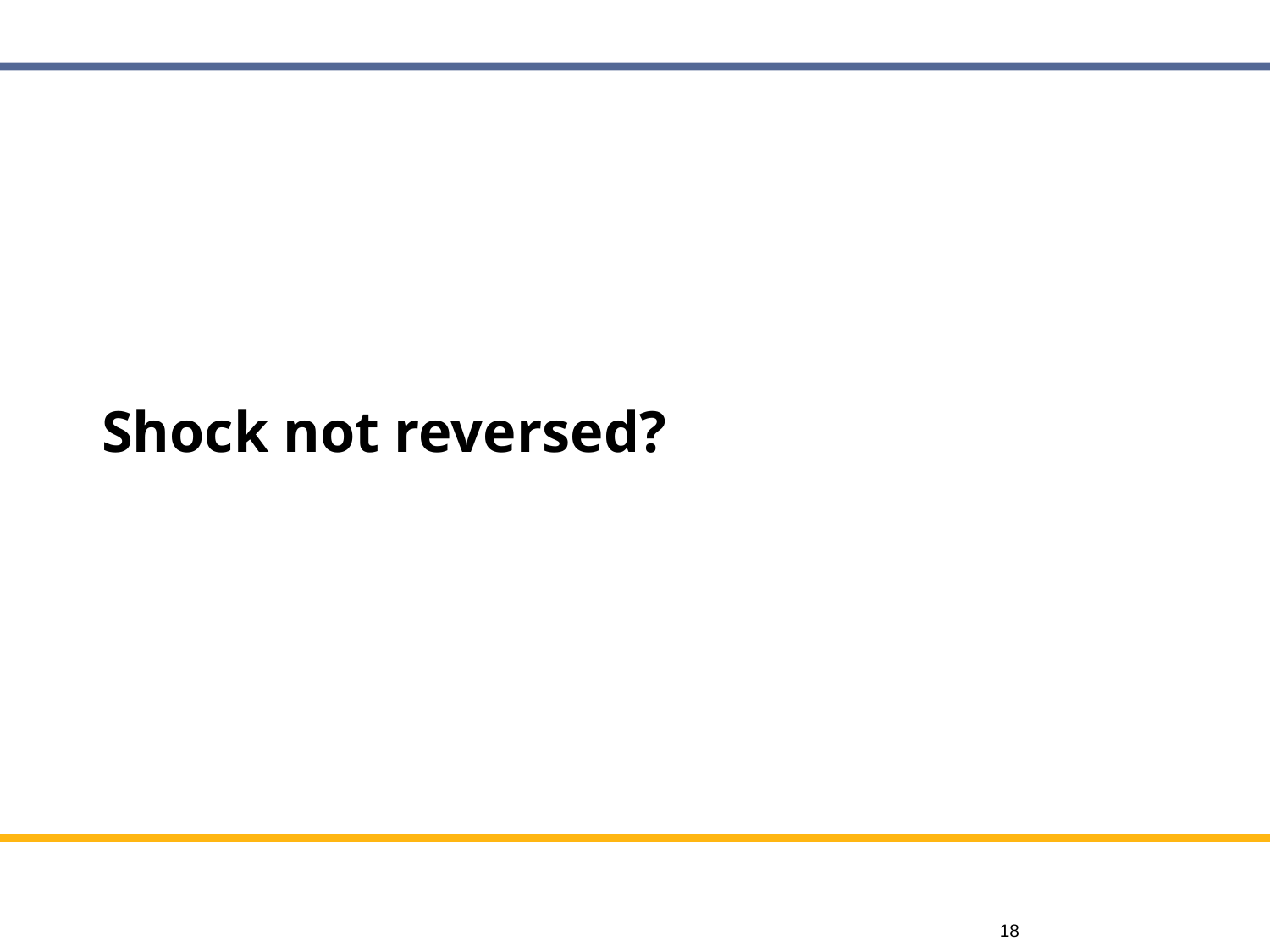

Shock not reversed?
18

## Slide 19
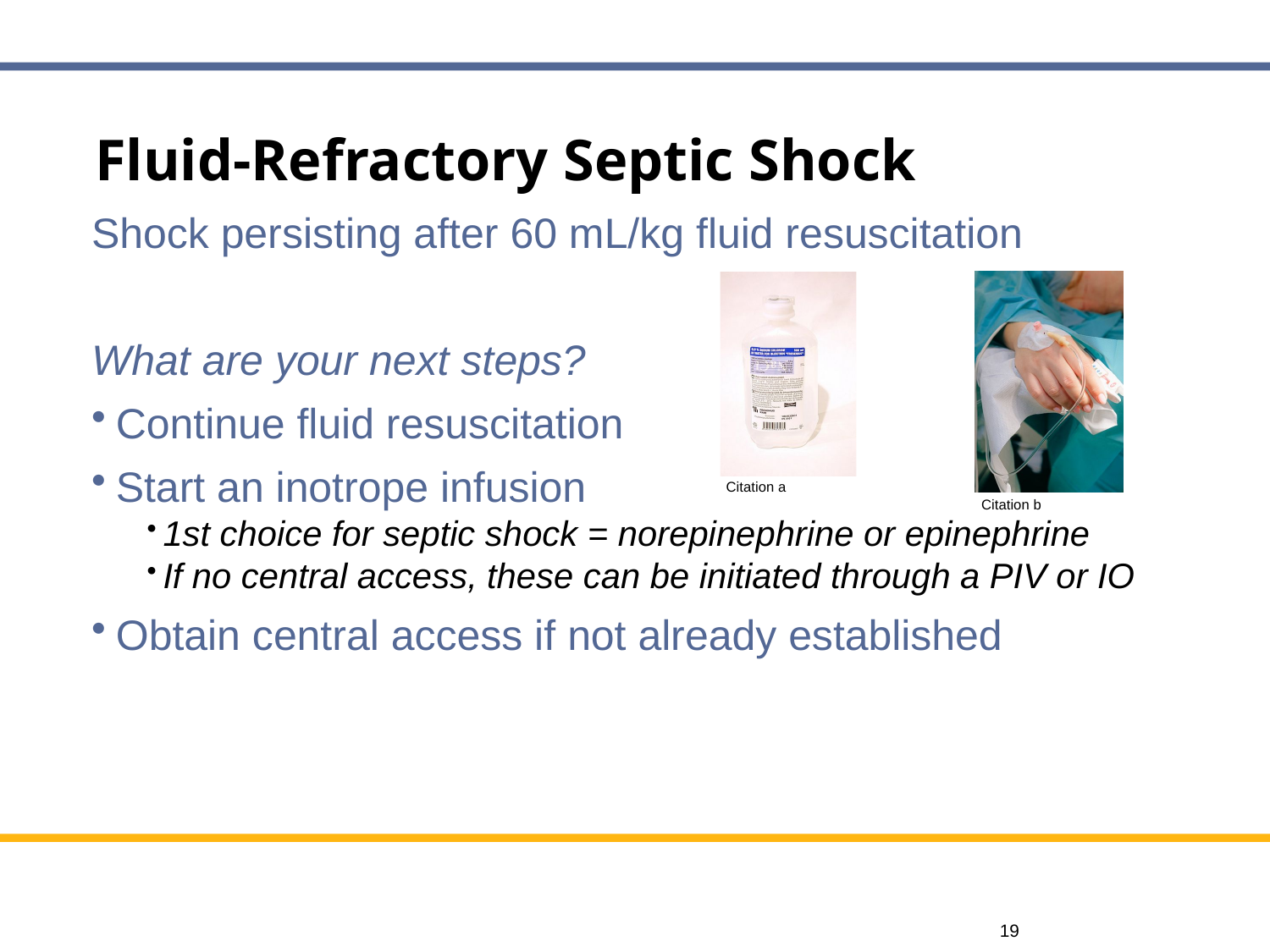

Fluid-Refractory Septic Shock
Shock persisting after 60 mL/kg fluid resuscitation
What are your next steps?
Continue fluid resuscitation
Start an inotrope infusion
1st choice for septic shock = norepinephrine or epinephrine
If no central access, these can be initiated through a PIV or IO
Obtain central access if not already established
Citation a
Citation b
19

## Slide 20
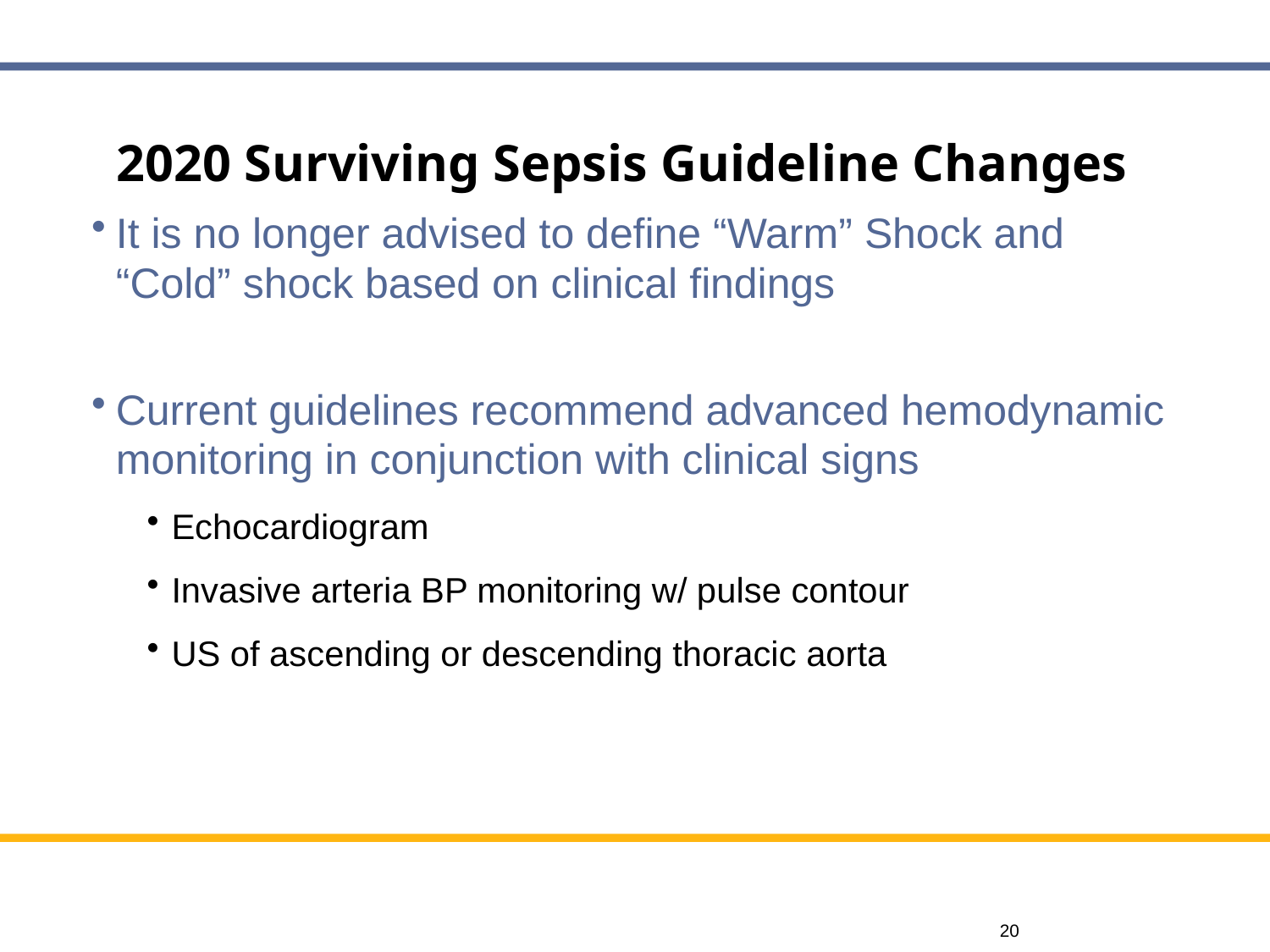

2020 Surviving Sepsis Guideline Changes
It is no longer advised to define “Warm” Shock and “Cold” shock based on clinical findings
Current guidelines recommend advanced hemodynamic monitoring in conjunction with clinical signs
Echocardiogram
Invasive arteria BP monitoring w/ pulse contour
US of ascending or descending thoracic aorta
20

## Slide 21
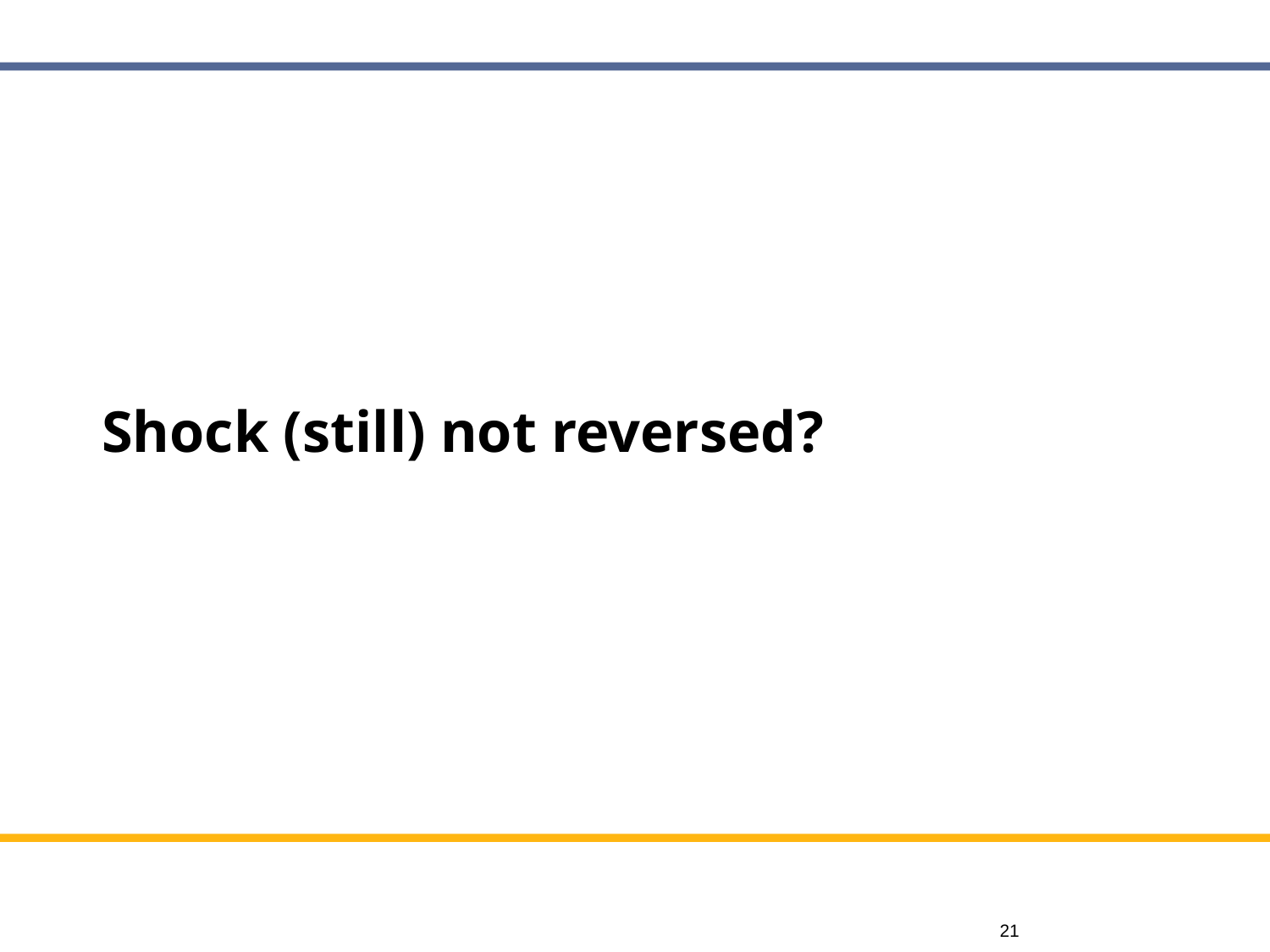

Shock (still) not reversed?
21

## Slide 22
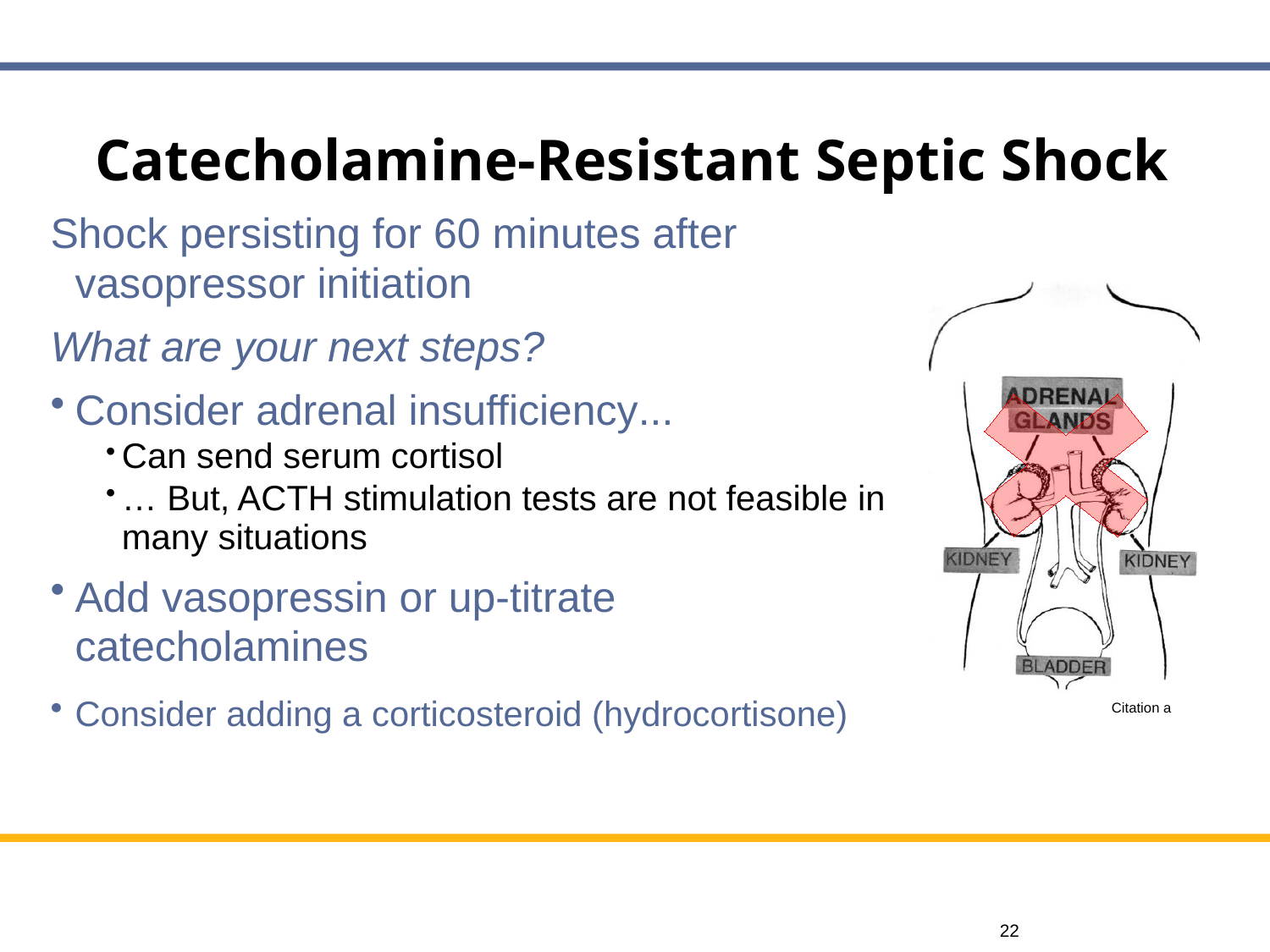

Catecholamine-Resistant Septic Shock
Shock persisting for 60 minutes after vasopressor initiation
What are your next steps?
Consider adrenal insufficiency...
Can send serum cortisol
… But, ACTH stimulation tests are not feasible in many situations
Add vasopressin or up-titrate catecholamines
Consider adding a corticosteroid (hydrocortisone)
Citation a
22

## Slide 23
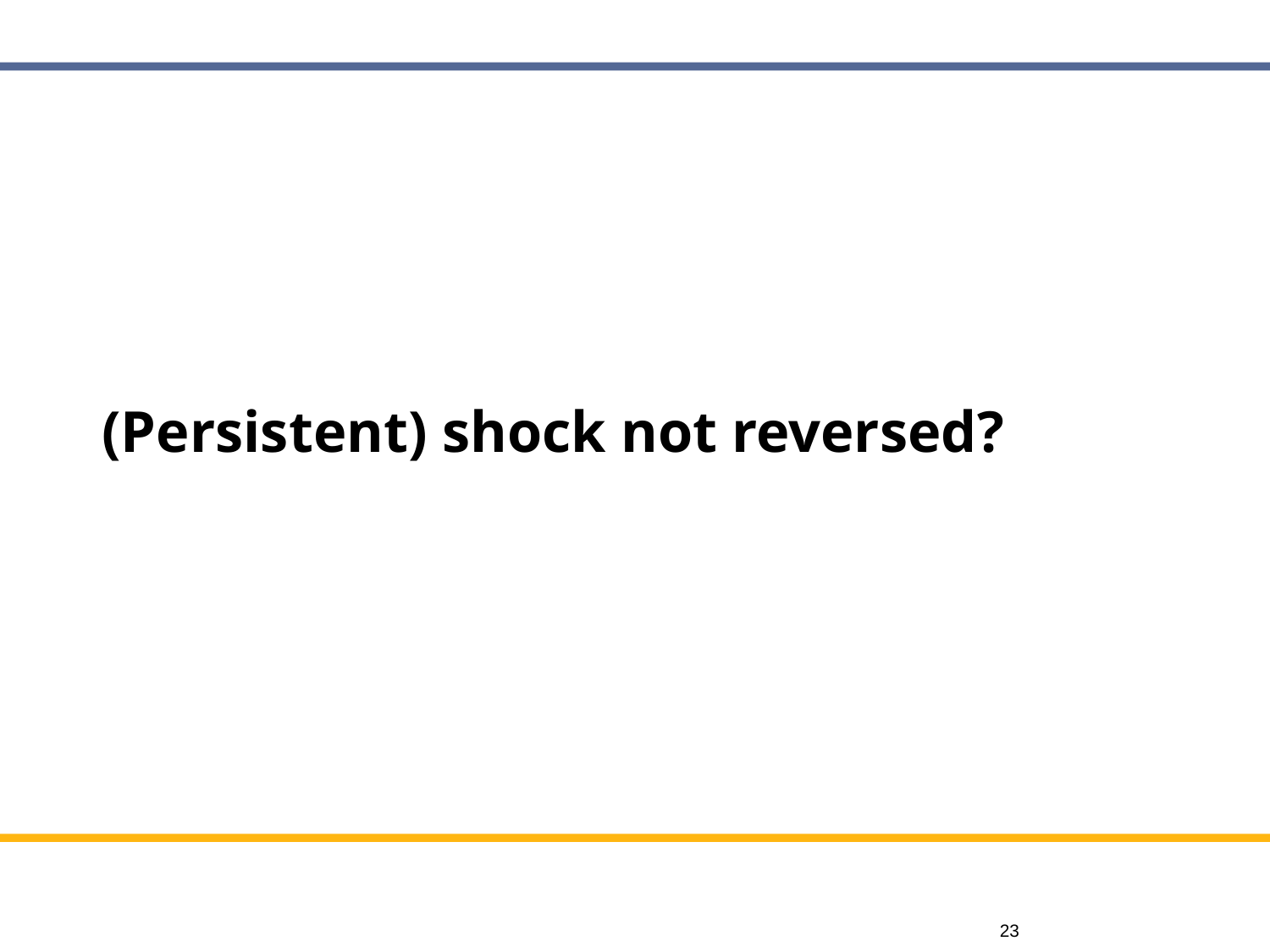

(Persistent) shock not reversed?
23

## Slide 24
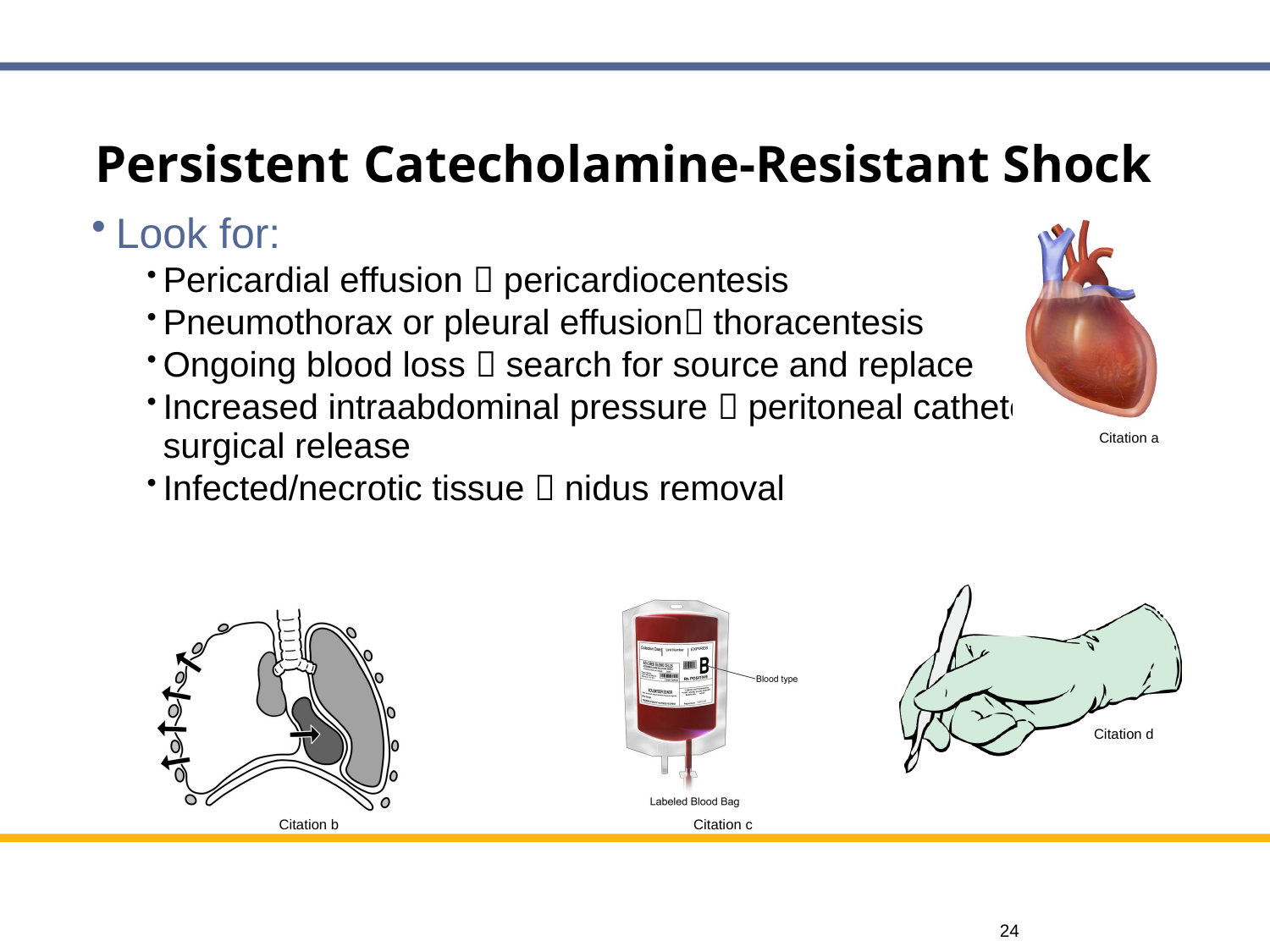

Persistent Catecholamine-Resistant Shock
Look for:
Pericardial effusion  pericardiocentesis
Pneumothorax or pleural effusion thoracentesis
Ongoing blood loss  search for source and replace
Increased intraabdominal pressure  peritoneal catheter or surgical release
Infected/necrotic tissue  nidus removal
Citation a
Citation d
Citation b
Citation c
24

## Slide 25
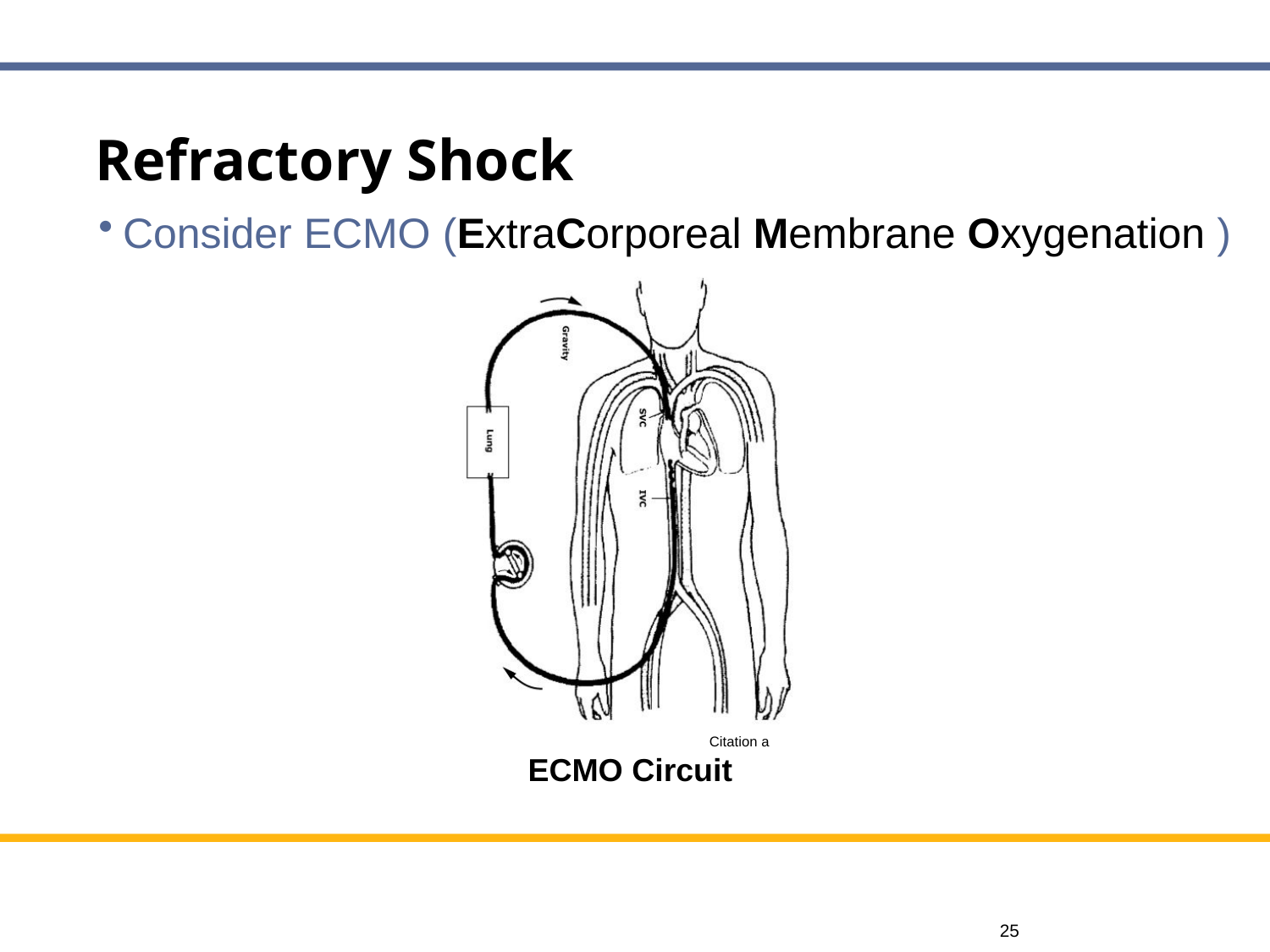

Refractory Shock
Consider ECMO (ExtraCorporeal Membrane Oxygenation )
Citation a
ECMO Circuit
25

## Slide 26
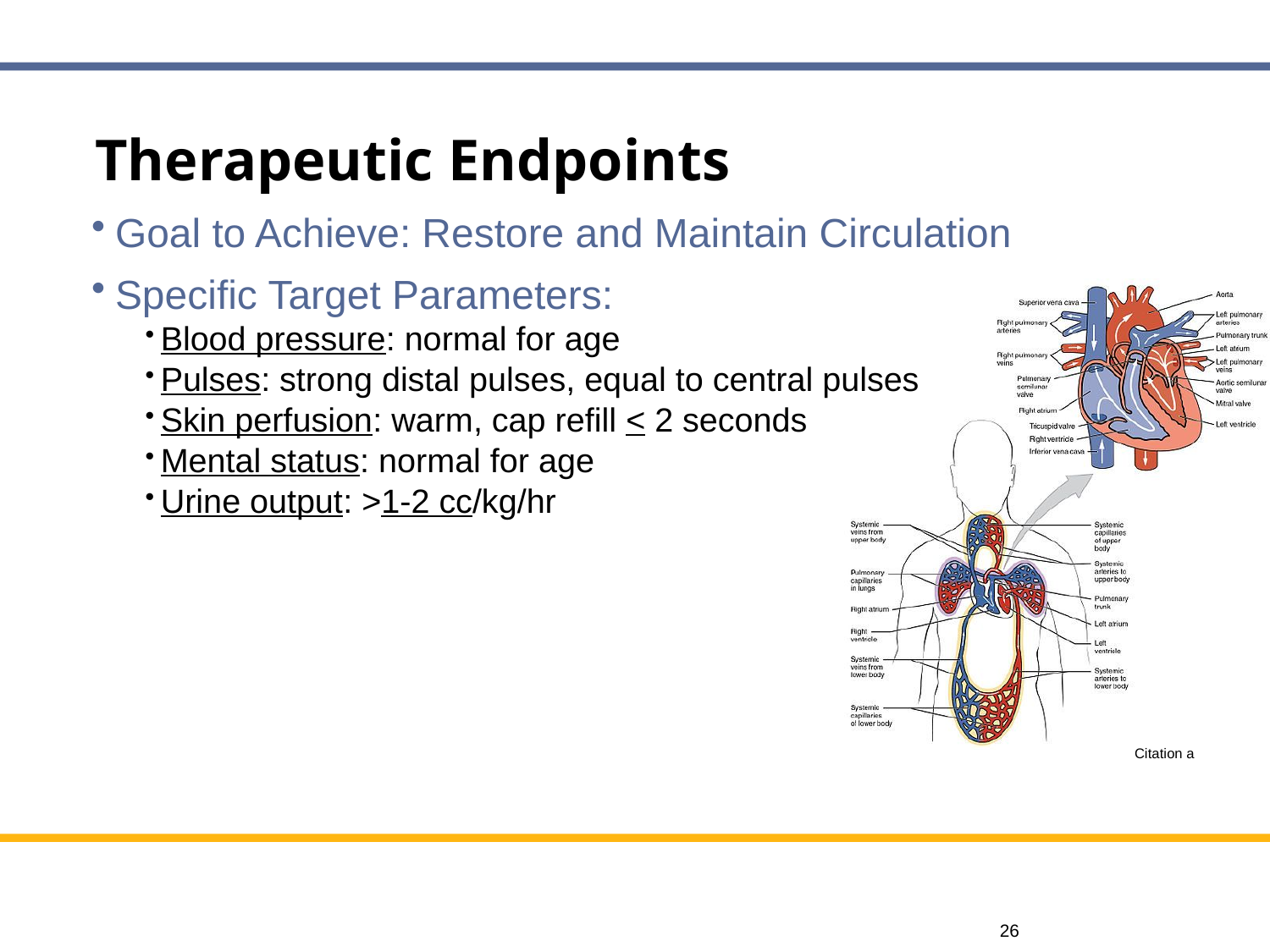

Therapeutic Endpoints
Goal to Achieve: Restore and Maintain Circulation
Specific Target Parameters:
Blood pressure: normal for age
Pulses: strong distal pulses, equal to central pulses
Skin perfusion: warm, cap refill < 2 seconds
Mental status: normal for age
Urine output: >1-2 cc/kg/hr
Citation a
26

## Slide 27
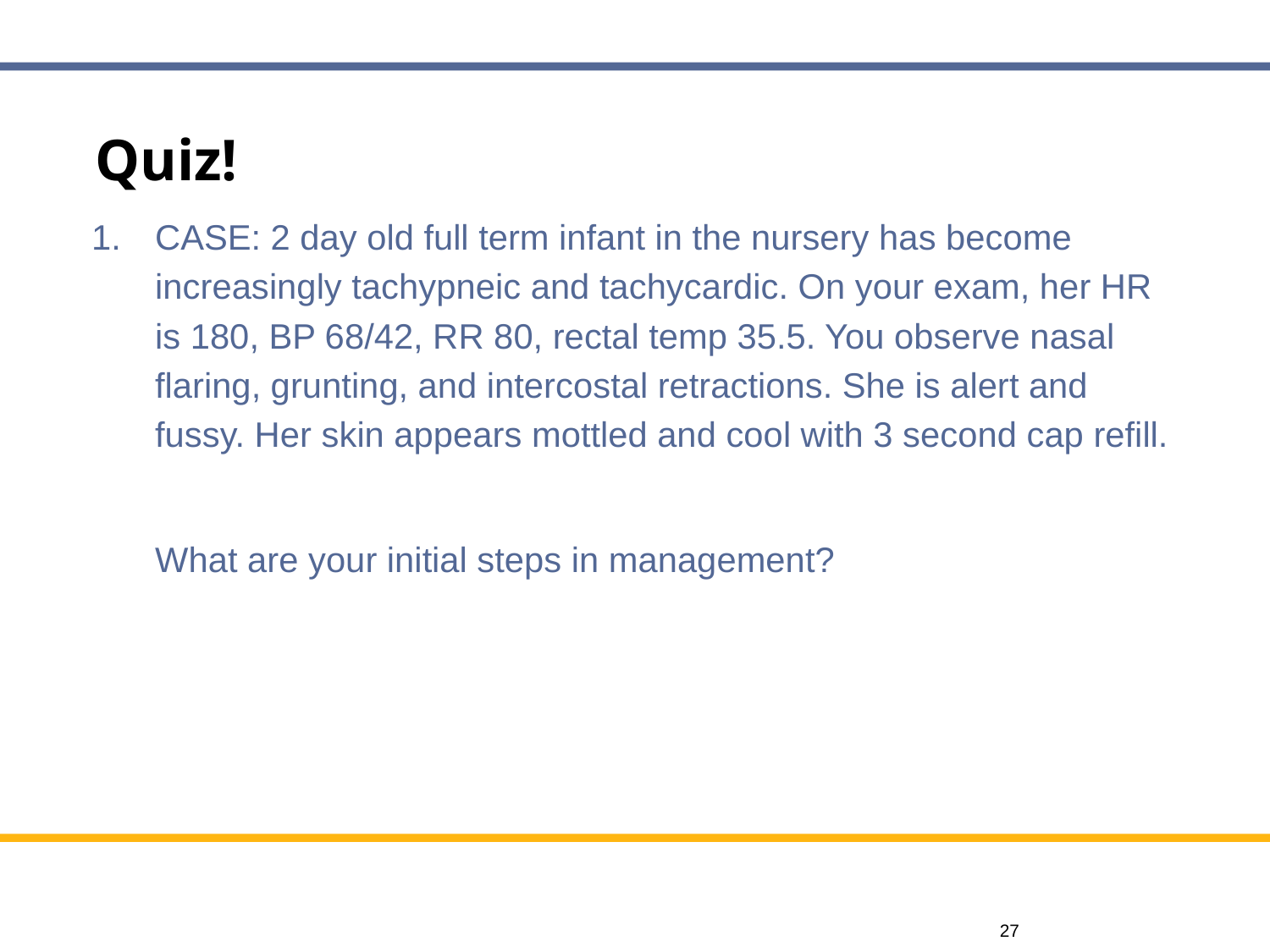

Quiz!
CASE: 2 day old full term infant in the nursery has become increasingly tachypneic and tachycardic. On your exam, her HR is 180, BP 68/42, RR 80, rectal temp 35.5. You observe nasal flaring, grunting, and intercostal retractions. She is alert and fussy. Her skin appears mottled and cool with 3 second cap refill.
	What are your initial steps in management?
27

## Slide 28
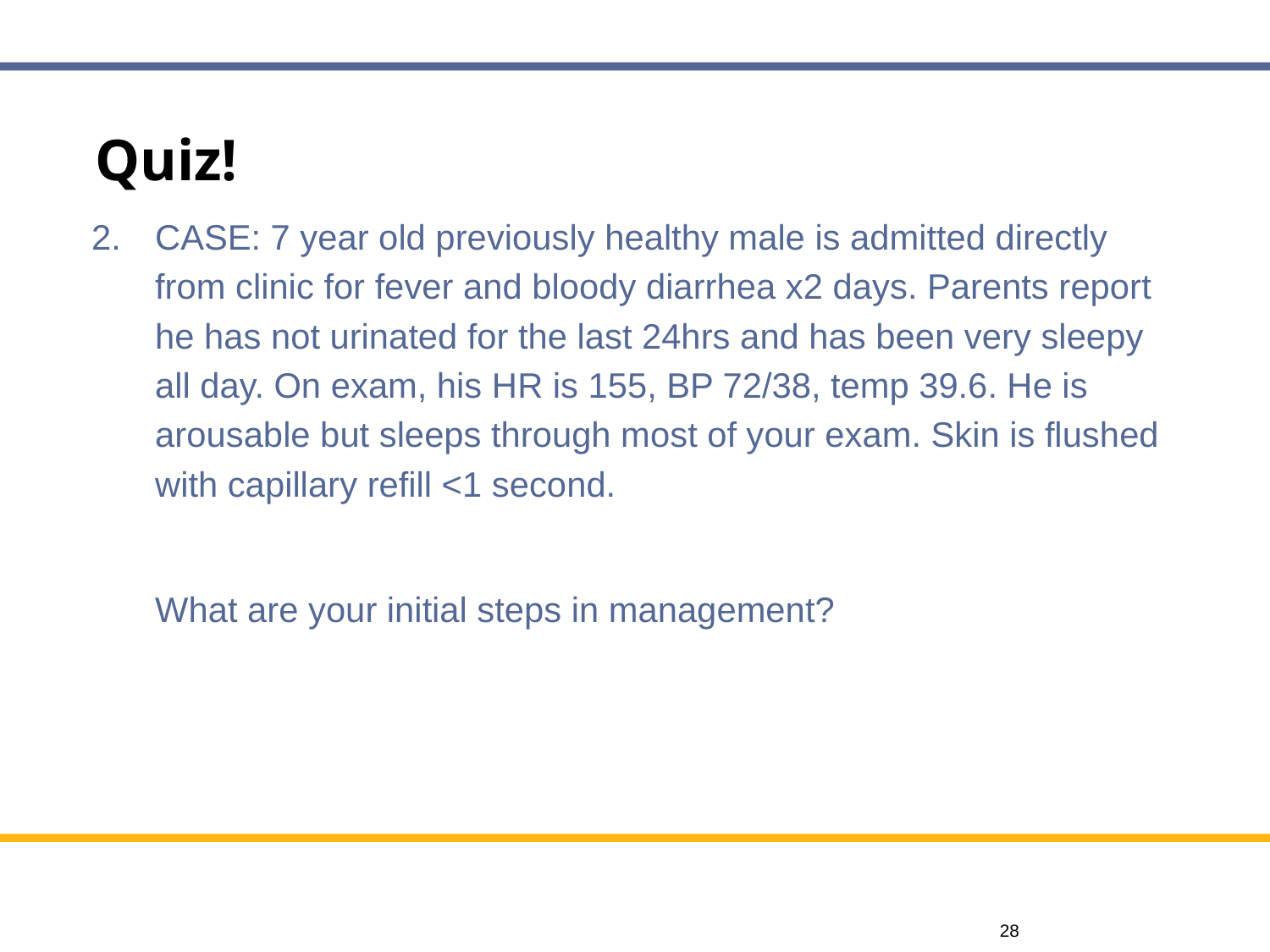

Quiz!
CASE: 7 year old previously healthy male is admitted directly from clinic for fever and bloody diarrhea x2 days. Parents report he has not urinated for the last 24hrs and has been very sleepy all day. On exam, his HR is 155, BP 72/38, temp 39.6. He is arousable but sleeps through most of your exam. Skin is flushed with capillary refill <1 second.
	What are your initial steps in management?
28

## Slide 29
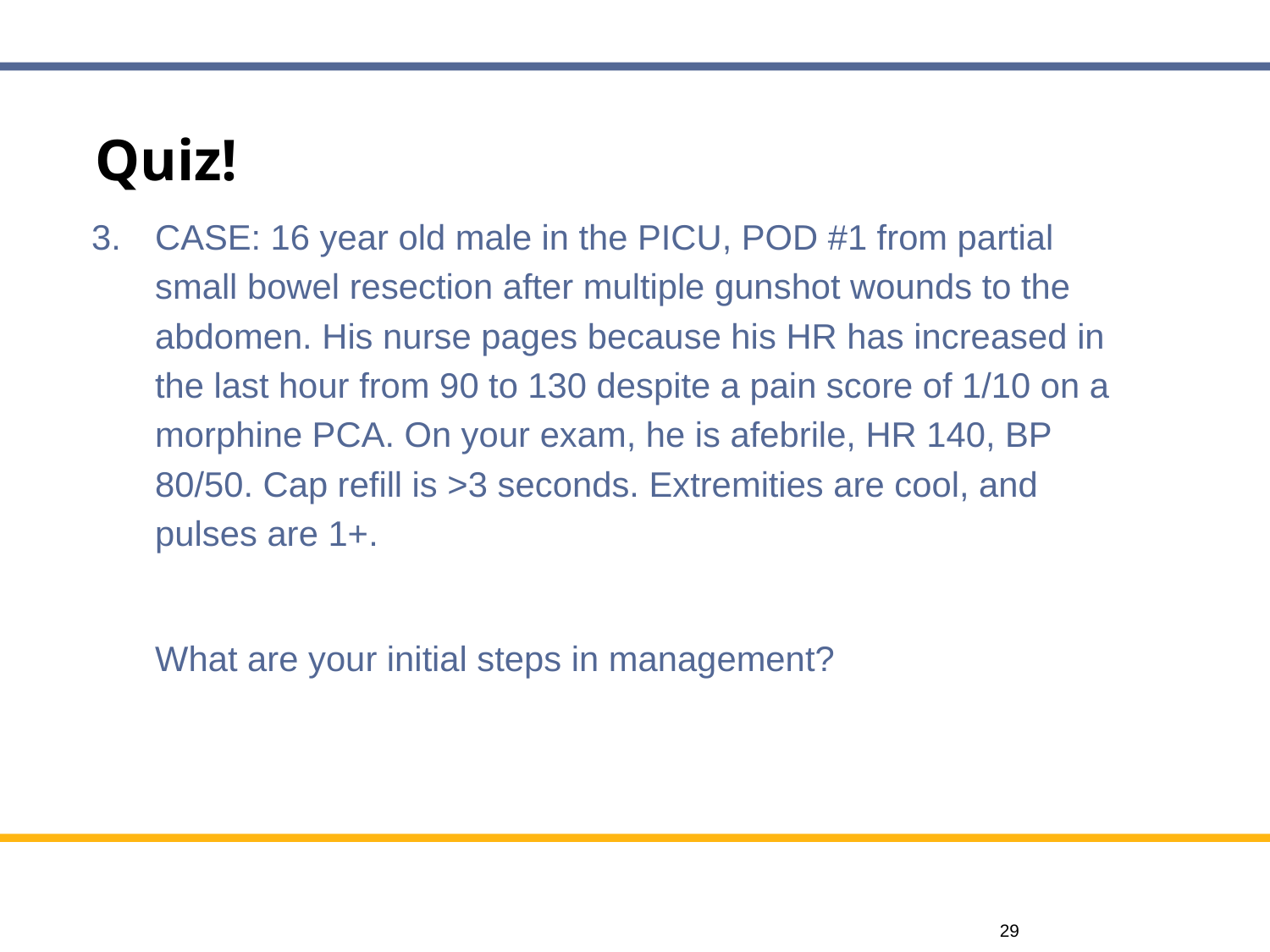

Quiz!
3.	CASE: 16 year old male in the PICU, POD #1 from partial small bowel resection after multiple gunshot wounds to the abdomen. His nurse pages because his HR has increased in the last hour from 90 to 130 despite a pain score of 1/10 on a morphine PCA. On your exam, he is afebrile, HR 140, BP 80/50. Cap refill is >3 seconds. Extremities are cool, and pulses are 1+.
	What are your initial steps in management?
29

## Slide 30
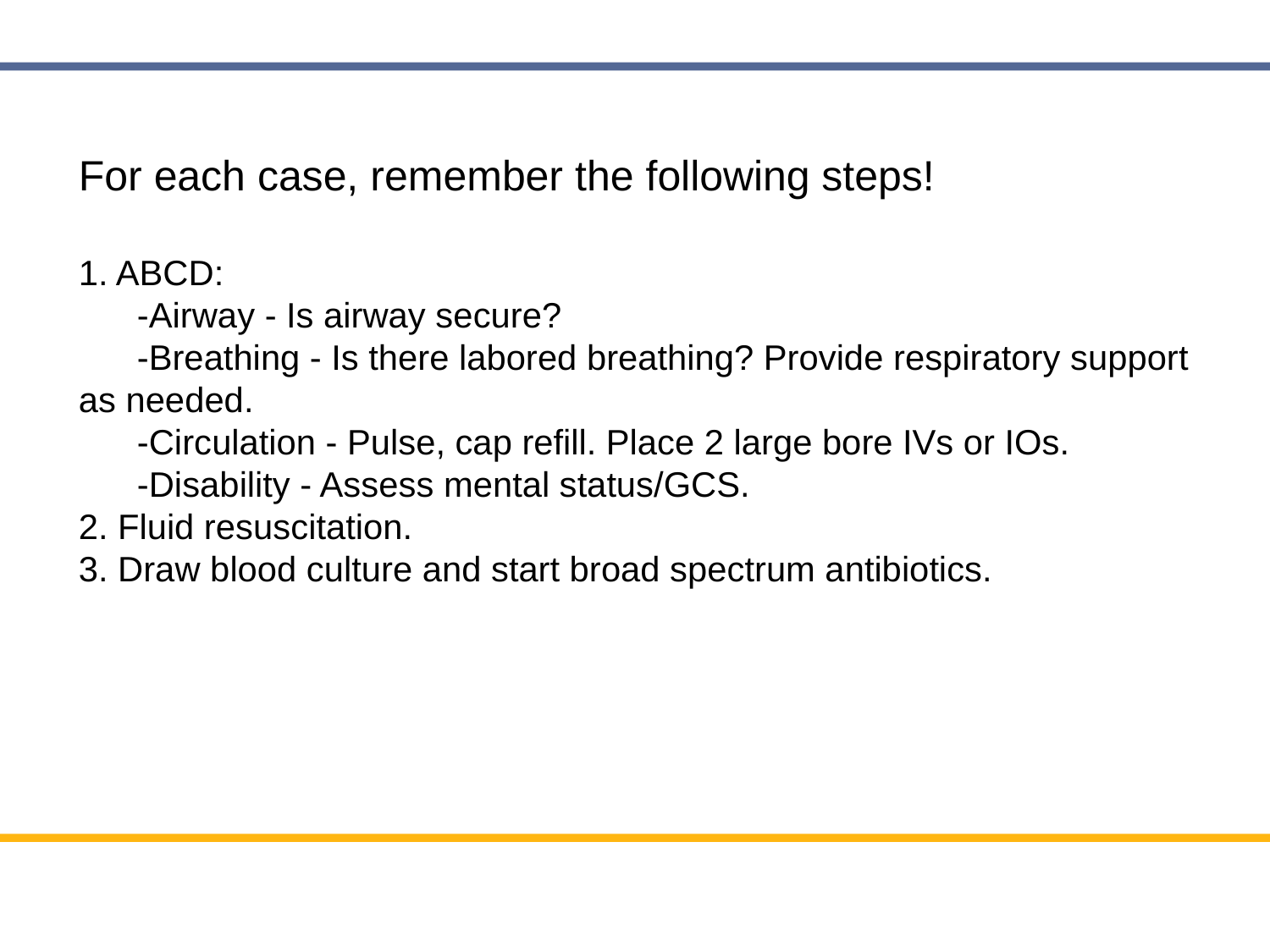

For each case, remember the following steps!
1. ABCD:
      -Airway - Is airway secure?
      -Breathing - Is there labored breathing? Provide respiratory support as needed.
      -Circulation - Pulse, cap refill. Place 2 large bore IVs or IOs.
      -Disability - Assess mental status/GCS.
2. Fluid resuscitation.
3. Draw blood culture and start broad spectrum antibiotics.

## Slide 31
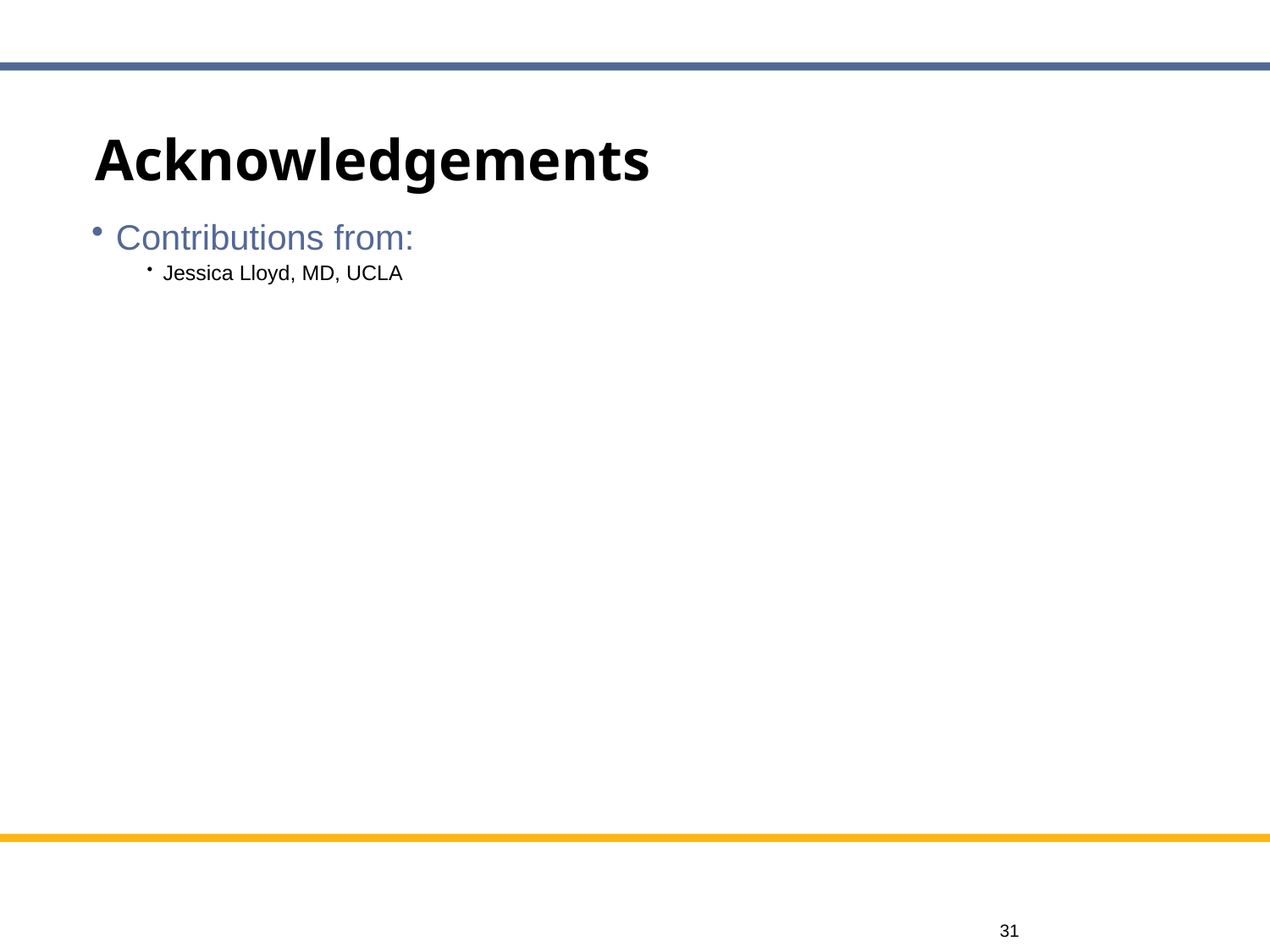

Acknowledgements
Contributions from:
Jessica Lloyd, MD, UCLA
31
