# Supplementary material for: Identification and Management of Pediatric Sepsis: A Medical Student Curricular Supplement for PICU and NICU Rotations
Source: MedEdPORTAL. 2021 Apr 23;17:11142. doi: 10.15766/mep_2374-8265.11142 (PMC8063627; doi:10.15766/mep_2374-8265.11142)
Supplement: Supplementary file 1 — Pre- & Posttest.docxModule 1 - Pediatric Shock.pptxScript 1 - Pediatric Shock.docxModule 2 - Pediatric Sepsis.pptxScript 2 - Pediatric Sepsis.docxModule 3 - Management of Sepsis & Septic Shock.pptxScript 3 - Management of Sepsis & Septic Shock. docxModule 4 - Hemodynamics & Pressor Support.pptxScript 4 - Hemodynamics & Pressor Support.docxSimulation Case 1.docxSimulation Case 2.docxSimulation Case 3.docxPostsimulation Review Quiz.pptx [file mep_2374-8265.11142-s001.zip › G. Script 3 - Management of Sepsis & Septic Shock.docx]

**Management of Sepsis and Septic Shock**

1. This education module continues our Pediatric Sepsis online curriculum. We will be discussing the management of sepsis and septic shock.
2. The learning objectives are:
   - Describe the Surviving Sepsis Guidelines for management of severe sepsis and septic shock
   - Identify the goals for resuscitation of pediatric patients with septic shock
   - Determine the appropriate antibiotic choices for pediatric patients with sepsis
3. Our goals for resuscitation are based on the guidelines from the Surviving Sepsis Campaign. These learning modules have been updated according to the 2020 Surviving Sepsis Guidelines. This campaign refers to an international collaboration of experts who worked together to establish an algorithm, which outlines the steps for management of the patient presenting with severe sepsis or in septic shock. In particular, pediatric patients benefit most from early goal-directed therapies.
4. Here is the resuscitation algorithm for pediatric patients. We will be discussing these steps in more detail during this education module.
5. Our primary goal for resuscitation of a patient with severe sepsis or septic shock is to restore and maintain circulation. This is achieved by meeting specific target parameters. Some parameters include strong distal pulses that are equal to central pulses, warm and well-perfused skin with a cap refill <2 seconds and mental status appropriate for age. Although not specified in the 2020 Sepsis Guidelines, in practice we typically aim for a urine output greater than 1 mL/kg/hr. Similarly, the guidelines do not recommend a target MAP, however in practice, MAPs between the 5^th^-50^th^ percentile for age are often targeted.
6. PEARL – Here’s a helpful pediatric pearl for you. How do you recognize hypotension in the pediatric patient? You can look at the mean arterial pressure <5^th^ percentile for age OR you can look at the systolic blood pressure less than 2 standard deviations below normal for age. Alternatively, I can give you an easy and short calculation! For children ages 1 through 10, just multiply their age by 2 and add to 70, as shown in the table here. Of note, a systolic pressure greater than 60 is generally considered adequate for a neonate.
7. Now in the first 5 minutes of your resuscitation, you’re going to be sure to assess your ABCs – airway, breathing, and circulation. Place your patient on a cardiac monitor, start supplemental oxygen by facemask, high-flow nasal cannula, or CPAP. And you’ll be prepared to intubate if the patient is presenting in respiratory failure.
8. PEARL – Here comes another pediatric pearl! Did you know, in children, up to 40% of their cardiac output may be required to support the work of breathing? So, by providing ventilatory support, more cardiac output can be directed to vital organs.
9. Also, within the first 5 minutes, you will need to obtain access. Ideally, you’ll place 2 large bore peripheral IVs, however this is often a challenge for pediatric patients in shock. If your attempts for an IV are unsuccessful, place an intraosseous line. Don’t spend more than 5 minutes trying for a PIV!
10. Once you obtain access, you need to begin fluid resuscitation immediately. There are several important questions to ask when initiating fluid resuscitation. The first is what fluids should be given. Isotonic crystalloid such as normal saline or Lactated Ringer’s is the standard. However, the 2020 Surviving Sepsis Guidelines recommends buffered or balanced crystalloids as opposed to normal saline, and does not recommend albumin for fluid resuscitation given cost and barriers to administration. Another important question is how much fluid to give. Give 10-20 mL/kg boluses run over 5-10 minutes. Patients usually require at least 40 to 60 mL/kg total of IV fluids, but there is no set limit on fluids. IV Fluid may be administered until perfusion improves and circulation is restored OR if the patient exhibits adverse effects such as hepatomegaly or pulmonary crackles, also known as rales.
11. Now you may be thinking about another fluid product such as packed red blood cells. Should you give blood? Yes, if there’s any evidence of trauma or acute hemorrhage. Prior guidelines advised considering transfusing packed RBCs after administering 60mL/kg of isotonic crystalloid. Previously, it was thought that *during* your resuscitation, your goal hemoglobin was greater than 10 in order to achieve a central venous saturation >70%, and that *after* your resuscitation, the goal should be lowered to a hemoglobin >7. However, according to the 2020 Surviving Sepsis Guidelines, there is no longer a recommendation for a hemoglobin transfusion threshold for children with unstable septic shock. There is no indication to transfuse a hemodynamically stable child with concentrated hemoglobin >7 as long as the MAP is > 2 SDs below normal for age and there has been no increase in vasoactive agents for > 2 hours.
12. During your initial resuscitation, it is also important to correct any metabolic derangements. Check stat electrolytes and glucose. For hypoglycemia (defined as a blood glucose <45 in the newborn or <70 in children), correct with 10% dextrose. In children, you can give higher concentrations of dextrose such as D25 if they have a central line, however in neonates you will generally be using D10 water. It is important to note that according to the 2020 Surviving Sepsis Guidelines, there is no clear recommendation for repleting to normal calcium levels, however this is often done in clinical practice for children needing vasoactive infusion support. For hypocalcemia, correct with calcium gluconate, or if they have a central line, you can administer calcium chloride. Suggested doses are listed for you on this slide.
13. PEARL – Time for another pearl! You may have already noticed that for our pediatric patients, all fluids and medications are dosed by weight. So, what do you do if you don’t know your patient’s weight in an emergency situation? You can use the Broselow tape, which is a colored coded measure of weight based on the child’s height, shown here in the picture. There are also several Broselow apps that you can download to your Android or iPhone.
14. We’ve already discussed the importance of checking for metabolic derangements, but there are other important labs to obtain as well. For all patients, you should obtain blood and urine cultures, a complete blood count, complete metabolic panel with an ionized calcium, magnesium, and phosphorus, a blood gas, serum lactate, and coagulation studies. For some patients, you may also want to consider obtaining fungal cultures, sputum cultures, viral studies, and performing an LP to send CSF studies after the initial stabilization/resuscitation.
15. In general, antibiotics should be given as quickly as possible in any patient presenting with sepsis. However, if the patient has signs of sepsis induced organ dysfunction but is not in shock, it is allowable to wait up to 3 hours prior to antibiotic administration. Otherwise, it is suggested to administer antibiotics within the first hour of identification of shock. This is commonly referred to as “the golden hour.” You should always try to obtain cultures prior to administering antibiotics, but if you are unable to obtain cultures for any reason, do NOT delay your antibiotics! Your choice of antibiotic depends on the patient’s age and risk factors, beginning with broad spectrum antibiotics, and narrowing as soon as possible to prevent resistance.
16. For neonates less than one month, give ampicillin and gentamicin or cefotaxime. It is important to note that cefotaxime is not always readily available. The combination of ampicillin and gentamicin will adequately cover for E.coli, GBS, and Listeria, which are the 3 most common causes of sepsis in the neonate. You can also consider acyclovir, as HSV can present with severe and devastating sepsis that causes permanent adverse neurologic sequelae. For infants and children, give a 3^rd^ generation cephalosporin such as ceftriaxone or cefotaxime if available, in addition to vancomycin depending on risk factors.
17. Immunocompromised patients represent a special category that requires additional antibiotic and antifungal considerations. You will need to administer an antipseudomonal agent such as cefepime, a carbepenem, and/or an aminoglycoside. You will also give vancomycin for MRSA coverage, and an antifungal such as fluconazole, amphotericin B or caspofungin.
18. So, if you’ve done all of these interventions, what do you do if shock is still present?
19. We have now entered a stage known as fluid-refractory shock. This is defined as failure to restore circulation after a total 60mL/kg of fluid resuscitation. What are your next steps at this point?

- Keep giving fluid boluses!
- Start a vasoactive medication, such as norepinephrine, or epinephrine. Dopamine is no longer recommended as a first line agent and should only be used if epinephrine or norepinephrine are not available.
- These medications can be given peripherally short-term. Your choice of vasopressor will depend on the patient’s clinical presentation.
- You will eventually need to obtain central access.

1. Previously, shock had been categorized based on clinical findings to define “hot” or “cold” shock. It is now not advised to rely heavily on clinical findings alone to make such distinctions, and instead it is recommended to use advanced hemodynamic monitoring in conjunction with clinical signs to determine type or types of shock present in your patient. Such types of monitoring include echocardiogram, invasive arterial BP monitoring with pulse contour, as well as ultrasound of the ascending or descending thoracic aorta.
2. So, if you’ve done this intervention, what do you do if shock is still present?
3. If shock is still not reversed after providing pressor support for at least 60 minutes, your patient has entered as stage known as catecholamine-resistant shock. It was previously recommended to perform an ACTH stimulation test, however this is not feasible in many situations. Current guidelines instead advise the addition of vasopressin or up-titration of catecholamines. You may also consider the addition of a corticosteroid, such as hydrocortisone.
4. So, if you’ve done all of the above interventions, what do you do if shock is still present?
5. This state is known as persistent catecholamine-resistant shock. At this time, you need to consider other etiologies. Look for pericardial effusion, and if present perform pericardiocentesis. For pleural effusion or pneumothorax, perform thoracentesis. If the patient has ongoing blood losses, find the source and replace with PRBCs. For increased intraabdominal pressure, your patient may need a peritoneal drain or surgical release. Lastly, if there is infected or necrotic tissue present, the tissue should be removed.
6. Another option for persistent catecholamine-resistant shock is the use of ECMO, which stands for extracorporeal membrane oxygenation. This is essentially a lung bypass machine that removes the patient’s blood volume from the body, artificially oxygenates it, and then returns it to the patient. It is a very high-risk and technically challenging therapeutic intervention. It should only be considered if all other interventions have been unsuccessful.
7. So now let’s return to our initial primary goal. Your resuscitation is effective when you are able to restore and maintain your patient’s circulation, as evidenced by meeting your target parameters that we previously discussed. It is important to remember that an effective therapeutic endpoint may not necessarily be the outcome of every resuscitation.
8. We finish with a short quiz – Here are the 3 cases from the previous lecture on pediatric shock. Let’s now consider the steps in management of these patients. The cases will be discussed at your simulation session.
