# Supplementary material for: Identification and Management of Pediatric Sepsis: A Medical Student Curricular Supplement for PICU and NICU Rotations
Source: MedEdPORTAL. 2021 Apr 23;17:11142. doi: 10.15766/mep_2374-8265.11142 (PMC8063627; doi:10.15766/mep_2374-8265.11142)
Supplement: Supplementary file 1 — Pre- & Posttest.docxModule 1 - Pediatric Shock.pptxScript 1 - Pediatric Shock.docxModule 2 - Pediatric Sepsis.pptxScript 2 - Pediatric Sepsis.docxModule 3 - Management of Sepsis & Septic Shock.pptxScript 3 - Management of Sepsis & Septic Shock. docxModule 4 - Hemodynamics & Pressor Support.pptxScript 4 - Hemodynamics & Pressor Support.docxSimulation Case 1.docxSimulation Case 2.docxSimulation Case 3.docxPostsimulation Review Quiz.pptx [file mep_2374-8265.11142-s001.zip › H. Module 4 - Hemodynamics & Pressor Support.pptx]

## Slide 1
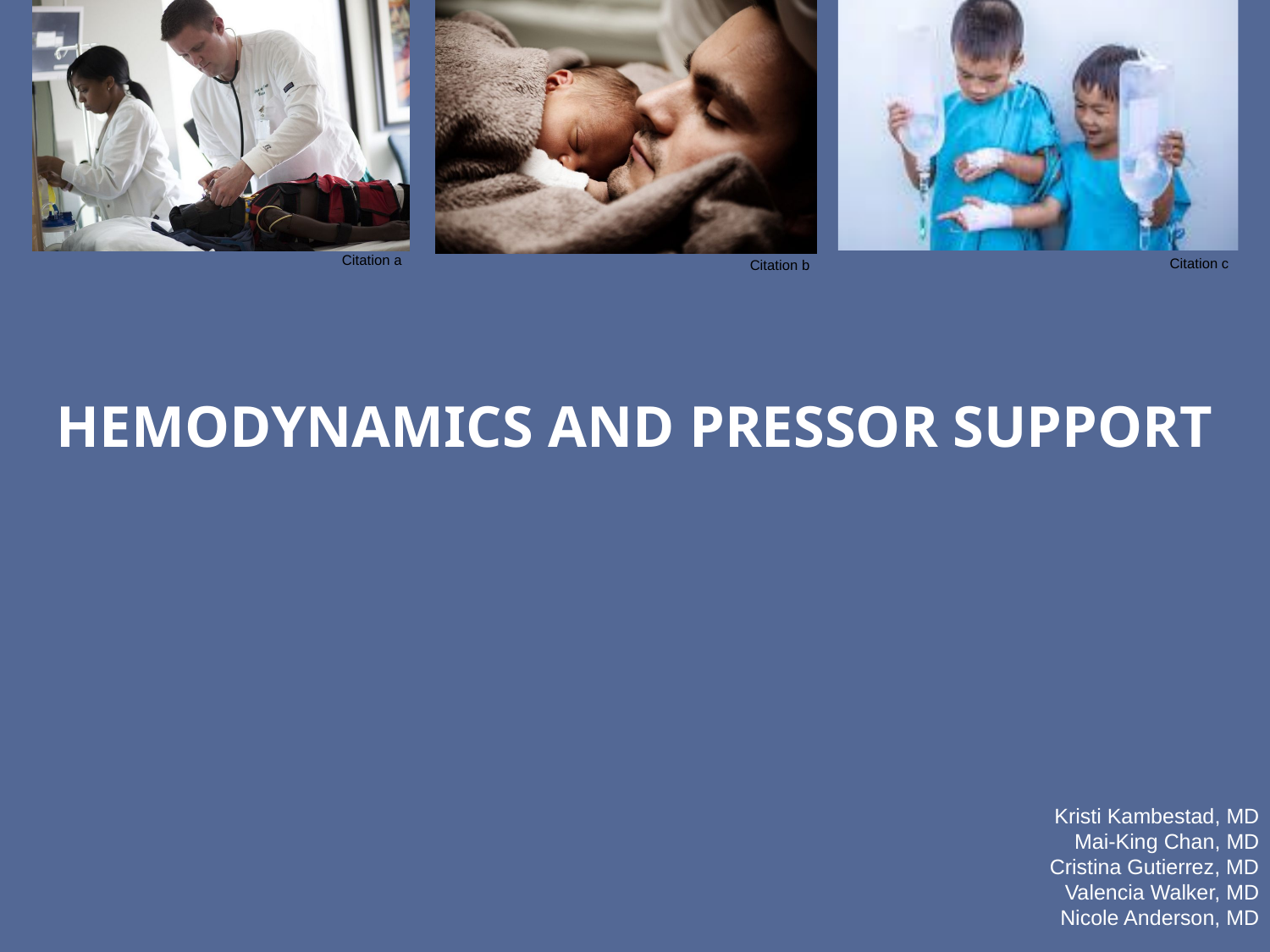

Citation a
Citation c
Citation b
HEMODYNAMICS AND PRESSOR SUPPORT
Kristi Kambestad, MD
Mai-King Chan, MD
Cristina Gutierrez, MD
Valencia Walker, MD
Nicole Anderson, MD

## Slide 2
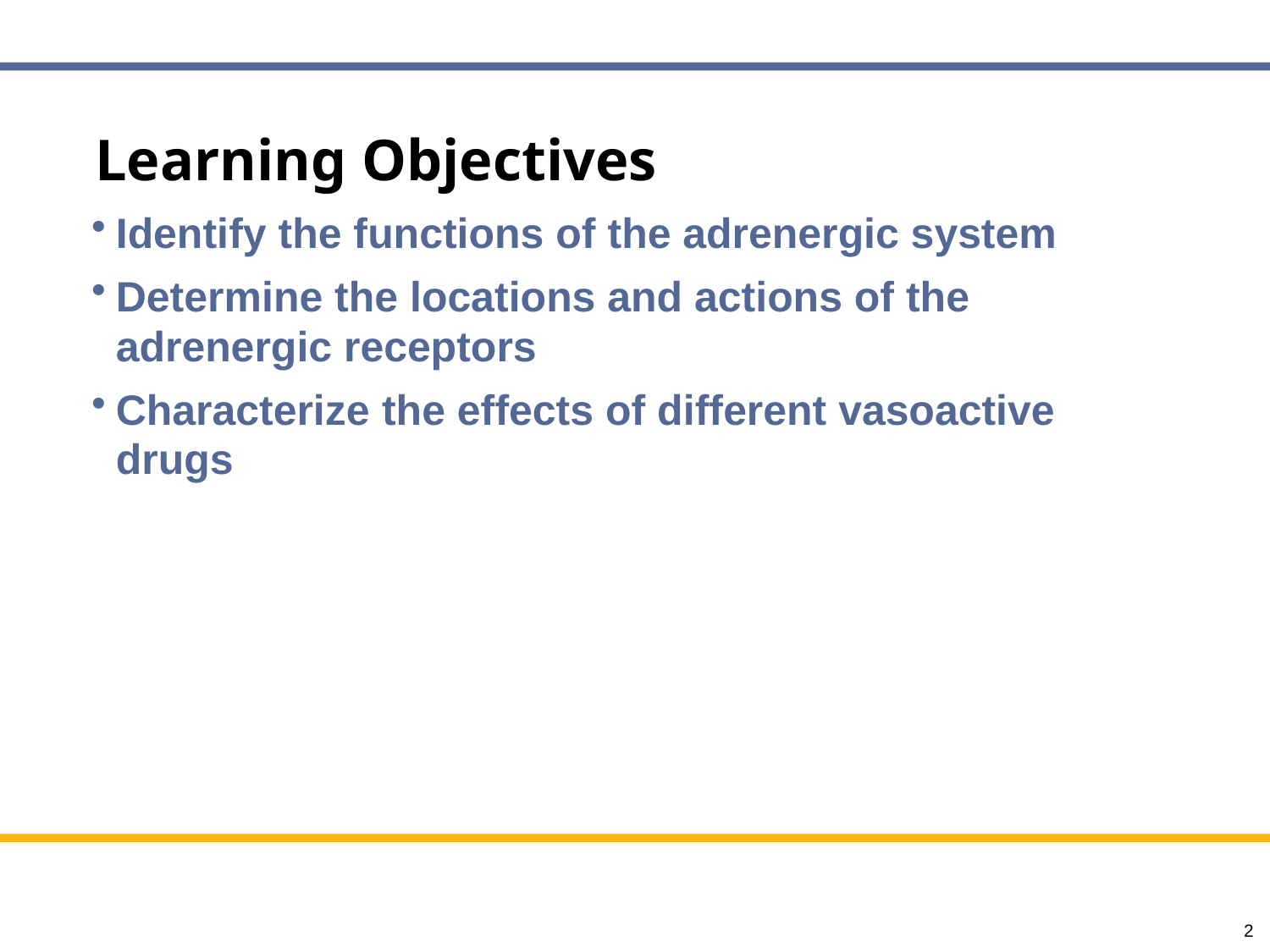

Learning Objectives
Identify the functions of the adrenergic system
Determine the locations and actions of the adrenergic receptors
Characterize the effects of different vasoactive drugs
2

## Slide 3
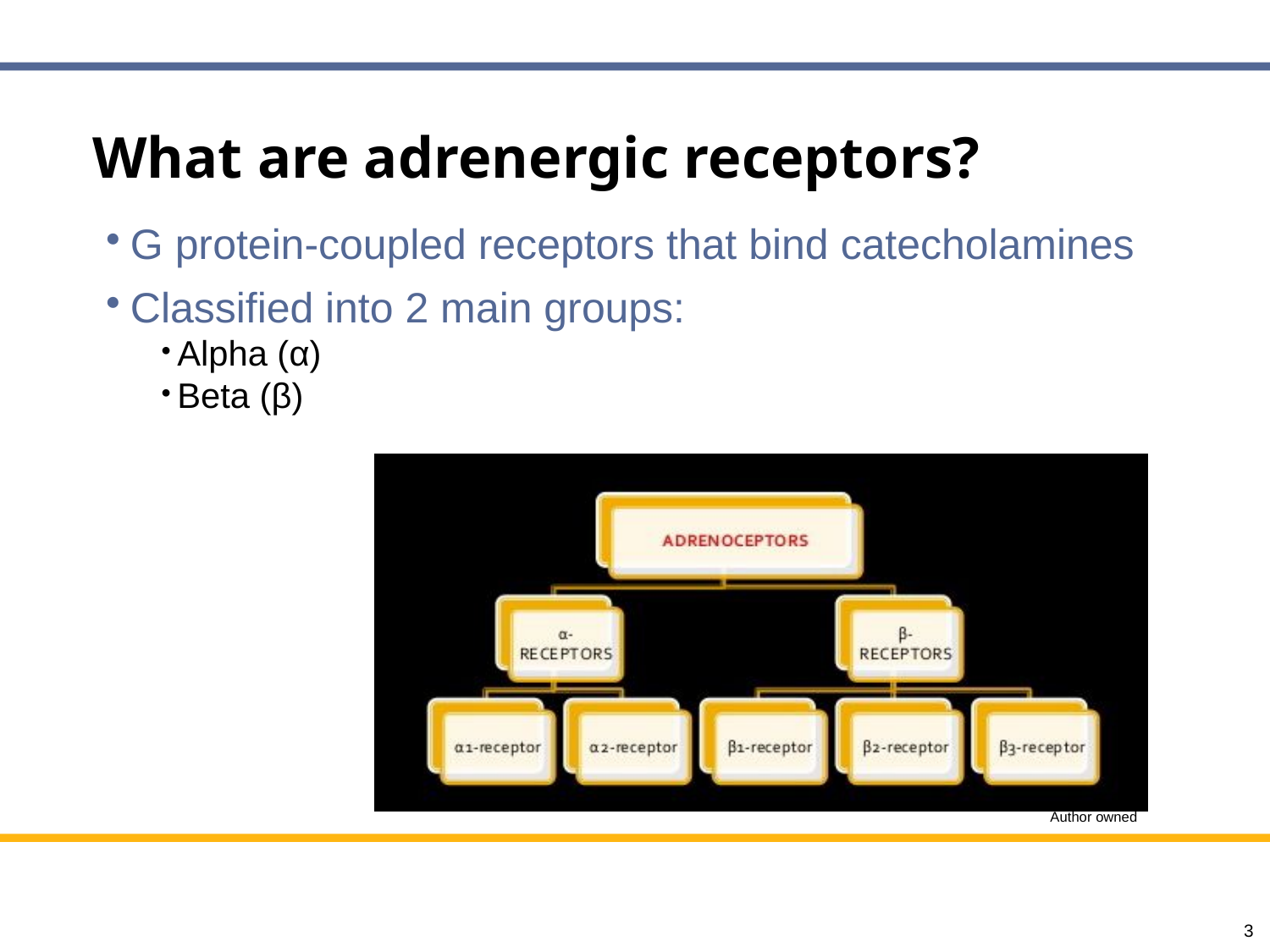

What are adrenergic receptors?
G protein-coupled receptors that bind catecholamines
Classified into 2 main groups:
Alpha (α)
Beta (β)
Author owned
3

## Slide 4
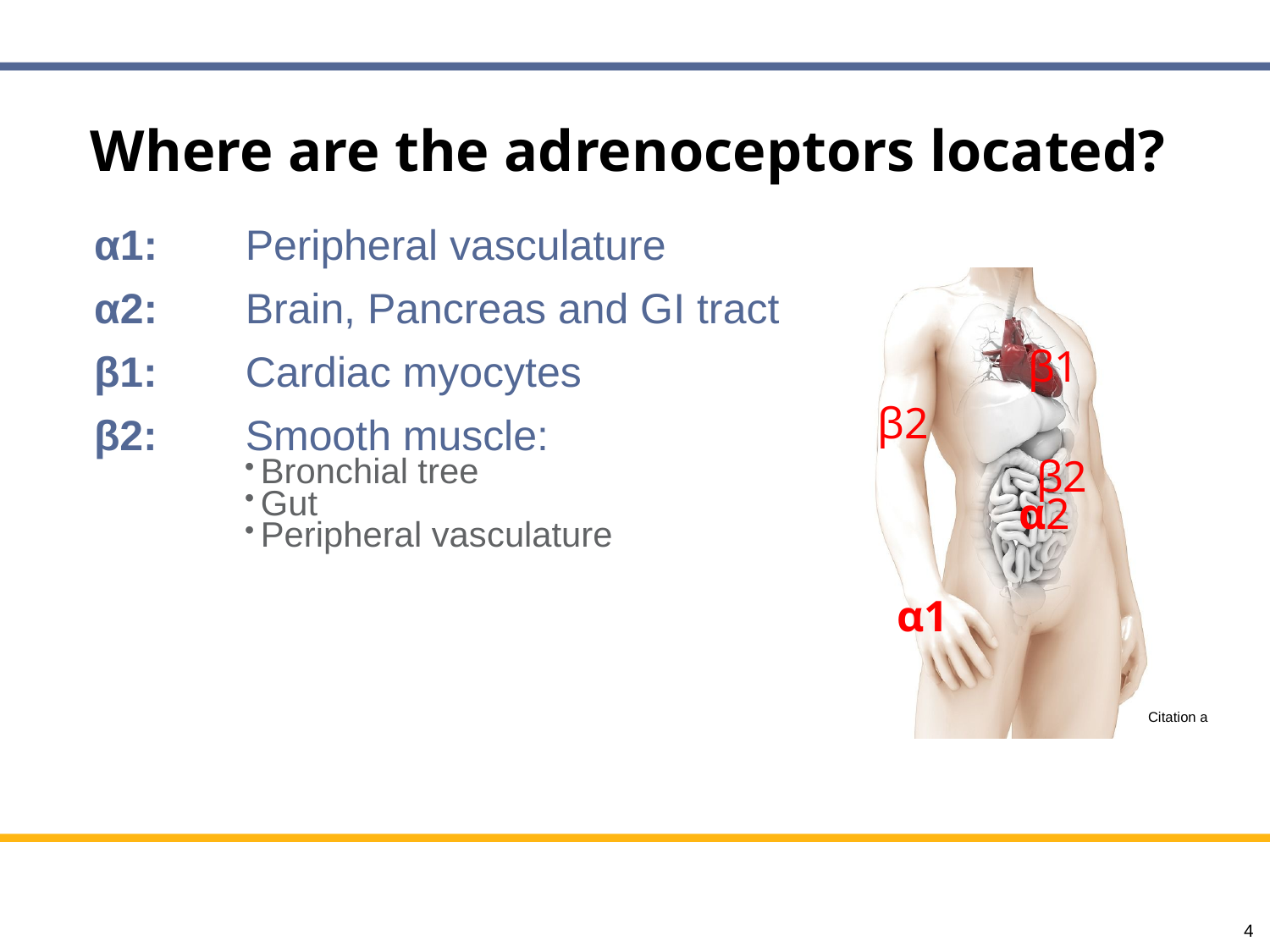

Where are the adrenoceptors located?
α1: 	Peripheral vasculature
α2:	Brain, Pancreas and GI tract
β1: 	Cardiac myocytes
β2:	Smooth muscle:
Bronchial tree
Gut
Peripheral vasculature
β1
β2
β2
α2
α1
Citation a
4

## Slide 5
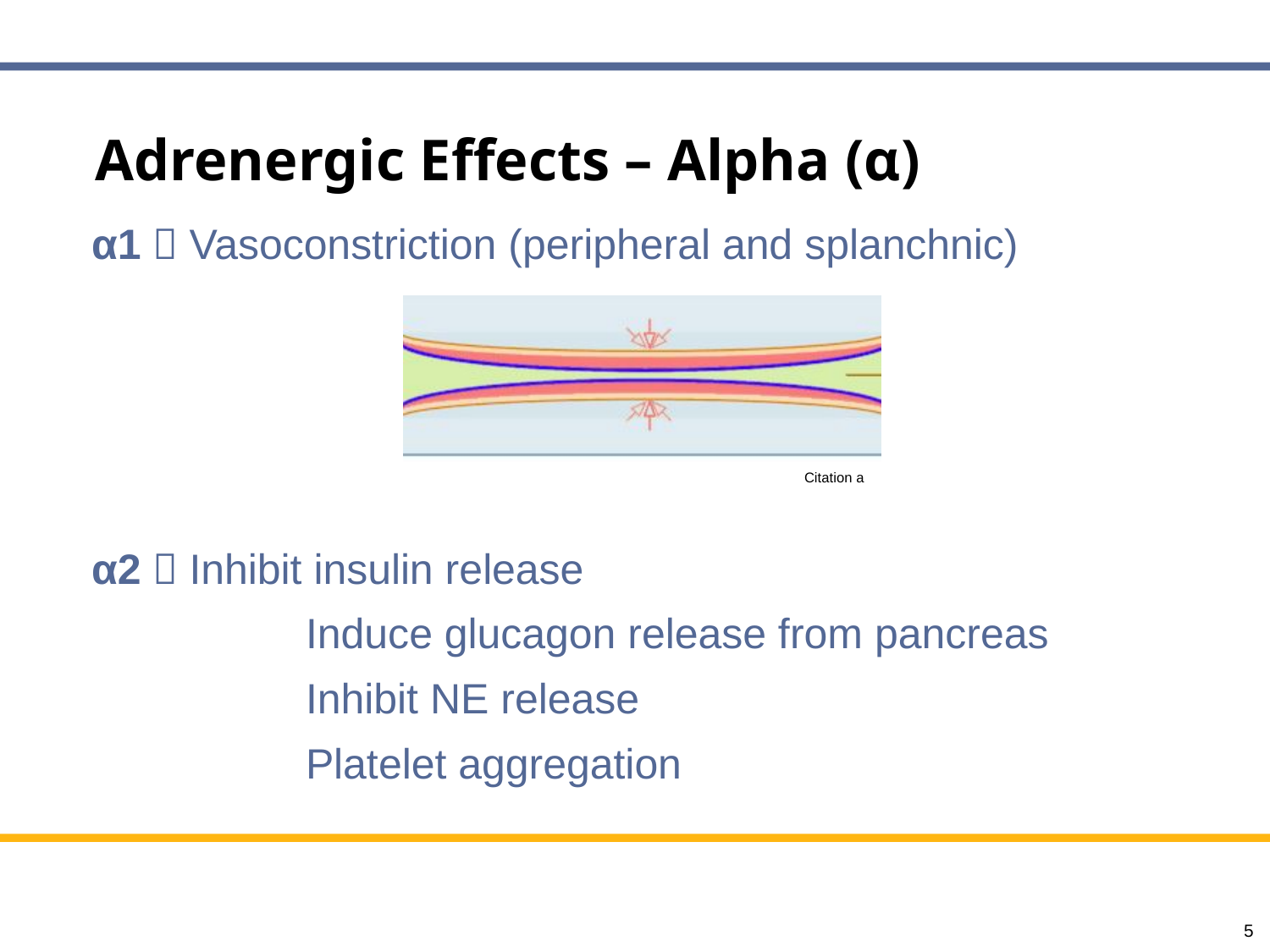

Adrenergic Effects – Alpha (α)
α1  Vasoconstriction (peripheral and splanchnic)
α2  Inhibit insulin release
		 Induce glucagon release from pancreas
		 Inhibit NE release
		 Platelet aggregation
Citation a
5

## Slide 6
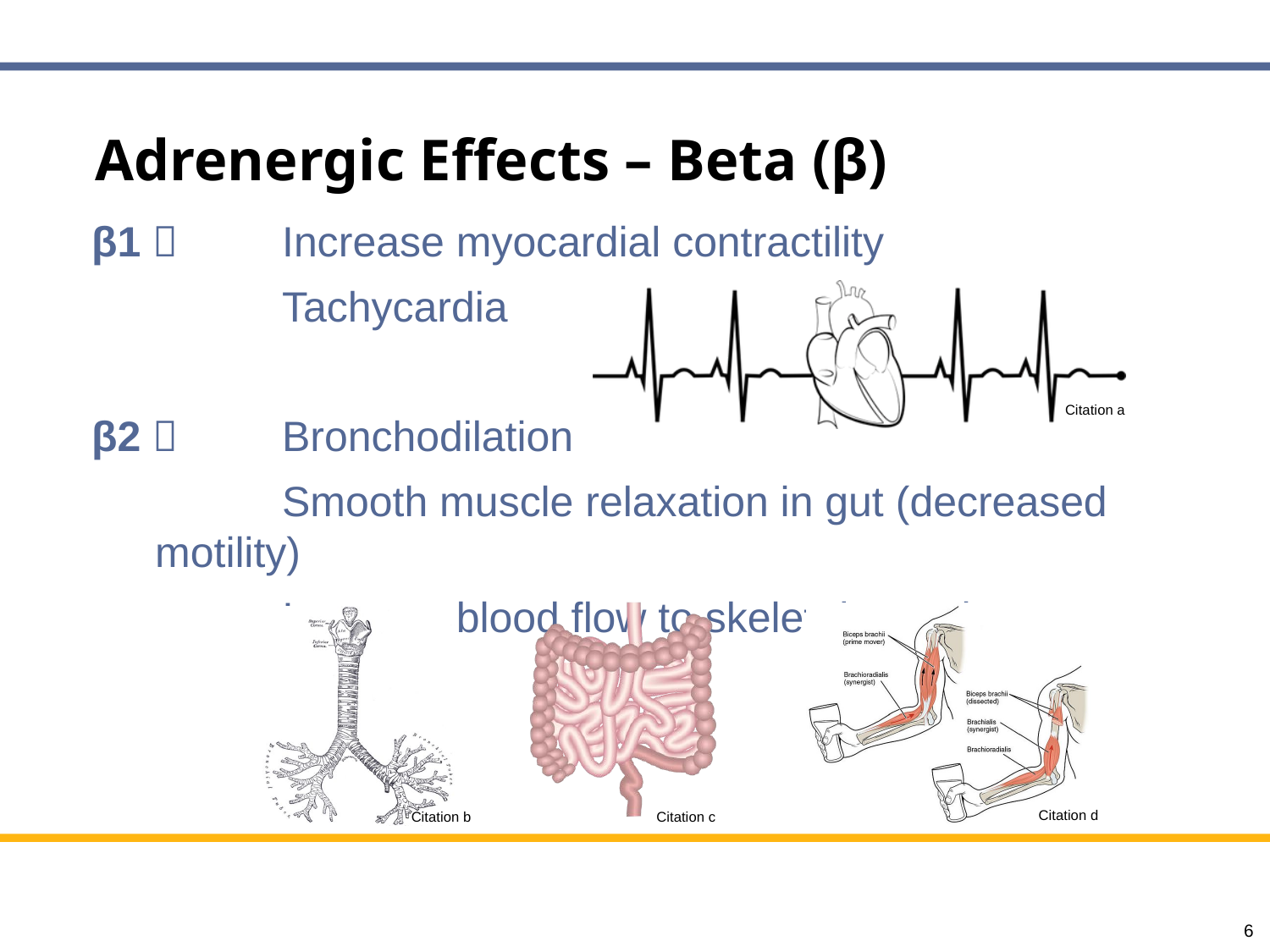

Adrenergic Effects – Beta (β)
β1  	Increase myocardial contractility
		Tachycardia
β2  	Bronchodilation
		Smooth muscle relaxation in gut (decreased motility)
		Increase blood flow to skeletal muscle
Citation a
Citation d
Citation b
Citation c
6

## Slide 7
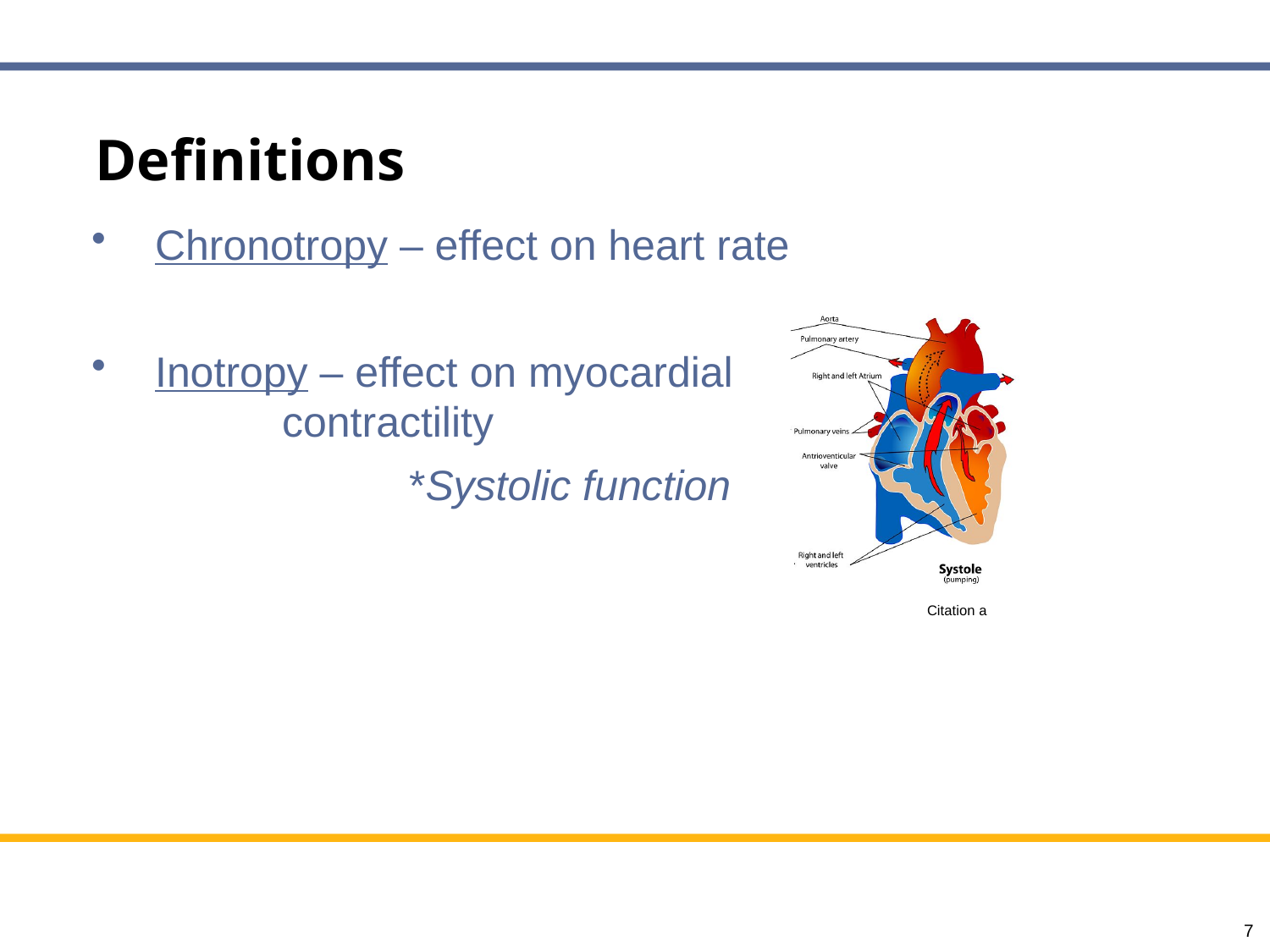

Definitions
Chronotropy – effect on heart rate
Inotropy – effect on myocardial 		contractility
			*Systolic function
Citation a
7

## Slide 8
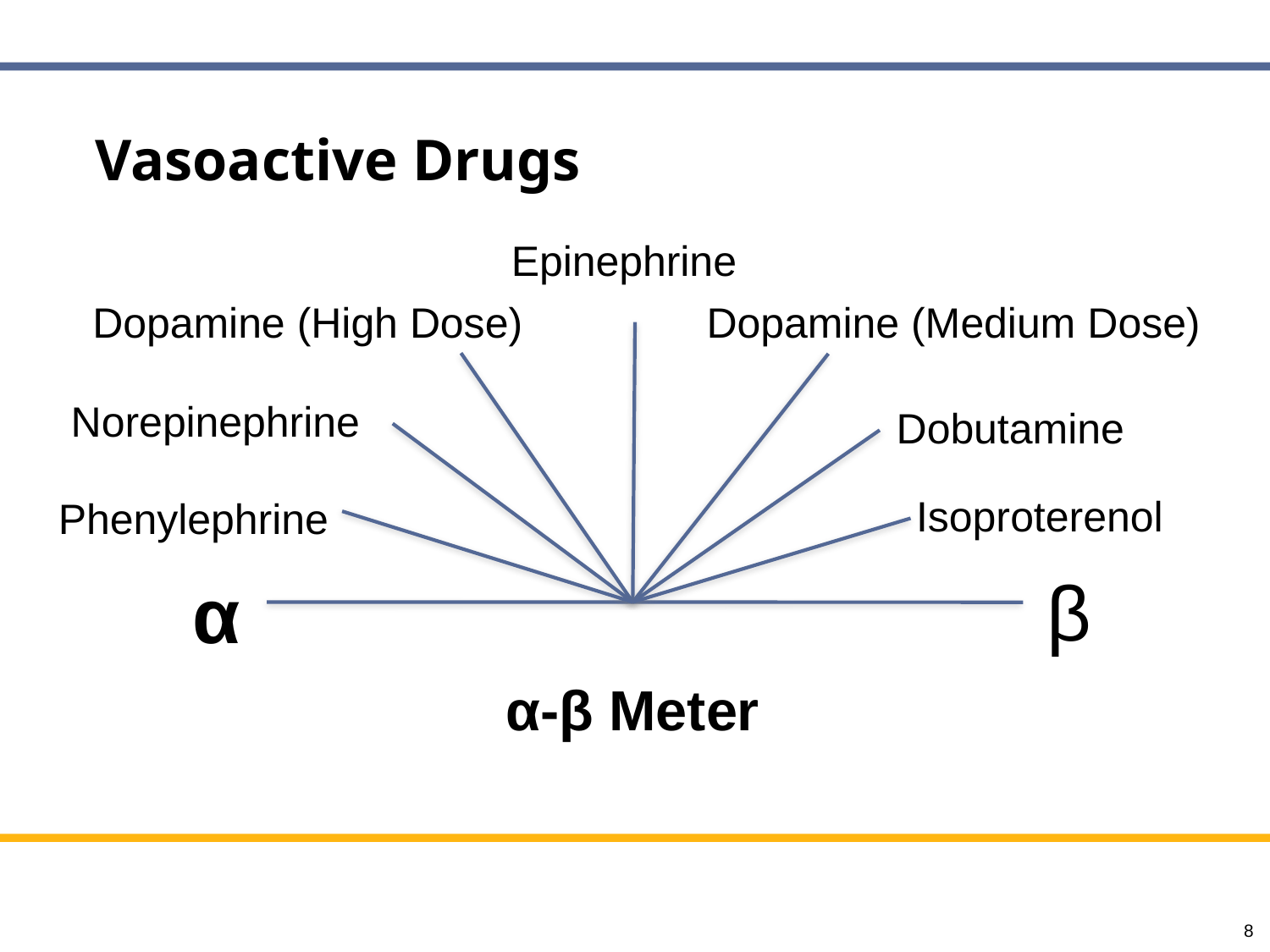

Vasoactive Drugs
Epinephrine
Dopamine (High Dose)
Dopamine (Medium Dose)
Norepinephrine
Dobutamine
Isoproterenol
Phenylephrine
β
α
α-β Meter
8

## Slide 9
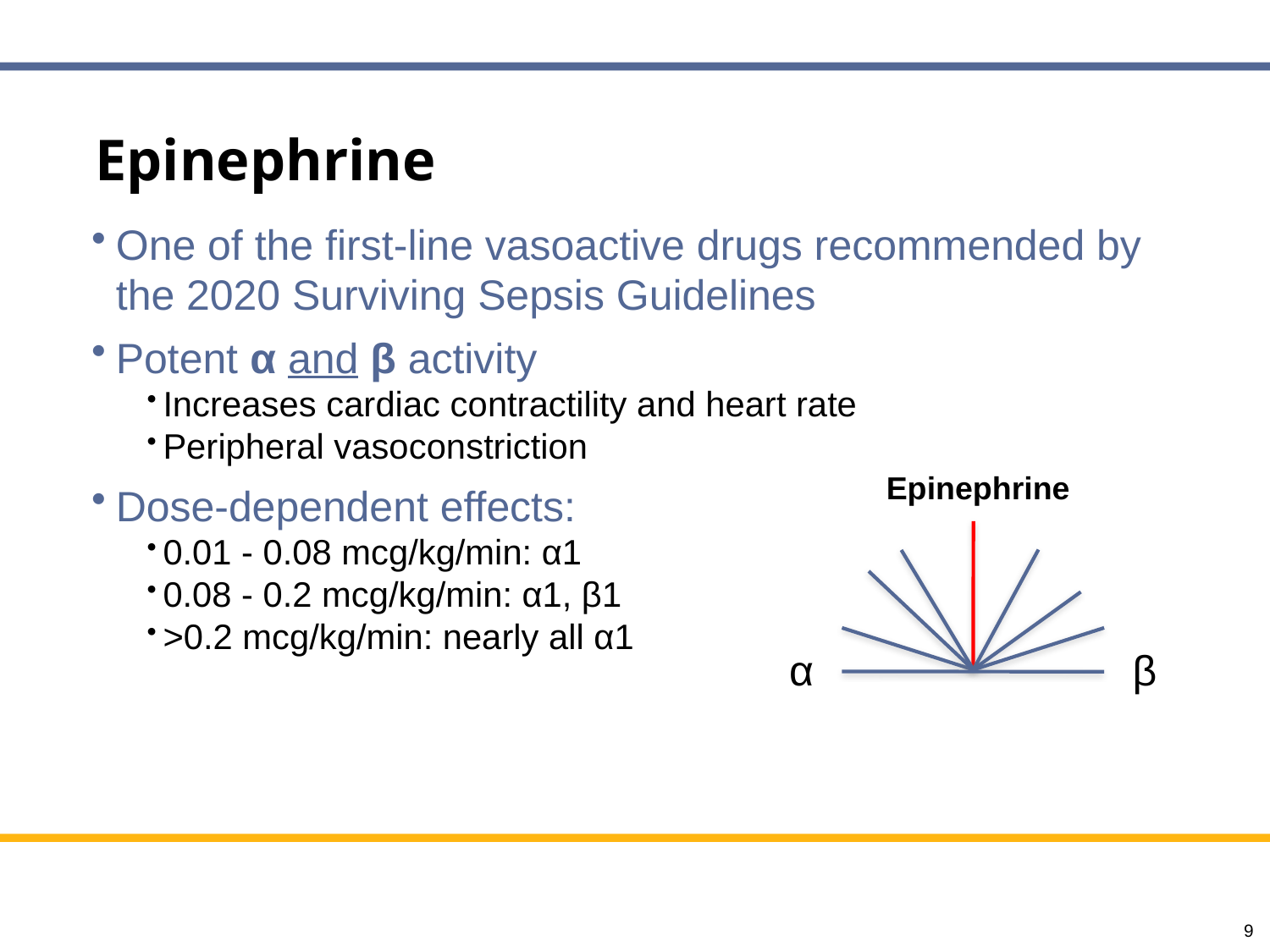

Epinephrine
One of the first-line vasoactive drugs recommended by the 2020 Surviving Sepsis Guidelines
Potent α and β activity
Increases cardiac contractility and heart rate
Peripheral vasoconstriction
Dose-dependent effects:
0.01 - 0.08 mcg/kg/min: α1
0.08 - 0.2 mcg/kg/min: α1, β1
>0.2 mcg/kg/min: nearly all α1
Epinephrine
α β
9

## Slide 10
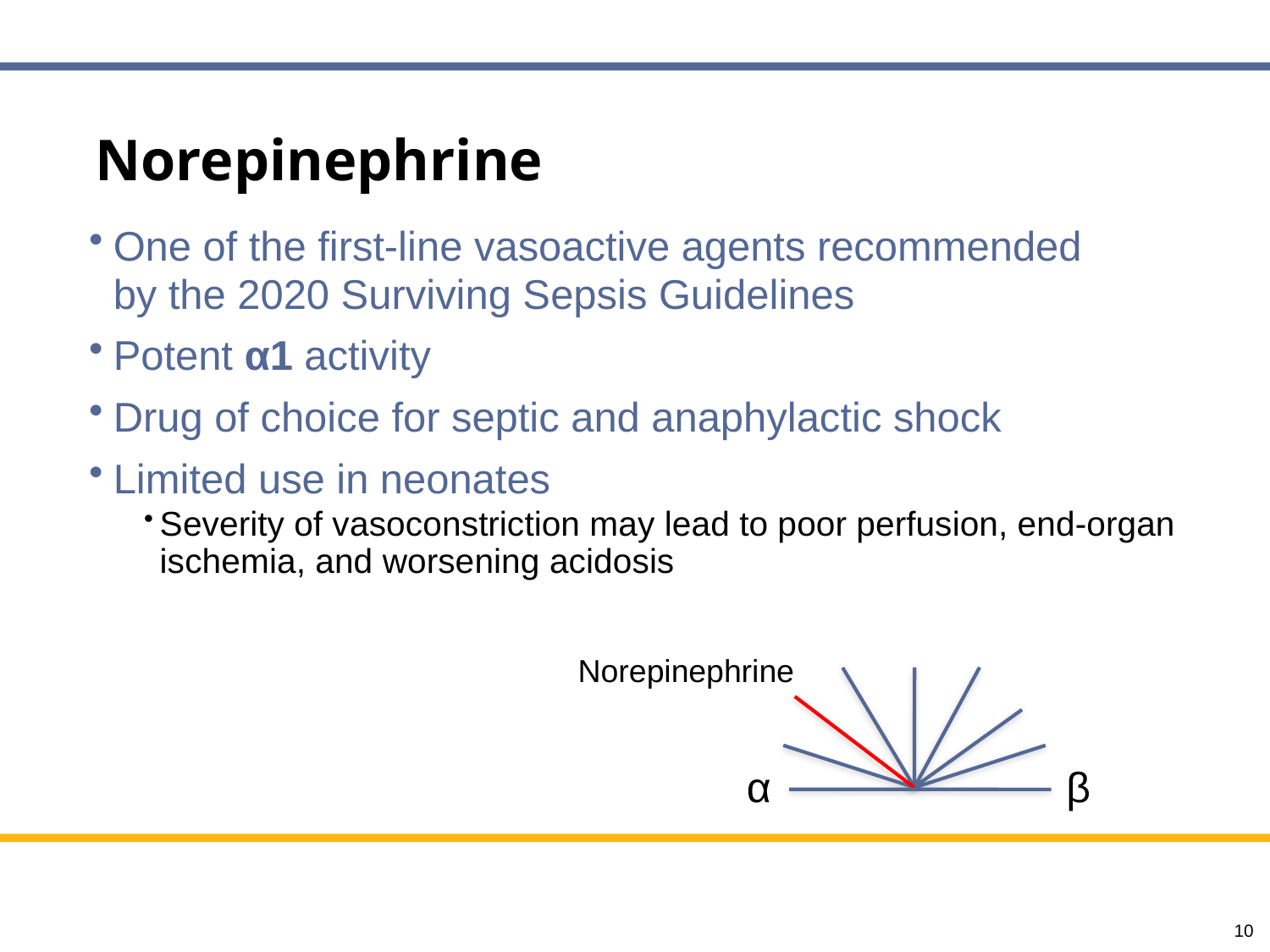

Norepinephrine
One of the first-line vasoactive agents recommended by the 2020 Surviving Sepsis Guidelines
Potent α1 activity
Drug of choice for septic and anaphylactic shock
Limited use in neonates
Severity of vasoconstriction may lead to poor perfusion, end-organ ischemia, and worsening acidosis
Norepinephrine
α β
10

## Slide 11
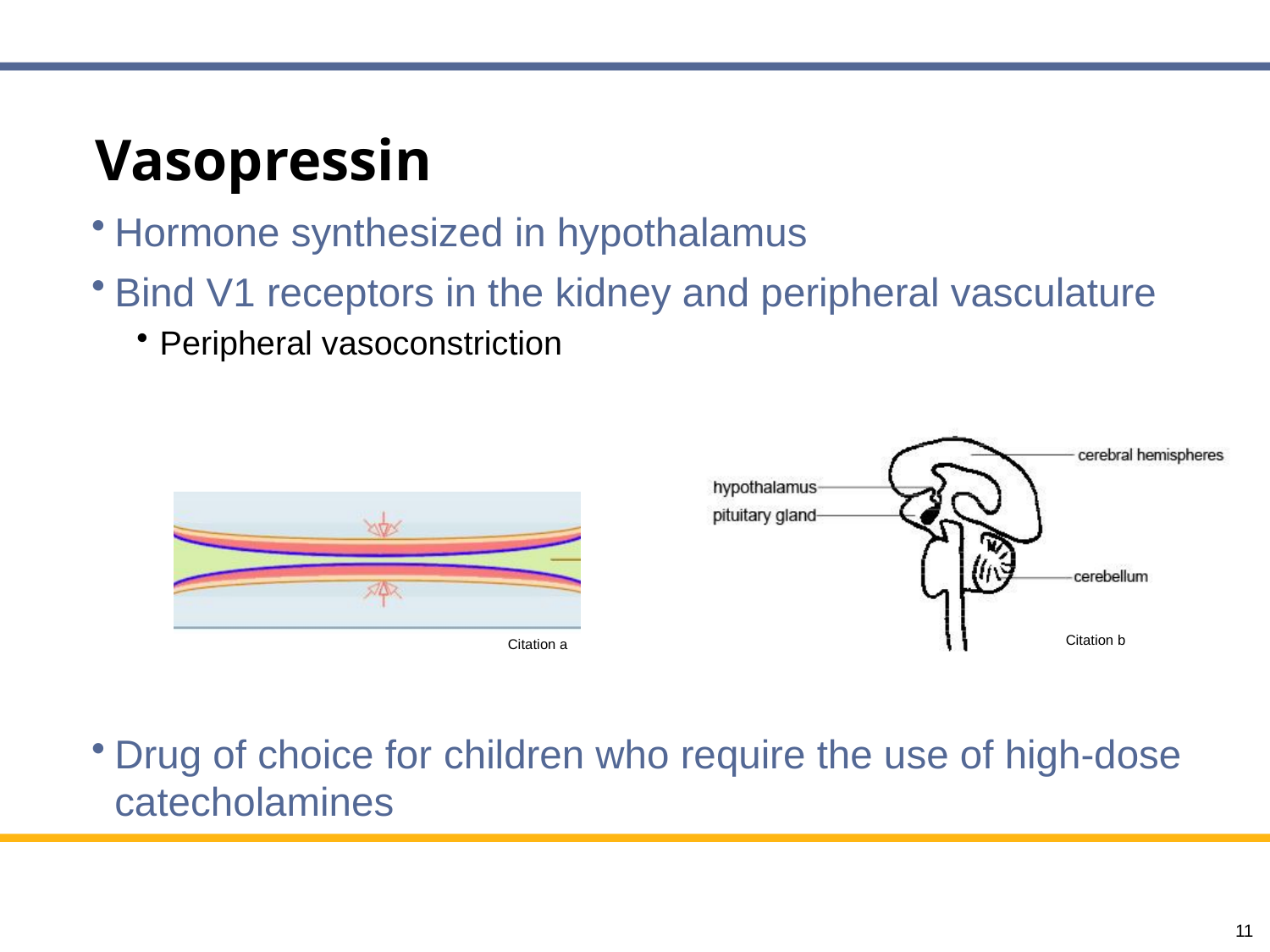

Vasopressin
Hormone synthesized in hypothalamus
Bind V1 receptors in the kidney and peripheral vasculature
Peripheral vasoconstriction
Drug of choice for children who require the use of high-dose catecholamines
Citation b
Citation a
11

## Slide 12
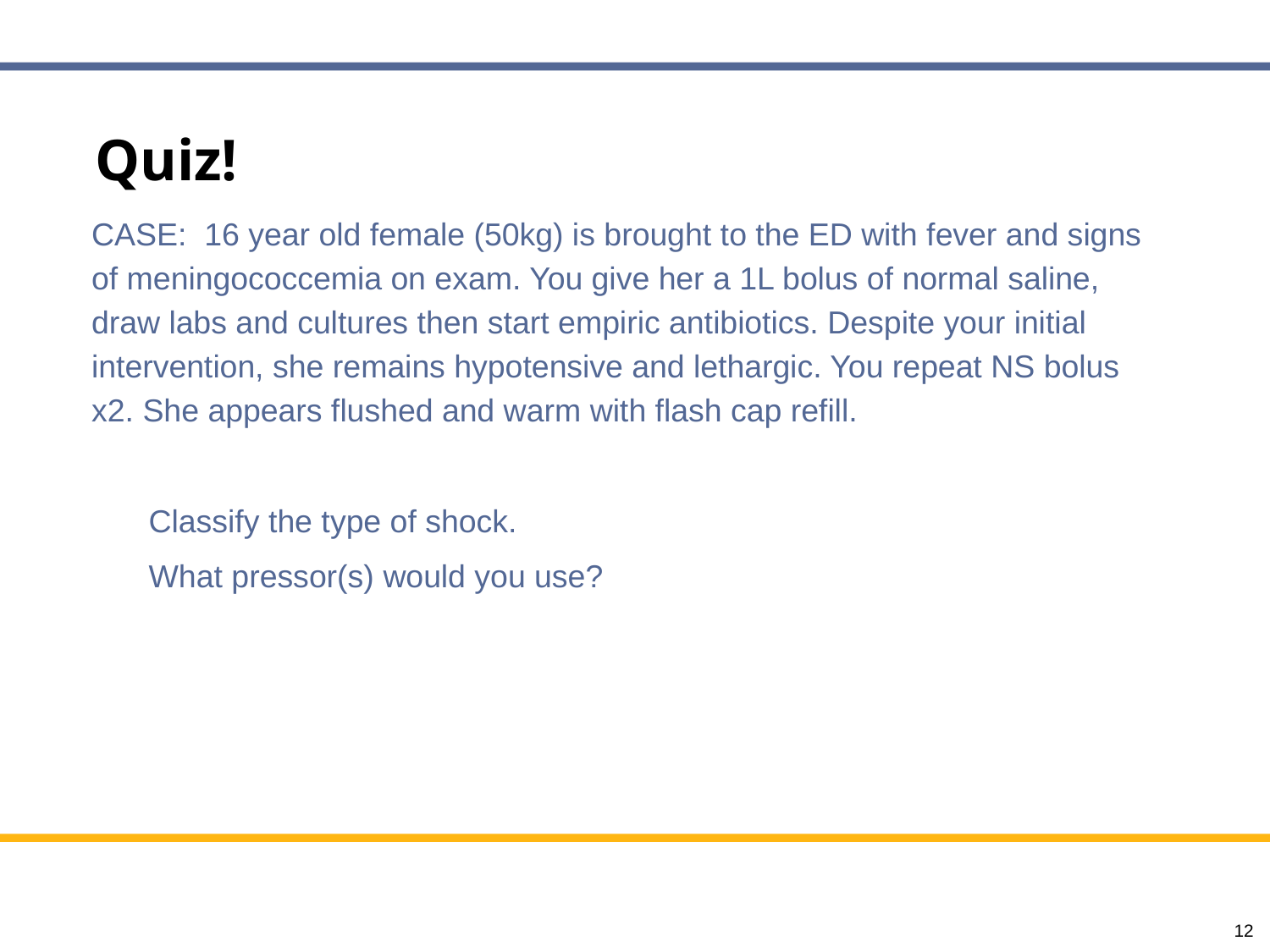

Quiz!
CASE:  16 year old female (50kg) is brought to the ED with fever and signs of meningococcemia on exam. You give her a 1L bolus of normal saline, draw labs and cultures then start empiric antibiotics. Despite your initial intervention, she remains hypotensive and lethargic. You repeat NS bolus x2. She appears flushed and warm with flash cap refill.
	Classify the type of shock.
	What pressor(s) would you use?
12

## Slide 13
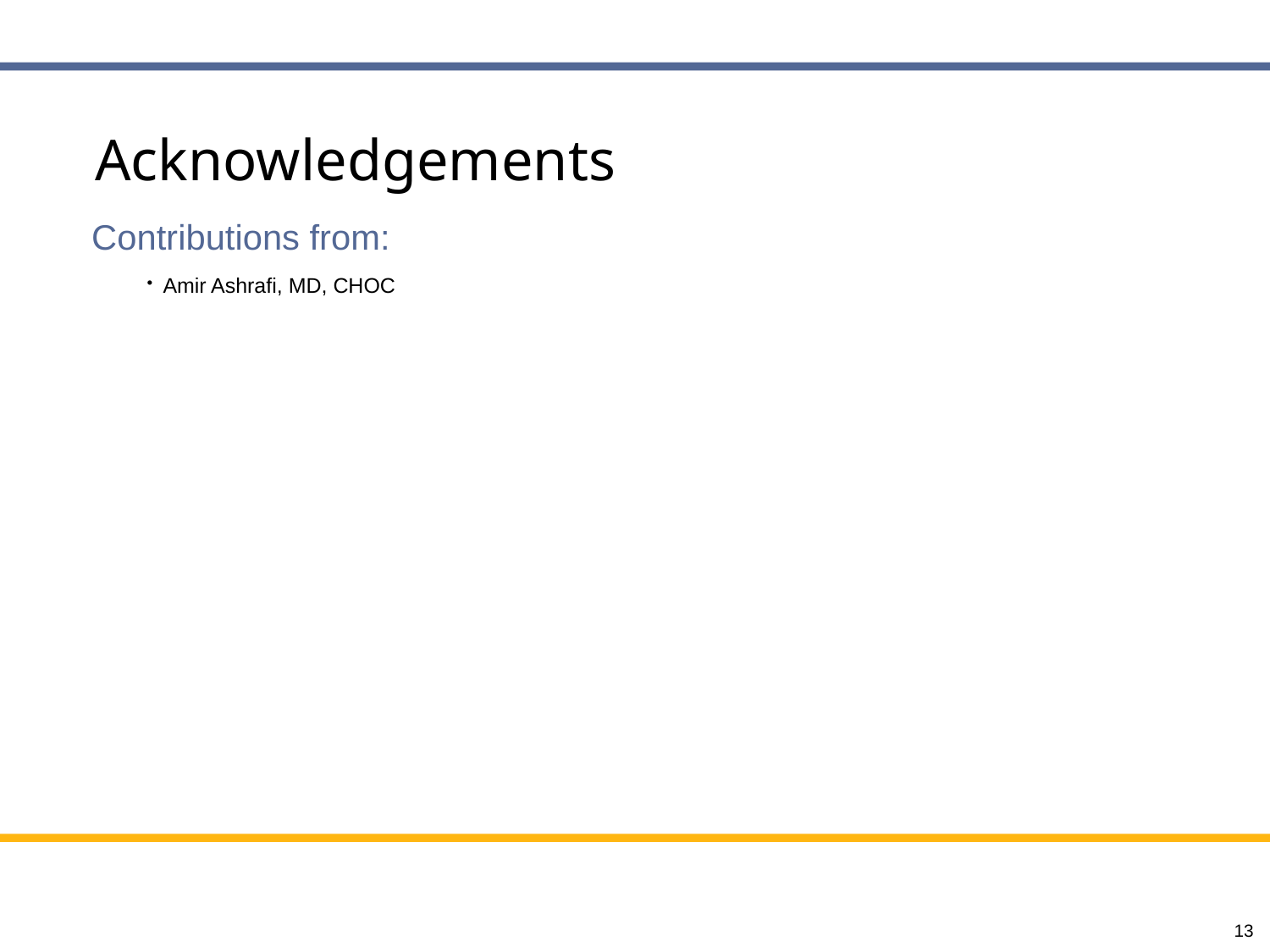

Acknowledgements
Contributions from:
Amir Ashrafi, MD, CHOC
13
