# Supplementary material for: Identification and Management of Pediatric Sepsis: A Medical Student Curricular Supplement for PICU and NICU Rotations
Source: MedEdPORTAL. 2021 Apr 23;17:11142. doi: 10.15766/mep_2374-8265.11142 (PMC8063627; doi:10.15766/mep_2374-8265.11142)
Supplement: Supplementary file 1 — Pre- & Posttest.docxModule 1 - Pediatric Shock.pptxScript 1 - Pediatric Shock.docxModule 2 - Pediatric Sepsis.pptxScript 2 - Pediatric Sepsis.docxModule 3 - Management of Sepsis & Septic Shock.pptxScript 3 - Management of Sepsis & Septic Shock. docxModule 4 - Hemodynamics & Pressor Support.pptxScript 4 - Hemodynamics & Pressor Support.docxSimulation Case 1.docxSimulation Case 2.docxSimulation Case 3.docxPostsimulation Review Quiz.pptx [file mep_2374-8265.11142-s001.zip › I. Script 4 - Hemodyanics & Pressor Support.docx]

**Pressors**

1. This education module continues our Pediatric Sepsis online curriculum. This topic will discuss pressor support and hemodynamics.
2. The learning objectives are:
   - Identify the functions of the adrenergic system
   - Determine the locations and actions of the adrenergic receptors
   - Characterize the effects of different vasoactive drugs
3. In order to discuss pressors, we first need to understand the different types of receptors that they act upon in the body. These are known as adrenergic receptors, and they are G-protein coupled receptors that bind catecholamines. They are classified into 2 groups – alpha and beta.
4. There are further subclassifications of alpha and beta receptors that can be found in different areas of the body. For example, alpha-1 receptors are located primarily in the peripheral vasculature. Alpha-2 receptors are found in the brain, pancreas, and GI tract. Beta-1 receptors are located in cardiac myocytes, and beta-2 receptors in the smooth muscle of the respiratory tree, GI tract, and peripheral blood vessels.
5. Let’s now describe some of the alpha-adrenergic effects. Alpha-1 is associated with vasoconstriction, both peripheral and splanchnic. Alpha-2 inhibits insulin release and induces glucagon release by the pancreas. In the brain, it leads to inhibition of norepinephrine release. It also causes platelet aggregation.
6. For beta-adrenergic effects, beta-1 will increase myocardial contractility and heart rate. Beta-2 will cause bronchodilation, smooth muscle relaxation in the gut (which leads to decreased motility), and increased blood flow to skeletal muscles.
7. So, before we continue, let’s go over some important definitions related to the effects of pressors. You can think of chronotrophy as the timing of the heart rate. For example, is it fast or is it slow? Inotropy refers to the ability of the heart to squeeze, which occurs in systole.
8. Now we’ll discuss specific vasoactive drugs. As a way to help you better understand their effects, we will use the concept of an alpha-beta meter. The meter shows how much each drug acts through alpha and/or beta-adrenergic receptors. Epinephrine has equal alpha and beta effects. Dopamine has different effects based on dose. Norepinephrine and phenylephrine act almost exclusively on alpha-adrenergic receptors, whereas dobutamine and isoproterenol have act almost exclusively on beta receptors.
9. Epinephrine is one of the two first-line vasoactive drugs recommended by current guidelines. Epinephrine has both potent alpha and beta activity, so it can increase cardiac contractility and heart rate as well as cause peripheral vasoconstriction. These effects are dose dependent as shown on the slide.
10. Norepinephrine is the other first-line vasoactive agent for septic shock. Norepinephrine is a potent alpha-1 agonist, and as such, causes peripheral vasoconstriction. This is a drug of choice for septic and anaphylactic shock. Its use, however, is limited in neonates since the severity of vasoconstriction can lead to poor perfusion, ischemia, and worsening acidosis.
11. Similar to norepinephrine, vasopressin is another peripheral vasoconstrictor, BUT it acts via different receptors. As a hormone, it binds vasopressin receptors in the kidney and in the periphery. Its vasoconstrictive effect also makes it a drug of choice for children who require the use of high-dose catecholamines.
12. We finish with a short quiz – There is 1 case here for you to think about in advance, and it will be discussed at your simulation session.
