# Supplementary material for: Identification and Management of Pediatric Sepsis: A Medical Student Curricular Supplement for PICU and NICU Rotations
Source: MedEdPORTAL. 2021 Apr 23;17:11142. doi: 10.15766/mep_2374-8265.11142 (PMC8063627; doi:10.15766/mep_2374-8265.11142)
Supplement: Supplementary file 1 — Pre- & Posttest.docxModule 1 - Pediatric Shock.pptxScript 1 - Pediatric Shock.docxModule 2 - Pediatric Sepsis.pptxScript 2 - Pediatric Sepsis.docxModule 3 - Management of Sepsis & Septic Shock.pptxScript 3 - Management of Sepsis & Septic Shock. docxModule 4 - Hemodynamics & Pressor Support.pptxScript 4 - Hemodynamics & Pressor Support.docxSimulation Case 1.docxSimulation Case 2.docxSimulation Case 3.docxPostsimulation Review Quiz.pptx [file mep_2374-8265.11142-s001.zip › K. Simulation Case 2.docx]

| Appendix K: Simulation Case 2  SIMULATION CASE TITLE: Pediatric Septic Shock, Case 2  AUTHORS: Nicole B. Anderson, MD, Mai-King Chan, MD, Cristina Gutierrez, MD, Valencia Walker, MD  LEARNER AUDIENCE: 4^th^ year medical students | |
| --- | --- |
| PATIENT NAME: Jack  PATIENT AGE: 4 years  CHIEF COMPLAINT: Fever  PHYSICAL SETTING: Emergency Department (ED) | |
|  | |
| Brief narrative description of case | 1. Patient presents to ED with signs and symptoms of septic shock. 2. Students should place him on cardiac monitor and provide supplemental oxygen. 3. Students begin initial resuscitation by establishing IV/IO access and giving fluid boluses. 4. Despite volume resuscitation (total 60cc/kg), patient continues to decompensate. He requires bag-mask ventilation and may ultimately require intubation for respiratory failure. 5. Pressors should be given for fluid-refractory shock. 6. Case ends when shock is reversed with appropriate therapy. |
| Primary Learning Objectives | 1. Make a clinical diagnosis of shock. 2. Administer appropriate treatment for septic shock (fluids, IV antibiotics, vasoactive drugs). 3. Recognize need for rapid volume resuscitation. 4. Initiate broad-spectrum antibiotic therapy early. 5. Follow the Surviving Sepsis Campaign guidelines. 6. Recognize indications for administration of vasoactive drugs. 7. Recognize indications for bag-mask ventilation and/or intubation. |
| Critical Actions | 1. Make a clinical diagnosis of shock. 2. Administer high-flow oxygen. 3. Establish appropriate vascular access. 4. Provide volume resuscitation with IV buffered crystalloid fluid boluses. 5. Send appropriate initial labs (accucheck, CBC, CMP, lactate, blood gas, blood culture, coags, type and cross). 6. Draw blood cultures and initiate early and appropriate broad-spectrum antibiotic therapy. 7. Recognize clinical deterioration in a patient with septic shock. 8. Utilize vasoactive drugs when indicated. 9. Attempt bag-mask ventilation and intubation when indicated. |
| Learner Preparation or Prework | 1. Learners should have viewed the following 4 online educational modules prior to this simulation:    1. Identifying pediatric sepsis (Appendix B)    2. Identifying pediatric shock (Appendix D)    3. Managing pediatric sepsis (Appendix F)    4. Hemodynamics and using vasopressors (Appendix H) 2. Introduction to the simulation staff and what roles they will be playing provided prior to case. 3. Introduction to the simulation room, mannequins, and equipment provided prior to case. |

| Initial Presentation | | | |
| --- | --- | --- | --- |
| Initial vital signs | T 39.1 °C  HR 150  BP 78/39  RR 30  SpO2 88% | | |
| Overall Setting and Appearance | Learners arrive to the ED room to find the patient (mannequin) lethargic and in moderate to severe respiratory distress. Mother and nurse are in the room. | | |
| Confederates (e.g., standardized participants) and their roles in the room at case start | Nurse: Played by simulation staff or other trained staff. Can assist with situational or physical exam clarifications.  “Thank you for coming, doctor. I will be your nurse. Jack is a 4-year-old male who came in for fever. Let me know what medication, equipment, or tests you need. This is Jack’s mother.”  Mother: Played by simulation staff or other trained staff. Provides answers to historical questions from learners.  “Hi doctor. Thank you for coming to see my son.” | | |
| HPI | Information obtained upon questioning:  Patient is a 4yo male. Mother reports fever x2 days and cough productive of yellow sputum. She measured an oral temp of 103.5 at home. Today he seemed to be breathing faster and seems very sleepy. | | |
| Past Medical/Surgical History | Medications | Allergies | Family History |
| Previously healthy  Vaccines are up to date | None | No known drug allergies | None significant to presenting complaint |
| Physical Examination | | | |
| General | Appears lethargic. In moderate to severe respiratory distress. | | |
| HEENT | Nares clear. Oropharynx clear without edema, erythema or exudates. No cervical lymphadenopathy. | | |
| Neck | Normal range of motion. No nuchal rigidity. | | |
| Lungs | Increased work of breathing with intercostal retractions and tachypnea. Crackles heard at right base. Rest of lung sounds clear. | | |
| Cardiovascular | Tachycardic, regular rhythm, no murmur. Peripheral pulses 1+. | | |
| Abdomen | Soft, non-distended, non-tender. No masses or hepatosplenomegaly. | | |
| Neurological | Pupils equal and reactive to light. Lethargic but opens eyes to voice and oriented. Able to converse. Follows commands for movement. No gross focal deficits. | | |
| Skin | Warm and flushed. | | |
| GU | Normal male genitalia. | | |
| Psychiatric | No psychomotor agitation. | | |

| Instructor Notes - Changes and Case Branch Points |
| --- |

| Event Name | Patient Vitals | Instructor/Operator Cues | Observable Actions |
| --- | --- | --- | --- |
| State 1:  Baseline/Initial Presentation | T 39.1 °C  HR 150  BP 78/39  RR 30  SpO2 88%  Exam findings:  Appears lethargic. Increased WOB with intercostal retractions, tachypnea, and crackles heard at right base. Peripheral pulses 1+. Skin warm and flushed.  Labs:  Na 134, K 4.0, Cl 98, CO2 17, BUN 25, Cr 0.9  ABG: 7.28/30/60/18  Lactate 21  CBC: 22>12.5/37<400 | HPI obtained.  Operator:  Level of alertness decreases despite interventions. SpO2 can increase to 93% if supplemental O2 is started. Peripheral pulses remain weak.  This state lasts 5-10 minutes. Provide clinical prompts if needed. | 1. Take an appropriate history.  2. Make a clinical diagnosis of septic shock.  3. Administer high flow oxygen.  4. Establish IV/IO access (at least 2 access points).  5. Push buffered crystalloid boluses 10-20cc/kg and repeat until 60cc/kg total given if no response.  6. Draw appropriate initial labs: CBC, CMP, lactate, blood gas, blood cultures, coags, type and cross.  7. Initiate early and appropriate antibiotic therapy with Ceftriaxone 80-100mg/kg or other broad-spectrum antibiotic(s). |
| State 2: Initial  Decompensation | HR 145  BP 80/40  RR 38  SpO2 92%  Exam findings:  Obtunded. Increased WOB with intercostal and suprasternal retractions. Distal pulses weak and thready. Skin warm and flushed. | Patient is in septic shock and does not respond to initial fluid boluses. Mental status continues to deteriorate.  Operator:  Despite all interventions, the patient and his vital signs do not improve. Work of breathing increases despite NC or facemask O2, and SpO2 declines below 90%.  This state lasts 5 minutes Provide clinical prompts if needed. | 1. Recognize clinical deterioration in a patient with septic shock.  2. If not already done, push buffered crystalloid fluid boluses for total 60cc/kg.  3. Attempt bag-mask ventilation when indicated and prepare for intubation. |
| State 3:  Worsening decompensation | HR 162  BP 62/32  RR 50  SpO2 85%  Exam findings:  Obtunded. Worsening respiratory distress. Peripheral pulses thready. Extremities appear mottled. | Patient develops signs of worsening perfusion and respiratory failure.  Operator:  SpO2 can increase to 90% if bag-mask ventilation is provided.  If patient is not intubated, he progresses further into respiratory failure and death.  This state lasts 5 minutes. Provide clinical prompts if needed. | 1. Recognize clinical deterioration in a patient with septic shock.  2. Attempt intubation.  3. Optional teaching/discussion point on immediate intervention for obtunded patient versus decision to perform rapid sequence intubation. |
| State 4:  On ventilator with progressive shock | HR 170  BP 64/32  RR per vent settings  SpO2 95%  Exam findings:  Comatose, on ventilator. Peripheral pulses thready. Extremities mottled. | Patient has fluid-refractory shock and develops signs of worsening septicemia.  Operator:  If pressors are not started, patient becomes more hypotensive and progresses to irreversible clinical deterioration.  Provide clinical prompts if needed.  If student(s) fails to appropriately intervene (reversal of shock via administration of vasoactive drugs), terminate clinical scenario when criteria for irreversible clinical deterioration is met. In consideration of psychological safety for the learner, the simulation should be terminated at this point, rather than at patient death. | 1. Administer vasoactive drugs: Peripheral epinephrine or norepinephrine 0.1mcg/kg/min. |
| State 5:  Resolution | HR 110  BP 88/51  RR per vent settings  SpO2 97%  Exam findings:  Patient moaning. Peripheral pulses strong. Extremities warm. | Shock is reversed after appropriate fluid resuscitation (60cc/kg) and pressor therapy.  Operator:  BP and HR begin to normalize after starting pressors. Patient is stable on ventilator and epinephrine/norepi gtt.  End scenario. |  |

Ideal Scenario Flow

The learners enter the room to find a patient in respiratory distress. They immediately place the patient on bedside monitors and recognize that the patient is hypoxic and in septic shock with a fever, tachycardia, and hypotension. Supplemental oxygen is provided, IV access is obtained, and an IV fluid bolus is ordered. A septic workup is sent, including blood culture, and IV antibiotics are ordered. Despite interventions, the patient’s mental and respiratory statuses decline. Learners initiate bag-mask ventilation and prepare for intubation. Intubation is then attempted and successful with post-intubation SpO2 stable at 90%. A total of 60 cc/kg of crystalloid fluid is given, however the patient’s shock does not respond to fluid boluses. Blood pressure continues to drop, extremities become mottled and cool, and peripheral pulses are weak and thready. Learners order vasoactive medication, which reverses the patient’s state of shock. Patient is stable on mechanical ventilation and pressor therapy. Transfer to the pediatric intensive care unit is initiated.

Anticipated Management Mistakes

1. Failure to bag-mask ventilate: Many of our learners recognized the need for intubation, however we found that most did not provide bag-mask ventilation while preparing to intubate or in between intubation attempts, leading to further respiratory decline. We found it helpful to bring up this learning point during debriefing after the first simulation case. Almost all learners then recognized the need for bag-mask ventilation in the second and third simulation cases.
2. Failure to provide a total of 60 cc/kg of fluid resuscitation prior to initiation of pressor support.: Many of our learners failed to give a total of three 20 cc/kg boluses prior to initiating pressor therapy. We discussed appropriate fluid resuscitation and the definition of fluid refractory shock in our debriefing sessions, with improvement in performance on subsequent simulations.
3. Failure to recognize sepsis, draw blood cultures, and start antibiotics in a timely manner. Many of our learners would get very focused on the patient’s other concerning vital signs, and not recognize or understand the implication of the fever, leading to a delay in obtaining a blood culture and starting antibiotic therapy. Again, this was discussed in our debriefing sessions, with improvement in performance on subsequent simulations.
