# Supplementary material for: Identification and Management of Pediatric Sepsis: A Medical Student Curricular Supplement for PICU and NICU Rotations
Source: MedEdPORTAL. 2021 Apr 23;17:11142. doi: 10.15766/mep_2374-8265.11142 (PMC8063627; doi:10.15766/mep_2374-8265.11142)
Supplement: Supplementary file 1 — Pre- & Posttest.docxModule 1 - Pediatric Shock.pptxScript 1 - Pediatric Shock.docxModule 2 - Pediatric Sepsis.pptxScript 2 - Pediatric Sepsis.docxModule 3 - Management of Sepsis & Septic Shock.pptxScript 3 - Management of Sepsis & Septic Shock. docxModule 4 - Hemodynamics & Pressor Support.pptxScript 4 - Hemodynamics & Pressor Support.docxSimulation Case 1.docxSimulation Case 2.docxSimulation Case 3.docxPostsimulation Review Quiz.pptx [file mep_2374-8265.11142-s001.zip › L. Simulation Case 3.docx]

| Appendix L: Simulation Case 3  SIMULATION CASE TITLE: Pediatric Septic Shock, Case 3  AUTHORS: Nicole B. Anderson, MD, Mai-King Chan, MD, Cristina Gutierrez, MD, Valencia Walker, MD  LEARNER AUDIENCE: 4^th^ year medical students | |
| --- | --- |
| PATIENT NAME: Johnny  PATIENT AGE: 6 months  CHIEF COMPLAINT: Fever  PHYSICAL SETTING: Emergency Department (ED) | |
|  | |
| Brief narrative description of case | 1. Patient presents to ED with signs and symptoms of septic shock. 2. Students should place him on cardiac monitor and provide supplemental oxygen. 3. Students begin initial resuscitation by establishing IV/IO access and giving fluid boluses. 4. Despite volume resuscitation (total 60cc/kg), patient continues to decompensate. He requires bag-mask ventilation and ultimately intubation for respiratory failure. 5. Pressors should be given for fluid-refractory shock. 6. Case ends when shock is reversed with appropriate therapy. |
| Primary Learning Objectives | 1. Make a clinical diagnosis of shock. 2. Administer appropriate treatment for septic shock (fluids, IV antibiotics, vasoactive drugs). 3. Recognize need for rapid volume resuscitation. 4. Initiate broad-spectrum antibiotic therapy early. 5. Follow the Surviving Sepsis Campaign guidelines. 6. Recognize indications for administration of vasoactive drugs. 7. Recognize indications for bag-mask ventilation and/or intubation. |
| Critical Actions | 1. Make a clinical diagnosis of shock. 2. Administer high-flow oxygen. 3. Establish appropriate vascular access. 4. Provide volume resuscitation with IV buffered crystalloid fluid boluses. 5. Send appropriate initial labs (accucheck, CBC, CMP, lactate, blood gas, blood culture, coags, type and cross). 6. Draw blood cultures and initiate early and appropriate broad-spectrum antibiotic therapy. 7. Recognize clinical deterioration in a patient with septic shock. 8. Utilize vasoactive drugs when indicated. 9. Attempt bag-mask ventilation and intubation when indicated. |
| Learner Preparation or Prework | 1. Learners should have viewed the following 4 online educational modules prior to this simulation:    1. Pediatric shock (Appendix B)    2. Pediatric sepsis (Appendix D)    3. Managing pediatric sepsis and septic shock (Appendix F)    4. Hemodynamics and pressor support (Appendix H) 2. Introduction to the simulation staff and what roles they will be playing provided prior to case. 3. Introduction to the simulation room, mannequins, and equipment provided prior to case. |

| Initial Presentation | | | |
| --- | --- | --- | --- |
| Initial vital signs | T 40.0 °C  HR 184  BP 69/38  RR 40  SpO2 92% | | |
| Overall Setting and Appearance | Learners arrive to the ED room to find the patient (mannequin) lethargic. Mother and nurse are in the room. | | |
| Confederates (e.g., standardized participants) and their roles in the room at case start | Nurse: Played by simulation staff or other trained staff. Can assist with situational or physical exam clarifications.  “Thank you for coming, doctor. I will be your nurse. Johnny is a 6-month-old male who came in for fever. Let me know what medication, equipment, or tests you need. This is Johnny’s mother.”  Mother: Played by simulation staff or other trained staff. Provides answers to historical questions from learners.  “Hi doctor. Thank you for coming to see my son.” | | |
| HPI | Patient is a 6mo male. Mother says baby felt warm yesterday, and today she measured a rectal temp of 102.5 °F. Baby has been very sleepy and is not even waking to feed. Only had 1 wet diaper today. | | |
| Past Medical/Surgical History | Medications | Allergies | Family History |
| Previously healthy  Unvaccinated | Poly-Vi-Sol | No known drug allergies | None significant to presenting complaint |
| Physical Examination | | | |
| General | Lethargic infant. Weak cry. | | |
| HEENT | Not producing tears. Nares and oropharynx clear. | | |
| Neck | Head lag present. No deformity. | | |
| Lungs | Lungs clear to auscultation in all fields bilaterally. | | |
| Cardiovascular | Tachycardic with II/VI systolic murmur. Weak distal pulses. | | |
| Abdomen | Soft, non-distended, non-tender. No masses or hepatosplenomegaly. | | |
| Neurological | Eyes opening spontaneously. Pupils equal and reactive to light. Decreased tone throughout. Infant is irritable and continually cries. Infant withdraws from pain, not from touch. No obvious focal deficits. | | |
| Skin | Warm and flushed. | | |
| GU | Normal male genitalia. | | |

| Instructor Notes - Changes and CASE Branch Points |
| --- |

| Event Name | Patient Vitals | Instructor/Operator Cues | Observable Actions |
| --- | --- | --- | --- |
| State 1:  Baseline/Initial Presentation | T 40.0 °C  HR 184  BP 69/38  RR 40  SpO2 92%  Exam findings:  Weak cry, no tears. Decreased tone throughout. Tachycardic with II/VI systolic murmur. Weak distal pulses. Skin feels warm and flushed.  Labs:  Accucheck 90  Na 144, K 4.0, Cl 101, CO2 15, BUN 30, Cr 0.8  ABG: 7.18/42/68/13  Lactate 30  CBC: 3>11/33<390 | HPI obtained.  Operator:  Vital signs and exam not improved despite interventions. SpO2 can increase to 96% if supplemental O2 is started. Peripheral pulses remain weak and thready.  This state lasts 5-10 minutes Provide clinical prompts if needed. | 1. Take an appropriate history.  2. Make a clinical diagnosis of septic shock.  3. Administer high flow oxygen.  4. Establish IV/IO access (at least 2 access points).  5. Push buffered crystalloid boluses 10-20cc/kg and repeat until 60cc/kg total given if no response.  6. Draw appropriate initial labs: CBC, CMP, lactate, blood gas, blood cultures, coags, type and cross.  7. Initiate early and appropriate antibiotic therapy with Ceftriaxone 80-100mg/kg or other broad-spectrum antibiotic(s). **Opportunity to discuss antibiotic choices if this patient were a neonate (<30 days of age) during feedback session.* |
| State 2: Initial  Decompensation | HR 178  BP 70/39  RR 44  SpO2 93%  Exam findings:  No cry. Minimally responsive. Extremities appear mottled. | Patient is in septic shock and does not respond to initial fluid boluses. Level of consciousness continues to decline.  Operator:  Despite all interventions, the patient and his vital signs do not improve. As he becomes less responsive, his respiratory rate declines to 15.  This state lasts 5 minutes. Provide clinical prompts if needed. | 1. Recognize clinical deterioration in a patient with septic shock.  2. If not already done, push buffered crystalloid fluid boluses for total 60cc/kg.  3. Attempt bag-mask ventilation when indicated and prepare for intubation. |
| State 3:  Worsening decompensation | HR 189  BP 65/30  RR 15  SpO2 82%  Exam findings:  No spontaneous movement. Respirations slow and shallow. Peripheral pulses thready. Mottling of extremities and trunk. | Patient develops signs of worsening perfusion and respiratory failure.  Operator:  SpO2 can increase to 95% if bag-mask ventilation is provided.  If patient is not intubated, he progresses further into respiratory failure and death.  This state lasts 5 minutes. Provide clinical prompts if needed. | 1. Recognize clinical deterioration in a patient with septic shock.  2. Attempt intubation.  3. Optional teaching/discussion point on immediate intervention for obtunded patient versus decision to perform rapid sequence intubation. |
| State 4:  On ventilator with progressive shock | HR 188  BP 57/26  RR per vent settings  SpO2 96%  Exam findings:  Minimally responsive, on ventilator. Thready peripheral pulses. Extremities mottled and cool. | Patient has fluid-refractory shock and develops signs of worsening septicemia.  Operator:  If pressors are not started, patient becomes more hypotensive and progresses to irreversible clinical deterioration.  Provide clinical prompts if needed.  If student(s) fails to appropriately intervene (reversal of shock via administration of vasoactive drugs), terminate clinical scenario when criteria for irreversible clinical deterioration is met. In consideration of psychological safety for the learner, the simulation should be terminated at this point, rather than at patient death. | 1. Administer vasoactive drugs: Peripheral epinephrine or norepinephrine 0.1mcg/kg/min. |
| State 5:  Resolution | HR 140  BP 80/45  RR per vent settings  SpO2 98%  Exam findings:  Patient attempting weak cry. Peripheral pulses strong. Extremities warm. | Shock is reversed after appropriate fluid resuscitation (60cc/kg) and pressor therapy.  Operator:  BP and HR begin to normalize after starting pressors. Patient is stable on ventilator and epinephrine/norepi gtt.  End scenario. |  |

Ideal Scenario Flow

The learners enter the room to find an ill-appearing, under-active infant. They immediately place the patient on bedside monitors and recognize that the patient is febrile, tachycardic, and hypotensive. Supplemental oxygen is provided, IV access is obtained, and an IV fluid bolus is ordered. A septic workup is sent, including blood culture, and IV antibiotics are ordered. Despite interventions, the patient’s status continues to decline. As he becomes less responsive, his respiratory rate declines to 15. Learners initiate bag-mask ventilation and prepare for intubation. Intubation is then attempted and successful with post-intubation SpO2 >90%. A total of 60 cc/kg of crystalloid fluid is given, however the patient’s shock does not respond to fluid boluses. Blood pressure continues to drop, extremities become mottled and cool, and peripheral pulses are weak and thready. Learners order vasoactive medication, which reverses the patient’s state of shock. Patient is stable on mechanical ventilation and pressor therapy. Transfer to the pediatric intensive care unit is initiated.

Anticipated Management Mistakes

1. Failure to bag-mask ventilate: Many of our learners recognized the need for intubation, however we found that most did not provide bag-mask ventilation while preparing to intubate or in between intubation attempts, leading to further respiratory decline. We found it helpful to bring up this learning point during debriefing after the first simulation case. Almost all learners then recognized the need for bag-mask ventilation in the second and third simulation cases.
2. Failure to provide a total of 60 cc/kg of fluid resuscitation prior to initiation of pressor support.: Many of our learners failed to give a total of three 20 cc/kg boluses prior to initiating pressor therapy. We discussed appropriate fluid resuscitation and the definition of fluid refractory shock in our debriefing sessions, with improvement in performance on subsequent simulations.
3. Failure to recognize sepsis, draw blood cultures, and start antibiotics in a timely manner. Many of our learners would get very focused on the patient’s other concerning vital signs, and not recognize or understand the implication of the fever, leading to a delay in obtaining a blood culture and starting antibiotic therapy. Again, this was discussed in our debriefing sessions, with improvement in performance on subsequent simulations.
