# Supplementary material for: Identification and Management of Pediatric Sepsis: A Medical Student Curricular Supplement for PICU and NICU Rotations
Source: MedEdPORTAL. 2021 Apr 23;17:11142. doi: 10.15766/mep_2374-8265.11142 (PMC8063627; doi:10.15766/mep_2374-8265.11142)
Supplement: Supplementary file 1 — Pre- & Posttest.docxModule 1 - Pediatric Shock.pptxScript 1 - Pediatric Shock.docxModule 2 - Pediatric Sepsis.pptxScript 2 - Pediatric Sepsis.docxModule 3 - Management of Sepsis & Septic Shock.pptxScript 3 - Management of Sepsis & Septic Shock. docxModule 4 - Hemodynamics & Pressor Support.pptxScript 4 - Hemodynamics & Pressor Support.docxSimulation Case 1.docxSimulation Case 2.docxSimulation Case 3.docxPostsimulation Review Quiz.pptx [file mep_2374-8265.11142-s001.zip › M. Postsimulation Review Quiz.pptx]

## Slide 1
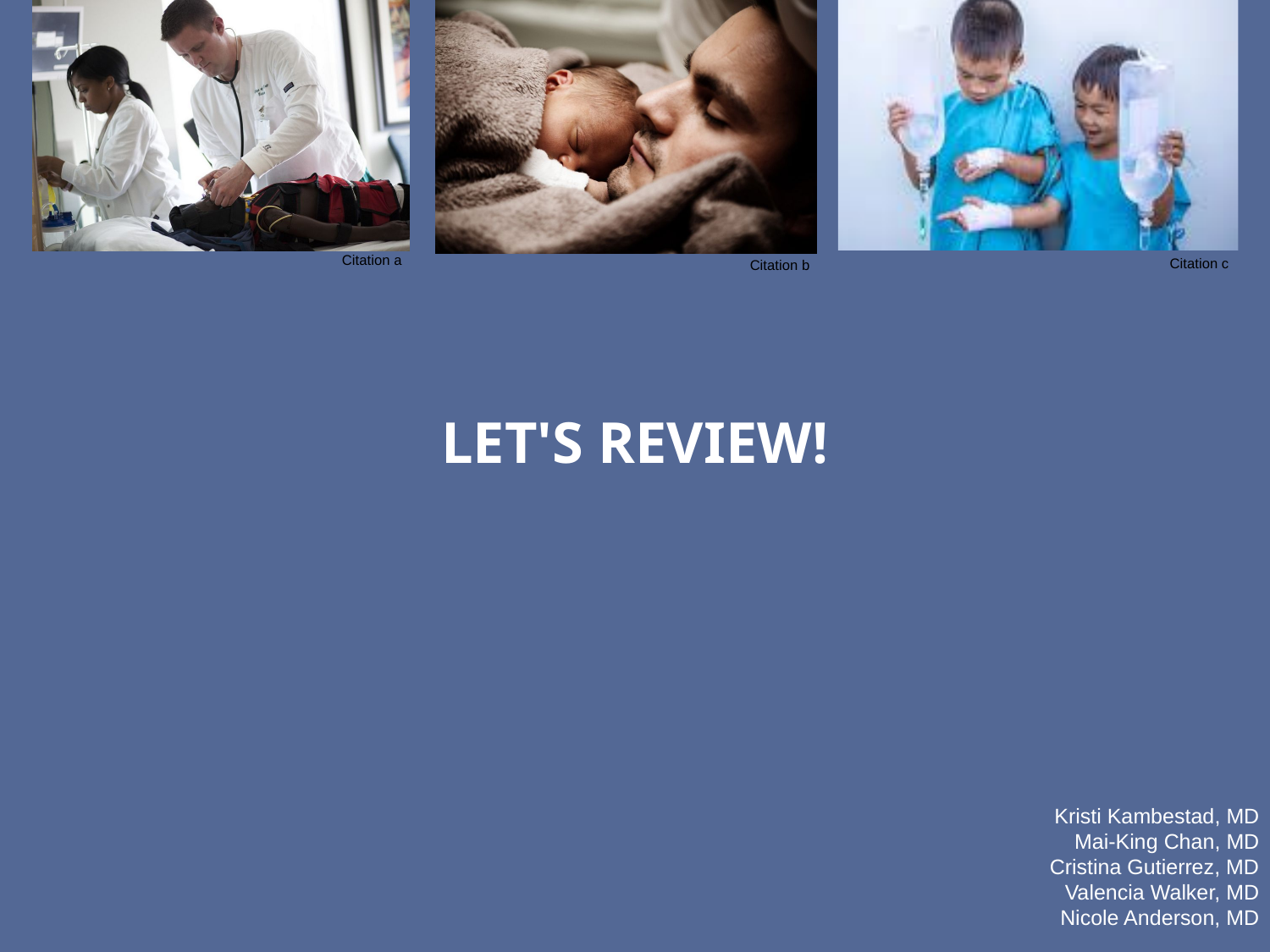

Citation a
Citation c
Citation b
LET'S REVIEW!
Kristi Kambestad, MD
Mai-King Chan, MD
Cristina Gutierrez, MD
Valencia Walker, MD
Nicole Anderson, MD

## Slide 2
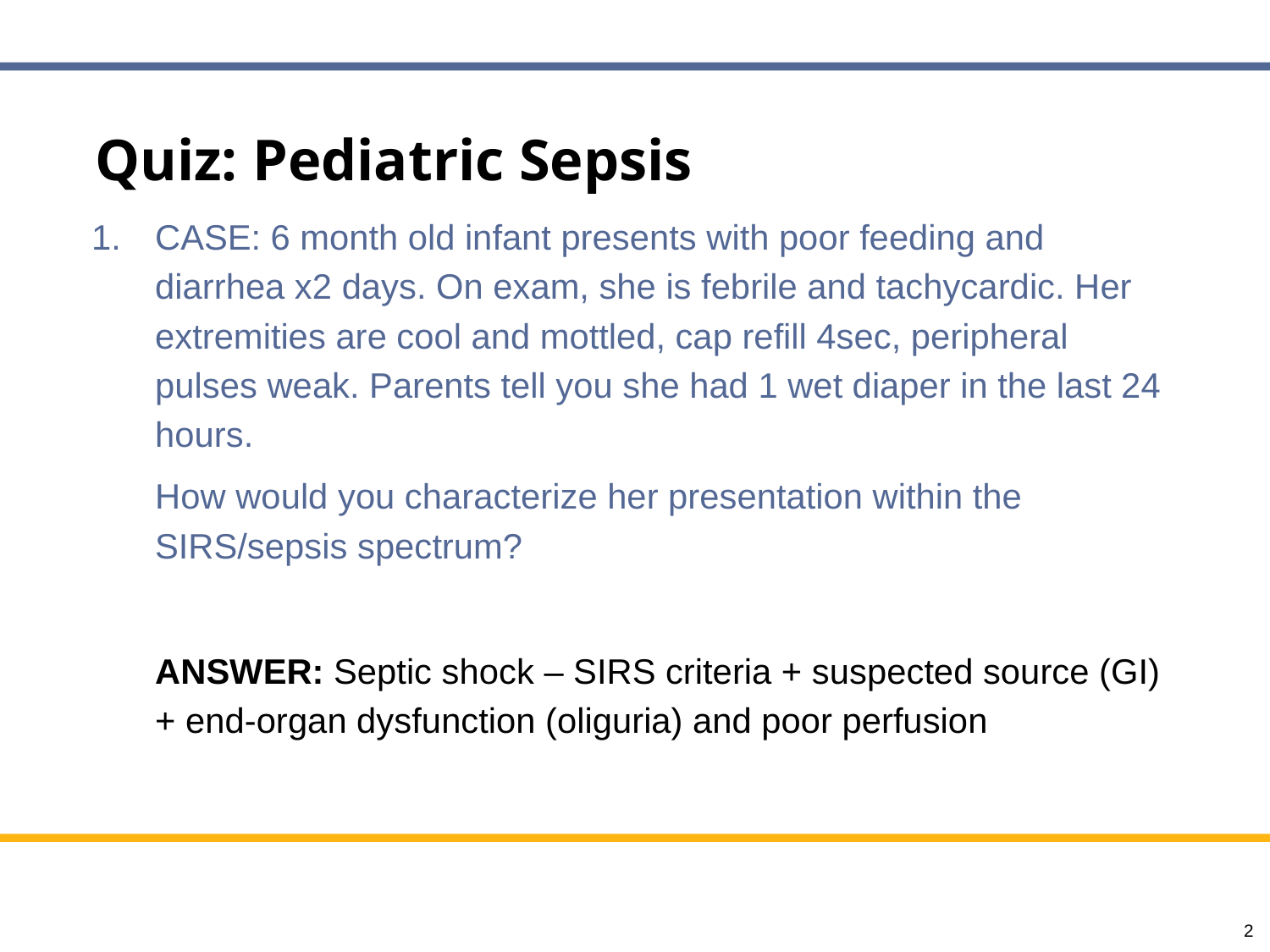

Quiz: Pediatric Sepsis
CASE: 6 month old infant presents with poor feeding and diarrhea x2 days. On exam, she is febrile and tachycardic. Her extremities are cool and mottled, cap refill 4sec, peripheral pulses weak. Parents tell you she had 1 wet diaper in the last 24 hours.
	How would you characterize her presentation within the SIRS/sepsis spectrum?
	ANSWER: Septic shock – SIRS criteria + suspected source (GI) + end-organ dysfunction (oliguria) and poor perfusion
2

## Slide 3
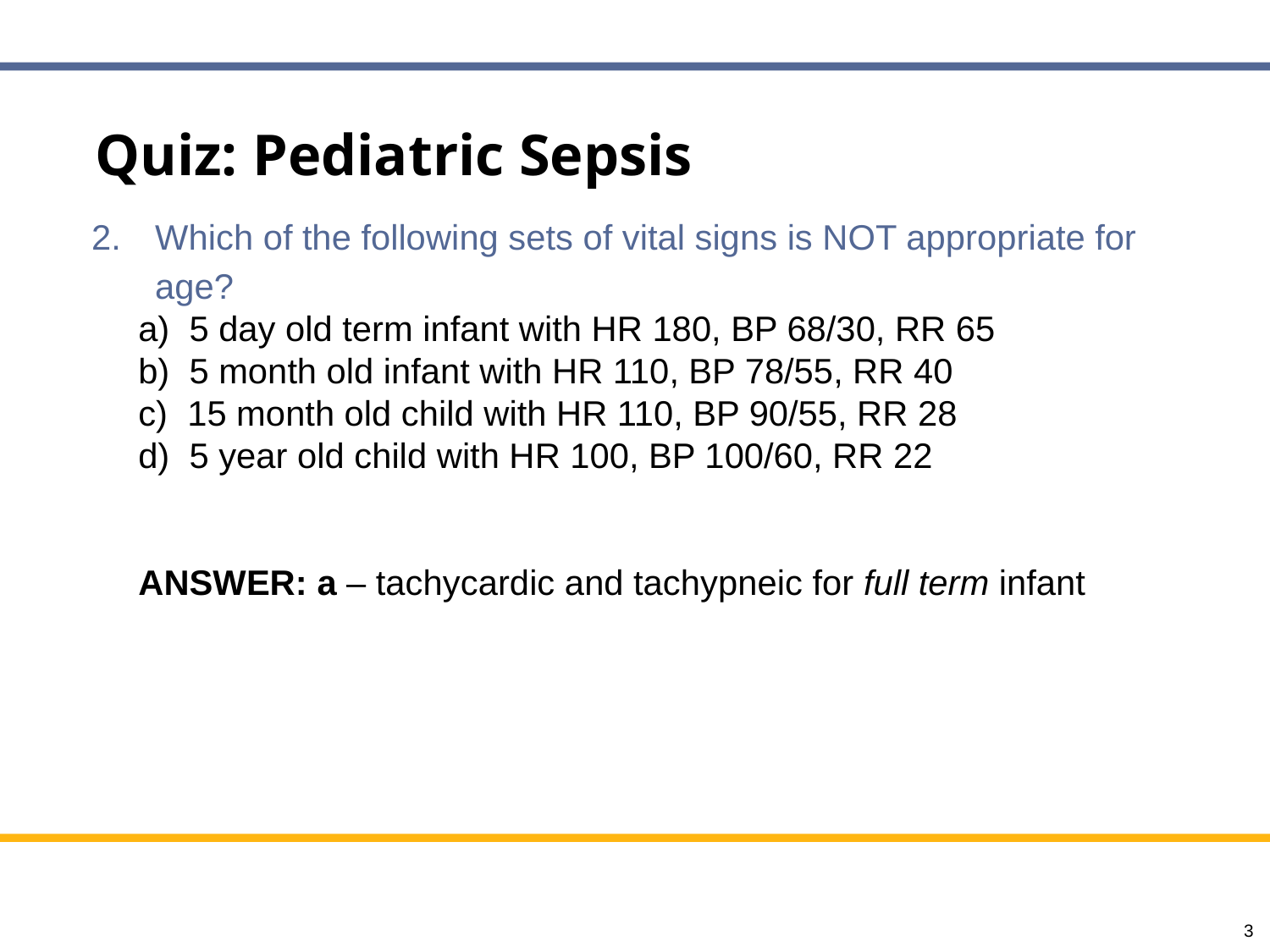

Quiz: Pediatric Sepsis
2.	Which of the following sets of vital signs is NOT appropriate for age?
a) 5 day old term infant with HR 180, BP 68/30, RR 65
b) 5 month old infant with HR 110, BP 78/55, RR 40
c) 15 month old child with HR 110, BP 90/55, RR 28
d) 5 year old child with HR 100, BP 100/60, RR 22
ANSWER: a – tachycardic and tachypneic for full term infant
3

## Slide 4
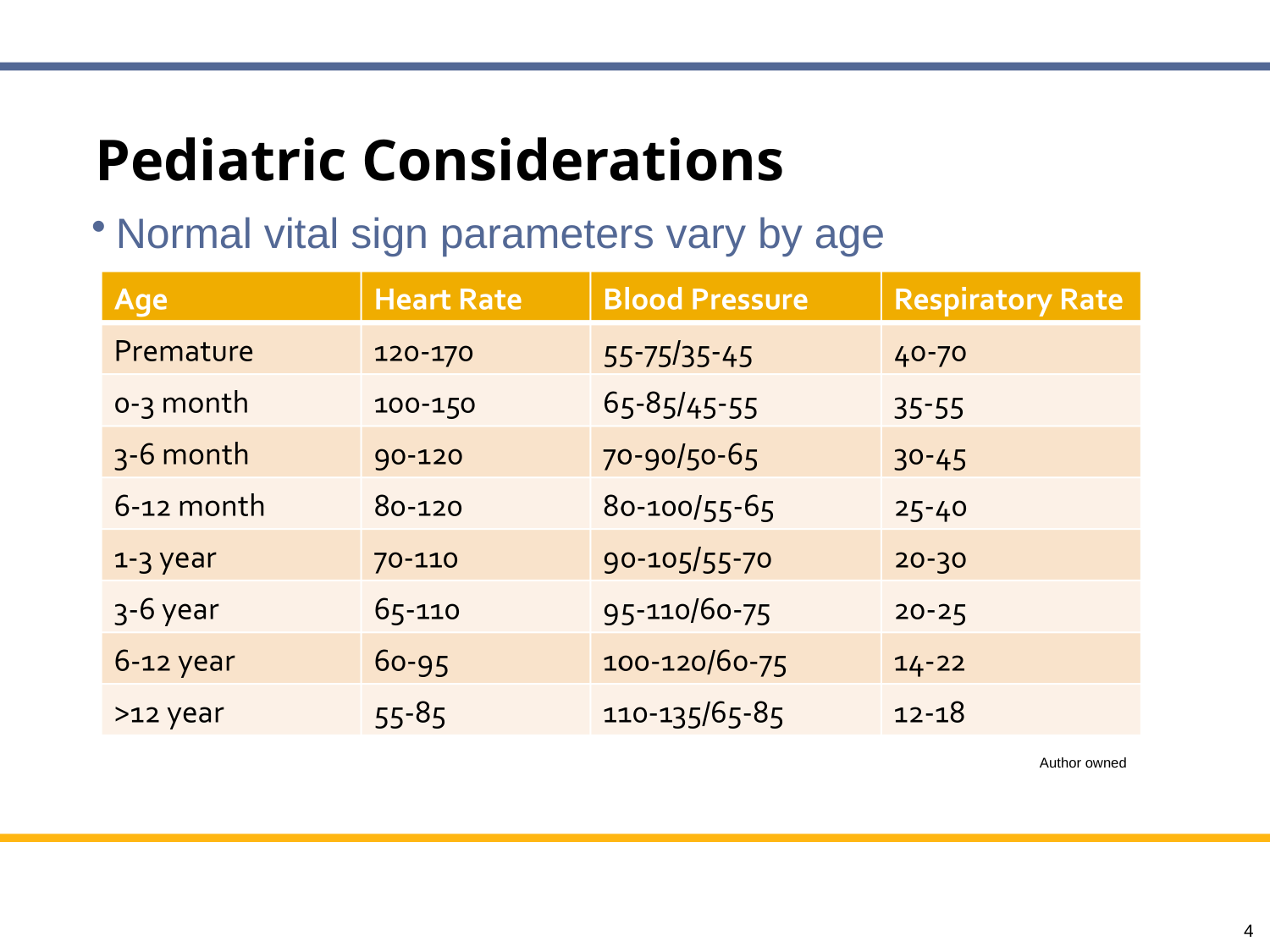

Pediatric Considerations
Normal vital sign parameters vary by age
Author owned
4

## Slide 5
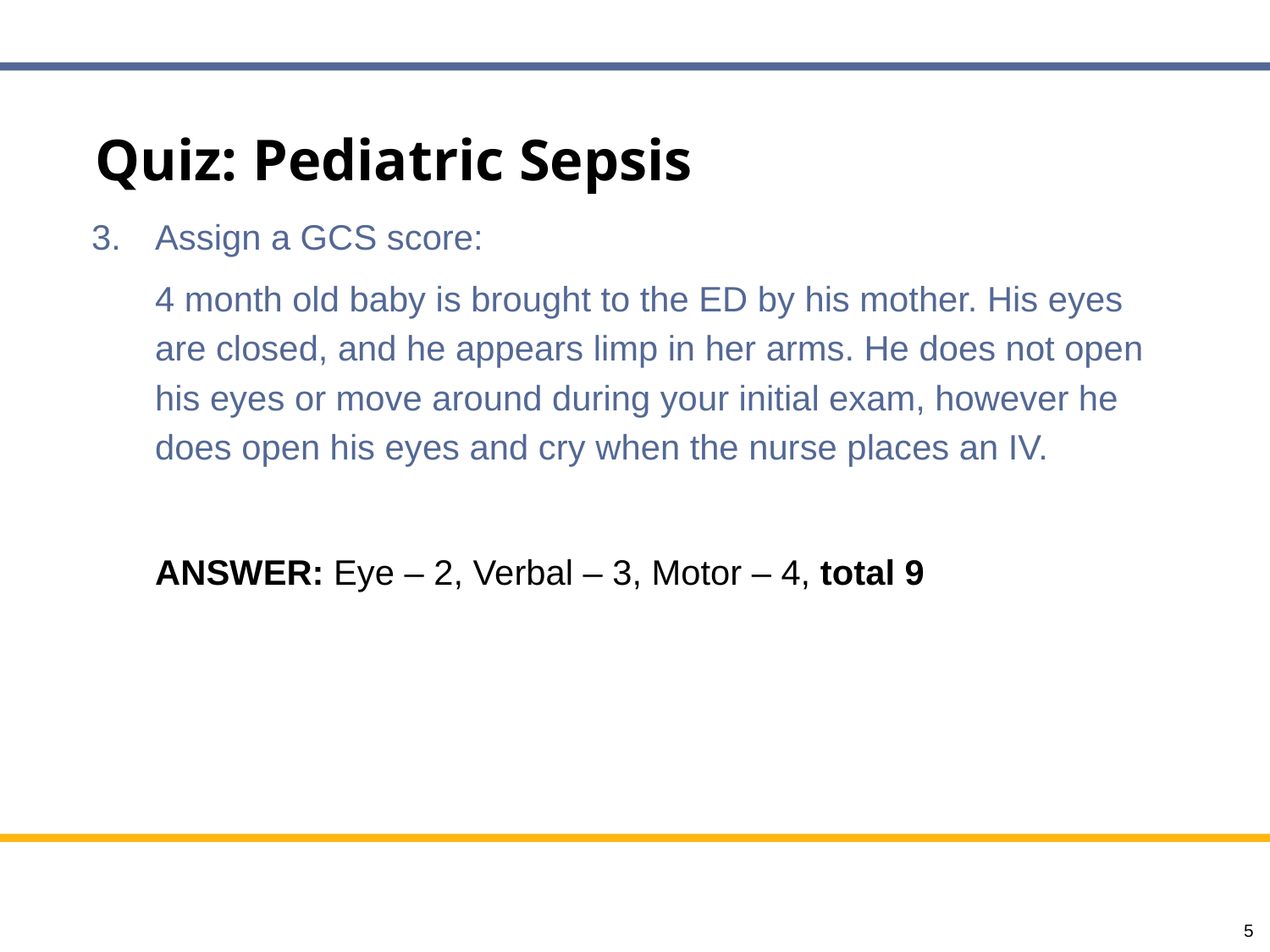

Quiz: Pediatric Sepsis
Assign a GCS score:
	4 month old baby is brought to the ED by his mother. His eyes are closed, and he appears limp in her arms. He does not open his eyes or move around during your initial exam, however he does open his eyes and cry when the nurse places an IV.
	ANSWER: Eye – 2, Verbal – 3, Motor – 4, total 9
5

## Slide 6
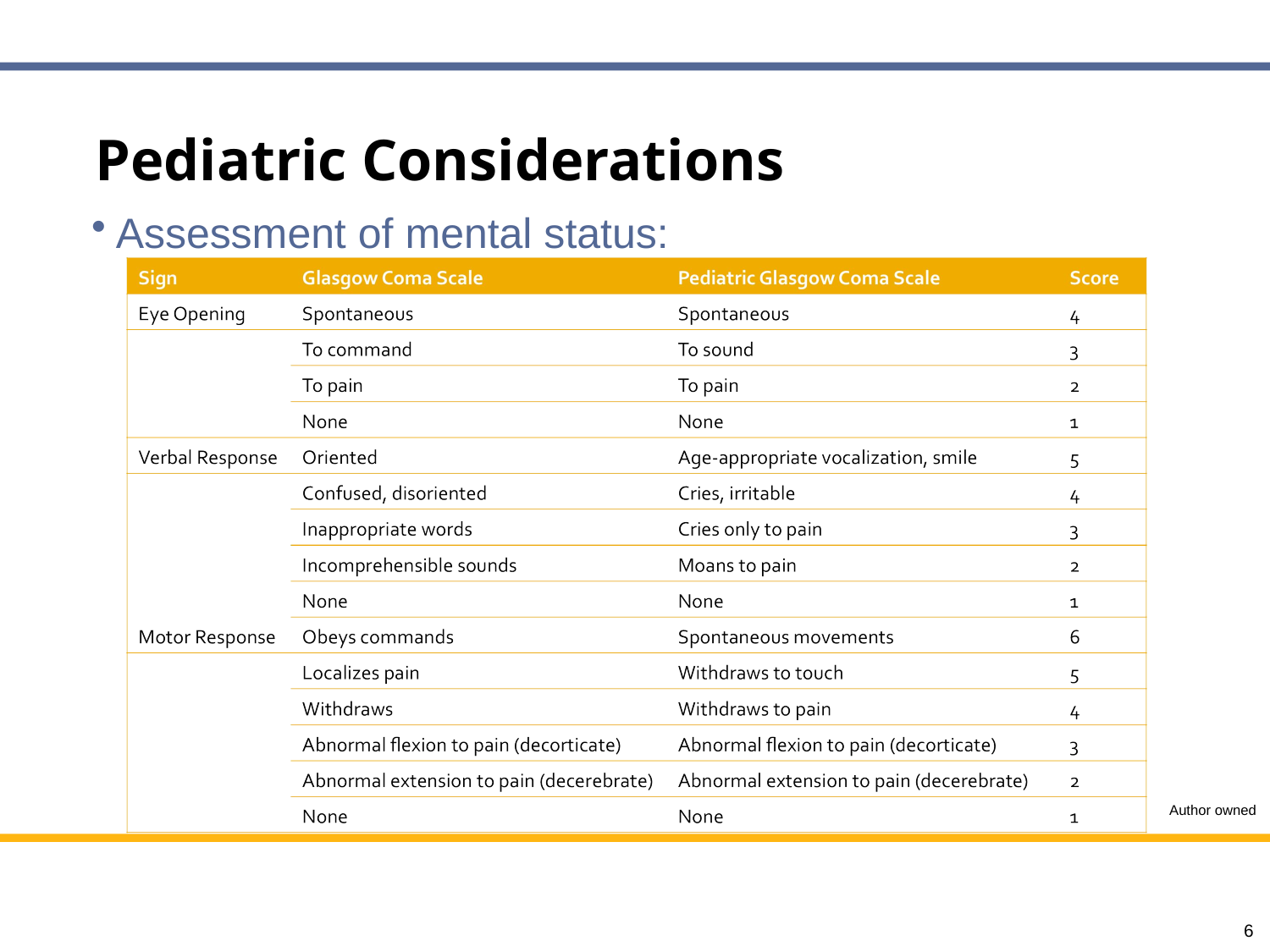

Pediatric Considerations
Assessment of mental status:
Author owned
6

## Slide 7
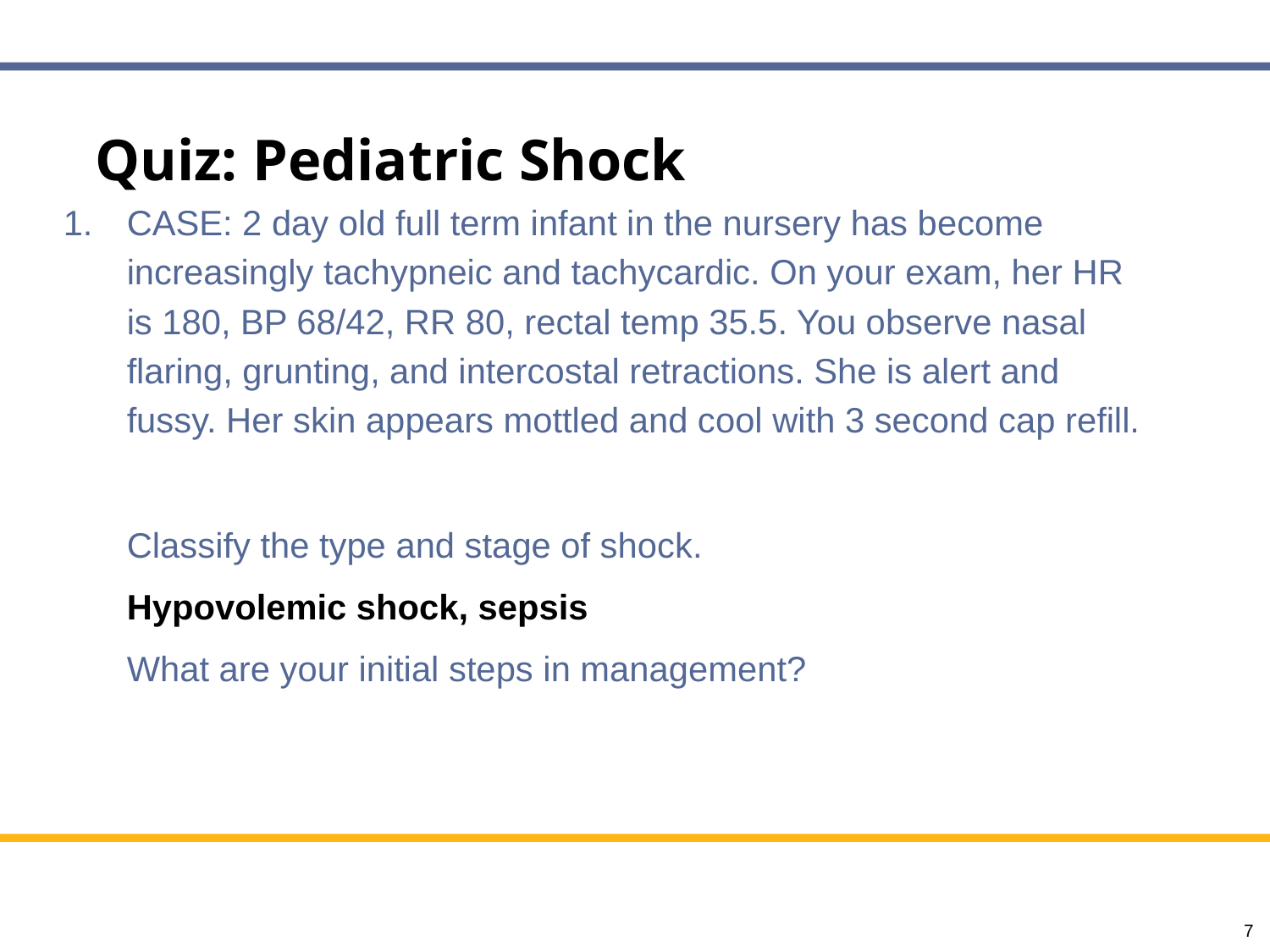

Quiz: Pediatric Shock
CASE: 2 day old full term infant in the nursery has become increasingly tachypneic and tachycardic. On your exam, her HR is 180, BP 68/42, RR 80, rectal temp 35.5. You observe nasal flaring, grunting, and intercostal retractions. She is alert and fussy. Her skin appears mottled and cool with 3 second cap refill.
	Classify the type and stage of shock.
	Hypovolemic shock, sepsis
	What are your initial steps in management?
7

## Slide 8
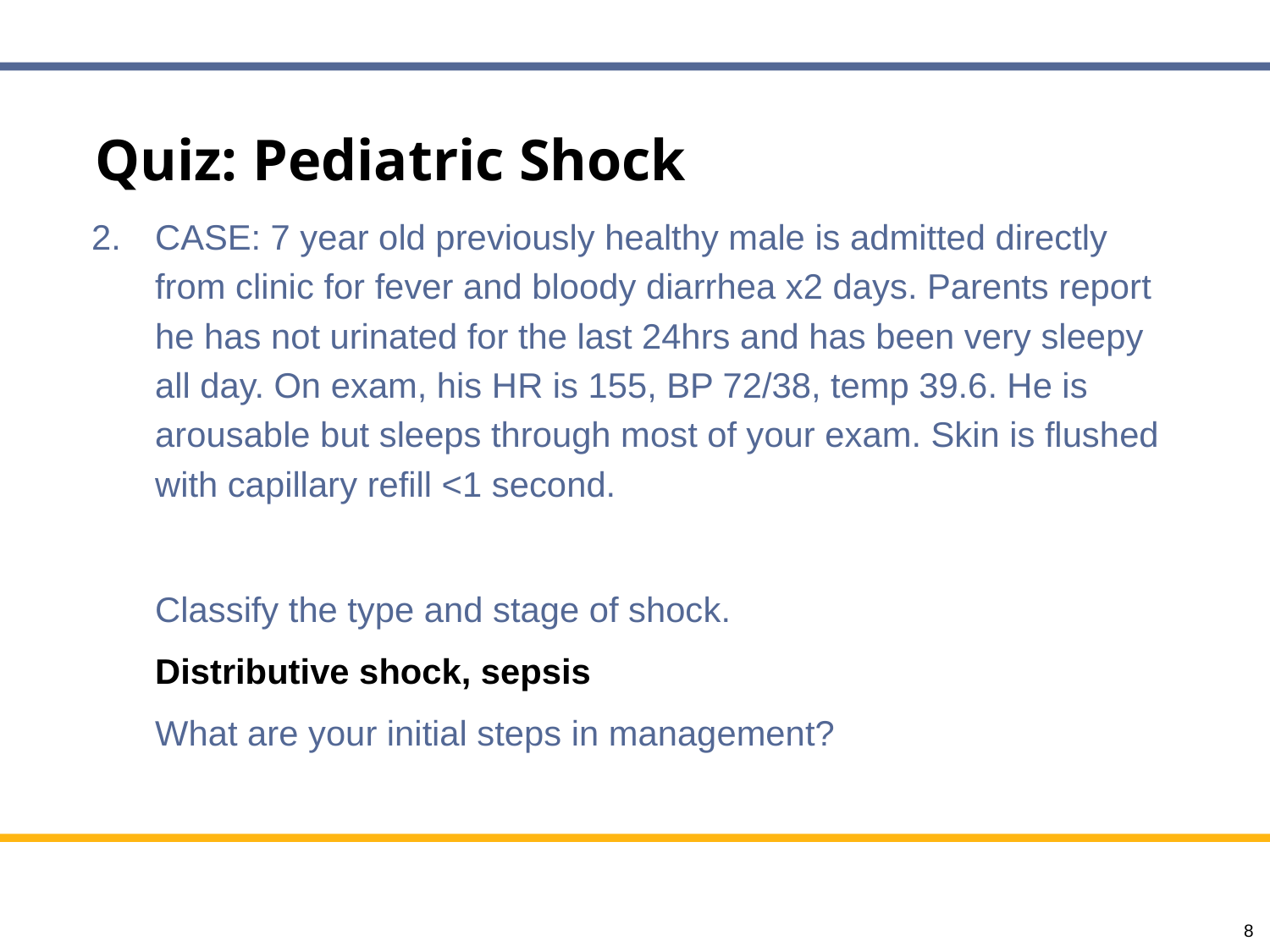

Quiz: Pediatric Shock
2.	CASE: 7 year old previously healthy male is admitted directly from clinic for fever and bloody diarrhea x2 days. Parents report he has not urinated for the last 24hrs and has been very sleepy all day. On exam, his HR is 155, BP 72/38, temp 39.6. He is arousable but sleeps through most of your exam. Skin is flushed with capillary refill <1 second.
	Classify the type and stage of shock.
	Distributive shock, sepsis
	What are your initial steps in management?
8

## Slide 9
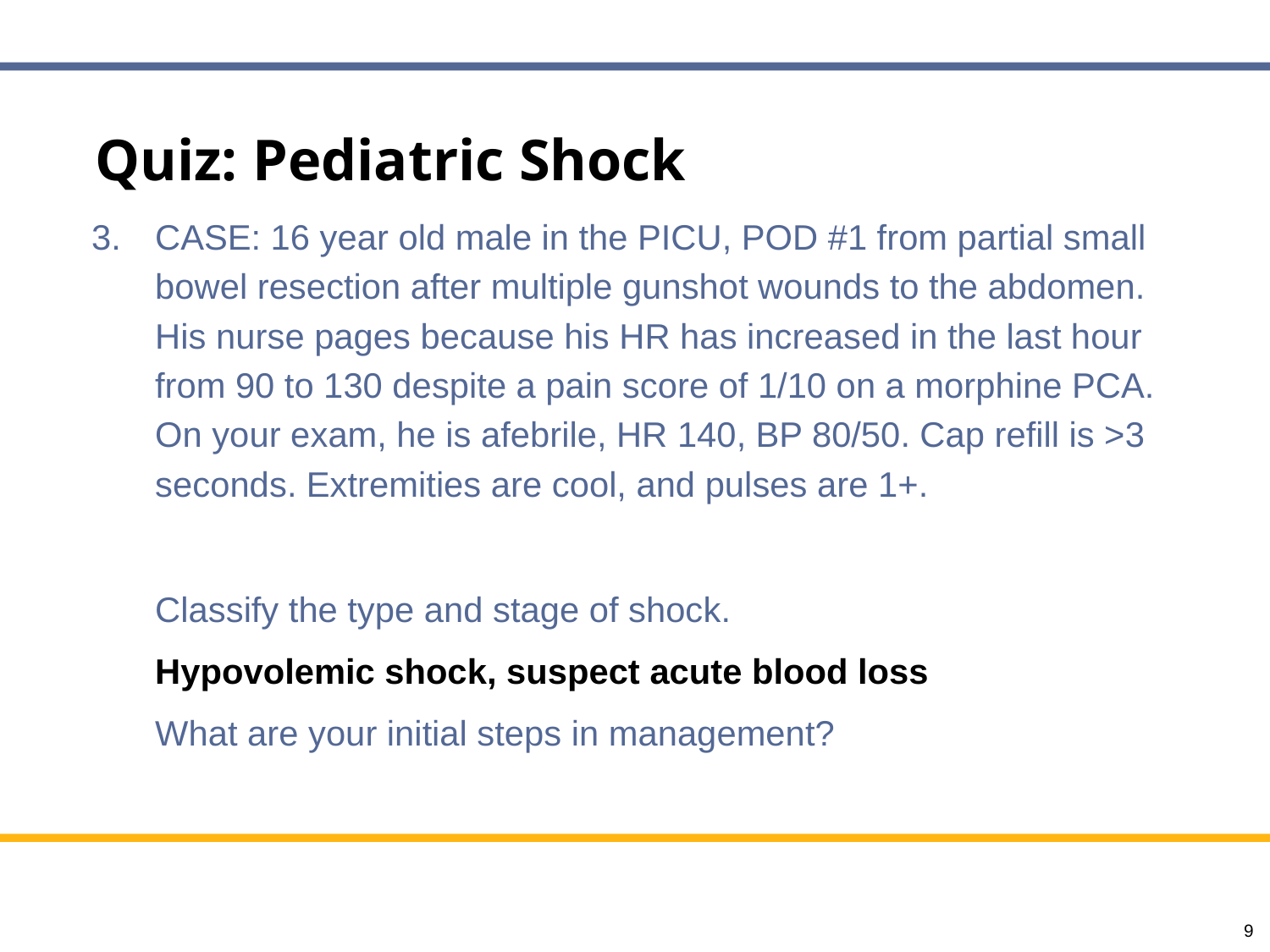

Quiz: Pediatric Shock
3.	CASE: 16 year old male in the PICU, POD #1 from partial small bowel resection after multiple gunshot wounds to the abdomen. His nurse pages because his HR has increased in the last hour from 90 to 130 despite a pain score of 1/10 on a morphine PCA. On your exam, he is afebrile, HR 140, BP 80/50. Cap refill is >3 seconds. Extremities are cool, and pulses are 1+.
	Classify the type and stage of shock.
	Hypovolemic shock, suspect acute blood loss
	What are your initial steps in management?
9

## Slide 10
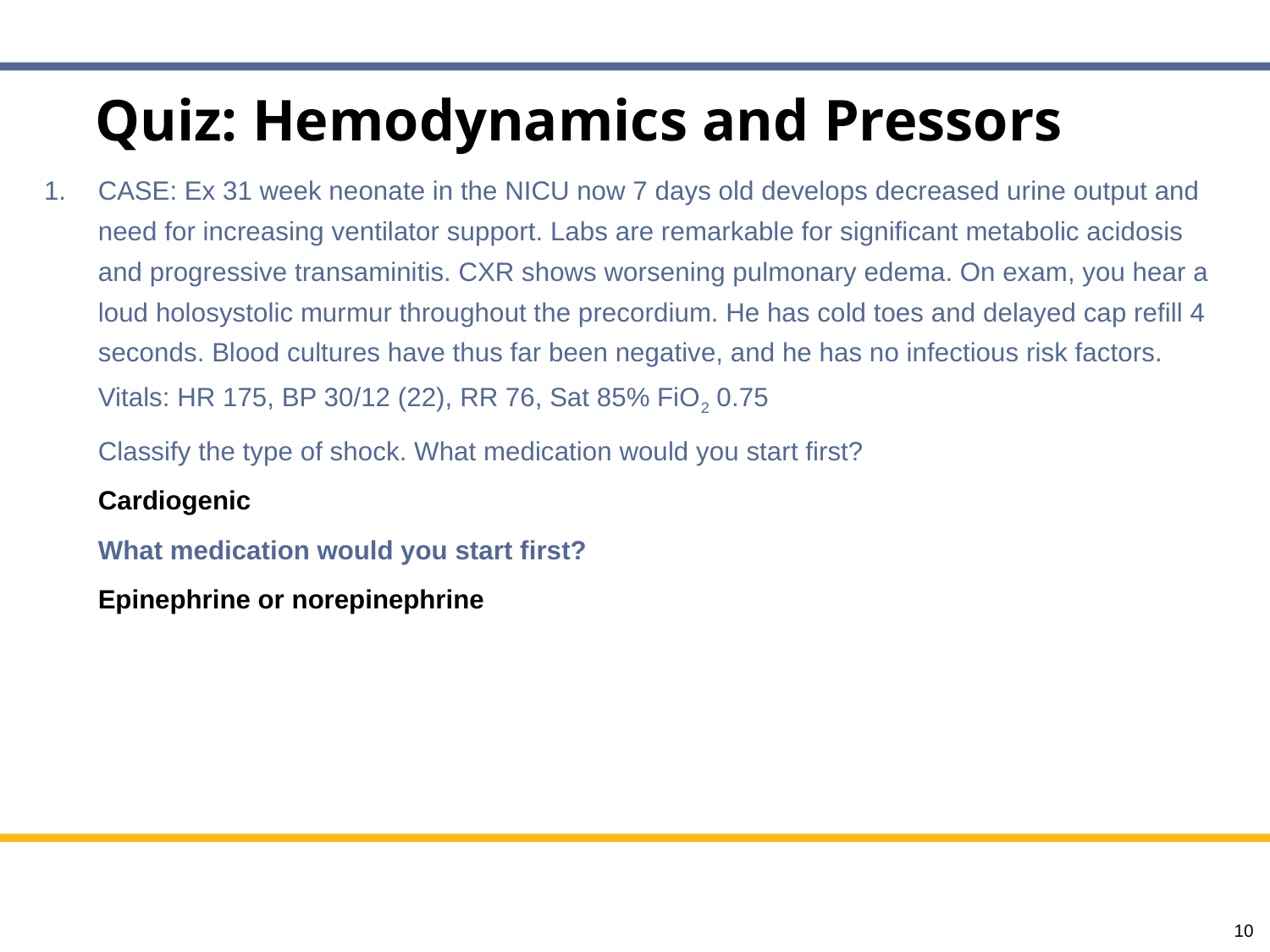

Quiz: Hemodynamics and Pressors
CASE: Ex 31 week neonate in the NICU now 7 days old develops decreased urine output and need for increasing ventilator support. Labs are remarkable for significant metabolic acidosis and progressive transaminitis. CXR shows worsening pulmonary edema. On exam, you hear a loud holosystolic murmur throughout the precordium. He has cold toes and delayed cap refill 4 seconds. Blood cultures have thus far been negative, and he has no infectious risk factors.
	Vitals: HR 175, BP 30/12 (22), RR 76, Sat 85% FiO2 0.75
	Classify the type of shock. What medication would you start first?
	Cardiogenic
	What medication would you start first?
	Epinephrine or norepinephrine
10

## Slide 11
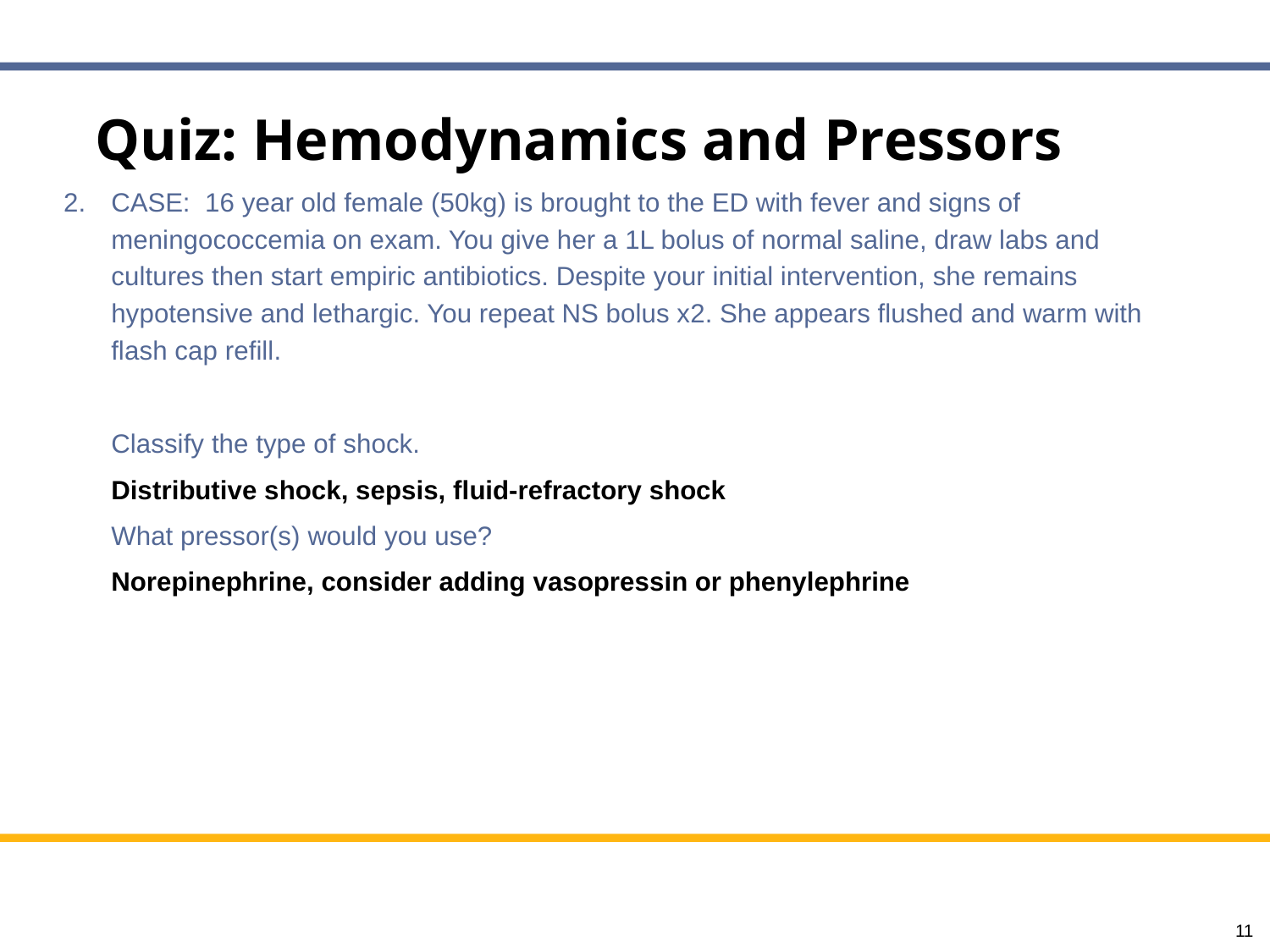

Quiz: Hemodynamics and Pressors
CASE: 16 year old female (50kg) is brought to the ED with fever and signs of meningococcemia on exam. You give her a 1L bolus of normal saline, draw labs and cultures then start empiric antibiotics. Despite your initial intervention, she remains hypotensive and lethargic. You repeat NS bolus x2. She appears flushed and warm with flash cap refill.
	Classify the type of shock.
	Distributive shock, sepsis, fluid-refractory shock
	What pressor(s) would you use?
	Norepinephrine, consider adding vasopressin or phenylephrine
11
